# Discovery of a Novel Shared Variant Among *RTEL1* Gene and *RTEL1-TNFRSF6B* lncRNA at Chromosome 20q13.33 in Familial Progressive Myoclonus Epilepsy

**DOI:** 10.1155/2024/7518528

**Published:** 2024-08-10

**Authors:** Sima Chaudhari, Lavanya Prakash Acharya, Dushyanth Babu Jasti, Akshay Pramod Ware, Sankar Prasad Gorthi, Kapaettu Satyamoorthy

**Affiliations:** ^1^ Department of Cell and Molecular Biology Manipal School of Life Sciences Manipal Academy of Higher Education 576104, Manipal, Karnataka, India; ^2^ Department of Neurology Kasturba Medical College 576104, Manipal, Karnataka, India; ^3^ Department of Bioinformatics Manipal School of Life Sciences Manipal Academy of Higher Education 576104, Manipal, Karnataka, India; ^4^ Department of Neurology Bharati Hospital and Research Center Bharati Vidyapeeth (Deemed to Be University) Medical College and Hospital, Dhankawadi 411043, Pune, Maharashtra, India; ^5^ SDM College of Medical Sciences and Hospital Shri Dharmasthala Manjunatheshwara (SDM) University, Manjushree Nagar, Sattur 580009, Dharwad, Karnataka, India

**Keywords:** ataxia, familial, mitochondrial genome sequencing, mutation, progressive myoclonic epilepsy, whole exome sequencing

## Abstract

**Background:** Progressive myoclonus epilepsy (PME) is a neurodegenerative disorder marked by recurrent seizures and progressive myoclonus. To date, based on the phenotypes and causal genes, more than 40 subtypes of PMEs have been identified, and more remain to be characterized. Our study is aimed at identifying the aberrant gene(s) possibly associated with PMEs in two siblings born to asymptomatic parents, in the absence of known genetic mutations.

**Methods:** Clinical assessments and molecular analyses, such as the repeat expansion test for *CSTB*; SCA1, 2, 3, 6, and 7; whole exome sequencing (WES); and mitochondrial genome sequencing coupled with computational analysis, were performed.

**Results:** A family-based segregation analysis of WES data was performed to identify novel genes associated with PMEs. The potassium channel, *KCNH8* [c.298T>C; (p.Tyr100His)], a DNA repair gene, regulator of telomere elongation helicase 1 (*RTEL1*) [c.691G>T; (p.Asp231Tyr)] and long noncoding RNA, *RTEL1-TNFRSF6B* [chr20:62298898_G>T; NR_037882.1, hg19] were among the candidate genes that were found to be associated with PMEs. These homozygous variations in siblings belong to genes with a loss-of-function intolerant (pLI) score of ≤ 0.86, expected to be detrimental by multiple computational analyses, and were heterozygous in parents. Additionally, computational analysis and the expression of *RTEL1* and *RTEL1-TNFRSF6B* revealed that *RTEL1-TNFRSF6B* may modulate *RTEL1* via hsa-miR-3529-3p. In the patient with the severe phenotype, a further deleterious mutation in *SLC22A17* was identified. No de novo variants specific to these probands were identified in the mitochondrial genome.

**Conclusions:** Our study is the first to report variants in *KCNH8*, *RTEL1*, and *RTEL1-TNFRSF6B* among PME cases. These genes when characterized fully may shed light on pathogenicity and have the potential to be used in the diagnosis of PME.

## 1. Introduction

Progressive myoclonus epilepsies (PMEs) are a rare but distinct group of neurological disorders typified by recurring seizures and progressive myoclonus, along with other neurological phenotypes such as ataxia and dementia [1, 2]. It accounts for approximately 1% of the epileptic syndromes prevalent throughout the world [2]. PMEs are genetic disorders, and the majority of them are of autosomal recessive inheritance, yet some are inherited in an autosomal dominant fashion or originate from mitochondria [1]. The heterogeneity in the clinical phenotypes and underlying genetic defects has paved the way to define more than 40 types of PMEs [2]. Although some PME types have distinguishing characteristics, the rarity and overlap of features among the other PMEs complicate the diagnosis, especially at an early-onset stage [2, 3]. Defects in genes with lysosomal function or protein degradation (*CSTB*, *NEU1*, *ASAH1*, *SCARB2*, *GBA*, *NHLRC1*, *PPT1*, *TPP1*, *CLN3*, *CLN5*, *CLN6*, *CTSD*, *GRN*, and *CTSF*), macromolecule trafficking (*GOSR2*, *DNAJC5*, *MFSD8*, *CLN8*, *TBC1D24*, *PRICKLE1*, and *SLC7A6OS*), potassium channel (*KCNC1*, *KCTD7*), neuronal development and function (*SERPINI1*, *SEMA6B*, and *AFG3L2*), DNA metabolism (*POLG*, *ATN1*, *PRICKLE1*, and *PRDM8*), metabolism (*EPM2A*, *CERS1*, *PPT1*, and *CLN8*), protein folding (*SACS*, *DNAJC5*), and mitochondrial function (*POLG*, *MTND5*, *MTTK*, *MTTL1*, *MTTH*, *MTTS1*, *MTTS2*, and *MTTF*) are all associated with PMEs [4]. However, the underpinning genetic defects in many individuals with clinically suspected PMEs remain unknown [5]. The majority of PMEs are the result of point mutations or frameshift mutations in the coding sequences of associated genes, excluding Unverricht–Lundborg disease (ULD), which results from abnormal expansion of the dodecamer in the 5′-UTR (untranslated region), and dentatorubral–pallidoluysian atrophy (DRPLA), which shows CAG repeat expansion in the *ATN1* gene. Large deletions (deletions of sequence > 100 bp) have also been reported in some PMEs [6]. All these factors make the precise and timely diagnosis of PMEs cumbersome.

The integration of whole exome sequencing (WES) approaches, focusing on the most informative region of the genome, can provide fast and detailed genetic information for clinically complex and genetically heterogeneous disorders such as PMEs. We present two siblings with cardinal features of PMEs born to a healthy couple in second-degree consanguineous marriage. The age of onset at infancy with myoclonic seizures in the foreground, followed by progressive cognitive regression and ataxia, as well as the absence of occipital seizures and pyramidal and extrapyramidal symptoms, implicated the siblings as ULD or KCTD7-related PME (EPM3). Differential diagnosis based on the phenotypes and molecular analysis excluded the known genetic basis, including mutations in the *CSTB* and *KCTD7* genes. WES of the family followed by family-based segregation analysis and extensive computational analysis revealed pathogenic variants in novel genes that were likely to be associated with PME phenotypes.

## 2. Materials and Methods

### 2.1. Patient Recruitment and Ethical Considerations

The subjects were identified and recruited during the study approved by the Institutional Ethical Committee of Kasturba Medical College, Manipal (registry no. IEC365/2017, CTRI/2017/07/00904). Blood samples and clinical details of probands as well as their parents were collected upon obtaining the written informed consent.

### 2.2. WES and Data Analysis

DNA extraction, WES, preprocessing, and annotation of sequenced data were performed as described earlier by Chaudhari et al. [7]. All the nonsynonymous mutations with a minor allele frequency of < 1% were further subjected to variant prioritization filtration criteria such as homozygous and heterozygous mutations, variants with the Phred score > 20, coverage of > 10×, absence in the UCSC common SNP database, presence within the homopolymer region (< 4), and genes expressed in the brain. The variants that were homozygous in the proband but heterozygous or absent in the parents were shortlisted. The heterozygous variants were shortlisted only if inherited as compound heterozygous in probands but present as heterozygous variants in their parents. Variants in the causal genes of neurodegenerative disorders were assessed. Further, the pathogenicity of the variants was evaluated with Variant Annotation and Filter Tool (VarAFT) software and UMD-Predictor [8].

### 2.3. Copy Number Variation (CNV) Analysis From WES Data

CNV was analyzed using EXCAVATOR2 [9] and further annotated using ClassifyCNV [10] as described previously by Chaudhari et al. [7].

### 2.4. Mitochondrial Genome Sequencing

Sequencing of the mitochondrial genome was performed as discussed by Mani et al. on the Ion Torrent Personal Genome Machine (PGM) System (Thermo Fisher Scientific, United States) platform [11]. Alignment, haplogroup prediction, and annotation were performed for sequenced data using the Mtool box [12]. The variants were segregated either as “haplogroup specific” or “private” on the basis of their haplogroup. The haplogroup-specific variants were shortlisted for further analysis if previously associated with disease, while the nonsynonymous private variants with a heteroplasmy fraction > 0.01 and a disease score of > 0.4311 or nucleotide variability of < 0.0026 were shortlisted for further analysis [11]. The impact of nonsynonymous private variants was identified by analysis of MitoMap (http://www.mitomap.org) and MitImpact databases (https://mitimpact.css-mendel.it/). Furthermore, the variants absent in the mother's mitochondrial genome sequencing data were further analyzed.

### 2.5. Primer Designing

Primers were designed using Primer-BLAST (https://www.ncbi.nlm.nih.gov/tools/primer-blast/), and the specificity of the designed primers was checked using the UCSC in silico PCR tool (http://www.genome.ucsc.edu/cgi-bin/hgPcr). For the expression analysis, the primers were designed with the criteria that at least one intron on the corresponding genomic DNA separates the primer pair.

### 2.6. Sanger Sequencing

The primers listed in Supporting Information 4 were used to amplify the targeted regions. For *CSTB*, primer sets reported by Joensuu et al. that amplified a 193 bp region of the *CSTB* gene harboring a dodecamer repeat expansion site were used [13]. Sanger sequencing was performed on the ABI 3130 genetic analyzer (Applied Biosystems, Monza, Italy) and further sequenced with a big dye terminator kit.

### 2.7. SCA Analysis

SCA1, SCA2, SCA3, SCA6, and SCA7 tests were performed by PCR amplification and capillary electrophoresis as described by Dorschner, Barden, and Stephens [14]. The fluorescently labeled amplicons were detected in the ABI 3130 genetic analyzer (Applied Biosystems, Monza, Italy) and analyzed using GeneScan software.

### 2.8. Telomere Length Assay

Telomere length was analyzed by following the qPCR method of Cawthon [15]. SYBR Green chemistry was employed for the assay, and single-copy albumin was used as an internal control. The cycle threshold (*C*_T_) value for each target was estimated from the amplification curve using QuantStudio 6Pro software (Thermo Fisher Scientific, United States). Relative telomere length was calculated in each sample with reference to age- and sex-matched controls.

### 2.9. RNA Extraction and cDNA Conversion

Total RNA was isolated from the blood using a TRI reagent (MRC, United States) as per the manufacturer's protocol. Further, the high-capacity cDNA reverse transcription kit (Applied Biosystems, United States) was used for the conversion of total RNA isolated from blood specimens as per the manufacturer's protocol.

### 2.10. Real-Time PCR for the Expression Analysis

SYBR Green chemistry was utilized for the expression analysis. The details of the primers used for the expression analysis of regulator of telomere elongation helicase 1 (*RTEL1*) and *RTEL1-TNFRSF6B* are listed in Supporting Information 4. The forward and reverse primers used for the expression analysis of *RTEL1* are complementary to region of Exon 13 (Exon 13 of *RTEL1-TNFRSSF6B*) and Exon 15 (Exon 15 of *RTEL1-TNFRSSF6B*), respectively. Hence, this primer pair can measure the expression of both *RTEL1* and *RTEL1-TNFRSSF6B* at the RNA level. As *RTEL1-TNFRSSF6B* is a read-through transcript overlapping *RTEL1* and *TNFRSF6B* genes, the primers were designed such that the forward primer is complementary to the region of Exon 35 of *RTEL1-TNFRSF6B* (Exon 35 of *RTEL1*) and the reverse primer is complementary to the region of Exon 36 of *RTEL1-TNFRSF6B* (Exon 1 of *TNFRSF6B*). Hence, the amplicon obtained from this primer pair is specifically from *RTEL1-TNFRSSF6B*.

The *ACTB* was considered as a reference gene. The *C*_T_ value for each target gene was estimated from the amplification curve using QuantStudio 6Pro software (Thermo Fisher Scientific, United States). Δ*C*_T_ was calculated by subtracting *C*_T_ of the reference gene from *C*_T_ of the test gene. Relative quantity (RQ) was estimated with the formula 2^−*ΔΔ*Cт^, where ΔΔC_T_ = ΔC_T_ of sample/ΔC_T_ of control sample. The *C*_T_ value of the mother was used as a control sample. An unpaired *t* test was used for statistical analysis.

### 2.11. Secondary Structure of RNA and Splice Site Prediction

The secondary structure of *RTEL1-TNFRSF6B* RNA (NR_037882.1) with and without mutation was predicted using the RNAfold web server (http://rna.tbi.univie.ac.at/cgi-bin/RNAWebSuite/RNAfold.cgi). SpliceAI (https://spliceailookup.broadinstitute.org/) and Spliceator (https://www.lbgi.fr/spliceator/) tools were used for prediction of the gain or loss of a splice site donor or acceptor.

### 2.12. Computational Analysis

In silico analysis for conservation of affected amino acid residues of candidate proteins and their visualization was performed as described earlier by Chaudhari et al. [7]. The expression of *RTEL1-TNFRSF6B* in the brain was confirmed in the circAtlas 2.0 database (http://www.ngdc.cncb.ac.cn/circatlas/search.php). Interacting partners of *RTEL1-TNFRSF6B* were identified by RNAInter (http://www.rnainter.org). Targets of RNA-binding proteins (RBPs) were identified with ENCORI (http://www.rnasysu.com/encori/). A custom prediction for miRNA binding sites was performed in the miRDB database (http://www.mirdb.org). Target genes of miRNAs were obtained from miRTarBase (https://mirtarbase.cuhk.edu.cn/), miRDB, and TargetScanHuman 8.0 (http://www.targetscan.org/vert_80/). The targets obtained from TargetScanHuman were further shortlisted with the filtration criteria of a cumulative weighted context score of less than or equal to −0.4.

### 2.13. Correlation Analysis and Gene Ontology (GO)

The data for the expression of miRNAs (in RPMM) in different compartments of the brain was obtained from miRNATissueAtlas2 (http://www.ccb.uni-saarland.de/tissueatlas2), while the data for targeted genes was attained from the Genotype-Tissue Expression (GTEx) database (in TPM) (http://www.gtexportal.org/home/). The Pearson correlation analysis for the expression of miRNA with their respective target genes in the brain was performed in RStudio 4.2.2 (RStudio Team, 2022).

The GO of the genes based on biological and molecular function was performed with Enrichr (https://maayanlab.cloud/Enrichr/). Further, the enriched genes were clustered and visualized using the simplifyEnrichmen R package in RStudio 4.2.2. Heatmaps were generated in SRplot (http://www.bioinformatics.com.cn/srplot).

## 3. Results

### 3.1. Clinical Phenotype

**Figure 1:**
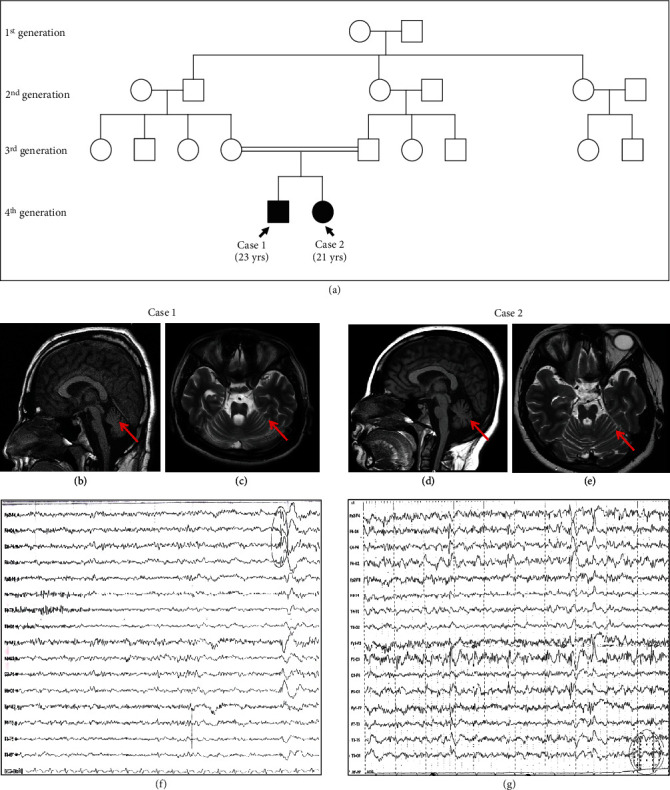
Clinical findings in a familial case of progressive myoclonus epilepsy. (a) Pedigree tree indicates sibling cases born to normal parent with second-degree consanguinity marriage. Brain MRI of (b, c) Case 1 and (d, e) Case 2. (b, d) Sagittal T1-weighted brain MRI and (c, e) axial FLAIR-weighted MRI indicated marked atrophy of cerebellum as well as caudate nuclei and putamen. (f, g) EEG showed focal epileptiform abnormalities over both the central and temporal regions.

A 23-year-old male born to second-degree consanguineous parents reported to the Clinical Department of Neurology, Kasturba Hospital, Manipal, as a follow-up for the seizure disorder (Figure 1(a)). The patient was diagnosed with progressive multifocal myoclonus associated with seizure since the age of 1 and was on antiepileptic drug medication (sodium valproate followed by additional clonazepam), however with incomplete seizure control. The early development of the patient was normal but gradually developed a myoclonic jerk, which is persistent until the present time. Systemic examination at the age of 21 reported disc pallor and ataxic features such as slow saccades, the presence of nystagmus, limb ataxia, and gait ataxia. Although cognition was mildly impaired, there were no indications of an abnormal pyramidal or extrapyramidal tract. Peripheral neuropathy was also absent (Table 1). The electroencephalogram (EEG) showed a mild diffuse disturbance of electric function (Figure 1(f)). Brain magnetic resonance imaging (MRI) reported diffuse brain stem atrophy with cruciate T2 wand flair hyperintensity in the pons (hot cross bun sign) and mild cerebellar and cerebral atrophy (Figures 1(b) and 1(c)). Genetic evaluation of the trinucleotide repeat region (SCA1, 2, 3, 6, and 7) showed the normal range of CAG repeats (Figure S1). The blood test showed no signs of cytopenia (Table 1).

A second sibling, a 21-year-old girl sister, was also diagnosed with PME and had seizure onset since the age of 1 (Figure 1(a)). Similar to her brother, her early development was normal but gradually developed myoclonic jerks which were persistent and left her bound to a wheelchair. The myoclonic jerk is exaggerated compared to her sibling (Case 1). The subject had frequent generalized tonic–clonic seizures with uprolling of the eyes. Since the age of 15, deterioration in speech has been observed, with occasional stammering. Perceptually, both ataxic and hyperkinetic features were observed with mildly impaired cognition, disc pallor, bilateral plantar extensor responses, and ataxic features such as slow saccades, the presence of nystagmus, limb ataxia, and gait ataxia. Peripheral neuropathy and abnormalities in the pyramidal and extrapyramidal tracts were absent (Table 1). EEG showed focal epileptiform abnormalities over both the central and temporal regions (generalized, anterior dominant epileptiform abnormalities with myoclonus), while somatosensory evoked potentials (SSEPs) did not show giant potentials (Figure 1(g)). MRI findings reported diffuse brain stem atrophy with cruciate T2 wand flair hyperintensity in the pons (hot cross bun sign) and mild cerebellar and cerebral atrophy (Figures 1(d) and 1(e)). Genetic evaluation for the trinucleotide repeat region (SCA1, 2, 3, 6, and 7) also showed the normal range of the CAG repeats. The blood test did not show any signs of cytopenia (Table 1).

### 3.2. Genetic Studies

The clinical features of the affected subjects suggested a case of PME, most likely ULD with defects in the associated gene, *CSTB*, or KCTD7-related PME (EPM3). Hence, extensive molecular analysis was performed to confirm the genetic defects. The amplification of the 193 bp region of *CSTB* gene revealed a single amplicon of 181 and 193 bp in two affected siblings, respectively, while two amplicons of 181 and 193 bp were observed in the parents. In accordance with the amplicon sizes, DNA sequencing showed homozygous genotypes with alleles of two dodecamer repeats and homozygous genotypes but with three dodecamer repeat alleles in respective siblings. Both parents were heterozygous for one allele with two repeats and another with three repeats (Figure S2). A homozygous missense mutation 2 nt downstream of the repeat region was also observed in affected individuals as well as in their parents (Figure S2).

Mitochondrial genome sequencing of parents and siblings revealed the H13a2a haplogroup; however, no mutation was observed in the previously reported PME-associated mitochondrial genes (*MT-ND5*, *MT-TL1*, *MT-TK*, *MT-TH*, *MT-TF*, *MT-TS2*, and *MT-TS1*). Further, there were no de novo variant(s) specific to affected individuals (Supporting Information 5).

The WES of both the affected siblings and their parents did not reveal mutations in any of the previously known PME genes, including the *KCTD7* gene. During the CNV analysis, gain was considered when the copy number was greater than 3, while the absence of both alleles was considered as loss. This was done to prevent false positives that may arise because of amplification variations during library preparation. Furthermore, the CNVs classified as likely pathogenic or pathogenic by ClassifyCNV were further assessed. No significant CNVs were identified among the probands (Supporting Information 6).

**Figure 2:**
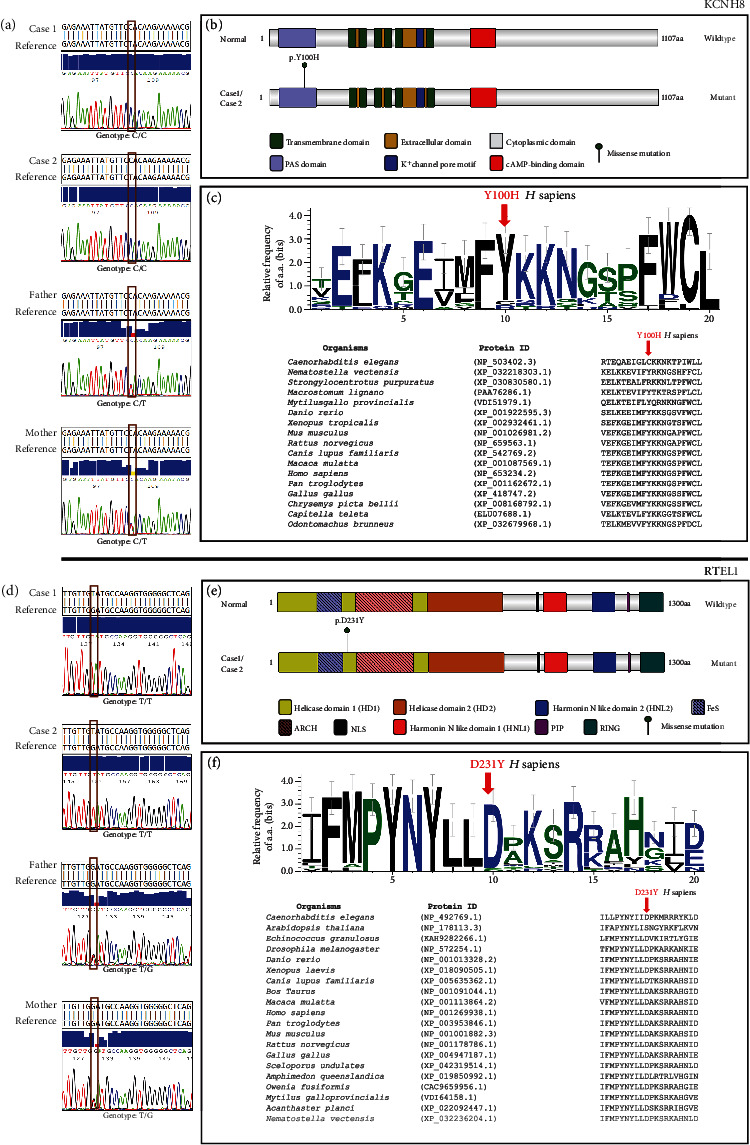
Molecular finding in familial case of progressive myoclonus epilepsy. (a, d) Whole exome sequencing followed by validation by Sanger sequencing confirmed homozygous [NM_144633.3:c.298T>C, p.(Tyr100His), rs565744148] in *KCNH8* gene and [NM_001283009.2:c.691G>T, p.(Asp231Tyr)] in *RTEL1* gene common to both the cases, while heterozygous alleles were observed in parents. (b) The mutation in KCNH8 residue lies in the PAS domain while (e) mutation in RTEL1 lies in the helicase domain of the protein. Logo plot showing conservation of (c) KCNH8 or (f) RTEL1 residues (mutated in cases) among the eukaryotes.

Family-based segregation analysis of WES data, followed by variant prioritization, shortlisted 19 variants in Case 1 and 16 variants in Case 2. Further, scrutinization of shortlisted variants for common variants in both affected probands followed by computational assessment for pathogenicity and validation by Sanger sequencing revealed homozygous missense mutations in Exon 2 of *KCNH8* [NM_144633.3:c.298T>C, p.(Tyr100His), rs565744148], Exon 8 of *RTEL1* [NM_001283009.2:c.691G>T, p.(Asp231Tyr)], and Exon 17 of *RAF1* [NM_002880.4:c.1922C>T, p.(Thr641Met), rs587777587] genes (Table 2 and Supporting Information 7). These variants were predicted as deleterious during in silico analysis, and parents were heterozygous for the prioritized variant (Figures 2(a) and 2(d)). The mutation identified in *RAF1* is a known natural variant but has been reported to be associated with dilated cardiomyopathy [16]. Apart from dilated cardiomyopathy characterized by decreased left ventricular ejection fraction, mitral regurgitation, and ventricular arrhythmia (OMIM:615916), RAF1 is also associated with autosomal dominant hypertrophic cardiomyopathy such as LEOPARD Syndrome 2 and Noonan Syndrome 5. LEOPARD Syndrome 2 is additionally characterized by lentigines, retardation of growth, EKG abnormalities, pulmonic stenosis, ocular hypertelorism, delayed puberty, and deafness (OMIM:611554), whereas Noonan Syndrome 5 is manifested with developmental delay, pectus anomaly, characteristic facies, short stature, and more (OMIM:611553) [4]. Our cases do not present any sign of cardiomyopathy; hence, we rule out *RAF1* as a likely cause of PME.

The c.298T>C mutation of *KCNH8* is a rare allele observed among only South Asian populations with an allele frequency of 0.0023 and has not been previously associated with any disease phenotype (http://www.ncbi.nlm.nih.gov/snp/rs565744148). This mutation altered the conserved neutral tyrosine residue of the Per-Arnt-Sim (PAS) domain of KCNH8 into a basic histidine residue (Figures 2(b) and 2(c)). Although the variant is predicted to be pathogenic by various pathogenicity prediction tools, it is interpreted as a variant of unknown significance (PP3 + PP1) in accordance with the American College of Medical Genetics and Genomics (ACMG) guidelines [17].

**Figure 3:**
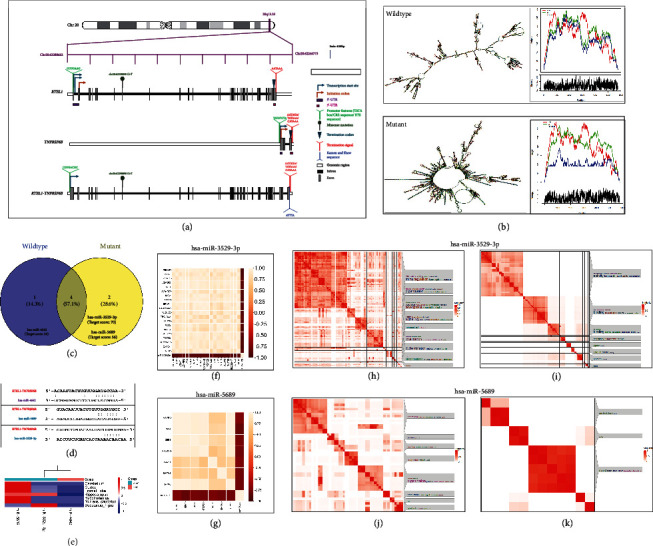
Computational analysis. (a) Genomic location of *RTEL1*, *TNFRSF6B*, and *RTEL1-TNFRSF6B*. (b) Secondary RNA structure prediction for wild-type and mutant *RTEL1-TNFRSF6B* lncRNA using RNAfold. (c) Venn diagram representing predicted loss and gain of two new miRNA sites after mutation of *RTEL1-TNFRSF6B*. (d) Alignment of selected miRNAs with *RTEL1-TNFRSF6B*. (e) Heatmap showing the expression of selected miRNAs (obtained from the miRNATissueAtlas2 database) in different compartments of the brain. (f, g) Heatmap showing the top 20 inversely correlated genes with miR3529-3p and miR5689 in the brain. Heatmap showing a further cluster of significantly enriched genes (*p* value of < 0.05) based on Gene Ontology of (h, j) biological processes and (i, k) molecular functions.

We also discovered a novel missense mutation in *RTEL1* [p.(Asp231Tyr)], which substituted the conserved hydrophilic, acidic aspartate residue with a neutral and hydrophobic tyrosine in the Helicase Domain 1 of RTEL1 (Figures 2(e) and 2(f)). Various in silico prediction tools predicted the variant as pathogenic; however, in accordance with the ACMG guidelines [17], the variant is of uncertain significance (PM2 + PP3 + PP1). *RTEL1* has multiple transcripts and isoforms that show tissue-dependent expression. In this manuscript, the mutation nomenclature for *RTEL1* refers to the 1300 aa isoform (NM_001283009.2), which is predominantly expressed in the human brain [18, 19]. *RTEL1* is involved in the maintenance of telomere integrity; hence, we assessed the telomere length in both the affected probands and parents; however, significant difference in telomere length with respect to their age- and sex-matched control was not detected (Figure S3). The variant identified in the *RTEL1* gene is also located within *RTEL1-TNFRSF6B* [chr20:62298898_G>T; NR_037882.1, hg19] (Figure 3(a)) which belongs to lncRNA [20] and is also indicated as circular RNA (circRNA) in various databases such as ENCORI, RNAInter, and circAtlas. This novel variant of *RTEl1* and *RTEL1-TNFRSF6B* identified in our cases is predicted to cause a donor loss by SpliceAI (Δscore = 0.4); however, the score is less than the recommended cut-off. In addition, Spliceator tool did not show this variant to cause a loss or gain in acceptor and donor sites. Hence, we did not further explore the alteration in the splice site.

Several additional variants, segregated by family but unique to each case were also identified. Additionally, homozygous mutations in *CACNA1F*, *GREB1L*, *KLHL13*, *DHX16*, *DSC3*, *MYCBP2*, and *SLAIN1* and compound heterozygous variants in *DCHS2*, *MYO15A*, *TG*, and *TTN* were segregated from parents but present only in Case 1, whereas in Case 2, homozygous variants in *SLC22A17*, *CIDEB*, *ACTL9*, *ZNF846*, and *PLPPR2* and a compound heterozygous variant in *ABCC1*, *CSMD3*, *SAG*, and *TTN* were segregated from parents and unique to Case 2. These additional variants might be responsible for the observed difference in severity among the siblings.

### 3.3. Computational Analysis

With the aim of delineating the impact of the identified variant (chr20:62298898_G>T) on the *RTEL1-TNFRSF6B* lncRNA, we predicted the centroid secondary structure of the *RTEL1-TNFRSF6B* RNA with and without mutation. A significant change in the conformation of the RNA structure was observed upon mutation (Figure 3(b)). Further, a change in minimum free energy (MFE) from −2050.68 kcal/mol in wild-type to −1505.42 kcal/mol in mutant *RTEL1-TNFRSF6B* RNA was observed. Additional analysis for the identification of interacting partners of *RTEL1-TNFRSF6B* suggested interactions of *RTEL1-TNFSF6B* with various RBPs (DKC1, DHX9, AIFM1, DICER1, ADAR, ACIN1, and DDX54) and miRNAs (hsa-miR-6806-3p, hsa-miR-1976, hsa-miR-5008-5p, hsa-miR-6729-5p, hsa-miR-632, hsa-miR-3652, hsa-mir-615, hsa-mir-615-3p, hsa-miR-181a-5p, and hsa-miR-132-3p) (Supporting Information 8).

Custom prediction for miRNA binding site because of mutation to *RTEL1-TNFRSF6B* predicted loss of binding site for hsa-miR-4642 (target score: 54), while gain of binding site was predicted for hsa-miR-3529-3p (target score: 70) and hsa-miR-5689 (target score: 56) (Figures 3(c) and 3(d)). Further exploration of the database revealed the expression of these miRNAs in various compartments of the brain (Figure 3(e)). We listed all the experimentally validated and strongly predicted target genes for the miRNAs (has-miR-5689 and has-miR-3529-3) whose binding sites were gained upon mutation. Further correlation analysis with the expression data of miRNA with their respective target gene expression data in various compartments of the brain revealed 720 target genes with an inverse but significant correlation (*r* ≤ −0.7) with hsa-mir-3529-3p and 7 target genes with a significant inverse correlation with hsa-mir-5689 (Figures 3(f) and 3(g)).

Additionally, functional enrichment of these significantly correlated genes based on biological process and molecular function (GO) followed by clustering of enriched GO terms (*p* value < 0.05) revealed involvement of target genes of hsa-mir-3529-3p in regulation of RNA and transcription, maintenance of chromatin and telomeres, chromatin organization and remodeling, development, signaling pathways, response to stimulus homeostasis, transport, calcium channel activity, cell cycle, metabolic pathways, ubiquitination, and more (Figures 3(h) and 3(i)). Target genes of hsa-mir-5689 are involved in secretion, transport, calcium channel activity, metabolic process, signaling, regulation, response to stimulus, organization of cell and organelle, immune response, nucleotidase activity, and more (Figures 3(j) and 3(k)).

### 3.4. Molecular Analysis

**Figure 4:**
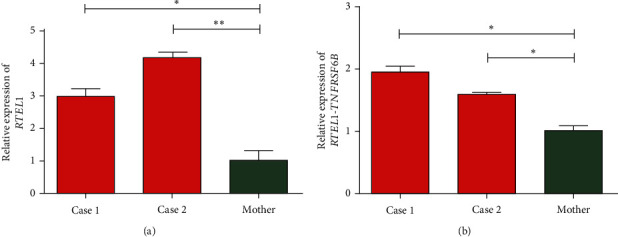
Gene expression analysis. Relative gene expression of (a) *RTEL1* and (b) *RTEL1-TNFRSF6B* in affected siblings and normal mother. One-way ANOVA was employed for the statistical analyses. ^∗^*p* ≤ 0.05.

The quantification of the expression of *RTEL1* and *RTEL1-TNFRSF6B* in affected probands with homozygous likely pathogenic novel variant relative to heterozygous normal mother revealed approximately three- and fourfold increased expression of *RTEL1* in Case 1 and Case 2, respectively (Figure 4(a)). The expression of *RTEL1-TNFRSF6B* in Case 1 and Case 2 was, respectively, 1.9- and 1.6-fold higher than in the mother (Figure 4(b)).

## 4. Discussion

PMEs are genetically heterogeneous disorders with overlapping phenotypes among them. In our studies, two affected siblings with asymptomatic parents presented cardinal features of PMEs. The clinical phenotype suggested that the siblings had ULD or KCTD7-related PME (EPM3). ULD results from the abnormal expansion of a dodecamer repeats located upstream (70 nucleotides) of the transcription start site (TSS) of the *CSTB* gene. While the normal allele contains 2–3 copies of this minisatellite repeat, alleles with 12–17 repeats or more than 60 repeats show genomic instability and a reduced level of *CSTB* transcripts [21]. In search of repeat expansion in the *CSTB* gene, our analysis of both the affected siblings showed homozygosity in the dodecamer repeat region, with the normal *CSTB* allele containing two and three repeats, respectively, while both the parents were heterozygous, presenting one allele with two repeats and another with three repeats. Additional homozygous missense mutations were identified two nucleotides downstream of the repeat site in the affected subjects as well as in their parents, thus suggesting the benign effect of missense mutations. Further, WES data indicated no mutations in the coding regions of *CSTB*, and hence, we ruled out *CSTB* as the cause of the disease phenotype.

Many subtypes of PME, such as myoclonic epilepsy with ragged-red fibers (MERRF) syndrome, are due to mutations in mitochondrial genome [2]. However, we neither found mutations in previously reported PME associated mitochondrial genes nor any de novo mitochondrial variants in our affected subjects. The WES of both siblings and parents did not reveal any mutations in previously associated PME genes, and the CNV analysis did not reveal significant alterations.

Further analysis of the exome data, followed by validation with Sanger sequencing, identified novel genes that could be associated with PMEs. Mutations in the potassium channel and their association with epilepsy have been known for decades [22]. In addition, defects in various potassium channels (*KCNC1*, *KCTD7*) have been associated with PMEs such as myoclonus epilepsy and ataxia due to potassium channel mutations (MEAK) and KCTD7-related PME (EPM3) [1, 23]. We did not find any heterozygous or homozygous mutations in these genes. Remarkably, we discovered a common homozygous mutation [c.298T>C, p.(Tyr100His)] in the PAS domain of the *KCNH8* gene in both siblings. This variant (rs565744148) is reported as homozygous in the control population of the Genome Aggregation Database (gnomAD) as well, but the control group of the gnomAD database includes genomic data from individuals of unknown history of severe pediatric disease or was not enrolled as part of a disease-specific study [24]. The meta-analysis conducted by Dhiman et al. reported the prevalence of epilepsy in 4.3 per 1000 people of India alone [25]. Additionally, epilepsy can develop at any age [26]. It is possible that some individuals in the control group have an undiagnosed condition or might develop a disease later in life. Hence, the possibility of including an individual who might have developed epilepsy in the control group of the gnomAD database cannot be ruled out. Additionally, there are few instances where pathogenic variants associated with autosomal recessive diseases are presented in a homozygous state in the control population of gnomAD. For example, the rs334 variant of the *HBB* gene (NM_000518.5:c.20A>T; p.Glu7Val) is reported as a known pathogenic variant in HbS disease, and the rs80338939 variant of the *GJB2* gene (NM_004004.6:c.35del; p.Gly12fs) is reported as pathogenic in autosomal recessive nonsyndromic hearing loss 1A disease. Both of these are represented as rare alleles in the gnomAD population. Among the individuals who have these *HBB* and *GJB2* variants in homozygous state, few belong to the control population [6, 17, 24]. Constraint metrics such as the loss-of-function intolerant (pLI) and loss-of-function observed/expected upper bound fraction (LOEUF) score aid in the identification of genes likely involved in a dominant or recessive disorder. KCNH8 is a gene with a pLI score of 0 and LOEUF of 1.55, suggesting the likelihood of involvement of *KCNH8* in recessive disorders such as those observed in our cases [27, 28]. Further, in silico pathogenicity prediction suggests the deleterious effect of a variant with a rare allele frequency of 0.0002917, and the literature review as described below suggests KCNH8 involvement in the hyperexcitability as observed among our cases.

Potassium voltage–gated channel subfamily H member 8 (KCNH8) is a member of ELK (EAG-like K^+^) of the ether-a`-go-go family (KCNH) channels that have been implicated in neuronal excitability, repolarization of cardiac action potential, cell differentiation, and tumor proliferation [29]. KCNH8, along with KCNH3 and KCHN4, is primarily expressed in the human nervous system, predominantly in the substantia nigra, thalamus, cerebellum, and pons [30]. Human KCNH8 contains a cytoplasmic N-terminus containing the PAS domain capped by a short sequence containing an amphipathic helix (Cap domain), followed by six transmembrane segments (S1–S6) with the conserved Kv channel motif, the K^+^ channel pore, and C-terminal cytoplasmic cyclic nucleotide binding homology (CNBH) domain [29, 30]. Similar to other KCNH channels, Elk channels are tetrameric complexes [30–33]. The first four transmembrane segments of each subunit of the tetrameric complex form the voltage sensor domain (VSD), while the pore domain is formed by the S5 and S6 segments of all four subunits together [31, 32].

The PAS domain of KCNH channels is a structurally conserved domain [34] and contains a hydrophobic patch that mediates its interaction with other regions of the channel [31–33, 35] to regulate the function of the channel. The homozygous pathogenic mutation, p.(Tyr100His) on the PAS domain of KCNH8 protein identified in our probands, lies around the hydrophobic patch; thus, the interaction of the PAS domain with other regions of the channel might be affected. Although mutations in the Elk subfamily have not been previously associated with PMEs or epilepsy, genetic deletion and drug inhibition of KCNH3 in a mouse model demonstrated hippocampal hyperexcitability and epilepsy with brief myoclonic activity [36]. The function of KCNH8 is not yet well understood, but consistent with the characteristics of other ELK channels, KCNH8 channels also exhibit voltage-dependent potentiation (VDP) and activate at hyperpolarized potential ranges (significantly at neuronal resting potentials). Further, it generates slow-activating voltage-dependent K^+^ currents and exhibits little inactivation near resting potential, thereby contributing to subthreshold activity in neurons [30, 35]. Considering the important role of KCNH8 in neurons, and the evidence demonstrating the pathogenic effect of mutation in PAS residues of the KCNH family, it is more likely that the mutation in *KCNH8* identified in the probands is the underlying cause of the observed epileptic phenotype.

Hyperexcitability is known to generate a condition for oxidative stress and inflammation, the former being one of the factors responsible for neuronal death via DNA damage [37, 38]. Mutations in various DNA repair genes are associated with neurodegenerative disorders [39]. A novel homozygous mutation predicted to be deleterious was identified in an essential helicase gene, *RTEL1*, which plays a critical role in maintaining genome stability via resolution of secondary structures that appears in the process of replication, recombination, and repair [40–43]. RTEL1 is a multidomain protein with a helicase domain at the N-terminus, followed by central two tandem harmonin homology domains HHD1 (886-978aa) and HHD2 (1056-1140aa) separated by a long-disordered linker region of about 75 residues, and a C-terminus with a C4C4-type RING domain [41–44]. The telomere T/D-loop is disassembled, and unwinding of G-quadruplex is mediated by the helicase domain of RTEL1 [40]. Recent studies have demonstrated the association of RTEL1 with POLDIP3 via helicase domain for the resolution of R-loops formed during replication stress at various susceptible genomic regions [43–45]. The c.691G>T mutation in *RTEL1* identified in our patients substituted conserved negatively charged aspartate for neutral tyrosine [p.(Asp231Tyr)] residues of the helicase domain.

Mutations in various domains of *RTEL1*, including the helicase domain, have been associated with telomere biology disorders such as dyskeratosis congenita, Hoyeraal–Hreidarsson syndrome, pulmonary fibrosis, bone marrow failure syndrome, myeloid neoplasms, and increased susceptibility to brain tumors [41, 46, 47]. Phenotypically severe DC and HH patients with the *RTEL1* mutation exhibit short and heterogeneous telomeres, fragility, and fusion [41, 48]. Telomere length estimation in our affected siblings and their parents did not reveal a significant difference in comparison to age- and sex-matched controls, and there were no symptoms of telomere biology–related disorders. However, our expression analysis for *RTEL1* revealed a comparative increase in the expression of *RTEL1* transcript in affected probands. Previously, the overexpression of *RTEL1* in SV40-transformed fibroblast has been shown to induce toxicity and cell death [49]. Whether the overexpression of *RTEL1* is responsible for neurodegeneration as indicated by MRI requires a detailed study.

The genomic location of the *RTEL1* gene overlaps with the genomic region of noncoding RNA, *RTEL1-TNFRSF6B*. *RTEL1-TNFRSF6B* is a naturally occurring read-through transcript transcribed between the neighboring *RTEL1* and *TNFRSF6B* genes on Chromosome 20 [20]. Although the read-through transcript is a candidate for nonsense mRNA decay (NMD) and unlikely to translate into protein, this transcript belongs to a class of long noncoding RNAs (lncRNAs) known to regulate the expression of genes [50]. This lncRNA is also indicated as circRNA in various databases such as ENCORI, RNAInter, and circAtlas. The centroid secondary RNA structure prediction for wild-type and mutant *RTEL1-TNFRSF6B* showed an increase in MFE and circular conformation upon mutation, whereas other missense variants of *RTEL1-TNFRSF6B*, which are reported as pathogenic or likely pathogenic in the ClinVar database and which are associated with telomere biology disorders, including dyskeratosis congenita, did not reveal a significant change in the conformation of the *RTEL1-TNFRSF6B* nor in MFE compared to the wild type (Supporting Information 9). However, these variants are associated with functional disruption of RTEL1. It is likely that the novel variant of *RTEL1-TNFRSF6B* (chr20:62298898_G>T; NR_037882.1, hg19) identified in our cases would be the one causing the PME, among all the other variants. An increase in MFE suggests instability of RNA; however, the expression analysis in probands and mother revealed a slight increase in *RTEL1-TNFRSF6B* expression in probands. The circular conformation of RNA protects it from degradation by nucleases and hence increases the stability of RNA. In various studies, the increase in MFE of circular form of RNA compared to its linear form can also be noted [51, 52]. Thus, the increase in *RTEL1-TNFRSF6B* expression might be due to conformational change. The role of lncRNAs in epilepsy has recently gained focus. The genes associated with epilepsy can be modulated by lncRNAs via their interaction with miRNAs [53]. Our computational analysis predicted the gain of binding sites for two miRNAs (hsa-miR-3529-3p and hsa-miR-5689) to *RTEL1-TNFRSF6B* upon mutation. Further correlation analysis of miRNAs with their candidate target genes using brain expression data from databases reported various significantly and inversely correlated target genes. Many of these genes are involved in biological and molecular processes known to be impaired in PMEs, such as neuronal development, metabolic processes, transport, channel activity, DNA metabolism, and chromatin maintenance.

Interestingly, some of the correlated genes of hsa-mir-3569-3p such as *RTEL1*, *TTN*, *MYO15A*, and *SLC22A17* was observed to be mutated in our cases. *RTEL1*, as discussed earlier, is mutated and overexpressed in both of our affected subjects. It is likely that the overexpression of *RTEL1-TNFRSF6B*, as observed in our probands, might have resulted in the overexpression of *RTEL1* via its interaction with hsa-miR-3569-3p, but this requires further validation. In our second case with a severe phenotype, an additional homozygous variant was observed in solute carrier, *SLC22A17*, which is also one of the significant target genes of hsa-miR-3569-3p. Various solute carriers and transporters are associated with epilepsy [54], and the severity in Case 2 can be explained by an additional pathogenic variant in *SLC22A17*, a target gene that is likely to be modulated by *RTEL1-TNFRSF6B* overexpression via hsa-miR-3569-3p.

The interactome analysis for *RTEL1-TNFRSF6B* also revealed interactions of the lncRNA with various RBPs and miRNAs, of which in the RBP, AIFM1 is associated with X-linked childhood cerebellar ataxia with severe sustained myoclonus [55]. Additionally, hsa-miR-181a-5p and hsa-miR-132-3p that are predicted to interact with *RTEL1-TNFRSF6B* have been previously reported to be upregulated in epileptic conditions in various animal models as well as patients [56]. These miRNAs are known to target various epilepsy-associated genes (Supporting Information 10). In addition, *RTEL1-TNFRSF6B* and *RTEL1* are located in the chromosomal region previously associated with epilepsy [57]. Thus, taking all these factors into consideration, *RTEL1-TNFRSF6B* could be considered one of the candidate genes that might be directly or indirectly associated with PME.

## 5. Conclusion

In summary, we have identified an idiopathic family of two siblings with the PME phenotype born to a healthy couple of second-degree consanguineous marriage. Differential diagnosis based on the phenotypes and the molecular analysis excludes the known genetic basis for the phenotype. This is the first report of the association of *KCNH8*, *RTEL1*, and *RTEL1-TNFRSF6B* in PMEs. The pLI score ≤ 0.86, homozygosity of the variant, segregation from parents, presence in both affected probands, deleterious pathogenicity as predicted by various computational tools, and literature review support the involvement of *KCNH8*, *RTEL1*, and *RTEL1-TNFRSF6B* with the recessive disease phenotype of our cases. Although mutations in various other potassium channels have been previously associated with PMEs, we report for the first time a mutation in *KCNH8* in PME cases that could be a reason for hyperexcitability. Further, we also report an additional novel homozygous mutation in the DNA repair gene, *RTEL1*, in both the siblings. Mutations in *RTEL1* have previously been associated with telomere biology disorders but not epilepsy or PMEs. The affected individuals in our study do not present any symptoms of telomere-associated disorders; however, considering the importance of *RTEL1* in maintaining the genome integrity and the fact that hyperexcitation due to seizure could result in oxidative stress followed by genome instability, we speculate that mutations in *RTEL1* along with its overexpression can impact the phenotype of PME. Interestingly, the novel variant identified in *RTEL1* is shared by the lncRNA, *RTEL1-TNFRSF6B*, and in silico analysis revealed a significant change in the conformation of the RNA. Also, *RTEL1-TNFRSF6B* was overexpressed in siblings. As various RBPs and miRNAs that regulate the expression of seizure-associated genes have been identified to interact with *RTEL1-TNFRSF6B*, this lncRNA might be associated with the PME phenotype. However, further functional studies are required.

## Figures and Tables

**Table 1:** Clinical findings for the familial sibling cases of idiopathic progressive myoclonus epilepsy with ataxia.

	**Case 1**	**Case 2**
Chief complaint	Seizure-GTCS/myoclonus	Seizure-GTCS/myoclonus
Family history	Younger sister	Elder brother
Early development	Normal	Normal
First neurological symptoms	Myoclonus	Myoclonus
Age of onset (year)	1	1
Progression	Myoclonus + GTCS + ataxia	Myoclonus + GTCS + ataxia
Examination	++	++
Dysmetria	++	++
Finger to nose test	++	++
Dysdiadochokinesia	++	++
Ataxic gait	++	++
Romberg test	Absent	Absent
Deep tendon reflex	2+	2+
Ankle clonus	Absent	Absent
Babinski sign	Absent	Absent
MRI	Hot cross bun sign/cerebral and cerebellar mild atrophy	Hot cross bun sign/cerebral and cerebellar mild atrophy
EGG	Dys I generalized	Dys III generalized
Attention and autistic feature	Absent	Absent
Frequency of ataxia	Moderate	Moderate
Epileptic seizure type	Myoclonus + GTCS	Myoclonus + GTCS
Action myoclonus	Present	Present
Progressive ataxia	Present	Present
Cognition regression	Present (frontal predominant)	Present (frontal predominant)
Optic atrophy	Present	Present
Retinitis	Absent	Absent
Hemoglobin	16.5 g/dL	11.4 g/dL
Neutrophils	40%	42.6%
Platelets	313 × 10^3^/*μ*L	458 × 10^3^/*μ*L

*Note:* ++: moderately present.

Abbreviation: GTCS: generalized tonic–clonic seizure.

**Table 2:** Details of variants in shortlisted candidate genes and output of in silico analysis for their pathogenicity.

**Gene symbol**	***KCNH8***	***RTEL1***
Gene name	Potassium voltage–gated channel subfamily H member 8	Regulator of telomere elongation helicase 1
Variant locus	chr3:19295367	chr20:62298898
Identified in	Case 1, Case 2	Case 1, Case 2
Variant type	SNV	SNV
Variant effect	Missense mutation	Missense mutation
Variant details	NM_144633.3:c.298T>C; (p.Tyr100His)	NM_032957.5:c.691G>T; (p.Asp231Tyr)
Variant location on gene	Exonic	Exonic
Exon affected	2	8
dbSNP ID	rs565744148	Novel
Minor allele frequency (MAF)	0.001	—
Zygosity in cases	Homozygous	Homozygous
Genotype of cases	C/C	T/T
Genotype of parent	T/C	G/T
In silico pathogenicity prediction		
SIFT_pred	Damaging	Damaging
Polyphen2	Damaging	Damaging
LRT_pred	Unknown	Deleterious
MutationTaster_pred	Disease causing	Disease causing
MutationAssessor_pred	Medium	High
PROVEAN_pred	Deleterious	Deleterious
VEST3_prediction	Damaging	Damaging
FATHMM_pred	Damaging	Tolerated
MetaSVM_pred	Damaging	Damaging
MetaLR_pred	Damaging	Damaging
M-CAP_pred	Deleterious	Deleterious
CADD_phred prediction	Damaging	Damaging
DANN_score	Deleterious	Deleterious
fathmm-MKL_coding_pred	Damaging	Damaging
GERP++_RS	Constrained	Constrained
Grantham	Moderately conservative	Radical
PhyloP	7.97	8.6
phyloP100way_vertebrate	7.941	8.694
phyloP20way_mammalian	0.964	0.953
phastCons100way_vertebrate	1	1
SiPhy_29way_logOdds	15.336	16.296
SiPhy_29way_logOdds_rankscore	0.739	0.825
VarAFT prediction	Pathogenic	Pathogenic
ACMG classification	Uncertain significance (PP3 + PP1)	Uncertain significance (PM2 + PP3 + PP1)

## Data Availability

The datasets generated during and/or analyzed during the current study are available from the corresponding author on reasonable request. The variants identified in the study have been submitted to the ClinVar database (https://www.ncbi.nlm.nih.gov/clinvar/). The accession numbers SCV002559858 and SCV002559860 correspond to variant [NM_144633.3:c.298T>C, p.(Tyr100His), rs565744148] of *KCNH8* and [NM_001283009.2:c.691G>T, p.(Asp231Tyr)] of *RTEL1*, respectively.

## Supporting Information

Supporting Information 1 Figure S1: Trinucleotide repeat expansion test result. Genetic evaluation for the trinucleotide repeat region (SCA1, 2, 3, 6, and 7) also showed the normal range of the CAG repeats.

Supporting Information 2 Figure S2: Genetic analysis of the dodecamer repeat region of CSTB. The amplification of region of the *CSTB* gene, which includes dodecamer repeat region, showed single amplicon of 181 and 193 bp in Case 1 and Case 2, respectively, while two amplicons of 181 and 193 bp were observed in both parents. Sanger sequencing of the amplicons revealed a homozygous genotype with an allele of two dodecamer repeats in Case 1, while Case 2 also had a homozygous genotype but with an allele of three dodecamer repeats. Both parents were heterozygous and showed one allele with two repeats and another with three repeats. A homozygous missense mutation 2 nt downstream to repeat site was also observed in cases as well as in parents.

Supporting Information 3 Figure S3: Relative telomere length in cases and parent: bar diagram showing the relative telomere length in both cases and parents. No significant difference in telomere length was observed in both the cases with respect to their age- and sex-matched control.

Supporting Information 4 Details of primers used for Sanger sequencing and expression analysis.

Supporting Information 5 Output of mitochondrial genome sequencing data analysis for both cases of progressive myoclonus epilepsy.

Supporting Information 6 Output of copy number variation analysis in both the probands and their parents.

Supporting Information 7 Family-based segregation analysis and information of shortlisted candidate genes and variants identified in whole exome sequencing of both sibling cases of PMEs.

Supporting Information 8 Information on various targets of *RTEL1-TNFRSF6B* RNA obtained from the RNAInter database.

Supporting Information 9 Centroid RNA secondary structure prediction using RNAfold for *RTEL1-TNFRSF6B* with missense pathogenic or likely pathogenic variants reported in ClinVar database.

Supporting Information 10 Computational analysis for hsa-miR3529-3p and hsa-miR-5689 and their respective target genes for identification of significantly and inversely correlated target genes, followed by enrichment of the shortlisted genes based on Gene Ontology.
