# Supplementary material for: Discovery of a Novel Shared Variant Among RTEL1 Gene and RTEL1-TNFRSF6B lncRNA at Chromosome 20q13.33 in Familial Progressive Myoclonus Epilepsy
Source: Int J Genomics. 2024 Aug 10;2024:7518528. doi: 10.1155/2024/7518528 (PMC11330336; doi:10.1155/2024/7518528)
Supplement: Supporting Information 1 — Figure S1: Trinucleotide repeat expansion test result. Genetic evaluation for the trinucleotide repeat region (SCA1, 2, 3, 6, and 7) also showed the normal range of the CAG repeats. [file 7518528.f1.pdf]

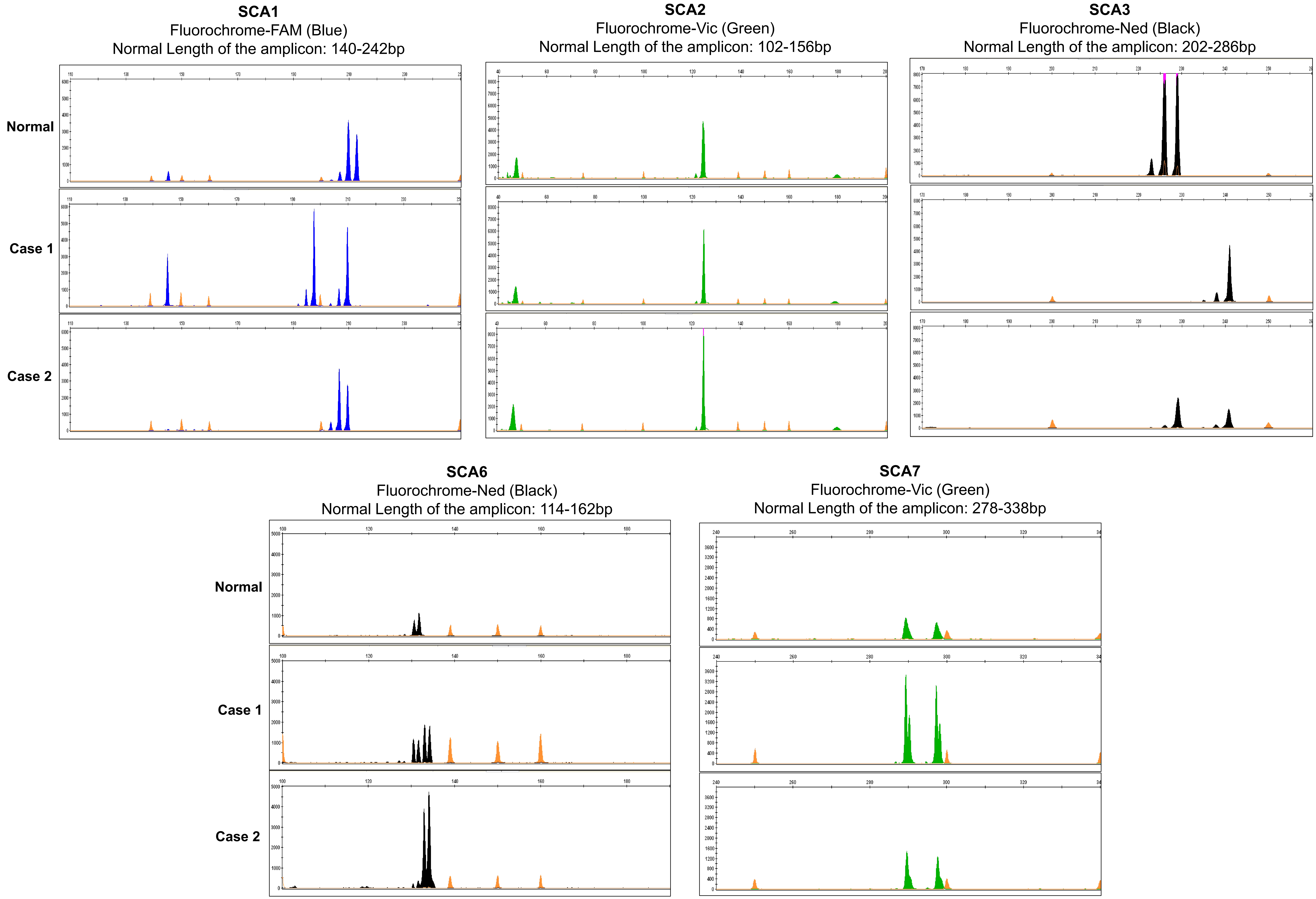
SCA1
Fluorochrome-FAM (Blue)
Normal Length of the amplicon: 140-242bp
SCA2
Fluorochrome-Vic (Green)
Normal Length of the amplicon: 102-156bp
SCA3
Fluorochrome-Ned (Black)
Normal Length of the amplicon: 202-286bp
Normal
Case 1
Case 2
SCA6
Fluorochrome-Ned (Black)
Normal Length of the amplicon: 114-162bp
SCA7
Fluorochrome-Vic (Green)
Normal Length of the amplicon: 278-338bp
Normal
Case 1
Case 2
