# Supplementary material for: Discovery of a Novel Shared Variant Among RTEL1 Gene and RTEL1-TNFRSF6B lncRNA at Chromosome 20q13.33 in Familial Progressive Myoclonus Epilepsy
Source: Int J Genomics. 2024 Aug 10;2024:7518528. doi: 10.1155/2024/7518528 (PMC11330336; doi:10.1155/2024/7518528)
Supplement: Supporting Information 2 — Figure S2: Genetic analysis of the dodecamer repeat region of CSTB. The amplification of region of the CSTB gene, which includes dodecamer repeat region, showed single amplicon of 181 and 193 bp in Case 1 and Case 2, respectively, while two amplicons of 181 and 193 bp were observed in both parents. Sanger sequencing of the amplicons revealed a homozygous genotype with an allele of two dodecamer repeats in Case 1, while Case 2 also had a homozygous genotype but with an allele of three dodecamer repeats. Both parents were heterozygous and showed one allele with two repeats and another with three repeats. A homozygous missense mutation 2 nt downstream to repeat site was also observed in cases as well as in parents. [file 7518528.f2.pdf]

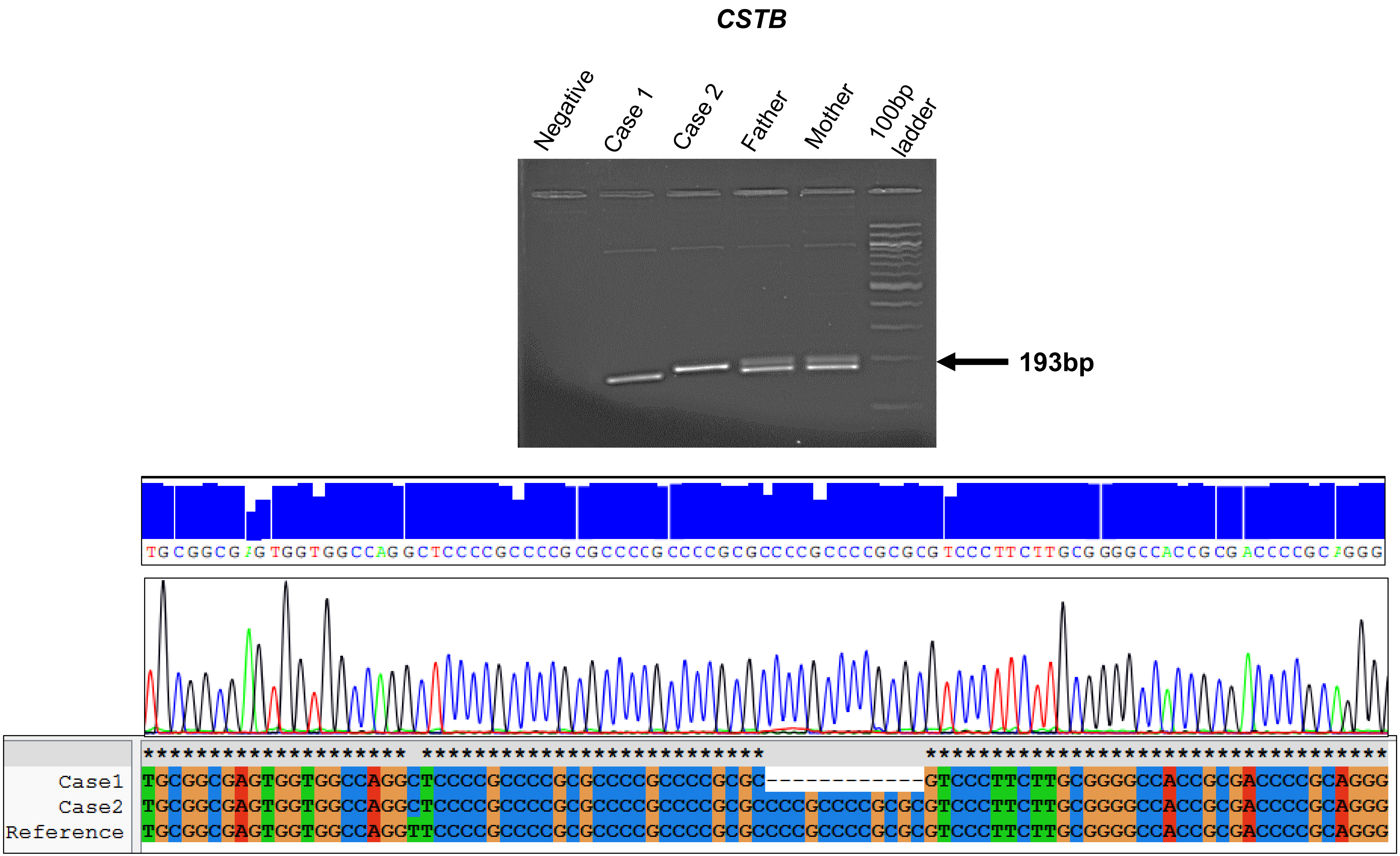

CSTB
Negative
Case 1
Case 2
Father
Mother
100bp ladder
193bp
Case1
Case2
Reference
