# Supplementary figures and images for: Discovery of a Novel Shared Variant Among RTEL1 Gene and RTEL1-TNFRSF6B lncRNA at Chromosome 20q13.33 in Familial Progressive Myoclonus Epilepsy
Source: Int J Genomics. 2024 Aug 10;2024:7518528. doi: 10.1155/2024/7518528 (PMC11330336; doi:10.1155/2024/7518528)

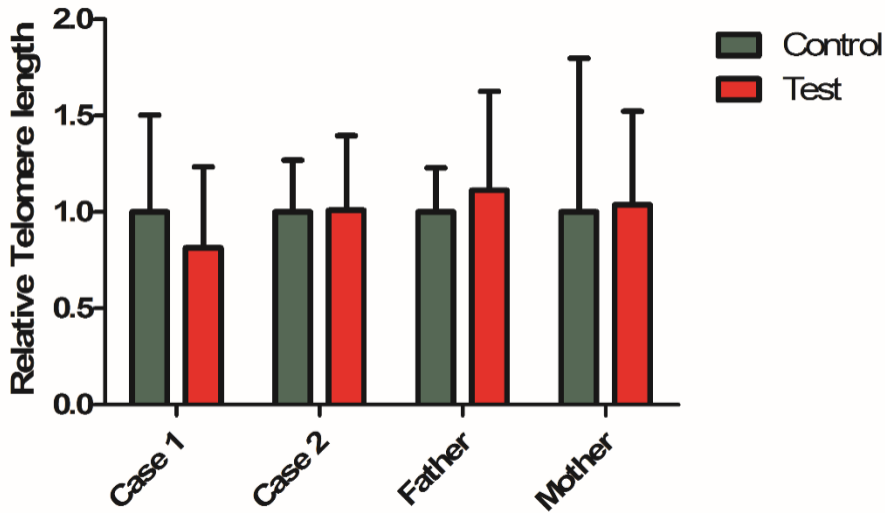

Supplement: Supporting Information 3 — Figure S3: Relative telomere length in cases and parent: bar diagram showing the relative telomere length in both cases and parents. No significant difference in telomere length was observed in both the cases with respect to their age- and sex-matched control. [file 7518528.f3.pdf]
