# Supplementary material for: Discovery of a Novel Shared Variant Among RTEL1 Gene and RTEL1-TNFRSF6B lncRNA at Chromosome 20q13.33 in Familial Progressive Myoclonus Epilepsy
Source: Int J Genomics. 2024 Aug 10;2024:7518528. doi: 10.1155/2024/7518528 (PMC11330336; doi:10.1155/2024/7518528)
Supplement: Supporting Information 4 — Details of primers used for Sanger sequencing and expression analysis. [file 7518528.f4.docx]

**Supplementary File 1-** Details of primers used for Sanger sequencing and expression analysis.

| Purpose | Primer | Primer sequence | Tm | Amplicon size | Amplicon region |
| --- | --- | --- | --- | --- | --- |
| Validation | CSTBF | CCCGGAAAGACGATACCAG | 64°C | 193bp | chr21:45196264-45196456 |
|  | CSTBR | GAGGAGGCACTTTGGCTTC |  |  |  |
|  | RTEL1F | TCCTTCCGGGTCAGAAGACA | 60°C | 461bp | chr20:62298736-62299196 |
|  | RTEL1R | AGCTGTCAGGATCAAGAGCG |  |  |  |
|  | KCNH8F | GCTGGATTTGCCCGAACTGA | 62°C | 339bp | chr3:19295226-19295564 |
|  | KCNH8R | TAGGCAGTTTATGCTGCTTATGG |  |  |  |
| Expression analysis | eRTEL1-TNFRSF6BF | AGAGCGTCATGCAGGTCTTC | 60°C | 135bp | - |
|  | eRTEL1-TNFRSF6BR | TGCCACTCCGCGTACAG |  |  |  |
|  | eRTEL1F | CTCGCTGGACCAGATCATCC | 60°C | 112bp | - |
|  | eRTEL1R | GGGGTCCACACTGAACACAA |  |  |  |
