# Supplementary material for: Discovery of a Novel Shared Variant Among RTEL1 Gene and RTEL1-TNFRSF6B lncRNA at Chromosome 20q13.33 in Familial Progressive Myoclonus Epilepsy
Source: Int J Genomics. 2024 Aug 10;2024:7518528. doi: 10.1155/2024/7518528 (PMC11330336; doi:10.1155/2024/7518528)
Supplement: Supporting Information 5 — Output of mitochondrial genome sequencing data analysis for both cases of progressive myoclonus epilepsy. [file 7518528.f5.docx]

**Supplementary File 2-** Output of mitochondrial genome sequencing data analysis for both cases of progressive myoclonus epilepsy.

| **Sample ID** | **Variant description** | **Coverage** | **MeanQ** | **HF_manual** | **RSRS** | **MHCS** | **rCRS** | **Haplogroup** | ***Locus*** | **Nt Variability** | **Codon Position** | **Aa Change** | **Aa Variability** | **Disease Score** |
| --- | --- | --- | --- | --- | --- | --- | --- | --- | --- | --- | --- | --- | --- | --- |
| Case1 | m.14323G>A | 915 | 27.65 | 0.995628 | yes | yes | yes | H13a2a | *MT-ND6* | 0.029274 | 3 | syn | 0.0335 | NA |
|  | m.15110G>A | 7172 | 31.24 | 1 | yes | yes | yes | H13a2a | *MT-CYB* | 0.0367 | 1 | A122T | NA | 0.11 |
|  | m.709G>A | 7186 | 30.21 | 0.999443 | yes | yes | yes | H13a2a | *MT-RNR1* | 0.449 | NA | NA | NA | NA |
|  | m.8632T>C | 2104 | 28.94 | 0.998099 | yes | yes | yes | H13a2a | *MT-ATP6* | 0.00182 | 1 | Y36H | 0.0045 | 0.19 |
|  | m.5442T>C | 2855 | 30.53 | 0.998599 | yes | yes | yes | H13a2a | *MT-ND2* | 0.105 | 1 | F325L | 0.9383 | 0.1 |
|  | m.16519T>C | 1295 | 29.86 | 0.999228 |  |  | yes | H13a2a | *MT-DLOOP* | 1 | NA | NA | NA | NA |
|  | m.2581A>G | 7439 | 32.23 | 0.998925 | yes | yes | yes | H13a2a | *MT-RNR2* | 0.015021 | NA | NA | NA | NA |
|  | m.1008A>G | 4393 | 30.14 | 0.995447 | yes | yes | yes | H13a2a | *MT-RNR1* | 0.00454 | NA | NA | NA | NA |
|  | m.8860A>G | 1778 | 30.27 | 0.982002 |  |  | yes | H13a2a | *MT-ATP6* | 0.0273 | 1 | T112A | 0.026 | 0.14 |
|  | m.15326A>G | 5680 | 31.74 | 0.998768 |  |  | yes | H13a2a | *MT-CYB* | 0.0383 | 1 | T194A | NA | 0.12 |
|  | m.750A>G | 5732 | 30.16 | 0.998604 |  |  | yes | H13a2a | *MT-RNR1* | 0.056 | NA | NA | NA | NA |
|  | m.263A>G | 4235 | 31.85 | 0.998347 |  |  | yes | H13a2a | *MT-DLOOP* | 0.0869 | NA | NA | NA | NA |
|  | m.4769A>G | 4919 | 31.36 | 0.99939 |  |  | yes | H13a2a | *MT-ND2* | 0.0937 | 3 | NA | 0.0752 | NA |
|  | m.1438A>G | 5891 | 31.92 | 0.998981 |  |  | yes | H13a2a | *MT-RNR1* | 0.164 | NA | NA | NA | NA |
|  | m.14495A>T | 813 | 27.3 | 0.222632 | yes | yes | yes | H13a2a | *MT-ND6* | 8.86E-09 | 2 | Stop-gain | 0 | NA |
|  | m.2259C>T | 5975 | 31.58 | 0.999498 | yes | yes | yes | H13a2a | *MT-RNR2* | 0.0362 | NA | NA | NA | NA |
|  | m.14872C>T | 4605 | 31.75 | 0.999783 | yes | yes | yes | H13a2a | *MT-CYB* | 0.036801 | 3 | syn | 0.0065 | NA |
| Case2 | m.14323G>A | 356 | 27.9 | 0.997191 | yes | yes | yes | H13a2a | *MT-ND6* | 0.029274 | 3 | syn | 0.0335 | NA |
|  | m.15110G>A | 5661 | 31.16 | 0.99947 | yes | yes | yes | H13a2a | *MT-CYB* | 0.0367 | 1 | A122T | NA | 0.11 |
|  | m.709G>A | 2728 | 30.26 | 0.9989 | yes | yes | yes | H13a2a | *MT-RNR1* | 0.449 | NA | NA | NA | NA |
|  | m.8632T>C | 236 | 29.38 | 1 | yes | yes | yes | H13a2a | *MT-ATP6* | 0.00182 | 1 | Y36H | 0.0045 | 0.19 |
|  | m.5442T>C | 547 | 30.94 | 1 | yes | yes | yes | H13a2a | *MT-ND2* | 0.105 | 1 | F325L | 0.9383 | 0.1 |
|  | m.16519T>C | 789 | 30.34 | 1 |  |  | yes | H13a2a | *MT-DLOOP* | 1 | NA | NA | NA | NA |
|  | m.2581A>G | 2214 | 32.09 | 0.999097 | yes | yes | yes | H13a2a | *MT-RNR2* | 0.015021 | NA | NA | NA | NA |
|  | m.1008A>G | 1405 | 30.43 | 0.994306 | yes | yes | yes | H13a2a | *MT-RNR1* | 0.00454 | NA | NA | NA | NA |
|  | m.8860A>G | 337 | 30.71 | 0.991098 |  |  | yes | H13a2a | *MT-ATP6* | 0.0273 | 1 | T112A | 0.026 | 0.14 |
|  | m.15326A>G | 3416 | 31.1 | 0.997951 |  |  | yes | H13a2a | *MT-CYB* | 0.0383 | 1 | T194A | NA | 0.12 |
|  | m.750A>G | 2397 | 30.12 | 0.999583 |  |  | yes | H13a2a | *MT-RNR1* | 0.056 | NA | NA | NA | NA |
|  | m.263A>G | 2106 | 31.36 | 0.998101 |  |  | yes | H13a2a | *MT-DLOOP* | 0.0869 | NA | NA | NA | NA |
|  | m.4769A>G | 968 | 31.31 | 1 |  |  | yes | H13a2a | *MT-ND2* | 0.0937 | 3 | NA | 0.0752 | NA |
|  | m.1438A>G | 2239 | 31.58 | 0.999553 |  |  | yes | H13a2a | *MT-RNR1* | 0.164 | NA | NA | NA | NA |
|  | m.2259C>T | 976 | 31.66 | 0.996926 | yes | yes | yes | H13a2a | *MT-RNR2* | 0.0362 | NA | NA | NA | NA |
|  | m.14872C>T | 3166 | 31.61 | 0.998421 | yes | yes | yes | H13a2a | *MT-CYB* | 0.036801 | 3 | syn | 0.0065 | NA |

^syn:Synonymous; NA: Not available; Nt: Nucleotide; Aa: Amino acid^
