# Supplementary material for: Discovery of a Novel Shared Variant Among RTEL1 Gene and RTEL1-TNFRSF6B lncRNA at Chromosome 20q13.33 in Familial Progressive Myoclonus Epilepsy
Source: Int J Genomics. 2024 Aug 10;2024:7518528. doi: 10.1155/2024/7518528 (PMC11330336; doi:10.1155/2024/7518528)
Supplement: Supporting Information 6 — Output of copy number variation analysis in both the probands and their parents. [file 7518528.f6.docx]

**Supplementary File 3-** Output of copy number variation analysis in both the pro-bands and their parents.

|  | **Location** | **Type** | **Classification** | **CN** | **Call** | **Known or predicted dosage-sensitive genes** | **All protein coding genes** |
| --- | --- | --- | --- | --- | --- | --- | --- |
| **Case1** | chr11:89774267-89819939 | DEL | Uncertain significance | 1 | -2 |  | *TRIM49C, UBTFL1* |
|  | chr12:88547929-88554669 | DEL | Uncertain significance | 0 | -2 |  | *TMTC3* |
|  | chr13:21176036-21230638 | DEL | Uncertain significance | 1 | -1 |  | *IFT88* |
|  | chr14:73989673-74010122 | DEL | Uncertain significance | 1 | -2 |  | *HEATR4, ACOT1* |
|  | chr18:29450419-29497760 | DEL | Uncertain significance | 1 | -1 |  | *TRAPPC8* |
|  | chr18:76751963-76807682 | DUP | Uncertain significance | 4 | 2 |  | *SALL3* |
|  | chr18:9931760-9945126 | DEL | Uncertain significance | 1 | -1 |  | *VAPA* |
|  | chr19:52132204-52149718 | DEL | Benign | 1 | -1 |  | *SIGLEC5, SIGLEC14* |
|  | chr19:55340801-55354460 | DUP | Benign | 5 | 2 |  | *KIR3DL1, KIR2DS4* |
|  | chr2:98128015-98162328 | DEL | Benign | 1 | -1 |  | *ANKRD36B* |
|  | chr20:1579384-1592224 | DEL | Benign | 0 | -2 |  | *SIRPB1* |
|  | chr4:76522294-76539756 | DEL | Uncertain significance | 1 | -1 |  | *CDKL2* |
|  | chr5:150029672-150050325 | DEL | Uncertain significance | 1 | -1 |  | *SYNPO, MYOZ3* |
|  | chr8:10202-167432 | DEL | Uncertain significance | 1 | -1 |  | *OR4F21* |
|  | chr8:7274096-7810066 | DEL | Benign | 1 | -1 |  | *DEFB4B, DEFB103B, SPAG11B, DEFB104B, DEFB106B, DEFB105B, DEFB107B, PRR23D2, PRR23D1, DEFB107A, DEFB105A, DEFB106A, DEFB104A, SPAG11A, DEFB103A, DEFB4A, ZNF705B* |
|  | chr9:94877919-95019178 | DEL | Uncertain significance | 1 | -1 |  | *PRSS47, IARS1* |
| **Case2** | chr10:56288382-58059976 | DEL | Uncertain significance | 1 | -1 |  | *PCDH15, MTRNR2L5* |
|  | chr14:82800460-84900459 | DEL | Uncertain significance | 1 | -1 |  |  |
|  | chr19:20044090-24611055 | DEL | Uncertain significance | 1 | -1 |  | *ZNF93, ZNF682, ZNF90, ZNF486, ZNF737, ZNF626, ZNF66, ZNF85, ZNF430, ZNF714, ZNF431, ZNF708, ZNF738, ZNF493, ZNF429, ZNF100, ZNF43, ZNF208, ZNF257, ZNF676, ZNF729, ZNF98, ZNF492, ZNF99, ZNF723, ZNF728, ZNF730, ZNF724, ZNF91, ZNF675, ZNF681, RPSAP58, ZNF726, ZNF254* |
|  | chr2:180459929-181458358 | DEL | Uncertain significance | 1 | -1 |  | *ZNF385B, CWC22* |
|  | chr2:82625256-84785013 | DEL | Uncertain significance | 1 | -1 |  | *SUCLG1, DNAH6* |
|  | chr21:20676152-25910505 | DEL | Uncertain significance | 1 | -1 |  | *NCAM2* |
|  | chr21:45949610-46131478 | DEL | Uncertain significance | 1 | -1 |  | *TSPEAR, KRTAP10-1, KRTAP10-2, KRTAP10-3, KRTAP10-4, KRTAP10-5, KRTAP10-6, KRTAP10-7, KRTAP10-8, KRTAP10-9, KRTAP10-10, KRTAP10-11, KRTAP12-4, KRTAP12-3, KRTAP12-2, KRTAP12-1, KRTAP10-12* |
|  | chr21:9411392-11147911 | DEL | Uncertain significance | 1 | -1 |  | *TPTE, BAGE4, BAGE3, BAGE2, BAGE5, BAGE* |
|  | chr3:102246690-104246689 | DEL | Uncertain significance | 1 | -1 |  |  |
|  | chr6:60202-2226395 | DUP | Uncertain significance | 3 | 1 |  | *FOXQ1, FOXF2, FOXC1, GMDS, DUSP22, IRF4, EXOC2, HUS1B* |
|  | chr6:66355518-68955517 | DEL | Uncertain significance | 1 | -1 |  | *EYS* |
|  | chr7:61080041-62480040 | DEL | Uncertain significance | 0 | -2 |  |  |
|  | chr9:139983283-140167457 | DEL | Uncertain significance | 1 | -1 |  | *MAN1B1, DPP7, GRIN1, LRRC26, TMEM210, ANAPC2, SSNA1, TPRN, TMEM203, NDOR1, RNF208, CYSRT1, RNF224, SLC34A3, TUBB4B, FAM166A, STPG3, NELFB* |
|  | chr9:8884114-11634113 | DEL | Uncertain significance | 1 | -1 | *PTPRD* | *PTPRD* |
| **Mother** | chr1:169139123-169279395 | DEL | Uncertain significance | 1 | -1 |  | *NME7* |
|  | chr1:212119882-212148742 | DEL | Uncertain significance | 1 | -2 |  | *INTS7* |
|  | chr10:47894444-47945803 | DUP | Benign | 5 | 2 |  |  |
|  | chr10:51852911-51892743 | DEL | Uncertain significance | 1 | -1 |  | *WASHC2A* |
|  | chr12:37880056-38430055 | DEL | Uncertain significance | 1 | -1 |  |  |
|  | chr12:53085008-53089677 | DEL | Uncertain significance | 1 | -2 |  | *KRT77* |
|  | chr18:11689564-13042371 | DEL | Uncertain significance | 2 | -2 |  | *GNAL, CHMP1B, MPPE1, IMPA2, ANKRD62, CIDEA, TUBB6, AFG3L2, PRELID3A, SPIRE1, CEP76, PSMG2, PTPN2, SEH1L, CEP192* |
|  | chr18:76740122-76757482 | DEL | Uncertain significance | 1 | -1 |  | *SALL3* |
|  | chr20:1579384-1592224 | DUP | Benign | 9 | 2 |  | *SIRPB1* |
|  | chr8:11970369-12285394 | DEL | Uncertain significance | 0 | -2 |  | *ZNF705D, USP17L7, USP17L2, FAM86B1, DEFB130A, FAM86B2* |
|  | chr8:7114249-8176826 | DEL | Uncertain significance | 1 | -1 |  | *USP17L1, USP17L4, ZNF705G, DEFB4B, DEFB103B, SPAG11B, DEFB104B, DEFB106B, DEFB105B, DEFB107B, PRR23D2, PRR23D1, DEFB107A, DEFB105A, DEFB106A, DEFB104A, SPAG11A, DEFB103A, DEFB4A, ZNF705B, USP17L8, USP17L3, DEFB109B, PRAG1* |
|  | chr9:69400383-69424215 | DEL | Uncertain significance | 1 | -2 |  |  |
| **Father** | chr14:105936174-105996169 | DEL | Uncertain significance | 1 | -1 |  | *MTA1, CRIP2, CRIP1, TEDC1, TMEM121* |
|  | chr19:52132685-52217408 | DEL | Uncertain significance | 0 | -2 |  | *SIGLEC5, SIGLEC14, SPACA6, HAS1* |
|  | chr7:82538152-83095969 | DUP | Uncertain significance | 3 | 1 |  | *PCLO, SEMA3E* |
|  | chrX:125686888-127286438 | DEL | Uncertain significance | 1 | -1 |  | *PRR32, ACTRT1* |
