# Supplementary material for: Discovery of a Novel Shared Variant Among RTEL1 Gene and RTEL1-TNFRSF6B lncRNA at Chromosome 20q13.33 in Familial Progressive Myoclonus Epilepsy
Source: Int J Genomics. 2024 Aug 10;2024:7518528. doi: 10.1155/2024/7518528 (PMC11330336; doi:10.1155/2024/7518528)
Supplement: Supporting Information 7 — Family-based segregation analysis and information of shortlisted candidate genes and variants identified in whole exome sequencing of both sibling cases of PMEs. [file 7518528.f7.docx]

**Supplementary File 4-** Family based segregation analysis and information of shortlisted candidate genes and variants identified in whole exome sequencing of both sibling cases of PMEs.

|  | **Locus** | **Genotype** | **Father** | **Mother** | **Zygosity** | **VarAFT** | **ClinVar** | **ACMG classification** | **Criteria** | **Ref** | **Observed Allele** | **Type** | **Genes** | **pLi** | **Location/Transcript** | **Exon** | **Coding** | **Amino Acid Change** | **Variant Effect** | **dbSNP** |
| --- | --- | --- | --- | --- | --- | --- | --- | --- | --- | --- | --- | --- | --- | --- | --- | --- | --- | --- | --- | --- |
| Case 1 | chrX:49082499 | T/T | C/C | C/T | Homo | Probably pathogenic | Benign/Likely benign | Uncertain significance | PP3+BP6 | C | T | SNV | *CACNA1F* | 0 | CACNA1F:exonic:NM_001256789.3 | 13 | c.1523G>A | p.Arg508Gln | missense | rs34162630 |
|  | chr6:30624241 | C/C | T/C | T/C | Homo | Pathogenic |  | Uncertain significance | PP3 | T | C | SNV | *DHX16* | 0 | DHX16:exonic:NM_003587.5 | 15 | c.2357A>G | p.Tyr786Cys | missense | rs149087216 |
|  | chr18:28574248 | A/A | C/A | C/A | Homo | Pathogenic |  | Uncertain significance | PM2+PP3 | C | A | SNV | *DSC3* | 0 | DSC3:exonic:NM_001941.5 | 16 | c.2584G>T | p.Gly862Cys | missense | rs528760662 |
|  | chr18:19031007 | T/T | G/T | G/T | Homo | Pathogenic |  | Uncertain significance | PM2+PP3 | G | T | SNV | *GREB1L* | 1 | GREB1L:exonic:NM_001142966.2 | 13 | c.1744G>T | p.Ala582Ser | missense | rs1290064070 |
|  | chr3:19295367 | C/C | T/C | T/C | Homo | Pathogenic |  | Uncertain significance | PP3+PP1 | T | C | SNV | *KCNH8* | 0 | KCNH8:exonic:NM_144633.3 | 2 | c.298T>C | p.Tyr100His | missense | rs565744148 |
|  | chrX:117079458 | T/T | G/G | G/T | Homo | Pathogenic |  | Uncertain significance | PM2+PP3 | G | T | SNV | *KLHL13* | 0.04 | KLHL13:exonic:NM_001168299.1 | 3 | c.188C>A | p.Ser63Tyr | missense | rs377144272 |
|  | chr3:12626038 | A/A | G/A | G/A | Homo | Pathogenic | Pathogenic | Uncertain significance | PM2+PP3+PP5+PP1 | G | A | SNV | *MKRN2,RAF1* | 0.85 | MKRN2:downstream:NM_014160.5, RAF1:exonic:NM_002880.4 | 17 | c.*2286G>A, c.1922C>T | p.?, p.Thr641Met | unknown, missense | rs587777587 |
|  | chr13:77779474 | C/C | T/C | T/C | Homo | Probably pathogenic |  | Uncertain significance | PM2+PP3 | T | C | SNV | *MYCBP2* | 1 | MYCBP2:exonic:NM_015057.5 | 26 | c.3760A>G | p.Ile1254Val | missense | rs573877665 |
|  | chr20:62298898 | T/T | G/T | G/T | Homo | Pathogenic |  | Uncertain significance | PM2+PP3+PP1 | G | T | SNV | *RTEL1,RTEL1-TNFRSF6B* | 0 | RTEL1-TNFRSF6B:exonic_nc:NR_037882.1, RTEL1:exonic:NM_032957.5 | 8 | c.763G>T | p.Asp255Tyr | missense |  |
|  | chr13:78318497 | A/A | T/A | T/A | Homo | Probably pathogenic |  | Uncertain significance | PM2+PP3 | T | A | SNV | *SLAIN1* | 0.9 | SLAIN1:exonic:NM_001242868.2 | 3 | c.846T>A | p.Asp282Glu | missense | rs531297065 |
|  | chr4:155157160 | G/T | G/T | G/G | Compound Hetero | Probably pathogenic |  | Uncertain significance | PM3+PP3 | G | T | SNV | *DCHS2* | 0 | DCHS2:exonic:NM_001358235.2 | 20 | c.8644C>A | p.Gln2882Lys | missense | rs200042662 |
|  | chr4:155254454 | A/C | A/A | A/C | Compound Hetero | Pathogenic |  | Uncertain significance | PM3+PP3 | A | C | SNV | *DCHS2* | 0 | DCHS2:exonic:NM_001358235.2 | 5 | c.2906T>G | p.Val969Gly | missense | rs147645073 |
|  | chr17:18052531 | G/A | G/A | A/A | Compound Hetero | Polymorphism |  | Uncertain significance | PM3 | G | A | SNV | *MYO15A* | 0 | MYO15A:exonic:NM_016239.4 | 33 | c.6958G>A | p.Val2320Met | missense | rs1308687672 |
|  | chr17:18046894 | G/A | G/G | G/A | Compound Hetero | Pathogenic | Conflicting interpretations of pathogenicity | Uncertain significance | PP3 | G | A | SNV | *MYO15A* | 0 | MYO15A:exonic:NM_016239.4 | 25 | c.5925G>A | p.Trp1975Ter | nonsense | rs375290498 |
|  | chr8:133899451 | A/A | C/A | C/A | Homo/Compound Hetero | Polymorphism | Conflicting interpretations of pathogenicity | Uncertain significance | PM2+PM3 | C | A | SNV | *TG* | 0 | TG:exonic:NM_003235.5 | 9 | c.1834C>A | p.Pro612Thr | missense | rs552359043 |
|  | chr8:134025976 | T/T | C/T | C/T | Homo/Compound Hetero | Pathogenic | Uncertain significance | Uncertain significance | PM2+PP3 | C | T | SNV | *TG* | 0 | TG:exonic:NM_003235.5 | 37 | c.6529C>T | p.Arg2177Cys | missense | rs555719562 |
|  | chr2:179438746 | G/A | G/A | G/G | Compound Hetero | Probably pathogenic | Benign/Likely benign | Uncertain significance | PM3+BP6+PP3 | G | A | SNV | *TTN,TTN-AS1* | 0 | TTN-AS1:intronic_nc:NR_038272.1, TTN:exonic:NM_001256850.1 | 276 | c.67190C>T | p.Thr22397Met | missense | rs370375696 |
|  | chr2:179392277 | A/G | A/A | A/G | Compound Hetero | Pathogenic | Conflicting interpretations of pathogenicity | Uncertain significance | PP3 | A | G | SNV | *TTN,TTN-AS1* | 0 | TTN-AS1:intronic_nc:NR_038272.1, TTN:exonic:NM_001256850.1 | 312 | c.102653T>C | p.Met34218Thr | missense | rs72629793 |
|  | chr2:179599473 | C/G | C/C | C/G | Compound Hetero | Probable polymorphism | Conflicting interpretations of pathogenicity | Uncertain significance | PM3 | C | G | SNV | *TTN* | 0 | TTN:exonic:NM_001256850.1 | 49 | c.14227G>C | p.Val4743Leu | missense | rs72648929 |
| Case 2 | chr3:12626038 | A/A | G/A | G/A | Homo | Pathogenic | Pathogenic | Uncertain significance | PM2+PP3+PP5+PP1 | G | A | SNV | *MKRN2,RAF1* | 0.85 | MKRN2:downstream:NM_014160.5, RAF1:exonic:NM_002880.4 | 17 | c.*2286G>A, c.1922C>T | p.?, p.Thr641Met | unknown, missense | rs587777587 |
|  | chr3:19295367 | C/C | T/C | T/C | Homo | Pathogenic |  | Uncertain significance | PP3+PP1 | T | C | SNV | *KCNH8* | 0 | KCNH8:exonic:NM_144633.3 | 2 | c.298T>C | p.Tyr100His | missense | rs565744148 |
|  | chr14:23821321 | T/T | C/T | C/T | Homo | Pathogenic |  | Uncertain significance | PM2+PP3 | C | T | SNV | *SLC22A17* | 0.01 | SLC22A17:exonic:NM_020372.3 | 1 | c.103G>A | p.Gly35Ser | missense | rs756794632 |
|  | chr14:24774961 | A/A | G/A | G/A | Homo | Pathogenic |  | Uncertain significance | PM2+PP3 | G | A | SNV | *CIDEB,NOP9* | 0 | CIDEB:exonic:NM_014430.3, NOP9:utr_3:NM_174913.3 | 7 | c.*660G>A, c.538C>T | p.?, p.Arg180Cys | unknown, missense | rs186107928 |
|  | chr19:8808706 | C/C | T/C | T/C | Homo | NA |  | Uncertain significance | PM2 | T | C | SNV | *ACTL9* | 0 | ACTL9:exonic:NM_178525.5 | 1 | c.346A>G | p.Ser116Gly | missense | rs567990063 |
|  | chr19:9868501 | T/T | C/T | C/T | Homo | Pathogenic |  | Uncertain significance | PM2+PP3 | C | T | SNV | *ZNF846* | 0 | ZNF846:exonic:NM_001077624.2 | 6 | c.1252G>A | p.Gly418Arg | missense | rs78118057 |
|  | chr19:11473328 | T/T | C/T | C/T | Homo | Pathogenic |  | Uncertain significance | PM2+PP3 | C | T | SNV | *PLPPR2* | 0.99 | PLPPR2:exonic:NM_001170635.2 | 7 | c.728C>T | p.Ala243Val | missense | rs765809903 |
|  | chr20:62298898 | T/T | G/T | G/T | Homo | Pathogenic |  | Uncertain significance | PM2+PP3+PP1 | G | T | SNV | *RTEL1,RTEL1-TNFRSF6B* | 0 | RTEL1-TNFRSF6B:exonic_nc:NR_037882.1, RTEL1:exonic:NM_032957.5 | 8 | c.763G>T | p.Asp255Tyr | missense |  |
|  | chr16:16173256 | A/C | A/C | A/A | Compound Hetero | Pathogenic |  | Uncertain significance | PM3+PP3 | A | C | SNV | *ABCC1* | 0 | ABCC1:exonic:NM_004996.4 | 16 | c.2036A>C | p.Gln679Pro | missense | rs551578363 |
|  | chr16:16208739 | C/T | C/C | C/T | Compound Hetero | Pathogenic |  | Uncertain significance | PM3+PP3 | C | T | SNV | *ABCC1* | 0 | ABCC1:exonic:NM_004996.4 | 23 | c.3196C>T | p.Arg1066Trp | missense | rs199773531 |
|  | chr8:113253954 | A/G | A/G | A/A | Compound Hetero | Probable polymorphism |  | Uncertain significance | PM3 | A | G | SNV | *CSMD3* | 0.06 | CSMD3:exonic:NM_198123.2 | 66 | c.10463T>C | p.Leu3488Pro | missense | rs61753736 |
|  | chr8:113304889 | T/C | T/T | T/C | Compound Hetero | Pathogenic |  | Uncertain significance | PP3 | T | C | SNV | *CSMD3* | 0.06 | CSMD3:exonic:NM_198123.2 | 55 | c.8665A>G | p.Asn2889Asp | missense | rs770903060 |
|  | chr2:234235842 | A/G | A/G | A/A | Compound Hetero | Probably pathogenic | Conflicting interpretations of pathogenicity | Uncertain significance | PP3 | A | G | SNV | *SAG* | 0 | SAG:exonic:NM_000541.5 | 7 | c.511A>G | p.Lys171Glu | missense | rs552862207 |
|  | chr2:234229395 | G/A | G/G | G/A | Compound Hetero | Polymorphism | Conflicting interpretations of pathogenicity | Uncertain significance | PM3 | G | A | SNV | *SAG* | 0 | SAG:exonic:NM_000541.5 | 5 | c.301G>A | p.Ala101Thr | missense | rs141521563 |
|  | chr2:179644761 | T/A | T/A | T/T | Compound Hetero | Pathogenic | Uncertain significance | Uncertain significance | PP3 | T | A | SNV | *LOC101927055,TTN* | 0 | LOC101927055:downstream:NR_120594.1, TTN:exonic:NM_001256850.1 | 22 | c.3695A>T | p.Asp1232Val | missense | rs576019235 |
|  | chr2:179599473 | C/G | C/C | C/G | Compound Hetero | Probable polymorphism | Conflicting interpretations of pathogenicity | Uncertain significance | PM3 | C | G | SNV | *TTN* | 0 | TTN:exonic:NM_001256850.1 | 49 | c.14227G>C | p.Val4743Leu | missense | rs72648929 |
