# Supplementary material for: Discovery of a Novel Shared Variant Among RTEL1 Gene and RTEL1-TNFRSF6B lncRNA at Chromosome 20q13.33 in Familial Progressive Myoclonus Epilepsy
Source: Int J Genomics. 2024 Aug 10;2024:7518528. doi: 10.1155/2024/7518528 (PMC11330336; doi:10.1155/2024/7518528)
Supplement: Supporting Information 8 — Information on various targets of RTEL1-TNFRSF6B RNA obtained from the RNAInter database. [file 7518528.f8.docx]

**Supplementary File 5-** Information on various targets *of RTEL1-TNFRSF6B* RNA obtained from the RNAInter database.

| **Interactor1** | **Category1** | **Interactor2** | **Category2** | **Score*** |
| --- | --- | --- | --- | --- |
| RTEL1-TNFRSF6B | circRNA | DKC1 | RBP | 0.4155 |
| RTEL1-TNFRSF6B | circRNA | DHX9 | RBP | 0.2559 |
| RTEL1-TNFRSF6B | circRNA | DICER1 | RBP | 0.2249 |
| RTEL1-TNFRSF6B | circRNA | ADAR | RBP | 0.2204 |
| RTEL1-TNFRSF6B | circRNA | AIFM1 | RBP | 0.2331 |
| RTEL1-TNFRSF6B | circRNA | ACIN1 | RBP | 0.2083 |
| RTEL1-TNFRSF6B | circRNA | DDX54 | RBP | 0.2105 |
| hsa-miR-6806-3p | miRNA | RTEL1-TNFRSF6B | mRNA | 0.231 |
| hsa-miR-1976 | miRNA | RTEL1-TNFRSF6B | mRNA | 0.231 |
| hsa-miR-5008-5p | miRNA | RTEL1-TNFRSF6B | mRNA | 0.231 |
| hsa-miR-6729-5p | miRNA | RTEL1-TNFRSF6B | mRNA | 0.231 |
| hsa-miR-632 | miRNA | RTEL1-TNFRSF6B | mRNA | 0.2568 |
| hsa-miR-3652 | miRNA | RTEL1-TNFRSF6B | mRNA | 0.2568 |
| hsa-mir-615-5p | miRNA | RTEL1-TNFRSF6B | mRNA | 0.2566 |
| hsa-mir-615-3p | miRNA | RTEL1-TNFRSF6B | mRNA | 0.2566 |
| hsa-miR-181a-5p | miRNA | RTEL1-TNFRSF6B | mRNA | 0.2152 |
| hsa-miR-132-3p | miRNA | RTEL1-TNFRSF6B | mRNA | 0.2152 |
| MALAT1 | lncRNA | RTEL1-TNFRSF6B | DNA | 0.2573 |
| LOC101928674 | lncRNA | RTEL1-TNFRSF6B | DNA | 0.2358 |
| RMRP | lncRNA | RTEL1-TNFRSF6B | DNA | 0.2358 |
| AL353615.1 | lncRNA | RTEL1-TNFRSF6B | DNA | 0.2358 |
| RN7SK | others | RTEL1-TNFRSF6B | DNA | 0.2482 |
