# Supplementary material for: Discovery of a Novel Shared Variant Among RTEL1 Gene and RTEL1-TNFRSF6B lncRNA at Chromosome 20q13.33 in Familial Progressive Myoclonus Epilepsy
Source: Int J Genomics. 2024 Aug 10;2024:7518528. doi: 10.1155/2024/7518528 (PMC11330336; doi:10.1155/2024/7518528)
Supplement: Supporting Information 9 — Centroid RNA secondary structure prediction using RNAfold for RTEL1-TNFRSF6B with missense pathogenic or likely pathogenic variants reported in ClinVar database. [file 7518528.f9.docx]

**Supplementary File 6-** Centroid RNA secondary structure prediction using RNAfold for *RTEL1-TNFRSF6B* with missense pathogenic or likely pathogenic variants reported in ClinVar database

| **Variant details**  **[RTEL1-TNFRSF6B; NR_037882.1; GRCh37)** | **Minimum free energy** | **Centroid Secondary structure** |
| --- | --- | --- |
| No mutation | -2038.38kcal/mol | 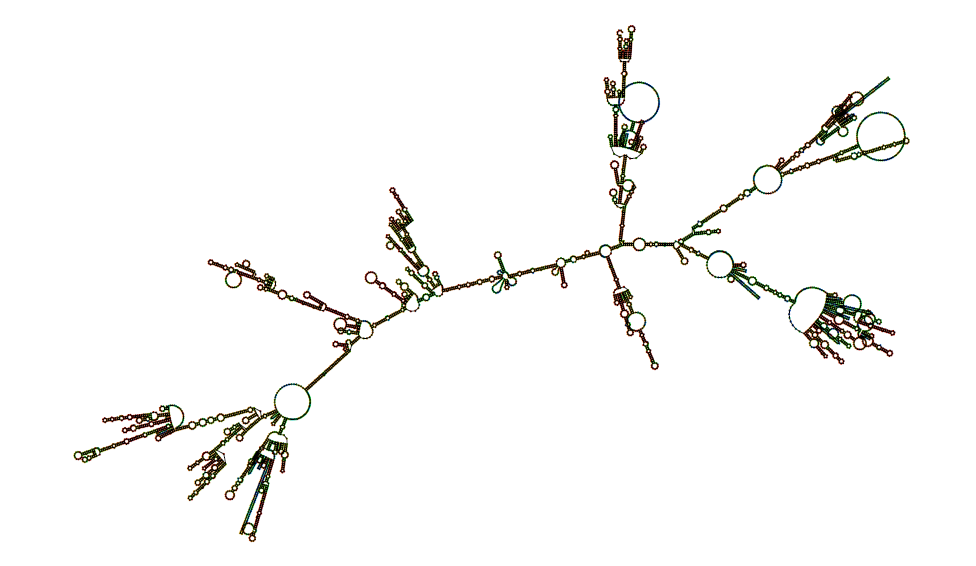 |
| Chr20:62290804C>T | -2043.98kcal/mol | 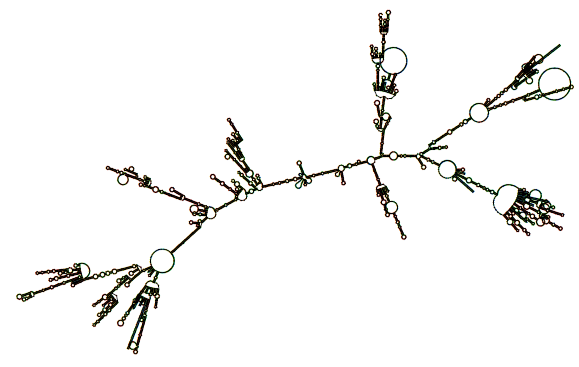 |
| Chr20:62319021C>T | -2059.58kcal/mol | 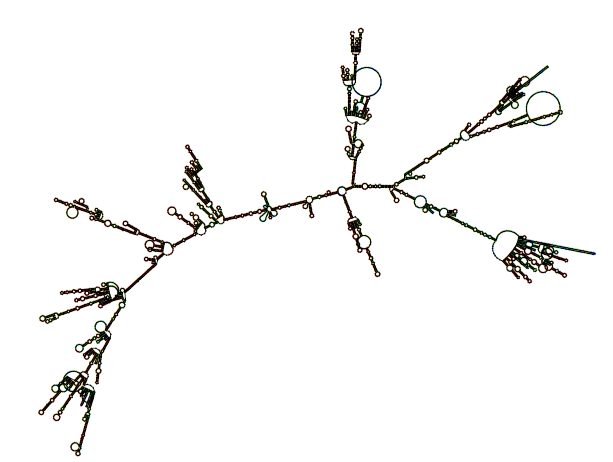 |
| Chr20:62319118G>T | -1999.26kcal/mol | 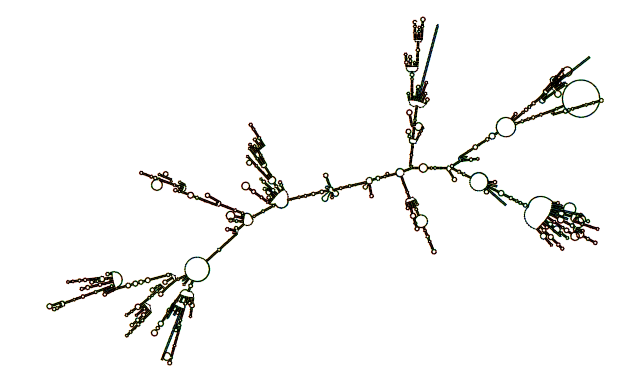 |
| Chr20:62319354G>C | -2131.51kcal/mol | 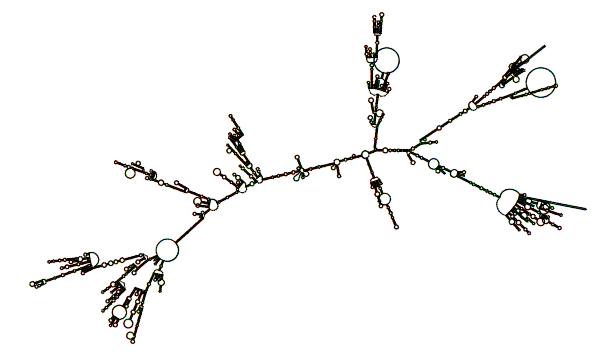 |
| Chr20:62319931G>T | -2041.88kcal/mol | 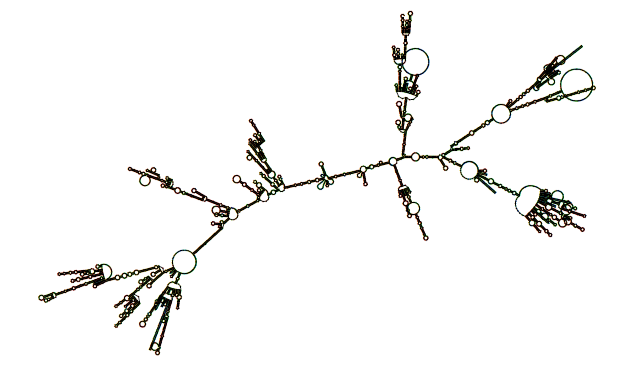 |
| Chr20:62320468G>A | -1832.89kcal/mol | 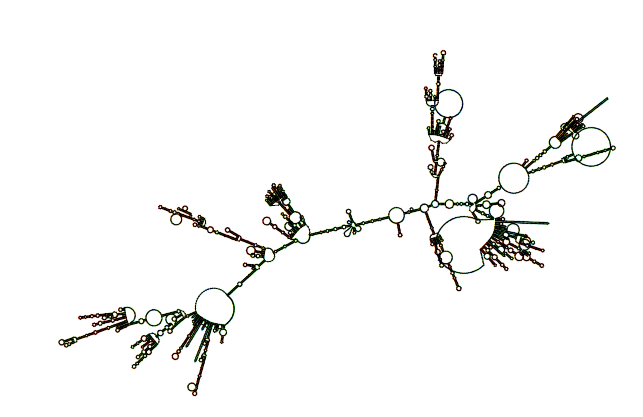 |
| Chr20:62320936_C>A | -2120.31kcal/mol | 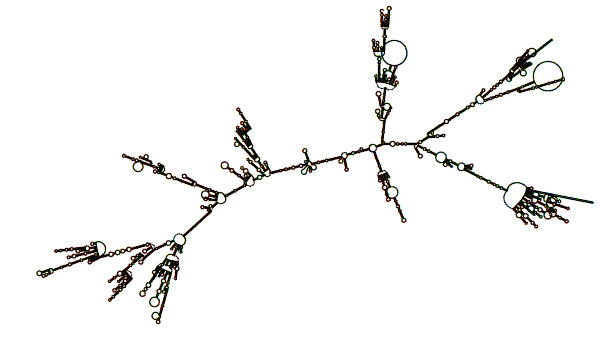 |
| Chr20:62321174C>G | -1970.47kcal/mol | 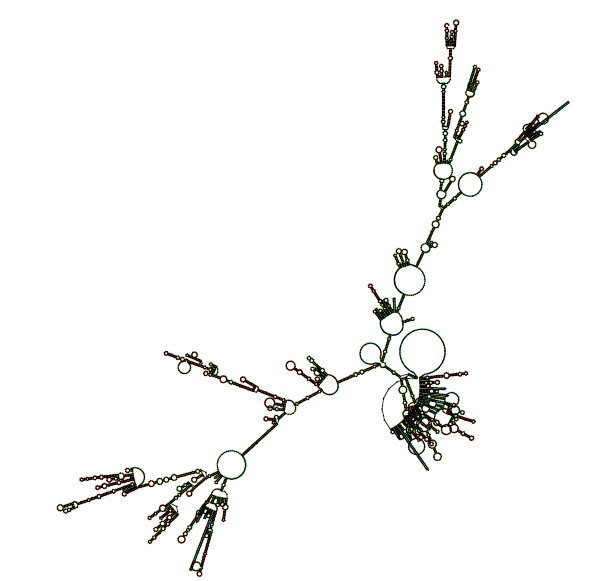 |
| Chr20:62326259_T>C | -2045.88kcal/mol | 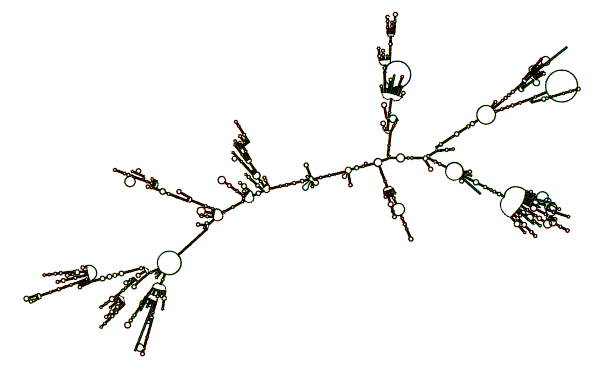 |
| Chr20:62326446_A>C | -2090.15kcal/mol | 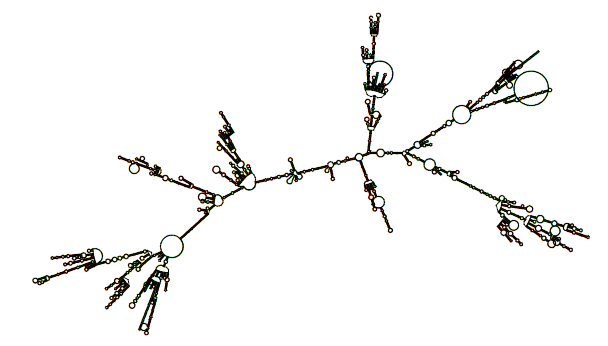 |
| Chr20:62326972G>A | -2057.09kcal/mol | 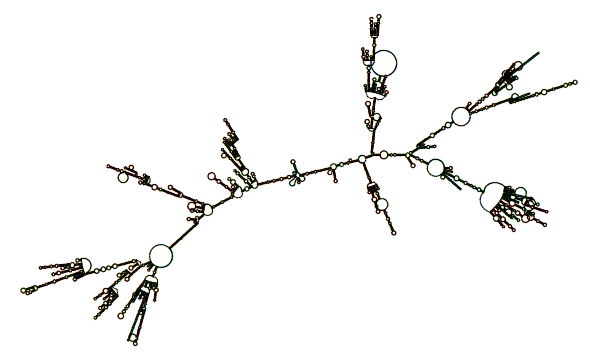 |
| **Chr20:62298898_G>T**  **(This manuscript)** | **-1501.32kcal/mol** | 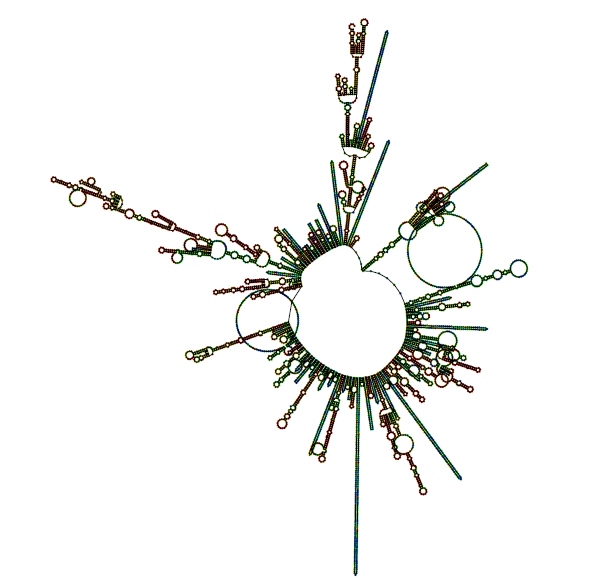 |
