# Supplementary material for: Discovery of a Novel Shared Variant Among RTEL1 Gene and RTEL1-TNFRSF6B lncRNA at Chromosome 20q13.33 in Familial Progressive Myoclonus Epilepsy
Source: Int J Genomics. 2024 Aug 10;2024:7518528. doi: 10.1155/2024/7518528 (PMC11330336; doi:10.1155/2024/7518528)
Supplement: Supporting Information 10 — Computational analysis for hsa-miR3529-3p and hsa-miR-5689 and their respective target genes for identification of significantly and inversely correlated target genes, followed by enrichment of the shortlisted genes based on Gene Ontology. [file 7518528.f10.pdf]

hsa-miR-3529-3p

**Target gene**	**Correlation value**
SLC35E2B	-0.737644815
DFFB	-0.730102077
TARDBP	-0.711080125
FBXO6	-0.934770426
PRDM2	-0.714395901
IFFO2	-0.706928498
HP1BP3	-0.786143824
NBPF3	-0.738595926
ELOA	-0.721772149
SRSF10	-0.72511472
PDIK1L	-0.797480183
AIM1L	-0.731351362
ARID1A	-0.736777177
AHDC1	-0.742200903
EYA3	-0.748662839
GMEB1	-0.718604301
TMEM200B	-0.749440513
PUM1	-0.826640625
KHDRBS1	-0.764795105
TXLNA	-0.725155361
RBBP4	-0.785662151
TFAP2E	-0.708598468
AGO3	-0.70131917
HIVEP3	-0.710549615
BTF3L4	-0.765615231
ORC1	-0.712311343
PRKAA2	-0.766520265
MYSM1	-0.718037078
SLC35D1	-0.76567087
PRKACB	-0.742813237
BCL10	-0.77989263
PKN2	-0.702246808
EVI5	-0.705371272
DR1	-0.758708815
ARHGAP29	-0.700101783
TMEM56	-0.779610497
DBT	-0.841266521
GNAI3	-0.76830218
DDX20	-0.748582485
ST7L	-0.747948774
RSBN1	-0.756024756
BCL2L15	-0.718156042
HIPK1	-0.742389557
NBPF8	-0.701758184
NBPF15	-0.71145615

NBPF20	-0.702023932
POLR3C	-0.758331583
NBPF12	-0.7029217
NBPF9	-0.710988816
RPRD2	-0.769695125
SETDB1	-0.718566945
SNX27	-0.76844062
THEM4	-0.765470843
PYGO2	-0.783374419
KIAA0907	-0.724531574
MEF2D	-0.746921509
DCAF8	-0.725587784
POU2F1	-0.725379081
SLC19A2	-0.743432787
VAMP4	-0.762876391
SUCO	-0.794942382
RALGPS2	-0.75601817
TOR1AIP2	-0.790455551
EDEM3	-0.766869982
DENND1B	-0.833541692
KDM5B	-0.768729344
TMEM183A	-0.760724626
AVPR1B	-0.706086292
INTS7	-0.916501268
RAB3GAP2	-0.785774408
MIA3	-0.806137012
AIDA	-0.802597758
LIN9	-0.74560237
IBA57	-0.726582526
IRF2BP2	-0.703158173
ARID4B	-0.701638133
GNG4	-0.785932433
ERO1B	-0.737737646
RGS7	-0.746658625
DESI2	-0.744686695
HNRNPU	-0.725804883
ZNF670	-0.74409579
SMC6	-0.71284228
PUM2	-0.7454847
DNMT3A	-0.743002909
SLC35F6	-0.761842115
PPM1G	-0.794373456
LCLAT1	-0.863317468
MEMO1	-0.730011063
GPATCH11	-0.798662623
NDUFAF7	-0.781973989
ATL2	-0.801879167

HNRNPLL	-0.772152228
SOS1	-0.732147661
MAP4K3	-0.735937835
PSME4	-0.702978809
CLHC1	-0.730869705
PPP4R3B	-0.77441713
PUS10	-0.786300296
EHBP1	-0.745716106
SERTAD2	-0.700323726
CEP68	-0.761922665
FBXO48	-0.734791197
GFPT1	-0.794206091
AAK1	-0.753001628
ZNF638	-0.724973635
EGR4	-0.732432584
ST3GAL5	-0.703765441
IMMT	-0.783026958
PLGLB1	-0.712038902
PLGLB2	-0.711472838
TMEM127	-0.701898353
TMEM131	-0.774904084
COA5	-0.744359196
AFF3	-0.737835494
LONRF2	-0.856250501
RNF149	-0.720274544
ZC3H6	-0.724572941
PTPN4	-0.735559105
EPB41L5	-0.714573437
TSN	-0.832583428
CCNT2	-0.729045812
LRP1B	-0.769020206
MBD5	-0.7599945
ARL5A	-0.799530314
CACNB4	-0.778235201
KCNJ3	-0.726034187
GPD2	-0.802585637
TTC21B	-0.733771302
SSB	-0.844401185
SP3	-0.747343777
ATF2	-0.791809295
PLEKHA3	-0.79566648
ZNF804A	-0.830123874
PMS1	-0.751513341
GLS	-0.718552728
STK17B	-0.730681921
INO80D	-0.718099981
CREB1	-0.730044438

KANSL1L	-0.736179058
USP37	-0.702987492
FAM134A	-0.775459993
CUL3	-0.784714016
TRIP12	-0.764756287
EIF4E2	-0.757745525
RBM44	-0.739421183
CNTN4	-0.749608265
SETD5	-0.719846567
ATP2B2	-0.757664013
VGLL4	-0.734846949
TSEN2	-0.771648037
NR2C2	-0.742994286
CLASP2	-0.71877251
PDCD6IP	-0.718898881
LRRFIP2	-0.71653028
WDR48	-0.791797883
GORASP1	-0.723773611
EIF1B	-0.786934789
CTNNB1	-0.734028046
TCAIM	-0.754761609
PRKAR2A	-0.748665748
NICN1	-0.747589443
CACNA2D2	-0.755008257
PHF7	-0.753067438
PBRM1	-0.716070596
DCP1A	-0.735443169
FAM208A	-0.716048101
APPL1	-0.740265055
RPP14	-0.774631155
CGGBP1	-0.776746537
TBC1D23	-0.737511595
CD47	-0.762253423
QTRT2	-0.789552242
POGLUT1	-0.763589738
GSK3B	-0.801230587
EPHB1	-0.785632944
MSL2	-0.72888729
NCK1	-0.745988991
CLDN18	-0.704577377
PIK3CB	-0.793649042
SLC25A36	-0.767881323
RASA2	-0.741285099
XRN1	-0.716302259
U2SURP	-0.721698683
PBX2P1	-0.783393868
COMMD2	-0.716863847

TSC22D2	-0.7758784
DHX36	-0.833036214
PLCH1	-0.790494866
PHC3	-0.722450519
TBL1XR1	-0.790745632
USP13	-0.742578404
TMEM41A	-0.718425465
ADIPOQ	-0.709306624
FYTTD1	-0.769124955
NSD2	-0.740981704
AFAP1	-0.766562534
FBXL5	-0.753735885
MED28	-0.74961739
PPARGC1A	-0.764407501
TBC1D1	-0.742025119
N4BP2	-0.762874752
KCTD8	-0.75900406
CLOCK	-0.75695908
AASDH	-0.733935565
ADGRL3	-0.882520093
SLC4A4	-0.79666152
EPGN	-0.757641249
CCNG2	-0.721344627
CXCL13	-0.718514419
LIN54	-0.731090154
GPAT3	-0.706499177
HERC3	-0.76315107
RP11.9B6.1	-0.708719411
SMARCAD1	-0.738535837
TSPAN5	-0.725737083
TIFA	-0.703586882
SPRY1	-0.810866872
HSPA4L	-0.80468327
NAA15	-0.750535233
ELMOD2	-0.742006984
FSTL5	-0.722897263
TMEM192	-0.801629684
ING2	-0.870037578
CASP3	-0.713790542
UFSP2	-0.820885613
CCDC127	-0.770301879
X44991	-0.799719194
ZFR	-0.776551668
PRKAA1	-0.700515682
PLCXD3	-0.721813822
TMEM267	-0.756228995
PELO	-0.880549965

MIER3	-0.710368911
GPBP1	-0.735165735
CWC27	-0.835970031
TRIM23	-0.768691928
SREK1	-0.750506359
SLC30A5	-0.79744788
CENPH	-0.741141947
MRPS27	-0.768851157
TNPO1	-0.701881247
FAM169A	-0.813025104
HMGCR	-0.721009806
SCAMP1	-0.707791491
JMY	-0.76348829
MTX3	-0.79868472
RASA1	-0.75271008
MBLAC2	-0.779228603
LYSMD3	-0.725113202
KIAA0825	-0.831967151
ERAP1	-0.867040569
LNPEP	-0.737390684
RIOK2	-0.768481449
FER	-0.74243885
NREP	-0.716489526
DCP2	-0.764154479
FEM1C	-0.796508418
DTWD2	-0.73395178
CEP120	-0.707538205
PRRC1	-0.700182203
RAPGEF6	-0.701062234
FNIP1	-0.723396509
SLC22A5	-0.723793393
KIF3A	-0.737274573
AFF4	-0.748789348
SMAD5	-0.701181415
SPOCK1	-0.706914718
HNRNPA0	-0.773192495
PCDHA2	-0.714294884
YIPF5	-0.805142261
RBM27	-0.765445944
FAM114A2	-0.856561322
MFAP3	-0.777288024
SLIT3	-0.726838852
FBXW11	-0.763715883
RNF44	-0.744843414
ZNF454	-0.774400256
CNOT6	-0.733053554
TRIM7	-0.701284488

C6orf201	-0.718289694
SSR1	-0.738052965
TMEM170B	-0.755179035
HIVEP1	-0.746129816
SIRT5	-0.769016385
JARID2	-0.71948265
ATXN1	-0.707017597
NUP153	-0.737412511
SOX4	-0.746457077
MICA	-0.768846605
PBX2	-0.777471916
UQCC2	-0.876280269
BRPF3	-0.748047637
RNF8	-0.783487479
TAF8	-0.76177942
GLTSCR1L	-0.742347701
MRPL2	-0.766011103
RP11.74E24.2	-0.710590153
ADGRB3	-0.78783523
PHIP	-0.703741291
ELOVL4	-0.82022613
SNAP91	-0.749930288
ZNF292	-0.717370282
RRAGD	-0.715739404
MDN1	-0.723971405
FUT9	-0.73673315
FAXC	-0.76631859
RPF2	-0.813204682
MARCKS	-0.717852851
CEP85L	-0.714296668
TBC1D32	-0.843630377
CLVS2	-0.732608661
RNF217	-0.722460648
TRMT11	-0.723363375
ARFGEF3	-0.704606839
ADAT2	-0.723428549
PEX3	-0.804211633
RGS17	-0.784395487
OPRM1	-0.717414989
WTAP	-0.766905603
MAP3K4	-0.735272511
FOXK1	-0.75762833
RBAK	-0.727124249
GLCCI1	-0.753647937
PHF14	-0.794179173
ETV1	-0.701675297
SP4	-0.732435241

TRA2A	-0.72591695
STK31	-0.701676712
HNRNPA2B1	-0.704031119
UBE2D4	-0.716662257
TMED4	-0.745872832
VWC2	-0.709615914
ZNF736	-0.79498971
BAZ1B	-0.726206903
GTF2I	-0.763545958
PHTF2	-0.736820549
CLDN12	-0.825242152
VPS50	-0.755362892
BET1	-0.741401783
PPP1R9A	-0.811377799
SMURF1	-0.765032306
ORAI2	-0.777733829
RELN	-0.702321618
BMT2	-0.835679098
ST7	-0.742147663
KCND2	-0.729811081
ING3	-0.720710514
FAM71F2	-0.740982452
CHCHD3	-0.802855686
TTC26	-0.889215594
UBN2	-0.725319144
KDM7A	-0.701499476
ZNF783	-0.726071678
NUB1	-0.742402035
DNAJB6	-0.756115784
FBXO25	-0.751606117
ERI1	-0.775972659
MTMR9	-0.784706726
PSD3	-0.740690196
FZD3	-0.859522603
ZNF703	-0.755338084
NSD3	-0.746895739
KAT6A	-0.703539381
RNF170	-0.765602921
HOOK3	-0.712627171
PRKDC	-0.770435031
TMEM68	-0.800468367
FAM110B	-0.779600801
CA8	-0.741938672
YTHDF3	-0.796663797
UBE2W	-0.77324408
HEY1	-0.719552663
RUNX1T1	-0.719062152

MTDH	-0.701220718
OSR2	-0.725675749
SLC25A32	-0.760384657
RP11.422N16.3	-0.784866352
FAM83A	-0.773092437
FAM49B	-0.818785978
EFR3A	-0.707976908
FAM135B	-0.757141496
COL22A1	-0.752908001
ZNF623	-0.803403471
GRINA	-0.749124397
ARHGAP39	-0.725848199
SPATA6L	-0.775670893
NFIB	-0.703426089
ZDHHC21	-0.746901552
PSIP1	-0.781460629
DENND4C	-0.712989238
MLLT3	-0.897205509
CDKN2A.AS1	-0.739475101
TUSC1	-0.797263374
CAAP1	-0.78155224
PLAA	-0.781753803
VCP	-0.777667528
C9orf40	-0.750157103
CARNMT1	-0.785065066
VPS13A	-0.748728671
TLE4	-0.734119908
NFIL3	-0.780562774
ZNF484	-0.753493684
ZNF169	-0.740978312
EPB41L4B	-0.770636644
PTBP3	-0.830631585
ZFP37	-0.726449242
ASTN2	-0.758737946
BRINP1	-0.732950539
RAB14	-0.76083786
MRRF	-0.746093574
ZBTB6	-0.769683715
SCAI	-0.704976303
PPP6C	-0.769317628
ZBTB34	-0.705606622
GTF3C4	-0.754406619
SURF4	-0.785226364
BRD3	-0.713034987
WDR5	-0.746962304
RABL6	-0.763861461
DIP2C	-0.733685183

SFMBT2	-0.713012457
SEC61A2	-0.76145745
TRDMT1	-0.722339646
NEBL	-0.831380241
SKIDA1	-0.76191783
OTUD1	-0.759834307
THNSL1	-0.705098862
ABI1	-0.737905244
ANKRD26	-0.779549572
WAC	-0.722228865
CUL2	-0.808708552
ZNF37A	-0.709545735
SLC25A16	-0.728856212
SPOCK2	-0.76983833
AP3M1	-0.761507242
CPEB3	-0.744109702
X44990	-0.825086849
IDE	-0.732715448
EXOC6	-0.778653659
SORBS1	-0.800163515
LCOR	-0.708566899
BTRC	-0.715440484
NOLC1	-0.776048873
DUSP5	-0.702102462
NHLRC2	-0.729744014
FAM160B1	-0.796419538
TRIM66	-0.765551115
TMEM9B	-0.804857128
PDE3B	-0.710714227
ZDHHC13	-0.784636442
NAV2	-0.736424419
LGR4	-0.742508011
LIN7C	-0.728818835
METTL15	-0.802666606
TRAF6	-0.743952628
API5	-0.758234586
CELF1	-0.742169597
OR5B2	-0.726939872
STX3	-0.719651832
NXF1	-0.73581998
C11orf95	-0.720255483
C11orf84	-0.784371459
KMT5B	-0.714574598
TPCN2	-0.727255086
PPFIA1	-0.726324135
NDUFC2	-0.811488732
CCDC89	-0.736214124

AMOTL1	-0.830956902
SRSF8	-0.805014089
DCUN1D5	-0.73375034
TMPRSS4	-0.720143278
CBL	-0.792666728
ARHGEF12	-0.799529805
TMEM225	-0.75728785
EI24	-0.807899861
CACNA1C	-0.777134593
PARP11	-0.760409001
ZNF384	-0.724737337
FOXJ2	-0.715286619
RIMKLB	-0.744054956
LRP6	-0.717312799
FAM234B	-0.747231682
ATF7IP	-0.708924004
AEBP2	-0.863288857
ETNK1	-0.773215838
MED21	-0.827303439
ALG10B	-0.731594701
SENP1	-0.734335706
SLC11A2	-0.700580706
SARNP	-0.70169081
USP15	-0.728212452
PPM1H	-0.725397521
TBC1D30	-0.785295781
CAND1	-0.776648807
CPSF6	-0.723441775
ZDHHC17	-0.789626524
TMTC3	-0.760084442
PLXNC1	-0.786079412
APAF1	-0.773627822
UBE3B	-0.740942203
RNFT2	-0.778158457
KSR2	-0.753492212
SPPL3	-0.776121941
CAMKK2	-0.734663626
KDM2B	-0.747678197
BCL7A	-0.761982312
SBNO1	-0.757737772
TCTN2	-0.913410331
ZNF84	-0.739591276
ZNF268	-0.816332796
WASF3	-0.777225061
POLR1D	-0.724245567
N4BP2L2	-0.737349556
NBEA	-0.761350699

COG6	-0.766704907
KBTBD6	-0.797875344
AKAP11	-0.828249751
INTS6	-0.751769817
DIS3	-0.74347641
KLF12	-0.776912603
FBXL3	-0.758394367
SPRY2	-0.724777787
GPR180	-0.765959634
MBNL2	-0.751994396
ARGLU1	-0.71266953
ANKRD10	-0.71679093
ARHGEF7	-0.744640386
SUPT16H	-0.751411923
SLC22A17	-0.874027144
G2E3	-0.758986814
ARHGAP5	-0.766367989
GEMIN2	-0.748566328
PRPF39	-0.707282606
KLHDC2	-0.768673499
L2HGDH	-0.778505468
GPR137C	-0.738674695
ATG14	-0.726155835
ARID4A	-0.747820341
JKAMP	-0.721634476
RTN1	-0.732347149
PPM1A	-0.766260438
SIX4	-0.750790968
MAX	-0.703163697
MED6	-0.800335249
RBM25	-0.717523363
GPATCH2L	-0.716448069
IRF2BPL	-0.763492306
GTF2A1	-0.801305928
ATXN3	-0.737698807
BTBD7	-0.724824793
YY1	-0.764960322
RCOR1	-0.719071662
MARK3	-0.733218883
NIPA2	-0.816556297
UBE3A	-0.785144444
SLC12A6	-0.769395662
LPCAT4	-0.776909632
RASGRP1	-0.746834244
CASC4	-0.910541296
MYEF2	-0.736602367
USP8	-0.757101195

MYO5A	-0.738081384
RFX7	-0.725851854
RNF111	-0.792359513
ZNF609	-0.713077987
FEM1B	-0.763066322
GLCE	-0.709072452
ARIH1	-0.783669601
NRG4	-0.72754156
PEAK1	-0.777615748
MRPS11	-0.76479373
ARPIN	-0.733525366
LRRC28	-0.772198576
MEF2A	-0.754440339
ASB7	-0.803135957
PDPK1	-0.788072815
TFAP4	-0.729600103
ERCC4	-0.785711874
RRN3	-0.777902231
NOMO3	-0.77654209
ERI2	-0.714055555
EEF2K	-0.716274241
TNRC6A	-0.738987414
SH2B1	-0.737595061
ORAI3	-0.750879657
ZNF720	-0.734094546
SIAH1	-0.756963555
PAPD5	-0.773706392
RPGRIP1L	-0.815616199
PDP2	-0.743056253
SLC7A6	-0.725915731
NFAT5	-0.771257446
AP1G1	-0.743530538
ZFP1	-0.7641004
TERF2IP	-0.842372243
CDYL2	-0.749072432
ZDHHC7	-0.750106751
FBXO31	-0.721053207
CNTROB	-0.72428955
HES7	-0.724949053
MPRIP	-0.748007453
NUFIP2	-0.732265878
DDX52	-0.739425845
MSL1	-0.751224106
KRT37	-0.779508396
P3H4	-0.793224502
KLHL11	-0.78213252
STAT3	-0.765170388

DHX8	-0.764391652
ARL17A	-0.716578068
MBTD1	-0.700917454
COIL	-0.748666595
MSI2	-0.70297123
SRSF1	-0.705129654
GDPD1	-0.800732442
APPBP2	-0.767928662
HN1	-0.786887184
SUMO2	-0.770408088
SRSF2	-0.727314368
WDR45B	-0.71456658
CEP76	-0.735671154
RPRD1A	-0.744878646
CELF4	-0.790669527
SYT4	-0.770796068
C18orf25	-0.786457721
PIAS2	-0.746916617
HDHD2	-0.729425308
KIAA1468	-0.791268881
ZNF236	-0.766666147
PARD6G	-0.745389326
CIRBP	-0.711504632
ZNF554	-0.773764906
ZNF555	-0.716536085
NFIC	-0.708208531
KDM4B	-0.713153595
CD209	-0.702323769
DNMT1	-0.713738394
TIMM29	-0.779251572
ELL	-0.738346082
ZNF14	-0.767455288
ZNF682	-0.757298056
ZNF714	-0.750126905
ZNF492	-0.81052002
ZNF260	-0.707472526
ZNF570	-0.741049213
SUPT5H	-0.758989369
ZNF780B	-0.715194602
ZNF780A	-0.753276929
IGFL4	-0.76503434
U2AF2	-0.742819055
ZNF264	-0.73683363
ZNF460	-0.724183759
ZNF544	-0.766976623
CDS2	-0.766774278
PLCB4	-0.701957125

POLR3F	-0.740618388
SEC23B	-0.770424663
RALGAPA2	-0.717684644
PLAGL2	-0.758868445
C20orf203	-0.785508207
NCOA6	-0.723687995
PHF20	-0.783586486
ZHX3	-0.759527945
CHD6	-0.751661616
ZSWIM1	-0.737793318
ZMYND8	-0.792074689
ARFGEF2	-0.74708462
CSE1L	-0.731276274
SPATA2	-0.702220119
LSM14B	-0.751510041
YTHDF1	-0.751178251
SON	-0.739909691
ITSN1	-0.780578093
DYRK1A	-0.782954864
CRKL	-0.761141852
MAPK1	-0.753356599
ZNRF3	-0.760449869
PRR14L	-0.748550708
RBFOX2	-0.742136386
JOSD1	-0.739315975
TNRC6B	-0.748716618
EP300	-0.721232154
PPP6R2	-0.731117802
RABL2B	-0.792235669
ASB11	-0.725995771
REPS2	-0.731115252
NHS	-0.753783891
SCML2	-0.726632044
PDHA1	-0.750664298
MAP7D2	-0.767507734
ZFX	-0.711995642
MAGEB4	-0.787792537
USP9X	-0.76550915
DDX3X	-0.777285312
CASK	-0.800210363
PHF8	-0.721098301
ZC3H12B	-0.807071749
ZMYM3	-0.811949389
RGAG4	-0.747345089
KIAA2022	-0.839122142
ABCB7	-0.731768874
ATRX	-0.769885182

BRWD3	-0.703708665
ARMCX5.GPRASP2	-0.807993872
GPRASP2	-0.825696045
SLC25A53	-0.724345468
MID2	-0.847689097
XIAP	-0.730898845
BCORL1	-0.743040351
FAM122B	-0.803099644
RBMX	-0.709428926
AFF2	-0.700291554
HCFC1	-0.738740195
RAB39B	-0.783949283
USP9Y	-0.749001142
RTEL1	-0.732543237
RAF1	-0.718326278
MYO15A	-0.762008666
TTN	-0.767283145

hsa-miR-5689

**Target gene**	**Correlation value**
VAMP3	-0.715633532
PKP4	-0.806456169
GBE1	-0.734744337
NT5DC1	-0.761583065
GNAI1	-0.725589668
MATN2	-0.764029938
TRPM6	-0.77052827

hsa-miR-3529-3p

Gene ontology (GO)	Term	P-value	Adjusted P-value	Old P-value	Old Adjusted P-value	Odds Ratio	Combined Score	Genes
Biological Process	Regulation Of DNA-templated Transcription (GO:0006355)	8.16E-13	2.16E-09	0	0	2.150272397	59.85041472	*ATF2;CCNT2;ZNF292;ARID4A;PRDM2;ARID4B;YY1;ZNF609;GPBP1;EP300;BTRC;ZFP37;RNF111;SOX4;HES7;ZNF682;OSR2;RBFOX2;KDM2B;FBXW11;ADIPOQ;DYRK1A;ZNF14;MED6;PIAS2;HCFC1;RFX7;TBL1XR1;KAT6A;ZNF714;PHIP;CLOCK;KHDRBS1;INO80D;MAX;BCL10;MTDH;MBTD1;SBNO1;ATXN1;TTC21B;DHX36;ZNF703;HIVEP1;HIVEP3;PPARGC1A;ZNF268;DR1;POU2F1;PBX2P1;EGR4;TFAP2E;BRPF3;IRF2BP2;SMAD5;ZNF37A;SP4;JMY;PHF14;RBAK;RCOR1;BRWD3;SETD5;LIN54;GMEB1;ZNF492;PHF20;HP1BP3;PHF8;ING2;ING3;HEY1;BCL7A;SIX4;WDR5;ELOA;BRD3;PBRM1;KLF12;ZHX3;NCOA6;DNMT3A;ATRX;MSL2;ETV1;BAZ1B;ARID1A;ZFX;MSL1;PPM1A;CREB1;AEBP2;MED21;TRAF6;WAC;LCOR;TERF2IP;ZNF780B;ZNF236;SOS1;VGLL4;KDM7A;USP13;NFAT5;PRKAA1;FOXK1;LIN9;MLLT3;NR2C2;LRP6;CAMKK2;NSD3;RBBP4;NFIL3;NSD2;PLAGL2;SRSF10;RUNX1T1;ATF7IP;ZNF460;STAT3;FOXJ2;TFAP4;NFIB;POLR3C;NFIC;ZNF736;POLR3F;CTNNB1;NAA15*
Biological Process	Positive Regulation Of DNA-templated Transcription (GO:0045893)	3.69E-09	4.89E-06	0	0	2.124740641	41.25549242	*ATF2;CCNT2;DDX3X;ELL;PHF20;CHD6;HNRNPU;PRDM2;MYSM1;PHF8;IRF2BPL;YY1;ING2;ZNF609;ING3;HEY1;SIX4;WDR5;EP300;BTRC;RNF111;SOX4;GTF2I;NCK1;MEF2A;OSR2;SUPT16H;NCOA6;FBXW11;ATRX;DYRK1A;MSL2;ETV1;BAZ1B;ARID1A;ZFX;MED6;HCFC1;MSL1;SENP1;PPM1A;CREB1;TBL1XR1;MED21;KAT6A;TRAF6;WAC;PHIP;CLOCK;GTF2A1;NFAT5;PRKAA1;INO80D;PRKDC;MAX;FOXK1;BCL10;MLLT3;NR2C2;LRP6;CAMKK2;NSD3;MBTD1;RBBP4;NFIL3;DHX36;HIVEP1;HIVEP3;PPARGC1A;ZNF268;MEF2D;ATF7IP;DR1;POU2F1;PBX2P1;EGR4;STAT3;FOXJ2;SUPT5H;SMAD5;TFAP4;NFIB;NFIC;CTNNB1;NAA15;RBMX*
Biological Process	Negative Regulation Of DNA-templated Transcription (GO:0045892)	1.94E-08	1.38E-05	0	0	2.172184484	38.57695224	*ATF2;ZMYND8;HNRNPU;ARID4A;PRDM2;ARID4B;N4BP2L2;IRF2BPL;YY1;ING2;HEY1;BCL7A;SIX4;EP300;BTRC;ZNF169;ZFP37;SOX4;MEF2A;OSR2;KLF12;RBFOX2;ZHX3;KDM2B;FBXW11;ADIPOQ;DNMT3A;ZBTB34;HCFC1;PPM1A;CREB1;TBL1XR1;KAT6A;TRAF6;LCOR;ZNF555;BCORL1;CLOCK;ZNF554;CGGBP1;MAGEB4;VGLL4;KHDRBS1;MAX;DDX20;FOXK1;NR2C2;MTDH;ATXN1;RBBP4;NFIL3;MIER3;ZNF703;HIVEP1;FNIP1;ZNF268;RUNX1T1;ATF7IP;DR1;POU2F1;USP9X;STAT3;IRF2BP2;SUPT5H;ZBTB6;TFAP4;NFIB;NFIC;PHF14;CTNNB1;CPEB3;RBAK;RCOR1*
Biological Process	Regulation Of Gene Expression (GO:0010468)	2.09E-08	1.38E-05	0	0	2.111516977	37.33830695	*ATF2;LIN54;GSK3B;DDX3X;ZNF492;PHF20;CELF1;HP1BP3;CHD6;PRDM2;PIK3CB;AFF3;AFF4;CRKL;AFF2;YY1;EPB41L4B;ING2;ING3;GPBP1;WDR5;ZFP37;SOX4;ZNF682;OSR2;MBNL2;SFMBT2;ATRX;MSL2;ZBTB34;ATG14;HCFC1;PTBP3;MSL1;KAT6A;ZNF638;ZNF714;TERF2IP;CLOCK;CGGBP1;USP13;DNMT1;PRKAA1;PRKAA2;SRSF1;TMPRSS4;RNF8;HNRNPLL;NR2C2;TTN;NSD3;SBNO1;RBBP4;NFIL3;NSD2;DHX36;ZNF703;STX3;PPARGC1A;ZNF268;SRSF10;ATF7IP;DR1;ZNF460;SETDB1;BRPF3;STAT3;ZNF804A;PUM1;SMAD5;PUM2;SON;ZBTB6;ZNF37A;ZNF736;SPRY2;RBAK;TARDBP*
Biological Process	Chromatin Remodeling (GO:0006338)	5.96E-08	2.68E-05	0	0	3.698197325	61.52188076	*LIN54;KDM5B;NFAT5;USP15;INO80D;HP1BP3;CHD6;RNF8;PSIP1;MYSM1;YY1;BCL7A;RBBP4;SMARCAD1;JARID2;PBRM1;DR1;KDM4B;SETDB1;KDM2B;ATRX;BAZ1B;ARID1A;HCFC1;WAC;TRIP12;CGGBP1*
Biological Process	Positive Regulation Of Nucleic Acid-Templated Transcription (GO:1903508)	6.07E-08	2.68E-05	0	0	2.57025901	42.71235754	*PRKAA1;INO80D;PHF20;MAX;FOXK1;BCL10;MLLT3;PHF8;LRP6;CAMKK2;NSD3;YY1;MBTD1;ING2;ING3;RBBP4;NFIL3;SIX4;WDR5;EP300;HIVEP3;BTRC;PPARGC1A;RNF111;SOX4;ATF7IP;OSR2;NCOA6;FBXW11;STAT3;DYRK1A;MSL2;ARID1A;SMAD5;HCFC1;MSL1;PPM1A;TFAP4;CREB1;NFIB;TBL1XR1;KAT6A;WAC;CTNNB1;PHIP;NAA15;CLOCK*
Biological Process	Regulation Of Transcription By RNA Polymerase II (GO:0006357)	8.91E-08	3.37E-05	0	0	1.782592058	28.93779312	*ATF2;CCNT2;ZNF292;ZMYND8;HNRNPU;ARID4A;PRDM2;ARID4B;YY1;ZNF609;EP300;ZNF169;ZFP37;SOX4;GTF2I;MEF2A;HES7;OSR2;KDM2B;ZBTB34;ZNF14;MED6;PIAS2;HCFC1;RFX7;TBL1XR1;PHIP;ZNF555;BCORL1;CLOCK;ZNF554;CGGBP1;MAGEB4;PRKDC;MAX;MTDH;ATXN1;MIER3;TTC21B;DHX36;HIVEP1;HIVEP3;PPARGC1A;ZNF268;DR1;POU2F1;PBX2P1;EGR4;TFAP2E;BRPF3;IRF2BP2;SUPT5H;SMAD5;ZBTB6;SP4;JMY;PHF14;CPEB3;RBAK;RCOR1;BRWD3;GMEB1;DDX3X;PHF20;CHD6;N4BP2L2;MYSM1;PHF8;IRF2BPL;ING2;HEY1;BCL7A;SIX4;WDR5;ELOA;NCK1;BRD3;PBRM1;KLF12;ZHX3;NCOA6;DNMT3A;ATRX;ETV1;BAZ1B;ARID1A;ZFX;SENP1;PPM1A;CREB1;AEBP2;MED21;TRAF6;LCOR;ZNF780B;ZNF236;SOS1;KDM7A;GTF2A1;NFAT5;DDX20;FOXK1;LIN9;NR2C2;LRP6;RBBP4;NFIL3;PLAGL2;FNIP1;MEF2D;USP9X;STAT3;FOXJ2;TFAP4;NFIB;NFIC;CTNNB1;RBMX*
Biological Process	Regulation Of Nucleic Acid-Templated Transcription (GO:1903506)	3.20E-06	9.65E-04	0	0	2.462576514	31.16095952	*USP13;ATF2;LIN54;ZNF492;HP1BP3;PRDM2;NR2C2;NSD3;SBNO1;ING2;ING3;RBBP4;NFIL3;GPBP1;NSD2;ZNF703;WDR5;PPARGC1A;ZNF268;SRSF10;ZFP37;SOX4;ATF7IP;ZNF682;DR1;ZNF460;BRPF3;STAT3;ATRX;HCFC1;ZNF37A;KAT6A;ZNF736;ZNF714;TERF2IP;RBAK;CLOCK*
Biological Process	Regulation Of Chromatin Organization (GO:1902275)	3.28E-06	9.65E-04	0	0	11.3903016	143.8327365	*SETD5;PHF20;WDR5;MSL2;HNRNPU;MLLT3;HCFC1;MSL1*
Biological Process	Ubiquitin-Dependent Protein Catabolic Process (GO:0006511)	4.01E-06	0.0010629	0	0	2.630336689	32.68475855	*GSK3B;VCP;UBE2D4;CUL3;RNF8;UBE3B;CBL;ATXN3;HERC3;NUB1;C18ORF25;ZNRF3;RNF217;FEM1C;ARIH1;BTRC;RNF111;KLHDC2;USP9X;FBXW11;SMURF1;FEM1B;SIAH1;UBE2W;KBTBD6;APPBP2;RNF149;TBL1XR1;CTNNB1;PLAA;TRIP12;CLOCK*
Biological Process	Regulation Of mRNA Splicing, Via Spliceosome (GO:0048024)	6.56E-06	0.0015806	0	0	4.759206799	56.79639826	*KHDRBS1;RBM25;RBFOX2;WTAP;CELF1;CELF4;CIRBP;HNRNPU;SON;U2AF2;TRA2A;SRSF10;RBMX;HNRNPA0*
Biological Process	mRNA Metabolic Process (GO:0016071)	1.04E-05	0.0022902	0	0	4.905390539	56.29489799	*CPSF6;SSB;CELF1;GEMIN2;SRSF1;SON;AGO3;U2AF2;HNRNPA2B1;SRSF2;PPARGC1A;DCP2;HNRNPA0*
Biological Process	Negative Regulation Of Transcription By RNA Polymerase II (GO:0000122)	1.70E-05	0.0034674	0	0	1.988058649	21.83152949	*ATF2;ZMYND8;HNRNPU;ARID4A;PRDM2;ARID4B;N4BP2L2;IRF2BPL;YY1;ING2;HEY1;EP300;ZNF169;ZFP37;SOX4;MEF2A;OSR2;KLF12;KDM2B;DNMT3A;ZBTB34;HCFC1;PPM1A;TBL1XR1;TRAF6;LCOR;ZNF555;BCORL1;ZNF554;CGGBP1;MAGEB4;MAX;DDX20;NR2C2;MTDH;ATXN1;RBBP4;NFIL3;MIER3;HIVEP1;FNIP1;ZNF268;DR1;USP9X;STAT3;IRF2BP2;SUPT5H;ZBTB6;NFIC;PHF14;CPEB3*
Biological Process	Positive Regulation Of Transcription By RNA Polymerase II (GO:0045944)	4.32E-05	0.0077223	0	0	1.831914309	18.41000448	*ATF2;CCNT2;DDX3X;ELL;ZMYND8;CHD6;HNRNPU;PRDM2;MYSM1;PHF8;IRF2BPL;YY1;ZNF609;HEY1;EP300;SOX4;GTF2I;NCK1;MEF2A;OSR2;NCOA6;ATRX;ETV1;BAZ1B;ZFX;MED6;HCFC1;MED28;SENP1;CREB1;TBL1XR1;MED21;TRAF6;PHIP;GTF2A1;NFAT5;PRKDC;MAX;NR2C2;LRP6;DHX36;HIVEP1;PPARGC1A;ZNF268;MEF2D;DR1;POU2F1;PBX2P1;EGR4;STAT3;FOXJ2;SUPT5H;SMAD5;NFIB;NFIC;TAF8;CTNNB1;RBMX*

Biological Process	Regulation Of Multicellular Organismal Development (GO:2000026)	4.37E-05	0.0077223	0	0	5.527737856	55.48484182	*YY1;DR1;INO80D;RAB14;BRPF3;KAT6A;DHX36;WDR5;TRIP12;MYSM1*
Biological Process	Regulation Of Embryonic Development (GO:0045995)	6.79E-05	0.0112427	0	0	5.208017335	49.98336222	*YY1;DR1;POGLUT1;INO80D;RAB14;DHX36;WDR5;TRIP12;NR2C2;CLASP2*
Biological Process	Positive Regulation Of Telomere Maintenance (GO:0032206)	8.87E-05	0.0138223	0	0	5.662937886	52.83633731	*YY1;RTEL1;INO80D;ATRX;DHX36;HNRNPA2B1;TERF2IP;MAPK1;MAP3K4*
Biological Process	Protein Ubiquitination (GO:0016567)	1.10E-04	0.0161874	0	0	2.184195303	19.90913444	*VCP;UBE2D4;CUL3;CUL2;FBXO25;RNF8;UBE3A;ASB11;UBE3B;CBL;NUB1;HERC3;TRIM7;ZNRF3;CAND1;RNF217;ARIH1;BTRC;RNF111;TRIM23;RNF44;USP9X;FBXW11;SMURF1;MSL2;LNPEP;UBE2W;TRAF6;CTNNB1;FBXL3;TRIP12;FBXL5*
Biological Process	mRNA Destabilization (GO:0061157)	1.29E-04	0.0173531	0	0	6.178170144	55.34156693	*YTHDF1;YTHDF3;AGO3;CELF1;DHX36;PUM1;CPEB3;TARDBP*
Biological Process	Positive Regulation Of Translation (GO:0045727)	1.36E-04	0.0173531	0	0	3.713317948	33.07106774	*KHDRBS1;YTHDF1;DDX3X;MRPS27;SSB;YTHDF3;PRKDC;UQCC2;CIRBP;HNRNPU;DHX36;CPEB3;SOX4*
Biological Process	Proteasomal Protein Catabolic Process (GO:0010498)	1.44E-04	0.0173531	0	0	2.725714286	24.11570506	*KLHDC2;GSK3B;VCP;FBXW11;SMURF1;FEM1B;CUL3;SIAH1;ATXN3;UBE2W;KBTBD6;APPBP2;TBL1XR1;PSME4;CTNNB1;PLAA;TRIP12;FEM1C;BTRC;CLOCK*
Biological Process	Protein Modification By Small Protein Conjugation (GO:0032446)	1.44E-04	0.0173531	0	0	2.281310212	20.17791896	*VCP;DCUN1D5;UBE2D4;CUL3;CUL2;FBXO25;ASB11;SMC6;CBL;HERC3;NUB1;TRIM7;CAND1;ZNRF3;SUMO2;ARIH1;BTRC;RNF111;TRIM23;RNF44;USP9X;FBXW11;SMURF1;MSL2;PIAS2;SENP1;FBXL3;FBXL5*
Biological Process	Regulation Of Transforming Growth Factor Beta Receptor Signaling Pathway (GO:0017015)	1.79E-04	0.0192421	0	0	3.599082066	31.05030259	*SMURF1;PDPK1;ARID4A;ARID4B;NREP;PPM1A;ING2;RBBP4;ZNF703;EP300;SPRY2;SPRY1;RNF111*
Biological Process	Positive Regulation Of mRNA Catabolic Process (GO:0061014)	1.80E-04	0.0192421	0	0	5.843607653	50.39865838	*YTHDF1;YTHDF3;CELF1;DHX36;PUM1;CPEB3;TNRC6A;TNRC6B*
Biological Process	Insulin Metabolic Process (GO:1901142)	1.82E-04	0.0192421	0	0	21.53631285	185.5076579	*ERO1B;SLC30A5;YIPF5;IDE*
Biological Process	Modification-Dependent Protein Catabolic Process (GO:0019941)	2.08E-04	0.0211722	0	0	2.815502505	23.87238315	*UBE2D4;SMURF1;CUL3;SIAH1;RNF8;UBE3B;CBL;ATXN3;HERC3;NUB1;C18ORF25;ZNRF3;RNF149;RNF217;FEM1C;TRIP12;ARIH1;BTRC*
Biological Process	Cellular Response To Insulin Stimulus (GO:0032869)	2.27E-04	0.0219802	0	0	3.739138905	31.36625657	*GSK3B;FER;PRKDC;PDPK1;DENND4C;PDE3B;MYO5A;ZDHHC7;PHIP;IDE;SORBS1;APPL1*
Biological Process	Proteasome-Mediated Ubiquitin-Dependent Protein Catabolic Process (GO:0043161)	2.42E-04	0.0219802	0	0	2.32295796	19.34012335	*GSK3B;VCP;CUL3;CUL2;ATXN3;FBXO6;FEM1C;BTRC;KLHDC2;FBXW11;SMURF1;FEM1B;SIAH1;FBXO31;UBE2W;KBTBD6;APPBP2;TBL1XR1;JKAMP;CTNNB1;PLAA;FBXL3;TRIP12;FBXL5;CLOCK*
Biological Process	Positive Regulation Of RNA Splicing (GO:0033120)	2.55E-04	0.0219802	0	0	6.517241379	53.91904411	*U2AF2;TRA2A;CELF4;CIRBP;DYRK1A;HNRNPLL;RBMX*
Biological Process	Regulation Of Tubulin Deacetylation (GO:0090043)	2.57E-04	0.0219802	0	0	11.22843823	92.80936091	*DR1;PRKAA1;PRKAA2;WDR5;EP300*
Biological Process	Positive Regulation Of Peptidyl-Lysine Acetylation (GO:2000758)	2.57E-04	0.0219802	0	0	11.22843823	92.80936091	*PRKAA1;PRKAA2;BAZ1B;PPARGC1A;SOX4*
Biological Process	Positive Regulation Of Protein Metabolic Process (GO:0051247)	2.68E-04	0.0222015	0	0	2.751656583	22.62906172	*USP8;GSK3B;YTHDF1;VCP;DDX3X;SSB;YTHDF3;PRKDC;FBXW11;CIRBP;ASB11;BCL10;IDE;RASGRP1;BTRC;CPEB3;SOX4;VGLL4*
Biological Process	mRNA Processing (GO:0006397)	2.83E-04	0.0226905	0	0	2.652730531	21.67610015	*CPSF6;DHX8;CELF1;GEMIN2;SRSF1;HNRNPU;RPRD2;CWC27;SON;U2AF2;HNRNPA2B1;TRA2A;SRSF2;RPRD1A;PPARGC1A;SRSF10;RBMX;SRSF8;HNRNPA0*
Biological Process	Regulation Of Transcription Elongation By RNA Polymerase II (GO:0034243)	4.27E-04	0.0323796	0	0	4.038890057	31.3372637	*ELL;ZMYND8;MED21;RNF8;HNRNPU;INTS7;INTS6;SUPT5H;MED6;MED28*
Biological Process	Histone H4 Acetylation (GO:0043967)	4.28E-04	0.0323796	0	0	5.905329593	45.80035137	*MBTD1;ING3;PHF20;WDR5;MSL2;HCFC1;MSL1*
Biological Process	mRNA 5'-Splice Site Recognition (GO:0000395)	4.40E-04	0.0323796	0	0	40.33054393	311.7004312	*PRPF39;SRSF1;PSIP1*
Biological Process	Regulation Of Alternative mRNA Splicing, Via Spliceosome (GO:0000381)	5.74E-04	0.0410619	0	0	4.802746567	35.84623697	*KHDRBS1;RBM25;RBFOX2;WTAP;CELF4;HNRNPU;RBMX;HNRNPA0*
Biological Process	Regulation Of Dosage Compensation By Inactivation Of X Chromosome (GO:1900095)	6.54E-04	0.0422726	0	0	13.45810056	98.67453255	*PHF20;MSL2;WDR5;MSL1*
Biological Process	Regulation Of Leukocyte Adhesion To Vascular Endothelial Cell (GO:1904994)	6.54E-04	0.0422726	0	0	13.45810056	98.67453255	*NFAT5;FUT9;TRAF6;ZDHHC21*
Biological Process	Histone H4-K16 Acetylation (GO:0043984)	6.54E-04	0.0422726	0	0	13.45810056	98.67453255	*PHF20;MSL2;WDR5;MSL1*

Biological Process	Positive Regulation Of Histone H3-K4 Methylation (GO:0051571)	6.54E-04	0.0422726	0	0	13.45810056	98.67453255	*PHF20;WDR5;CTNNB1;HCFC1*
Biological Process	Chromatin Organization (GO:0006325)	7.41E-04	0.0467169	0	0	2.315007559	16.68638137	*KDM5B;PBRM1;SUPT16H;DR1;KDM4B;INO80D;KDM2B;ATRX;CHD6;HNRNPU;PSIP1;BAZ1B;ARID1A;MYSM1;HCFC1;YY1;BCL7A;RBBP4;WAC;SMARCAD1;JARID2*
Biological Process	SCF-dependent Proteasomal Ubiquitin-Dependent Protein Catabolic Process (GO:0031146)	7.92E-04	0.0488007	0	0	5.248091008	37.47529951	*FBXW11;CUL2;FBXO6;FBXL3;BTRC;FBXL5;FBXO31*
Biological Process	Positive Regulation Of DNA-templated Transcription, Elongation (GO:0032786)	8.36E-04	0.0503434	0	0	4.501872659	31.90312754	*SUPT16H;CCNT2;ELL;ZMYND8;MED21;SUPT5H;MED6;MED28*
Biological Process	Regulation Of DNA-templated Transcription Elongation (GO:0032784)	9.13E-04	0.0537561	0	0	5.105985368	35.73461214	*SETD5;SUPT16H;CCNT2;ELL;INTS7;INTS6;SUPT5H*
Biological Process	RNA Processing (GO:0006396)	9.80E-04	0.0564456	0	0	2.60111595	18.01994422	*CPSF6;DHX8;CELF1;DIS3;GEMIN2;SRSF1;DDX20;HNRNPU;ATXN1;SON;U2AF2;HNRNPA2B1;SRSF2;TARDBP;PPARGC1A;HNRNPA0*
Biological Process	Protein K63-linked Deubiquitination (GO:0070536)	0.0010281	0.0579435	0	0	5.99221911	41.22693111	*USP13;ATXN3;USP8;USP9X;SPATA2;OTUD1*
Biological Process	Brain Development (GO:0007420)	0.0011978	0.064882	0	0	2.642442664	17.77651227	*KDM4B;RTN1;PBX2P1;FBXW11;NCOA6;UBE3A;AFF2;PHF8;ATXN1;RELN;RBBP4;NFIB;TBC1D23;CNTN4;ADGRL3*
Biological Process	Regulation Of Histone H3-K4 Methylation (GO:0051569)	0.0012491	0.064882	0	0	10.76536313	71.96965195	*PHF20;WDR5;CTNNB1;HCFC1*
Biological Process	Histone mRNA Catabolic Process (GO:0071044)	0.0012491	0.064882	0	0	10.76536313	71.96965195	*SSB;XRN1;ERI1;DCP2*
Biological Process	miRNA-mediated Gene Silencing By Inhibition Of Translation (GO:0035278)	0.0012491	0.064882	0	0	10.76536313	71.96965195	*AGO3;EIF4E2;TNRC6A;TNRC6B*
Biological Process	Regulation Of mRNA Stability (GO:0043488)	0.00132	0.0672435	0	0	3.741966894	24.80972405	*YTHDF1;YTHDF3;CELF1;DHX36;CIRBP;HNRNPU;PUM1;DCP2;PUM2*
Biological Process	Positive Regulation Of Telomere Maintenance Via Telomere Lengthening (GO:1904358)	0.0014175	0.0708469	0	0	5.578383077	36.58794248	*RTEL1;DHX36;HNRNPA2B1;MAPK1;CTNNB1;MAP3K4*
Biological Process	Negative Regulation Of Translation (GO:0017148)	0.001642	0.0790837	0	0	3.100376117	19.87915405	*YTHDF1;DDX3X;AGO3;YTHDF3;CELF1;DHX36;PUM1;CPEB3;EIF4E2;TNRC6A;TNRC6B*
Biological Process	Positive Regulation Of Autophagy (GO:0010508)	0.001642	0.0790837	0	0	3.100376117	19.87915405	*GSK3B;PRKAA1;PRKAA2;WAC;GNAI3;PIK3CB;SUPT5H;MID2;TRIM23;MTDH;CAMKK2*
Biological Process	Regulation Of Cell Differentiation (GO:0045595)	0.0017111	0.0809412	0	0	2.45287622	15.62633651	*GSK3B;PBRM1;UQCC2;NREP;ARID1A;RELN;HEY1;BCL7A;RBBP4;SPOCK2;ST7;TAF8;CTNNB1;RAF1;ZNF268;CLASP2*
Biological Process	Negative Regulation Of Transforming Growth Factor Beta Receptor Signaling Pathway (GO:0030512)	0.0017516	0.0814012	0	0	3.576321668	22.69981439	*PPM1A;ING2;RBBP4;PDPK1;SMURF1;ARID4A;SPRY2;ARID4B;SPRY1*
Biological Process	Protein Acylation (GO:0043543)	0.0019102	0.0861224	0	0	5.217945243	32.66718191	*KAT6A;EP300;ZDHHC13;ZDHHC21;ZDHHC7;ZDHHC17*
Biological Process	Negative Regulation Of Protein Modification Process (GO:0031400)	0.0019182	0.0861224	0	0	3.524307467	22.04942872	*GSK3B;PRKAA1;PRKAA2;PRKDC;TERF2IP;ATG14;TARDBP;CRKL;LRP6*
Biological Process	Positive Regulation Of Developmental Process (GO:0051094)	0.0020098	0.088731	0	0	2.273703041	14.1191012	*GSK3B;PBRM1;PRKAA1;ARID4A;ARID4B;ARID1A;NR2C2;ING2;RELN;BCL7A;RBBP4;ZNF703;CTNNB1;KMT5B;CPEB3;ARHGEF7;ZNF268;CLASP2*
Biological Process	Regulation Of Transcription By RNA Polymerase III (GO:0006359)	0.0020908	0.0893293	0	0	6.413253413	39.57123761	*ELL;POLR3C;DHX36;POLR3F;BAZ1B*
Biological Process	miRNA-mediated Gene Silencing (GO:0035195)	0.0020908	0.0893293	0	0	6.413253413	39.57123761	*CNOT6;AGO3;EIF4E2;TNRC6A;TNRC6B*
Biological Process	RNA 3'-End Processing (GO:0031123)	0.0022004	0.0910757	0	0	5.054621849	30.92983175	*CPSF6;SSB;INTS7;RPRD1A;RPRD2;INTS6*
Biological Process	Regulation Of Telomere Maintenance (GO:0032204)	0.0022004	0.0910757	0	0	5.054621849	30.92983175	*RTEL1;ERCC4;DHX36;ATRX;TERF2IP;SMC6*
Biological Process	RNA Secondary Structure Unwinding (GO:0010501)	0.0022718	0.0911807	0	0	16.12970711	98.18467526	*DDX3X;AGO3;DHX36*
Biological Process	Ubiquitin-Dependent Protein Catabolic Process Via The C-end Degron Rule Pathway (GO:0140627)	0.0022718	0.0911807	0	0	16.12970711	98.18467526	*KLHDC2;FEM1B;FEM1C*
Biological Process	Regulation Of Cell Development (GO:0060284)	0.0024877	0.0983554	0	0	4.196509272	25.16400292	*HES7;HEY1;BRPF3;KAT6A;CTNNB1;MYSM1;CLOCK*

Biological Process	Regulation Of Telomere Maintenance Via Telomere Lengthening (GO:1904356)	0.0027253	0.1061652	0	0	8.279759347	48.893545	*RTEL1;ERCC4;DHX36;HNRNPA2B1*
Biological Process	Positive Regulation Of Stem Cell Population Maintenance (GO:1902459)	0.002879	0.1096294	0	0	4.756796836	27.82879702	*ING2;BCL7A;RBBP4;ARID4A;ARID4B;ARID1A*
Biological Process	Regulation Of Autophagy (GO:0010506)	0.002897	0.1096294	0	0	2.191215362	12.80566471	*USP13;GSK3B;PRKAA1;PRKAA2;STAT3;MTMR9;RAB39B;FOXK1;PIK3CB;ATG14;MID2;TPCN2;MTDH;CASP3;RRAGD;WAC;EP300;TRIM23*
Biological Process	Positive Regulation Of mRNA Processing (GO:0050685)	0.0029438	0.1098336	0	0	5.854971116	34.12304084	*DHX36;TRA2A;CELF4;CIRBP;RBMX*
Biological Process	protein-RNA Complex Assembly (GO:0022618)	0.003052	0.1122897	0	0	2.569291444	14.88119306	*CPSF6;PRKDC;CELF1;GEMIN2;MRPS11;SRSF1;CELF4;DDX20;RPF2;COIL;AGO3;SRSF10;MDN1*
Biological Process	ERBB Signaling Pathway (GO:0038127)	0.0030957	0.1123345	0	0	4.017516636	23.21221645	*EPGN;PDPK1;REPS2;MAPK1;SOS1;CBL;FAM83A*
Biological Process	Central Nervous System Development (GO:0007417)	0.0033106	0.1148802	0	0	2.065942423	11.79783367	*KDM4B;RTN1;PBX2P1;NCOA6;STAT3;UBE3A;MSI2;PHF8;AFF2;MARCKS;RELN;ATXN1;RBBP4;NFIB;SPOCK1;TBC1D23;CNTN4;JARID2;SOX4;ADGRL3*
Biological Process	Positive Regulation Of RIG-I Signaling Pathway (GO:1900246)	0.0033169	0.1148802	0	0	13.44072524	76.72954597	*USP15;PUM1;PUM2*
Biological Process	Positive Regulation Of Protein Acetylation (GO:1901985)	0.0033169	0.1148802	0	0	13.44072524	76.72954597	*PRKAA1;DDX3X;PRKAA2*
Biological Process	Regulation Of Protein Deacetylation (GO:0090311)	0.0034052	0.1148802	0	0	7.687948923	43.68649246	*DR1;DYRK1A;WDR5;EP300*
Biological Process	ncRNA-mediated Post-Transcriptional Gene Silencing (GO:0035194)	0.0034052	0.1148802	0	0	7.687948923	43.68649246	*CNOT6;CELF1;TNRC6A;TNRC6B*
Biological Process	mRNA Splice Site Recognition (GO:0006376)	0.0034533	0.1148802	0	0	5.610722611	31.80393792	*PRPF39;SRSF1;CELF4;PSIP1;SRSF10*
Biological Process	Positive Regulation Of Amide Metabolic Process (GO:0034250)	0.0034694	0.1148802	0	0	3.19853431	18.1157832	*YTHDF1;DDX3X;SSB;YTHDF3;PRKDC;CASP3;CIRBP;CPEB3;SOX4*
Biological Process	Regulation Of RNA Splicing (GO:0043484)	0.0037206	0.1216784	0	0	2.937538273	16.43218326	*MBNL2;SON;CELF1;U2AF2;ZNF638;SRSF1;DYRK1A;HNRNPLL;SRSF10;PTBP3*
Biological Process	Transcription By RNA Polymerase II (GO:0006366)	0.0041291	0.1276329	0	0	2.296309653	12.60601667	*GTF2A1;NFAT5;SUPT16H;PBRM1;CCNT2;ELL;ETV1;SUPT5H;CREB1;NFIC;TAF8;ELOA;RPRD1A;PPARGC1A;RBMX*
Biological Process	Intracellular Glucose Homeostasis (GO:0001678)	0.004175	0.1276329	0	0	4.370429253	23.94406106	*PRKAA1;PRKAA2;FOXK1;ZNF236;PPARGC1A;SOX4*
Biological Process	RNA Destabilization (GO:0050779)	0.0041918	0.1276329	0	0	7.175046555	39.28069914	*RBM27;YTHDF1;YTHDF3;PUM1*
Biological Process	Cellular Response To Misfolded Protein (GO:0071218)	0.0041918	0.1276329	0	0	7.175046555	39.28069914	*ATXN3;UBE2W;VCP;CUL3*
Biological Process	Negative Regulation Of RNA Splicing (GO:0033119)	0.0041918	0.1276329	0	0	7.175046555	39.28069914	*U2AF2;RBMX;SRSF10;PTBP3*
Biological Process	Positive Regulation Of Histone Methylation (GO:0031062)	0.0041918	0.1276329	0	0	7.175046555	39.28069914	*PHF20;WDR5;CTNNB1;HCFC1*
Biological Process	Regulation Of Double-Strand Break Repair (GO:2000779)	0.0043789	0.131814	0	0	3.07659029	16.70885034	*MBTD1;PBRM1;RTEL1;ING3;BCL7A;TERF2IP;TRIP12;PPP4R3B;ARID1A*
Biological Process	Mitotic G1/S Transition Checkpoint Signaling (GO:0044819)	0.0046124	0.1355689	0	0	11.52002391	61.9663821	*PRKDC;WAC;FBXO31*
Biological Process	Peptidyl-Threonine Phosphorylation (GO:0018107)	0.0046455	0.1355689	0	0	3.701647279	19.88471444	*ATF2;GSK3B;PDPK1;PRKDC;DYRK1A;MAPK1;HIPK1*
Biological Process	Negative Regulation Of DNA Biosynthetic Process (GO:2000279)	0.0046571	0.1355689	0	0	5.17859064	27.80568405	*XRN1;ERCC4;ADIPOQ;HNRNPU;DCP2*
Biological Process	Positive Regulation Of Gene Expression (GO:0010628)	0.0047889	0.1378901	0	0	1.752147503	9.359003284	*ATF2;GSK3B;DNMT1;PRKAA1;DDX3X;PRKDC;CELF1;PIK3CB;HNRNPLL;TTN;EPB41L4B;U2AF2;DHX36;WDR5;EP300;PPARGC1A;SOX4;OSR2;YTHDF1;SSB;YTHDF3;STAT3;DYRK1A;CIRBP;ZNF804A;ARID1A;HCFC1;SPRY2;CPEB3*
Biological Process	Positive Regulation Of Proteasomal Protein Catabolic Process (GO:1901800)	0.0050816	0.1447435	0	0	3.000312549	15.84804101	*USP13;GSK3B;ATXN3;NUB1;VCP;SUMO2;RNF217;TMTC3;ARIH1*
Biological Process	Epidermal Growth Factor Receptor Signaling Pathway (GO:0007173)	0.0052502	0.1478195	0	0	4.145873734	21.76369677	*EPGN;PDPK1;REPS2;CBL;SOS1;FAM83A*
Biological Process	Negative Regulation Of Gene Expression (GO:0010629)	0.0053012	0.1478195	0	0	1.903765079	9.975393503	*GSK3B;DNMT1;PRKAA1;DDX3X;PRKAA2;SETDB1;CELF1;SFMBT2;STAT3;PUM1;SMAD5;CRKL;PTBP3;YY1;AGO3;DHX36;CPEB3;TARDBP;EIF4E2;DCP2;LGR4;TNRC6B*
Biological Process	miRNA Processing (GO:0035196)	0.005359	0.1478743	0	0	4.986531987	26.07448531	*PUS10;AGO3;HNRNPA2B1;PUM1;PUM2*
Biological Process	Positive Regulation Of DNA Metabolic Process (GO:0051054)	0.0055871	0.1525809	0	0	2.756826674	14.30045086	*YY1;RTEL1;INO80D;BRPF3;USP9X;EYA3;DHX36;ATRX;RNF8;TERF2IP*
Biological Process	Regulation Of mRNA Catabolic Process (GO:0061013)	0.005858	0.1572501	0	0	4.042016807	20.77577623	*YTHDF1;YTHDF3;DHX36;PUM1;DCP2;PUM2*

Biological Process	Transcription Initiation At RNA Polymerase II Promoter (GO:0006367)	0.0059293	0.1572501	0	0	3.174487773	16.27831212	*GTF2A1;DR1;MED21;TAF8;ELOA;PPARGC1A;MED6;MED28*
Biological Process	Positive Regulation Of Translational Initiation (GO:0045948)	0.0061141	0.1572501	0	0	6.330266185	32.26638704	*KHDRBS1;YTHDF1;DDX3X;YTHDF3*
Biological Process	Positive Regulation Of RNA Metabolic Process (GO:0051254)	0.0061324	0.1572501	0	0	4.808191808	24.49377469	*RBM27;U2AF2;DYRK1A;RIOK2;HNRNPLL*
Biological Process	Lipoprotein Transport (GO:0042953)	0.0061737	0.1572501	0	0	10.07949791	51.27908631	*SURF4;MIA3;ZDHHC17*
Biological Process	Positive Regulation Of Circadian Rhythm (GO:0042753)	0.0061737	0.1572501	0	0	10.07949791	51.27908631	*FBXW11;BTRC;CLOCK*
Biological Process	Regulation Of RNA Export From Nucleus (GO:0046831)	0.0061737	0.1572501	0	0	10.07949791	51.27908631	*KHDRBS1;CPSF6;NUP153*
Biological Process	Insulin Receptor Signaling Pathway (GO:0008286)	0.0065154	0.1630776	0	0	3.943226071	19.84855884	*GSK3B;FER;PHIP;IDE;SORBS1;APPL1*
Biological Process	DNA Damage Response (GO:0006974)	0.0065256	0.1630776	0	0	1.812260536	9.119344747	*ATF2;NFAT5;RTEL1;VCP;WDR48;DCUN1D5;PRKDC;NCOA6;ATRX;RNF8;XIAP;SMC6;PPP4R3B;BAZ1B;FBXO31;YY1;ING2;ERCC4;PSME4;WAC;FBXO6;TRIP12;KMT5B;MICA*
Biological Process	Negative Regulation Of Transmembrane Receptor Protein Serine/Threonine Kinase Signaling Pathway (GO:0090101)	0.0067601	0.1673609	0	0	2.674522382	13.36381586	*PPM1A;ING2;RBBP4;SMURF1;PDPK1;VWC2;ARID4A;SPRY2;ARID4B;SPRY1*
Biological Process	Regulation Of DNA Repair (GO:0006282)	0.0069532	0.1705472	0	0	2.5194473	12.51799795	*YY1;MBTD1;PBRM1;INO80D;ING3;BCL7A;EYA3;RNF8;TRIP12;PPP4R3B;ARID1A*
Biological Process	Protein Deubiquitination (GO:0016579)	0.0071908	0.174757	0	0	2.648163491	13.06855044	*USP13;USP8;ATXN3;USP37;USP15;USP9X;USP9Y;SPATA2;JOSD1;OTUD1*
Biological Process	Telomere Organization (GO:0032200)	0.0073296	0.1765092	0	0	3.37026648	16.56768969	*YY1;RTEL1;INO80D;PRKDC;ERCC4;ATRX;TERF2IP*
Biological Process	Regulation Of Protein Ubiquitination (GO:0031396)	0.0076424	0.1823856	0	0	2.622316423	12.78127317	*GSK3B;U2AF2;CUL3;TRAF6;XIAP;CTNNB1;SPRY2;UBE3A;BCL10;SOX4*
Biological Process	Protein Polyubiquitination (GO:0000209)	0.0079548	0.1829969	0	0	2.063852814	9.976623045	*UBE2D4;FBXW11;SMURF1;CUL3;RNF8;LNPEP;UBE3A;UBE3B;UBE2W;KBTBD6;TRAF6;RNF217;CTNNB1;TRIP12;ARIH1;BTRC*
Biological Process	Negative Regulation Of Cysteine-Type Endopeptidase Activity Involved In Apoptotic Process (GO:0043154)	0.0079884	0.1829969	0	0	3.759429353	18.15717907	*DDX3X;DNAJB6;XIAP;CAAP1;RAF1;FNIP1*
Biological Process	Lipoprotein Localization (GO:0044872)	0.0080135	0.1829969	0	0	8.959088796	43.24223806	*SURF4;MIA3;ZDHHC17*
Biological Process	Negative Regulation Of Stem Cell Differentiation (GO:2000737)	0.0080135	0.1829969	0	0	8.959088796	43.24223806	*GSK3B;HNRNPU;N4BP2L2*
Biological Process	Positive Regulation Of Nuclear-Transcribed mRNA poly(A) Tail Shortening (GO:0060213)	0.0080135	0.1829969	0	0	8.959088796	43.24223806	*CPEB3;TNRC6A;TNRC6B*
Biological Process	Regulation Of Canonical Wnt Signaling Pathway (GO:0060828)	0.0081488	0.1840388	0	0	2.115248227	10.1740971	*TLE4;USP8;GSK3B;VCP;DDX3X;XIAP;TMEM170B;MLLT3;PPM1A;ZNRF3;TBL1XR1;ZNF703;BTRC;SOX4;LGR4*
Biological Process	Negative Regulation Of Nucleic Acid-Templated Transcription (GO:1903507)	0.008198	0.1840388	0	0	1.712015257	8.224284625	*KHDRBS1;ARID4A;FOXK1;ATXN1;HEY1;RBBP4;NFIL3;BCL7A;SIX4;ZNF703;BTRC;RUNX1T1;ATF7IP;RBFOX2;ZHX3;POU2F1;FBXW11;ADIPOQ;DNMT3A;TFAP4;KAT6A;TRAF6;CTNNB1;RBAK;CLOCK;RCOR1;VGLL4*
Biological Process	Regulation Of Nuclear-Transcribed mRNA Catabolic Process, Deadenylation-Dependent Decay (GO:1900151)	0.0085462	0.1870984	0	0	5.663334313	26.9703137	*DHX36;HNRNPU;CPEB3;TNRC6B*
Biological Process	Histone mRNA Metabolic Process (GO:0008334)	0.0085462	0.1870984	0	0	5.663334313	26.9703137	*SSB;XRN1;ERI1;DCP2*
Biological Process	Positive Regulation Of mRNA Splicing, Via Spliceosome (GO:0048026)	0.0085462	0.1870984	0	0	5.663334313	26.9703137	*TRA2A;CELF4;CIRBP;RBMX*
Biological Process	Protein Localization (GO:0008104)	0.0086759	0.1883816	0	0	1.81553026	8.618688908	*CEP68;WDR45B;USP9X;SMURF1;ITSN1;VPS13A;RABL2B;HOOK3;ARL17A;AKAP11;SNX27;NBEA;RAB14;KIF3A;RRAGD;CTNNB1;RAB3GAP2;PHIP;STX3;ARL5A;TRIM23;TMED4*
Biological Process	Peptidyl-Threonine Modification (GO:0018210)	0.00941	0.2014899	0	0	3.198397794	14.92365129	*ATF2;GSK3B;PDPK1;PRKDC;DYRK1A;MAPK1;HIPK1*
Biological Process	Organelle Organization (GO:0006996)	0.0094964	0.2014899	0	0	1.728723891	8.050396947	*ATL2;TOR1AIP2;MIA3;ZMYM3;KIF3A;PPARGC1A;WASF3;TMED4;CLASP2;YTHDF1;PDCD6IP;YTHDF3;COG6;MTX3;SURF4;SIRT5;LYSMD3;RAB14;PEX3;KAT6A;GORASP1;PHIP;ARHGEF7;SLC25A36;BRWD3*
Biological Process	Generation Of Neurons (GO:0048699)	0.0095078	0.2014899	0	0	2.211247809	10.29477347	*FZD3;RTN1;MYEF2;USP9X;CEP85L;ATP2B2;RELN;SIX4;FUT9;CASP3;SPOCK1;ASTN2;ADGRL3*

Biological Process	mRNA Splicing, Via Spliceosome (GO:0000398)	0.0096276	0.202046	0	0	2.07164568	9.618893206	*DHX8;GEMIN2;SRSF1;CELF4;DDX20;HNRNPU;CWC27;COIL;U2AF2;HNRNPA2B1;TRA2A;SRSF2;SRSF10;RBMX;SRSF8*
Biological Process	Regulation Of Telomere Maintenance Via Telomerase (GO:0032210)	0.0096866	0.202046	0	0	3.591970121	16.65600228	*XRN1;HNRNPU;MAPK1;CTNNB1;MAP3K4;DCP2*
Biological Process	Stress Granule Assembly (GO:0034063)	0.0099687	0.2063057	0	0	5.379888268	24.79215788	*YTHDF1;DDX3X;YTHDF3;CIRBP*
Biological Process	Cellular Response To Arsenic-Containing Substance (GO:0071243)	0.010142	0.2082652	0	0	8.062761506	37.01668852	*VCP;DDX3X;DHX36*
Biological Process	Positive Regulation Of Cellular Biosynthetic Process (GO:0031328)	0.0104155	0.2122368	0	0	2.183550476	9.966719075	*YTHDF1;DDX3X;SSB;AVPR1B;YTHDF3;PRKDC;CIRBP;SORBS1;CTNNB1;CPEB3;PPARGC1A;SOX4;APPL1*
Biological Process	Cell Cycle G1/S Phase Transition (GO:0044843)	0.0106256	0.2134392	0	0	3.513701133	15.96797282	*PPP6C;KHDRBS1;USP37;CUL3;CUL2;PHF8*
Biological Process	Positive Regulation Of Double-Strand Break Repair (GO:2000781)	0.0106883	0.2134392	0	0	2.839148433	12.88578099	*MBTD1;PBRM1;WDR48;ING3;BCL7A;PRKDC;KMT5B;ARID1A*
Biological Process	Protein Localization To Plasma Membrane (GO:0072659)	0.0107163	0.2134392	0	0	2.358497325	10.69812458	*EFR3A;SMURF1;GORASP1;ADIPOQ;DENND4C;TSPAN5;MYO5A;ZDHHC7;RAPGEF6;LRP6;CLASP2*
Biological Process	Telomere Maintenance (GO:0000723)	0.0110178	0.2178073	0	0	3.093210402	13.94494178	*YY1;RTEL1;INO80D;PRKDC;ERCC4;TERF2IP;SMC6*
Biological Process	Positive Regulation Of Canonical Wnt Signaling Pathway (GO:0090263)	0.0114383	0.21992	0	0	2.802130454	12.5277327	*USP8;PPM1A;VCP;DDX3X;TBL1XR1;XIAP;SOX4;LGR4*
Biological Process	Cellular Response To Hexose Stimulus (GO:0071331)	0.0115362	0.21992	0	0	5.123437084	22.8621493	*PRKAA1;PRKAA2;ZNF236;SOX4*
Biological Process	Negative Regulation Of Telomere Maintenance Via Telomere Lengthening (GO:1904357)	0.0115362	0.21992	0	0	5.123437084	22.8621493	*XRN1;ERCC4;HNRNPU;DCP2*
Biological Process	Endomembrane System Organization (GO:0010256)	0.0116603	0.21992	0	0	2.0808455	9.26301565	*USP8;ARFGEF2;COG6;SURF4;ATL2;TOR1AIP2;MIA3;PLEKHA3;HOOK3;LYSMD3;GORASP1;ARHGEF7;TMED4;CLASP2*
Biological Process	Regulation Of Cell Cycle (GO:0051726)	0.0119047	0.21992	0	0	1.845898588	8.178845119	*DR1;INO80D;STAT3;HNRNPU;XIAP;LIN9;PUM1;HCFC1;LRP6;MBTD1;YY1;PPM1A;ING3;BRINP1;SON;ZNF703;WDR5;TARDBP;ZNF268*
Biological Process	Regulation Of Post-Transcriptional Gene Silencing By RNA (GO:1900368)	0.0120379	0.21992	0	0	17.89879294	79.10720602	*PUM1;PUM2*
Biological Process	Insulin Processing (GO:0030070)	0.0120379	0.21992	0	0	17.89879294	79.10720602	*SLC30A5;YIPF5*
Biological Process	Lipid Droplet Disassembly (GO:1905691)	0.0120379	0.21992	0	0	17.89879294	79.10720602	*PRKAA1;PRKAA2*
Biological Process	Negative Regulation Of Heterochromatin Formation (GO:0031452)	0.0120379	0.21992	0	0	17.89879294	79.10720602	*DYRK1A;PHF8*
Biological Process	Negative Regulation Of Lens Fiber Cell Differentiation (GO:1902747)	0.0120379	0.21992	0	0	17.89879294	79.10720602	*SPRY2;SPRY1*
Biological Process	Positive Regulation Of Pyruvate Dehydrogenase Activity (GO:1904184)	0.0120379	0.21992	0	0	17.89879294	79.10720602	*PDP2;PPM1H*
Biological Process	Regulation Of Hemopoiesis (GO:1903706)	0.0125674	0.223429	0	0	7.329402815	32.07825086	*BRPF3;KAT6A;MYSM1*
Biological Process	Regulation Of Nuclear-Transcribed mRNA poly(A) Tail Shortening (GO:0060211)	0.0125674	0.223429	0	0	7.329402815	32.07825086	*CPEB3;TNRC6A;TNRC6B*
Biological Process	Negative Regulation Of mRNA Splicing, Via Spliceosome (GO:0048025)	0.0125674	0.223429	0	0	7.329402815	32.07825086	*U2AF2;RBMX;SRSF10*
Biological Process	Positive Regulation Of Nuclear-Transcribed mRNA Catabolic Process, Deadenylation-Dependent Decay (GO:1900153)	0.0125674	0.223429	0	0	7.329402815	32.07825086	*DHX36;CPEB3;TNRC6B*
Biological Process	Protein Localization To Nucleus (GO:0034504)	0.012756	0.225271	0	0	2.410462777	10.51384358	*SIX4;CSE1L;STAT3;TAF8;HNRNPU;TOR1AIP2;NUP153;RPF2;TNPO1;APPL1*
Biological Process	Glucose Homeostasis (GO:0042593)	0.0130537	0.2290009	0	0	2.730905988	11.84854591	*PRKAA1;MBD5;PRKAA2;ADIPOQ;STAT3;FOXK1;PPARGC1A;SOX4*
Biological Process	Protein Localization To Cell Periphery (GO:1990778)	0.0137581	0.2397717	0	0	2.267886174	9.720443474	*EFR3A;SMURF1;ADIPOQ;DENND4C;TSPAN5;MYO5A;ZDHHC7;LIN7C;RAPGEF6;LRP6;CLASP2*
Biological Process	Cellular Response To Peptide Hormone Stimulus (GO:0071375)	0.0148299	0.245796	0	0	2.663198779	11.21502513	*FER;PDPK1;PRKDC;PDE3B;ADIPOQ;DENND4C;MYO5A;SORBS1*

Biological Process	Regulation Of Stem Cell Population Maintenance (GO:2000036)	0.0150289	0.245796	0	0	3.231932773	13.56694551	*ING2;BCL7A;RBBP4;ARID4A;ARID4B;ARID1A*
Biological Process	Positive Regulation Of DNA-templated Transcription Initiation (GO:2000144)	0.0150289	0.245796	0	0	3.231932773	13.56694551	*GTF2A1;MED21;DHX36;TAF8;MED6;MED28*
Biological Process	post-Golgi Vesicle-Mediated Transport (GO:0006892)	0.0150289	0.245796	0	0	3.231932773	13.56694551	*ARFGEF2;RAB14;VPS13A;MYO5A;EXOC6;SCAMP1*
Biological Process	Protein Quality Control For Misfolded Or Incompletely Synthesized Proteins (GO:0006515)	0.0151253	0.245796	0	0	4.677435026	19.60494242	*ATXN3;UBE2W;VCP;CUL3*
Biological Process	Regulation Of Cellular Response To Heat (GO:1900034)	0.0152954	0.245796	0	0	6.718270572	28.08373375	*GSK3B;DNAJB6;EP300*
Biological Process	Cardiac Myofibril Assembly (GO:0055003)	0.0152954	0.245796	0	0	6.718270572	28.08373375	*MEF2A;NEBL;TTN*
Biological Process	Regulation Of Telomere Maintenance In Response To DNA Damage (GO:1904505)	0.0152954	0.245796	0	0	6.718270572	28.08373375	*YY1;RTEL1;INO80D*
Biological Process	Sympathetic Nervous System Development (GO:0048485)	0.0152954	0.245796	0	0	6.718270572	28.08373375	*FZD3;CTNNB1;SOX4*
Biological Process	Negative Regulation Of mRNA Processing (GO:0050686)	0.0152954	0.245796	0	0	6.718270572	28.08373375	*U2AF2;RBMX;SRSF10*
Biological Process	Negative Regulation Of Telomere Maintenance (GO:0032205)	0.0152954	0.245796	0	0	6.718270572	28.08373375	*RTEL1;ERCC4;TERF2IP*
Biological Process	Regulation Of Microtubule-Based Process (GO:0032886)	0.0153101	0.245796	0	0	3.738150738	15.62264883	*GSK3B;PRKAA1;PRKAA2;MEMO1;CLASP2*
Biological Process	Stem Cell Differentiation (GO:0048863)	0.0153101	0.245796	0	0	3.738150738	15.62264883	*CTNNB1;MLLT3;JARID2;PUM1;LRP6*
Biological Process	Regulation Of Transcription Initiation By RNA Polymerase II (GO:0060260)	0.0163014	0.2585771	0	0	3.16839677	13.04272165	*GTF2A1;MED21;DHX36;TAF8;MED6;MED28*
Biological Process	Post-Transcriptional Regulation Of Gene Expression (GO:0010608)	0.0163014	0.2585771	0	0	3.16839677	13.04272165	*CELF1;NOLC1;MSI2;ATG14;PUM1;PUM2*
Biological Process	Regulation Of RNA Metabolic Process (GO:0051252)	0.0167753	0.2638317	0	0	2.598754569	10.623309	*MBNL2;SON;CELF1;RASA1;ZNF638;SRSF1;HNRNPLL;PTBP3*
Biological Process	Negative Regulation Of Developmental Process (GO:0051093)	0.0168917	0.2638317	0	0	2.427848101	9.907886325	*ING2;BCL7A;HEY1;RBBP4;NFIB;PHF14;ARID4A;ARID4B;ARPIN*
Biological Process	Transcription Elongation By RNA Polymerase II (GO:0006368)	0.0171555	0.2638317	0	0	4.482309125	18.2225344	*USP15;PBRM1;SUPT16H;SUPT5H*
Biological Process	G-quadruplex DNA Unwinding (GO:0044806)	0.0176286	0.2638317	0	0	13.42339833	54.20678961	*DHX36;HNRNPA2B1*
Biological Process	Regulation Of Lens Fiber Cell Differentiation (GO:1902746)	0.0176286	0.2638317	0	0	13.42339833	54.20678961	*SPRY2;SPRY1*
Biological Process	Regulation Of Mitochondrial Membrane Permeability Involved In Apoptotic Process (GO:1902108)	0.0176286	0.2638317	0	0	13.42339833	54.20678961	*ATF2;THEM4*
Biological Process	Antigen Processing And Presentation Of Endogenous Peptide Antigen Via MHC Class I (GO:0019885)	0.0176286	0.2638317	0	0	13.42339833	54.20678961	*ERAP1;IDE*
Biological Process	Regulation Of Pyruvate Dehydrogenase Activity (GO:1904182)	0.0176286	0.2638317	0	0	13.42339833	54.20678961	*PDP2;PPM1H*
Biological Process	Detection Of Muscle Stretch (GO:0035995)	0.0176286	0.2638317	0	0	13.42339833	54.20678961	*CTNNB1;TTN*
Biological Process	Negative Regulation Of Smooth Muscle Cell Apoptotic Process (GO:0034392)	0.0176286	0.2638317	0	0	13.42339833	54.20678961	*DNMT1;LRP6*
Biological Process	Negative Regulation Of Phosphorylation (GO:0042326)	0.0182179	0.2711194	0	0	2.773781041	11.10996329	*PRKDC;HNRNPU;TERF2IP;ATG14;TARDBP;CRKL;LRP6*
Biological Process	Positive Regulation Of Telomere Capping (GO:1904355)	0.0183302	0.2712668	0	0	6.201158674	24.79969825	*RTEL1;MAPK1;MAP3K4*

Biological Process	Intracellular Steroid Hormone Receptor Signaling Pathway (GO:0030518)	0.0185501	0.2714877	0	0	3.541037909	14.11910515	*RBFOX2;MAPK1;UBE3A;ZDHHC7;ARID1A*
Biological Process	Positive Regulation Of Transcription Elongation By RNA Polymerase II (GO:0032968)	0.0185501	0.2714877	0	0	3.541037909	14.11910515	*ELL;ZMYND8;MED21;MED6;MED28*
Biological Process	Double-Strand Break Repair (GO:0006302)	0.0186617	0.2716205	0	0	2.077792264	8.272273435	*YY1;MBTD1;VCP;WDR48;PRKDC;ERCC4;ZMYND8;EYA3;NSD2;RNF8;SMARCAD1;SMC6*
Biological Process	RNA Metabolic Process (GO:0016070)	0.0188079	0.2722524	0	0	2.379995036	9.456855435	*RBFOX2;ATXN1;DDX3X;DHX8;AGO3;DIS3;DHX36;DDX20;HNRNPU*
Biological Process	Mitotic Cytokinesis (GO:0000281)	0.0190654	0.2742897	0	0	3.04851752	12.0717651	*USP8;PDCD6IP;SON;RASA1;EXOC6;CNTROB*
Biological Process	Golgi Organization (GO:0007030)	0.0191558	0.2742897	0	0	2.248826291	8.894450269	*COG6;GORASP1;SURF4;ATL2;YIPF5;LYSMD3;ARHGEF7;TMED4;CLASP2;BET1*
Biological Process	Protein Acetylation (GO:0006473)	0.0193479	0.2755514	0	0	4.302793296	16.9752588	*KAT6A;EP300;NAA15;CLOCK*
Biological Process	Androgen Receptor Signaling Pathway (GO:0030521)	0.0216742	0.2963814	0	0	5.757919904	22.06224675	*MAPK1;ZDHHC7;ARID1A*
Biological Process	Embryo Development Ending In Birth Or Egg Hatching (GO:0009792)	0.0216742	0.2963814	0	0	5.757919904	22.06224675	*OSR2;CELF1;CELF4*
Biological Process	mRNA Cis Splicing, Via Spliceosome (GO:0045292)	0.0216742	0.2963814	0	0	5.757919904	22.06224675	*PRPF39;SRSF1;PSIP1*
Biological Process	Peptidyl-Threonine Dephosphorylation (GO:0035970)	0.0216742	0.2963814	0	0	5.757919904	22.06224675	*DUSP5;PPM1A;PPM1G*
Biological Process	Positive Regulation Of Dendrite Extension (GO:1903861)	0.0216742	0.2963814	0	0	5.757919904	22.06224675	*SYT4;SMURF1;PLAA*
Biological Process	Regulation Of DNA Methylation-Dependent Heterochromatin Formation (GO:0090308)	0.0216742	0.2963814	0	0	5.757919904	22.06224675	*ATF7IP;SETDB1;DYRK1A*
Biological Process	Regulation Of Aerobic Respiration (GO:1903715)	0.0216742	0.2963814	0	0	5.757919904	22.06224675	*VCP;UQCC2;IDE*
Biological Process	Protein Palmitoylation (GO:0018345)	0.0217055	0.2963814	0	0	4.137086377	15.84581625	*ZDHHC13;ZDHHC21;ZDHHC7;ZDHHC17*
Biological Process	BMP Signaling Pathway (GO:0030509)	0.0221329	0.297635	0	0	2.937356761	11.19336229	*USP15;SMURF1;USP9X;USP9Y;HIVEP1;SMAD5*
Biological Process	Regulation Of Innate Immune Response (GO:0045088)	0.0221344	0.297635	0	0	2.656164194	10.12163581	*PPP6C;PRKDC;POLR3C;ERAP1;POLR3F;XIAP;APPL1*
Biological Process	Cellular Response To Nutrient Levels (GO:0031669)	0.0221344	0.297635	0	0	2.656164194	10.12163581	*PRKAA1;PRKAA2;WDR45B;RRAGD;JMY;ATG14;FNIP1*
Biological Process	Response To Insulin (GO:0032868)	0.02356	0.2999465	0	0	2.619136668	9.817064157	*FER;PDPK1;PRKDC;PDE3B;DENND4C;MYO5A;SORBS1*
Biological Process	Negative Regulation Of Cellular Component Organization (GO:0051129)	0.02356	0.2999465	0	0	2.619136668	9.817064157	*GSK3B;RTEL1;DDX3X;DNAJB6;EP300;SPRY2;RAF1*
Biological Process	Positive Regulation Of Protein Modification By Small Protein Conjugation Or Removal (GO:1903322)	0.02356	0.2999465	0	0	2.619136668	9.817064157	*GSK3B;DCUN1D5;TRAF6;CUL3;XIAP;UBE3A;BCL10*
Biological Process	Regulation Of Protein Localization (GO:0032880)	0.0237069	0.2999465	0	0	2.422800151	9.066092823	*USP8;VCP;PRKAA1;PRKAA2;DNAJB6;RAB14;CTNNB1;ZDHHC7*
Biological Process	Protein Lipidation (GO:0006497)	0.0237844	0.2999465	0	0	2.884753902	10.78530205	*WDR45B;SLC30A5;ZDHHC13;ZDHHC7;ZDHHC21;ZDHHC17*
Biological Process	Amino-Acid Betaine Transport (GO:0015838)	0.0240966	0.2999465	0	0	10.73816156	40.00701614	*SLC7A6;SLC22A5*
Biological Process	Regulation Of Phospholipase A2 Activity (GO:0032429)	0.0240966	0.2999465	0	0	10.73816156	40.00701614	*AVPR1B;PLAA*
Biological Process	Response To UV-C (GO:0010225)	0.0240966	0.2999465	0	0	10.73816156	40.00701614	*YY1;MAP3K4*
Biological Process	Deadenylation-Dependent Decapping Of Nuclear-Transcribed mRNA (GO:0000290)	0.0240966	0.2999465	0	0	10.73816156	40.00701614	*DCP1A;DCP2*
Biological Process	Store-Operated Calcium Entry (GO:0002115)	0.0240966	0.2999465	0	0	10.73816156	40.00701614	*ORAI3;ORAI2*
Biological Process	Negative Regulation Of Protein K63-linked Ubiquitination (GO:1900045)	0.0240966	0.2999465	0	0	10.73816156	40.00701614	*PLAA;TRIP12*
Biological Process	Peptidyl-Serine Autophosphorylation (GO:0036289)	0.0240966	0.2999465	0	0	10.73816156	40.00701614	*DYRK1A;MARK3*
Biological Process	Protein Localization To Cytoplasmic Stress Granule (GO:1903608)	0.0240966	0.2999465	0	0	10.73816156	40.00701614	*DDX3X;SSB*

Biological Process	Response To Glucose (GO:0009749)	0.024206	0.2999465	0	0	3.281425891	12.21068834	*PRKAA1;PRKAA2;ADIPOQ;ZNF236;SOX4*
Biological Process	Regulation Of Response To Biotic Stimulus (GO:0002831)	0.0242312	0.2999465	0	0	3.983654045	14.8196413	*ELMOD2;ERAP1;XIAP;APPL1*
Biological Process	Regulation Of Transmembrane Receptor Protein Serine/Threonine Kinase Signaling Pathway (GO:0090092)	0.0242312	0.2999465	0	0	3.983654045	14.8196413	*FSTL5;ZNF703;XIAP;NREP*
Biological Process	Response To Estradiol (GO:0032355)	0.0242312	0.2999465	0	0	3.983654045	14.8196413	*ZNF703;STAT3;CTNNB1;LCOR*
Biological Process	Positive Regulation Of I-kappaB kinase/NF-kappaB Signaling (GO:0043123)	0.0247649	0.3035917	0	0	1.987834044	7.351662832	*PPM1A;TRAF6;DHX36;TIFA;ZDHHC13;TERF2IP;BCL10;ZDHHC17;MID2;TMEM9B;MTDH;TMED4*
Biological Process	Nervous System Development (GO:0007399)	0.0248451	0.3035917	0	0	1.591012562	5.878941009	*MBD5;RBFOX2;APAF1;ZMYND8;STAT3;SIAH1;DYRK1A;MSI2;NAV2;NR2C2;IRF2BPL;SLC25A16;ATXN3;MARCKS;ATXN1;RELN;SPOCK2;EP300;SPOCK1;CNTN4;JARID2;EPHB1;SOX4;ADGRL3*
Biological Process	Negative Regulation Of Protein Serine/Threonine Kinase Activity (GO:0071901)	0.0250475	0.3035917	0	0	2.583123595	9.523931168	*TFAP4;PRKAR2A;ADIPOQ;SPRY2;SPRY1;PIK3CB;LRP6*
Biological Process	Regulation Of Dendrite Extension (GO:1903859)	0.025328	0.3035917	0	0	5.373779637	19.75318868	*SYT4;SMURF1;PLAA*
Biological Process	Negative Regulation Of DNA-templated Transcription, Elongation (GO:0032785)	0.025328	0.3035917	0	0	5.373779637	19.75318868	*RNF8;HNRNPU;SUPT5H*
Biological Process	Positive Regulation Of Glycolytic Process (GO:0045821)	0.025328	0.3035917	0	0	5.373779637	19.75318868	*PRKAA1;PRKAA2;SLC4A4*
Biological Process	Regulation Of RIG-I Signaling Pathway (GO:0039535)	0.025328	0.3035917	0	0	5.373779637	19.75318868	*USP15;PUM1;PUM2*
Biological Process	G1/S Transition Of Mitotic Cell Cycle (GO:0000082)	0.0255165	0.3044739	0	0	2.833996757	10.39631912	*PPP6C;KHDRBS1;USP37;CUL3;CUL2;PHF8*
Biological Process	Positive Regulation Of TOR Signaling (GO:0032008)	0.0263121	0.312559	0	0	3.203130203	11.65211517	*USP9X;RRAGD;WAC;FNIP1;GPR137C*
Biological Process	Regulation Of Metaphase/Anaphase Transition Of Cell Cycle (GO:1902099)	0.0269273	0.3170239	0	0	3.841181165	13.88439207	*PBRM1;BCL7A;RIOK2;ARID1A*
Biological Process	Histone Lysine Methylation (GO:0034968)	0.0269273	0.3170239	0	0	3.841181165	13.88439207	*SETDB1;NCOA6;WDR5;HCFC1*
Biological Process	Regulation Of Translational Initiation (GO:0006446)	0.0292278	0.3373559	0	0	2.73764421	9.671096219	*KHDRBS1;YTHDF1;DDX3X;YTHDF3;EIF1B;NCK1*
Biological Process	Regulation Of Cellular Response To Growth Factor Stimulus (GO:0090287)	0.029291	0.3373559	0	0	5.037656904	17.78532228	*FSTL5;XIAP;ZDHHC17*
Biological Process	Negative Regulation Of Telomere Maintenance Via Telomerase (GO:0032211)	0.029291	0.3373559	0	0	5.037656904	17.78532228	*XRN1;HNRNPU;DCP2*
Biological Process	rRNA Catabolic Process (GO:0016075)	0.029291	0.3373559	0	0	5.037656904	17.78532228	*XRN1;DIS3;PELO*
Biological Process	Regulation Of Behavior (GO:0050795)	0.029291	0.3373559	0	0	5.037656904	17.78532228	*MBD5;RELN;STAT3*
Biological Process	Positive Regulation Of Chromosome Organization (GO:2001252)	0.0297956	0.3398501	0	0	3.708534001	13.02954897	*RTEL1;DHX36;ATRX;TERF2IP*
Biological Process	Vesicle-Mediated Transport To The Plasma Membrane (GO:0098876)	0.0298924	0.3398501	0	0	2.48077065	8.707876556	*DENND1B;ARFGEF2;SNX27;RAB14;VPS50;EXOC6;VAMP4*
Biological Process	Positive Regulation Of Ubiquitin-Dependent Protein Catabolic Process (GO:2000060)	0.0298924	0.3398501	0	0	2.48077065	8.707876556	*GSK3B;NUB1;VCP;SMURF1;SUMO2;RNF217;ARIH1*
Biological Process	Regulation Of Mitotic Metaphase/Anaphase Transition (GO:0030071)	0.030866	0.3414763	0	0	3.057215512	10.63330413	*PBRM1;BCL7A;CUL3;RIOK2;ARID1A*
Biological Process	Cellular Response To BMP Stimulus (GO:0071773)	0.0312096	0.3414763	0	0	2.691876751	9.33281667	*USP15;SMURF1;USP9X;USP9Y;HIVEP1;SMAD5*
Biological Process	Brown Fat Cell Differentiation (GO:0050873)	0.0313715	0.3414763	0	0	8.948003714	30.97669	*ADIPOQ;PPARGC1A*
Biological Process	Regulation Of Type B Pancreatic Cell Development (GO:2000074)	0.0313715	0.3414763	0	0	8.948003714	30.97669	*GSK3B;CLOCK*

Biological Process	Sympathetic Ganglion Development (GO:0061549)	0.0313715	0.3414763	0	0	8.948003714	30.97669	*FZD3;CTNNB1*
Biological Process	Maintenance Of Apical/Basal Cell Polarity (GO:0035090)	0.0313715	0.3414763	0	0	8.948003714	30.97669	*PDCD6IP;LIN7C*
Biological Process	Maintenance Of Epithelial Cell Apical/Basal Polarity (GO:0045199)	0.0313715	0.3414763	0	0	8.948003714	30.97669	*PDCD6IP;LIN7C*
Biological Process	Negative Regulation Of Chromosome Organization (GO:2001251)	0.0313715	0.3414763	0	0	8.948003714	30.97669	*ERCC4;TERF2IP*
Biological Process	Positive Regulation Of ATP Biosynthetic Process (GO:2001171)	0.0313715	0.3414763	0	0	8.948003714	30.97669	*VCP;PPARGC1A*
Biological Process	Positive Regulation Of Dendritic Spine Morphogenesis (GO:0061003)	0.0313715	0.3414763	0	0	8.948003714	30.97669	*RELN;DHX36*
Biological Process	Regulation Of Translation (GO:0006417)	0.0314535	0.3414763	0	0	1.857337434	6.424987404	*YTHDF1;DDX3X;SSB;YTHDF3;PRKDC;CIRBP;NOLC1;MSI2;DHX36;CPEB3;EIF4E2;EIF1B;SOX4*
Biological Process	Positive Regulation Of Protein Ubiquitination (GO:0031398)	0.0316383	0.3420807	0	0	2.448425347	8.45536215	*GSK3B;DCUN1D5;TRAF6;CUL3;XIAP;UBE3A;BCL10*
Biological Process	Positive Regulation Of Macromolecule Biosynthetic Process (GO:0010557)	0.0322385	0.3424668	0	0	2.147081886	7.374356214	*YTHDF1;DDX3X;SSB;YTHDF3;PRKDC;CIRBP;SORBS1;CPEB3;SOX4*
Biological Process	Protein Autophosphorylation (GO:0046777)	0.0326978	0.3424668	0	0	1.965448958	6.722713424	*GSK3B;FER;EEF2K;PDPK1;PEAK1;STK17B;DYRK1A;RIOK2;EPHB1;MARK3;CAMKK2*
Biological Process	DNA Damage Checkpoint Signaling (GO:0000077)	0.0328375	0.3424668	0	0	3.584729981	12.24609638	*ERCC4;FBXO6;INTS7;CLOCK*
Biological Process	Cellular Response To Glucose Stimulus (GO:0071333)	0.0328375	0.3424668	0	0	3.584729981	12.24609638	*PRKAA1;PRKAA2;ZNF236;SOX4*
Biological Process	Inner Mitochondrial Membrane Organization (GO:0007007)	0.0328375	0.3424668	0	0	3.584729981	12.24609638	*CHCHD3;TIMM29;MTX3;IMMT*
Biological Process	Negative Regulation Of Ras Protein Signal Transduction (GO:0046580)	0.0328375	0.3424668	0	0	3.584729981	12.24609638	*CUL3;SPRY2;SPRY1;SCAI*
Biological Process	Positive Regulation Of Carbohydrate Metabolic Process (GO:0045913)	0.0328375	0.3424668	0	0	3.584729981	12.24609638	*PRKAA1;PRKAA2;AVPR1B;SLC4A4*
Biological Process	Positive Regulation Of Organelle Organization (GO:0010638)	0.0328375	0.3424668	0	0	3.584729981	12.24609638	*GSK3B;SURF4;RAB3GAP2;PPARGC1A*
Biological Process	Protein Targeting To Vacuole (GO:0006623)	0.0328375	0.3424668	0	0	3.584729981	12.24609638	*AP3M1;SMURF1;VPS13A;ATG14*
Biological Process	Positive Regulation Of DNA-binding Transcription Factor Activity (GO:0051091)	0.0333089	0.3445891	0	0	1.754536244	5.968810315	*ATF2;STAT3;BCL10;MID2;MTDH;LRP6;FER;RELN;TRAF6;JMY;CTNNB1;EP300;TERF2IP;CLOCK;PPARGC1A*
Biological Process	Negative Regulation Of Stem Cell Population Maintenance (GO:1902455)	0.0335613	0.3445891	0	0	4.741078021	16.09302334	*ING2;RBBP4;ARID4B*
Biological Process	Positive Regulation Of Phospholipase Activity (GO:0010518)	0.0335613	0.3445891	0	0	4.741078021	16.09302334	*AVPR1B;PDPK1;PLAA*
Biological Process	Positive Regulation Of Vascular Associated Smooth Muscle Cell Proliferation (GO:1904707)	0.0335613	0.3445891	0	0	4.741078021	16.09302334	*DNMT1;FOXJ2;MEF2D*
Biological Process	Protein Dephosphorylation (GO:0006470)	0.0352511	0.3585971	0	0	2.109521189	7.056896472	*PPP6C;DUSP5;PPM1A;PDP2;FBXW11;PPM1H;PPP4R3B;NCK1;PPM1G*
Biological Process	Regulation Of Mitotic Cell Cycle Phase Transition (GO:1901990)	0.0353318	0.3585971	0	0	2.386191347	7.976973033	*PBRM1;TFAP4;BCL7A;CUL3;RIOK2;ARID1A;APPL1*
Biological Process	Negative Regulation Of Angiogenesis (GO:0016525)	0.0353318	0.3585971	0	0	2.386191347	7.976973033	*ATF2;PDE3B;FOXJ2;CTNNB1;SPRY2;PIK3CB;GTF2I*
Biological Process	Regulatory ncRNA Processing (GO:0070918)	0.0360542	0.3645328	0	0	3.468913318	11.52626807	*AGO3;PUM1;TSN;PUM2*
Biological Process	Negative Regulation Of Macromolecule Metabolic Process (GO:0010605)	0.0371337	0.3705434	0	0	1.861094876	6.129014089	*YY1;GSK3B;DNMT1;PRKAA1;PRKAA2;SETDB1;CELF1;SFMBT2;STAT3;TARDBP;SMAD5;CRKL*
Biological Process	Regulation Of Histone Deacetylation (GO:0031063)	0.0381359	0.3705434	0	0	4.477452348	14.62604394	*DR1;ING2;WDR5*

Biological Process	Antigen Processing And Presentation Of Endogenous Peptide Antigen (GO:0002483)	0.0381359	0.3705434	0	0	4.477452348	14.62604394	*ERAP1;IDE;MICA*
Biological Process	Focal Adhesion Assembly (GO:0048041)	0.0381359	0.3705434	0	0	4.477452348	14.62604394	*PEAK1;SORBS1;ARHGEF7*
Biological Process	Peptide Catabolic Process (GO:0043171)	0.0381359	0.3705434	0	0	4.477452348	14.62604394	*ERAP1;LNPEP;IDE*
Biological Process	Positive Regulation Of Dendritic Spine Development (GO:0060999)	0.0381359	0.3705434	0	0	4.477452348	14.62604394	*RELN;DHX36;CPEB3*
Biological Process	Cellular Response To Starvation (GO:0009267)	0.0384516	0.3705434	0	0	2.073244618	6.755366645	*PRKAA1;PRKAA2;WDR45B;BMT2;RRAGD;JMY;MAPK1;ATG14;FNIP1*
Biological Process	Positive Regulation Of Transcription Initiation By RNA Polymerase II (GO:0060261)	0.0385725	0.3705434	0	0	2.861627734	9.315211941	*GTF2A1;MED21;TAF8;MED6;MED28*
Biological Process	Regulation Of Cellular Response To Stress (GO:0080135)	0.0393409	0.3705434	0	0	2.176937919	7.043464374	*YY1;GSK3B;INO80D;DNAJB6;FEM1B;EP300;OPRM1;SOX4*
Biological Process	Adherens Junction Assembly (GO:0034333)	0.0393872	0.3705434	0	0	7.669319538	24.80500011	*ZNF703;CTNNB1*
Biological Process	Regulation Of Protein Acetylation (GO:1901983)	0.0393872	0.3705434	0	0	7.669319538	24.80500011	*GSK3B;DDX3X*
Biological Process	Telomere Maintenance Via Recombination (GO:0000722)	0.0393872	0.3705434	0	0	7.669319538	24.80500011	*ERCC4;SMC6*
Biological Process	Iron Ion Transmembrane Transport (GO:0034755)	0.0393872	0.3705434	0	0	7.669319538	24.80500011	*ABCB7;SLC11A2*
Biological Process	Negative Regulation Of Vascular Endothelial Growth Factor Signaling Pathway (GO:1900747)	0.0393872	0.3705434	0	0	7.669319538	24.80500011	*SPRY2;PIK3CB*
Biological Process	Positive Regulation Of ATP Metabolic Process (GO:1903580)	0.0393872	0.3705434	0	0	7.669319538	24.80500011	*VCP;PPARGC1A*
Biological Process	Positive Regulation Of mRNA 3'-End Processing (GO:0031442)	0.0393872	0.3705434	0	0	7.669319538	24.80500011	*DHX36;CPEB3*
Biological Process	Positive Regulation Of miRNA-mediated Gene Silencing (GO:2000637)	0.0393872	0.3705434	0	0	7.669319538	24.80500011	*PUM1;PUM2*
Biological Process	Protein Localization To Microtubule (GO:0035372)	0.0393872	0.3705434	0	0	7.669319538	24.80500011	*HNRNPU;MID2*
Biological Process	COPII-coated Vesicle Budding (GO:0090114)	0.0394463	0.3705434	0	0	3.360335196	10.86334219	*PPP6C;CUL3;MIA3;SEC23B*
Biological Process	Protein Destabilization (GO:0031648)	0.0394463	0.3705434	0	0	3.360335196	10.86334219	*FBXW11;CUL3;EP300;FBXL3*
Biological Process	Cytosolic Transport (GO:0016482)	0.0401237	0.375575	0	0	2.055567475	6.610268608	*RAB14;AP1G1;VPS50;VPS13A;PLEKHA3;TBC1D23;HOOK3;ATG14;EVI5*
Biological Process	Negative Regulation Of Cysteine-Type Endopeptidase Activity (GO:2000117)	0.0413793	0.3833526	0	0	2.801864802	8.923864879	*DDX3X;DNAJB6;XIAP;RAF1;FNIP1*
Biological Process	Positive Regulation Of NIK/NF-kappaB Signaling (GO:1901224)	0.0413793	0.3833526	0	0	2.801864802	8.923864879	*NFAT5;DDX3X;TRAF6;EP300;TERF2IP*
Biological Process	Positive Regulation Of DNA Repair (GO:0045739)	0.0413888	0.3833526	0	0	2.29853248	7.32024163	*YY1;PBRM1;INO80D;BCL7A;EYA3;RNF8;ARID1A*
Biological Process	Fc-epsilon Receptor Signaling Pathway (GO:0038095)	0.0430105	0.3873389	0	0	4.241576745	13.34531793	*FER;TRAF6;SOS1*
Biological Process	Regulation Of Telomere Capping (GO:1904353)	0.0430105	0.3873389	0	0	4.241576745	13.34531793	*RTEL1;MAPK1;MAP3K4*
Biological Process	Membrane Protein Ectodomain Proteolysis (GO:0006509)	0.0430105	0.3873389	0	0	4.241576745	13.34531793	*ERAP1;SPPL3;RBMX*
Biological Process	Regulation Of Catabolic Process (GO:0009894)	0.0430105	0.3873389	0	0	4.241576745	13.34531793	*NUB1;UBE3A;ZDHHC7*
Biological Process	RNA Stabilization (GO:0043489)	0.043014	0.3873389	0	0	3.258337566	10.25147475	*DHX36;CIRBP;HNRNPU;TRDMT1*
Biological Process	Regulation Of Vascular Associated Smooth Muscle Cell Proliferation (GO:1904705)	0.043014	0.3873389	0	0	3.258337566	10.25147475	*DNMT1;ADIPOQ;FOXJ2;MEF2D*
Biological Process	Spliceosomal snRNP Assembly (GO:0000387)	0.043014	0.3873389	0	0	3.258337566	10.25147475	*COIL;GEMIN2;DDX20;SRSF10*
Biological Process	Regulation Of Proteasomal Ubiquitin-Dependent Protein Catabolic Process (GO:0032434)	0.0431351	0.3873389	0	0	2.133607743	6.706819624	*GSK3B;NUB1;VCP;USP9X;RNF217;SUMO2;WAC;ARIH1*

Biological Process	Positive Regulation Of Wnt Signaling Pathway (GO:0030177)	0.0431351	0.3873389	0	0	2.133607743	6.706819624	*USP8;PPM1A;VCP;DDX3X;TBL1XR1;XIAP;SOX4;LGR4*
Biological Process	Negative Regulation Of Apoptotic Process (GO:0043066)	0.0440442	0.3938516	0	0	1.481589346	4.626353744	*GSK3B;PRKAA1;PRKAA2;DDX3X;PRKDC;XIAP;CBL;MTDH;DNAJB6;SIX4;FNIP1;PPARGC1A;ZNF268;FZD3;KDM2B;API5;SMAD5;SON;RASA1;CTNNB1;SPRY2;PHIP;NAA15;RAF1;GRINA*
Biological Process	Negative Regulation Of TOR Signaling (GO:0032007)	0.0443064	0.3938516	0	0	2.744541173	8.553705755	*PRKAA1;PRKAA2;TMEM127;BMT2;FNIP1*
Biological Process	Organelle Disassembly (GO:1903008)	0.0443064	0.3938516	0	0	2.744541173	8.553705755	*PRKAA1;VCP;PRKAA2;PELO;MRRF*
Biological Process	Response To Ionizing Radiation (GO:0010212)	0.0449327	0.396756	0	0	2.446396741	7.590163301	*PRKAA1;ERCC4;EYA3;RNF8;INTS7;CLOCK*
Biological Process	Transforming Growth Factor Beta Receptor Signaling Pathway (GO:0007179)	0.0449327	0.396756	0	0	2.446396741	7.590163301	*USP15;USP9X;USP9Y;STAT3;SMAD5;APPL1*
Biological Process	Transmembrane Receptor Protein Serine/Threonine Kinase Signaling Pathway (GO:0007178)	0.0454333	0.3988168	0	0	2.004289152	6.196278497	*USP15;SMURF1;USP9X;USP9Y;STAT3;HIVEP1;SMAD5;RNF111;APPL1*
Biological Process	Protein Localization To Membrane (GO:0072657)	0.0454672	0.3988168	0	0	1.798493409	5.558717802	*EFR3A;SMURF1;ADIPOQ;DENND4C;TSPAN5;MYO5A;ZDHHC7;LIN7C;ARL5A;RAPGEF6;LRP6;CLASP2*
Biological Process	Mitotic Cell Cycle Phase Transition (GO:0044772)	0.0471634	0.402616	0	0	2.091960292	6.389133054	*KHDRBS1;PPP6C;USP37;CUL3;CCNG2;CUL2;PHF8;CLASP2*
Biological Process	Neuron Migration (GO:0001764)	0.0473544	0.402616	0	0	2.68951049	8.20326404	*RELN;USP9X;CEP85L;SPOCK1;ASTN2*
Biological Process	Regulation Of mRNA Polyadenylation (GO:1900363)	0.0480809	0.402616	0	0	6.710306407	20.36491453	*CPSF6;CPEB3*
Biological Process	Regulation Of Peptidyl-Lysine Acetylation (GO:2000756)	0.0480809	0.402616	0	0	6.710306407	20.36491453	*PRKAA1;PRKAA2*
Biological Process	Regulation Of Phospholipase Activity (GO:0010517)	0.0480809	0.402616	0	0	6.710306407	20.36491453	*PDPK1;RASGRP1*
Biological Process	Cell Proliferation In Forebrain (GO:0021846)	0.0480809	0.402616	0	0	6.710306407	20.36491453	*CEP120;HOOK3*
Biological Process	Cellular Response To Interleukin-15 (GO:0071350)	0.0480809	0.402616	0	0	6.710306407	20.36491453	*MFAP3;STAT3*
Biological Process	Steroid Hormone Mediated Signaling Pathway (GO:0043401)	0.0480809	0.402616	0	0	6.710306407	20.36491453	*MAPK1;ZDHHC7*
Biological Process	Fatty Acid Homeostasis (GO:0055089)	0.0480809	0.402616	0	0	6.710306407	20.36491453	*PRKAA1;PRKAA2*
Biological Process	Interleukin-15-Mediated Signaling Pathway (GO:0035723)	0.0480809	0.402616	0	0	6.710306407	20.36491453	*MFAP3;STAT3*
Biological Process	Microtubule Anchoring At Centrosome (GO:0034454)	0.0480809	0.402616	0	0	6.710306407	20.36491453	*KIF3A;HOOK3*
Biological Process	Positive Regulation Of Post-Transcriptional Gene Silencing By RNA (GO:1900370)	0.0480809	0.402616	0	0	6.710306407	20.36491453	*PUM1;PUM2*
Biological Process	Progesterone Receptor Signaling Pathway (GO:0050847)	0.0480809	0.402616	0	0	6.710306407	20.36491453	*UBE3A;ZDHHC7*
Biological Process	Regulation Of ERAD Pathway (GO:1904292)	0.0480809	0.402616	0	0	6.710306407	20.36491453	*USP13;ATXN3*
Biological Process	Negative Regulation Of Neuron Differentiation (GO:0045665)	0.0481802	0.402616	0	0	4.029288703	12.22005799	*GSK3B;HEY1;CNTN4*
Biological Process	Neuron Differentiation (GO:0030182)	0.0487296	0.4059267	0	0	1.830939073	5.532125253	*FZD3;RTN1;PBX2P1;MYEF2;FUT9;CASP3;SPOCK1;CTNNB1;ATP2B2;SOX4;LRP6*
Biological Process	RNA Polymerase II Preinitiation Complex Assembly (GO:0051123)	0.0505236	0.4156036	0	0	2.636637872	7.871195488	*GTF2A1;MED21;TAF8;MED6;MED28*
Biological Process	Peptide Metabolic Process (GO:0006518)	0.0505236	0.4156036	0	0	2.636637872	7.871195488	*CARNMT1;ERAP1;LNPEP;IDE;SPPL3*
Biological Process	Vesicle Cytoskeletal Trafficking (GO:0099518)	0.0506757	0.4156036	0	0	3.071827614	9.161137363	*SYT4;FBXW11;MYO15A;MYO5A*
Biological Process	Nuclear-Transcribed mRNA Catabolic Process, Deadenylation-Dependent Decay (GO:0000288)	0.0506757	0.4156036	0	0	3.071827614	9.161137363	*CNOT6;DIS3;DCP1A;DCP2*
Biological Process	Positive Regulation Of Pattern Recognition Receptor Signaling Pathway (GO:0062208)	0.0506757	0.4156036	0	0	3.071827614	9.161137363	*USP15;DDX3X;PUM1;PUM2*
Biological Process	Negative Regulation Of Cell Motility (GO:2000146)	0.0511922	0.4185433	0	0	1.955492038	5.812052799	*ING2;RBBP4;ZMYND8;ADIPOQ;ARID4A;ARID4B;MIA3;ARPIN;SCAI*
Biological Process	Regulation Of Apoptotic Process (GO:0042981)	0.05194	0.4233508	0	0	1.374533463	4.06541153	*GSK3B;PRKAA1;VCP;RBM25;PRKAA2;DDX3X;PRKDC;DDX20;XIAP;BCL10;CBL;MTDH;MBTD1;ING3;SIX4;CASP3;ZNF268;SOX4;APAF1;API5;SIAH1;SMAD5;SON;TFAP4;STK17B;RASA1;JMY;CTNNB1;SPRY2;PHIP;NAA15;ARHGEF7;TARDBP;RAF1*

Biological Process	Peptidyl-Lysine Modification (GO:0018205)	0.0529065	0.4293787	0	0	2.165868679	6.365986188	*SUMO2;EP300;SIRT5;P3H4;SMC6;PIAS2;SENP1*
Biological Process	Regulation Of Cytoplasmic Translation (GO:2000765)	0.053639	0.4293787	0	0	3.837218569	11.22570494	*DHX36;HNRNPU;CPEB3*
Biological Process	Amyloid Fibril Formation (GO:1990000)	0.053639	0.4293787	0	0	3.837218569	11.22570494	*USP9X;SIAH1;TARDBP*
Biological Process	Positive Regulation Of Cellular Response To Transforming Growth Factor Beta Stimulus (GO:1903846)	0.053639	0.4293787	0	0	3.837218569	11.22570494	*ING2;EP300;RNF111*
Biological Process	Positive Regulation Of Transforming Growth Factor Beta Receptor Signaling Pathway (GO:0030511)	0.053639	0.4293787	0	0	3.837218569	11.22570494	*ING2;EP300;RNF111*
Biological Process	Protein Modification By Small Protein Removal (GO:0070646)	0.0536528	0.4293787	0	0	2.03243587	5.945324724	*USP13;USP8;USP37;USP15;USP9X;USP9Y;JOSD1;SENP1*
Biological Process	Signal Transduction In Response To DNA Damage (GO:0042770)	0.0538142	0.4293787	0	0	2.585798817	7.556269211	*ERCC4;ATRX;FBXO6;INTS7;CLOCK*
Biological Process	Regulation Of Glycolytic Process (GO:0006110)	0.0547684	0.4343754	0	0	2.986343886	8.674261641	*PRKAA1;PRKAA2;EP300;SLC4A4*
Biological Process	Regulation Of Microtubule Cytoskeleton Organization (GO:0070507)	0.0547684	0.4343754	0	0	2.986343886	8.674261641	*GSK3B;PRKAA1;PRKAA2;CLASP2*
Biological Process	Regulation Of Ras Protein Signal Transduction (GO:0046578)	0.0558831	0.4394093	0	0	2.306122449	6.651995107	*KBTBD6;DENND4C;SPRY2;SPRY1;RAF1;RASGRP1*
Biological Process	Protein Modification Process (GO:0036211)	0.0570975	0.4394093	0	0	1.361912226	3.899148583	*GSK3B;PRKAA1;PRKAA2;PRKDC;WDR45B;PPM1H;PPM1G;CAMKK2;PPP6C;FUT9;ST3GAL5;MAPK1;BTRC;PRKACB;EPHB1;MARK3;MAP4K3;DUSP5;FBXW11;PDPK1;DYRK1A;PPP4R3B;ATG14;BAZ1B;HIPK1;PPM1A;FER;PDP2;CREB1;PEAK1;STK17B;TAF8;PKN2;ALG10B*
Biological Process	Regulation Of Leukocyte Cell-Cell Adhesion (GO:1903037)	0.0573936	0.4394093	0	0	5.964407304	17.04521821	*FUT9;MIA3*
Biological Process	Regulation Of Protein K63-linked Ubiquitination (GO:1900044)	0.0573936	0.4394093	0	0	5.964407304	17.04521821	*DDX3X;PLAA*
Biological Process	Central Nervous System Projection Neuron Axonogenesis (GO:0021952)	0.0573936	0.4394093	0	0	5.964407304	17.04521821	*NFIB;EPHB1*
Biological Process	Telomere Maintenance In Response To DNA Damage (GO:0043247)	0.0573936	0.4394093	0	0	5.964407304	17.04521821	*RTEL1;TERF2IP*
Biological Process	Ganglion Development (GO:0061548)	0.0573936	0.4394093	0	0	5.964407304	17.04521821	*FZD3;CTNNB1*
Biological Process	Methylguanosine-Cap Decapping (GO:0110156)	0.0573936	0.4394093	0	0	5.964407304	17.04521821	*DCP1A;DCP2*
Biological Process	Mitochondrial Genome Maintenance (GO:0000002)	0.0573936	0.4394093	0	0	5.964407304	17.04521821	*MEF2A;SLC25A36*
Biological Process	Negative Regulation Of Cellular Response To Vascular Endothelial Growth Factor Stimulus (GO:1902548)	0.0573936	0.4394093	0	0	5.964407304	17.04521821	*SPRY2;PIK3CB*
Biological Process	Positive Regulation Of Protein Polyubiquitination (GO:1902916)	0.0573936	0.4394093	0	0	5.964407304	17.04521821	*DDX3X;XIAP*
Biological Process	Post-Transcriptional Gene Silencing (GO:0016441)	0.0573936	0.4394093	0	0	5.964407304	17.04521821	*CELF1;PUM1*
Biological Process	Cellular Response To Transforming Growth Factor Beta Stimulus (GO:0071560)	0.0580233	0.44295	0	0	2.11697685	6.026844817	*USP15;USP9X;USP9Y;STAT3;SMAD5;CRKL;APPL1*
Biological Process	Actomyosin Structure Organization (GO:0031032)	0.0588504	0.4466899	0	0	2.273523494	6.440337951	*EPB41L4B;EPB41L5;SIX4;CUL3;SORBS1;TTN*
Biological Process	Negative Regulation Of Blood Vessel Morphogenesis (GO:2000181)	0.0588504	0.4466899	0	0	2.273523494	6.440337951	*ATF2;PDE3B;FOXJ2;CTNNB1;SPRY2;GTF2I*
Biological Process	Myotube Differentiation (GO:0014902)	0.0593803	0.4494237	0	0	3.662609357	10.34245272	*MYEF2;SIX4;ADGRB3*
Biological Process	Protein Localization To Organelle (GO:0033365)	0.0595868	0.4497022	0	0	1.893987342	5.341652179	*CEP68;TTC26;PRKAA1;DDX3X;PRKAA2;SSB;SIX4;TTC21B;DCP1A*

Biological Process	Negative Regulation Of Signal Transduction (GO:0009968)	0.0600073	0.4508468	0	0	1.606551841	4.519693901	*VCP;ADIPOQ;RPGRIP1L;PIK3CB;SMAD5;HEY1;SPRY2;SPRY1;CD47;BTRC;VGLL4;LGR4;APPL1;GRINA;NCK1*
Biological Process	Positive Regulation Of Cellular Process (GO:0048522)	0.0606375	0.4508468	0	0	1.390148944	3.896366771	*SLC35F6;GSK3B;PRKAA1;PRKAA2;DDX3X;CUL3;LRP6;CRKL;EPB41L4B;BCL7A;SPOCK2;DHX36;ZNF703;SSR1;STX3;TNPO1;ZNF268;SOX4;OSR2;PBRM1;EGR4;ARID1A;HCFC1;CNOT6;EPGN;FER;CTNNB1;PHIP;CD47*
Biological Process	DNA-templated Transcription Initiation (GO:0006352)	0.0607596	0.4508468	0	0	2.48976949	6.973419614	*RRN3;NCOA6;TAF8;ELOA;PPARGC1A*
Biological Process	Transcription Preinitiation Complex Assembly (GO:0070897)	0.0607596	0.4508468	0	0	2.48976949	6.973419614	*GTF2A1;MED21;TAF8;MED6;MED28*
Biological Process	Negative Regulation Of Protein-Containing Complex Assembly (GO:0031333)	0.0607596	0.4508468	0	0	2.48976949	6.973419614	*GSK3B;PPM1A;DDX3X;EP300;RAF1*
Biological Process	Positive Regulation Of Protein Binding (GO:0032092)	0.0607596	0.4508468	0	0	2.48976949	6.973419614	*GSK3B;USP9X;IDE;SPPL3;MARK3*
Biological Process	RNA Splicing, Via Transesterification Reactions With Bulged Adenosine As Nucleophile (GO:0000377)	0.0613222	0.4537497	0	0	1.754458734	4.897771604	*DHX8;U2AF2;SRSF1;TRA2A;HNRNPA2B1;SRSF2;HNRNPU;CWC27;SRSF10;RBMX;SRSF8*
Biological Process	Proteolysis Involved In Protein Catabolic Process (GO:0051603)	0.0619101	0.4543367	0	0	2.241830065	6.236932868	*ATXN3;UBE2W;VCP;PDCD6IP;USP9X;IDE*
Biological Process	Regulation Of TOR Signaling (GO:0032006)	0.0619101	0.4543367	0	0	2.241830065	6.236932868	*PRKAA1;PRKAA2;TMEM127;RRAGD;GPAT3;FNIP1*
Biological Process	Phosphorylation (GO:0016310)	0.061916	0.4543367	0	0	1.461550376	4.065998382	*GSK3B;PRKAA1;PRKAA2;PRKDC;PDPK1;STAT3;DYRK1A;PIK3CB;ATG14;HIPK1;CAMKK2;FER;CREB1;PEAK1;STK17B;TAF8;MAPK1;PKN2;PRKACB;EPHB1;MARK3;MAP4K3*
Biological Process	Protein Phosphorylation (GO:0006468)	0.0622384	0.4554405	0	0	1.424081787	3.954366827	*ATF2;GSK3B;PRKAA1;USP15;PRKAA2;PRKDC;CAMKK2;RELN;EEF2K;MAPK1;RIOK2;PRKACB;EPHB1;MARK3;MAP4K3;PDPK1;DYRK1A;ATG14;HIPK1;FER;CREB1;PEAK1;STK17B;TAF8;PKN2*
Biological Process	Regulation Of Cell Population Proliferation (GO:0042127)	0.0627525	0.4579373	0	0	1.33741889	3.70272118	*SLC35F6;PRKDC;CELF1;CUL3;HP1BP3;XIAP;CRKL;ZNF609;RBBP4;BCL7A;SIX4;TMEM127;ZNF703;SSR1;STX3;ZNF268;SOX4;OSR2;PBRM1;RBFOX2;EGR4;AVPR1B;TFAP2E;STAT3;OPRM1;CNOT6;EPGN;FER;TFAP4;AGO3;PHF14;CTNNB1;PHIP;CD47;SOS1;RAF1*
Biological Process	DNA Integrity Checkpoint Signaling (GO:0031570)	0.0634716	0.460458	0	0	2.828873861	7.799667418	*ERCC4;FBXO6;INTS7;CLOCK*
Biological Process	Positive Regulation Of Intracellular Signal Transduction (GO:1902533)	0.063621	0.460458	0	0	1.410041986	3.884400259	*NFAT5;DDX3X;BCL10;PIK3CB;CBL;MTDH;RELN;TIFA;DHX36;EP300;FNIP1;SH2B1;TMED4;AVPR1B;ADIPOQ;SIAH1;ZDHHC13;ZDHHC17;MID2;PPM1A;TRAF6;RRAGD;TERF2IP;SPRY2;RAF1;TMEM9B*
Biological Process	Histone Acetylation (GO:0016573)	0.0644141	0.460458	0	0	2.444373808	6.703505148	*MBTD1;DR1;ING3;WDR5;EP300*
Biological Process	Positive Regulation Of Catabolic Process (GO:0009896)	0.0650622	0.460458	0	0	2.21100495	6.041376884	*GSK3B;VCP;SMURF1;ASB11;IDE;VGLL4*
Biological Process	DNA-templated Transcription Elongation (GO:0006354)	0.0653968	0.460458	0	0	3.503183555	9.554169874	*PBRM1;SUPT16H;SUPT5H*
Biological Process	Fc Receptor Signaling Pathway (GO:0038093)	0.0653968	0.460458	0	0	3.503183555	9.554169874	*FER;TRAF6;SOS1*
Biological Process	Regulation Of Nucleotide-Excision Repair (GO:2000819)	0.0653968	0.460458	0	0	3.503183555	9.554169874	*PBRM1;BCL7A;ARID1A*
Biological Process	Histone H3-K4 Methylation (GO:0051568)	0.0653968	0.460458	0	0	3.503183555	9.554169874	*NCOA6;WDR5;HCFC1*
Biological Process	Mitotic G1 DNA Damage Checkpoint Signaling (GO:0031571)	0.0653968	0.460458	0	0	3.503183555	9.554169874	*PRKDC;WAC;FBXO31*
Biological Process	Regulation Of Protein-Containing Complex Assembly (GO:0043254)	0.0656846	0.460458	0	0	1.940378581	5.28343916	*ATF7IP;GSK3B;VCP;DDX3X;EP300;RAF1;FNIP1;HCFC1*
Biological Process	Regulation Of Hematopoietic Stem Cell Differentiation (GO:1902036)	0.0672696	0.460458	0	0	5.367688022	14.48763891	*PRKDC;N4BP2L2*
Biological Process	Atrial Cardiac Muscle Cell Action Potential (GO:0086014)	0.0672696	0.460458	0	0	5.367688022	14.48763891	*CACNA1C;KCNJ3*
Biological Process	Cristae Formation (GO:0042407)	0.0672696	0.460458	0	0	5.367688022	14.48763891	*CHCHD3;IMMT*
Biological Process	Ribosome Disassembly (GO:0032790)	0.0672696	0.460458	0	0	5.367688022	14.48763891	*MRRF;PELO*
Biological Process	Spinal Cord Development (GO:0021510)	0.0672696	0.460458	0	0	5.367688022	14.48763891	*RELN;SOX4*
Biological Process	Exonucleolytic Catabolism Of Deadenylated mRNA (GO:0043928)	0.0672696	0.460458	0	0	5.367688022	14.48763891	*DIS3;DCP2*
Biological Process	tRNA 5'-Leader Removal (GO:0001682)	0.0672696	0.460458	0	0	5.367688022	14.48763891	*SSB;RPP14*

Biological Process	Microtubule Anchoring At Microtubule Organizing Center (GO:0072393)	0.0672696	0.460458	0	0	5.367688022	14.48763891	*KIF3A;HOOK3*
Biological Process	Negative Regulation By Host Of Viral Transcription (GO:0043922)	0.0672696	0.460458	0	0	5.367688022	14.48763891	*TFAP4;TARDBP*
Biological Process	Negative Regulation Of Hippo Signaling (GO:0035331)	0.0672696	0.460458	0	0	5.367688022	14.48763891	*MARK3;VGLL4*
Biological Process	Positive Regulation Of Alcohol Biosynthetic Process (GO:1902932)	0.0672696	0.460458	0	0	5.367688022	14.48763891	*PRKAA1;AVPR1B*
Biological Process	Positive Regulation Of Neuron Migration (GO:2001224)	0.0672696	0.460458	0	0	5.367688022	14.48763891	*ZNF609;PLAA*
Biological Process	Positive Regulation Of Purine Nucleotide Biosynthetic Process (GO:1900373)	0.0672696	0.460458	0	0	5.367688022	14.48763891	*VCP;PPARGC1A*
Biological Process	Primary miRNA Processing (GO:0031053)	0.0672696	0.460458	0	0	5.367688022	14.48763891	*PUS10;HNRNPA2B1*
Biological Process	Regulation Of Chromosome Organization (GO:0033044)	0.0680789	0.4633258	0	0	2.756195387	7.406139889	*YY1;INO80D;TERF2IP;SMC6*
Biological Process	Positive Regulation Of Organelle Assembly (GO:1902117)	0.0681892	0.4633258	0	0	2.400599401	6.44673383	*GSK3B;CNOT6;PDCD6IP;CEP120;RAB3GAP2*
Biological Process	Regulation Of Protein Localization To Plasma Membrane (GO:1903076)	0.0683066	0.4633258	0	0	2.181012946	5.85329125	*PDPK1;PPFIA1;ZDHHC7;STX3;SORBS1;APPL1*
Biological Process	Regulation Of Macromolecule Metabolic Process (GO:0060255)	0.0683882	0.4633258	0	0	1.772424018	4.754624317	*RASA1;TMPRSS4;CHD6;MTMR9;STX3;AFF3;TARDBP;AFF4;CGGBP1;AFF2*
Biological Process	Response To UV (GO:0009411)	0.0691444	0.467254	0	0	2.025501817	5.411245314	*YY1;ERCC4;DHX36;CIRBP;EP300;MAP3K4;MAP4K3*
Biological Process	Negative Regulation Of Cell Migration (GO:0030336)	0.0706374	0.4761287	0	0	1.760747491	4.666324096	*ING2;RBBP4;ZMYND8;ADIPOQ;ARID4A;ARID4B;MIA3;ARPIN;SCAI;CLASP2*
Biological Process	Positive Regulation Of TORC1 Signaling (GO:1904263)	0.0716807	0.4819347	0	0	3.357043236	8.847599173	*RRAGD;WAC;GPR137C*
Biological Process	Positive Regulation Of Nucleocytoplasmic Transport (GO:0046824)	0.0728541	0.4885834	0	0	2.687150838	7.038445652	*GSK3B;KHDRBS1;CPSF6;EP300*
Biological Process	Activation Of GTPase Activity (GO:0090630)	0.0751508	0.502278	0	0	1.982652986	5.13161936	*TBC1D1;RALGAPA2;TBC1D30;ARHGAP29;CXCL13;RASGRP1;EVI5*
Biological Process	Negative Regulation Of Multicellular Organismal Process (GO:0051241)	0.0752753	0.502278	0	0	1.607814386	4.158777015	*GSK3B;ADIPOQ;ARID4A;LNPEP;ARID4B;HMGCR;ING2;RBBP4;NFIB;PHF14;SPRY2;SPRY1;LGR4*
Biological Process	Regulation Of Cellular Catabolic Process (GO:0031329)	0.0760989	0.5028781	0	0	2.317578973	5.969438233	*USP13;WAC;EP300;RAB39B;TPCN2*
Biological Process	Regulation Of I-kappaB kinase/NF-kappaB Signaling (GO:0043122)	0.0772645	0.5028781	0	0	1.600388806	4.097829399	*PDPK1;ZDHHC13;BCL10;ZDHHC17;MID2;MTDH;PPM1A;TRAF6;DHX36;TIFA;TERF2IP;TMEM9B;TMED4*
Biological Process	Regulation Of Gastrulation (GO:0010470)	0.0776565	0.5028781	0	0	4.879463155	12.46927407	*POGLUT1;CLASP2*
Biological Process	Spliceosomal tri-snRNP Complex Assembly (GO:0000244)	0.0776565	0.5028781	0	0	4.879463155	12.46927407	*DDX20;SRSF10*
Biological Process	Interleukin-6-Mediated Signaling Pathway (GO:0070102)	0.0776565	0.5028781	0	0	4.879463155	12.46927407	*FER;STAT3*
Biological Process	Lipid Oxidation (GO:0034440)	0.0776565	0.5028781	0	0	4.879463155	12.46927407	*ADIPOQ;PPARGC1A*
Biological Process	Negative Regulation Of Fibroblast Growth Factor Receptor Signaling Pathway (GO:0040037)	0.0776565	0.5028781	0	0	4.879463155	12.46927407	*SPRY2;SPRY1*
Biological Process	Positive Regulation Of Double-Strand Break Repair Via Nonhomologous End Joining (GO:2001034)	0.0776565	0.5028781	0	0	4.879463155	12.46927407	*PRKDC;KMT5B*
Biological Process	Positive Regulation Of Protein Deacetylation (GO:0090312)	0.0776565	0.5028781	0	0	4.879463155	12.46927407	*ING2;DYRK1A*
Biological Process	Peptidyl-Serine Modification (GO:0018209)	0.077663	0.5028781	0	0	1.726616107	4.412154671	*GSK3B;POGLUT1;PRKDC;PDPK1;DYRK1A;MAPK1;PKN2;HIPK1;PRKACB;MARK3*
Biological Process	Regulation Of Protein Modification Process (GO:0031399)	0.0777948	0.5028781	0	0	2.621474315	6.694408351	*FER;PHIP;FNIP1;ATG14*

Biological Process	Regulation Of Telomerase Activity (GO:0051972)	0.0777948	0.5028781	0	0	2.621474315	6.694408351	*ERCC4;MAPK1;CTNNB1;MAP3K4*
Biological Process	Regulation Of Chromosome Segregation (GO:0051983)	0.0782238	0.5028781	0	0	3.222594142	8.211755735	*SMC6;PUM1;PUM2*
Biological Process	Cell-Substrate Junction Assembly (GO:0007044)	0.0782238	0.5028781	0	0	3.222594142	8.211755735	*PEAK1;SORBS1;ARHGEF7*
Biological Process	Epigenetic Regulation Of Gene Expression (GO:0040029)	0.0785925	0.5028781	0	0	2.095711012	5.330396807	*ATF2;DNMT3A;WDR5;RNF8;EP300;CGGBP1*
Biological Process	Positive Regulation Of Proteasomal Ubiquitin-Dependent Protein Catabolic Process (GO:0032436)	0.0785925	0.5028781	0	0	2.095711012	5.330396807	*GSK3B;NUB1;VCP;SUMO2;RNF217;ARIH1*
Biological Process	Regulation Of NIK/NF-kappaB Signaling (GO:1901222)	0.0785925	0.5028781	0	0	2.095711012	5.330396807	*PPM1A;NFAT5;DDX3X;TRAF6;EP300;TERF2IP*
Biological Process	Endocytic Recycling (GO:0032456)	0.0802318	0.5086981	0	0	2.278179448	5.747471481	*DENND1B;SNX27;RAB14;VPS50;VAMP4*
Biological Process	Positive Regulation Of Macroautophagy (GO:0016239)	0.0802318	0.5086981	0	0	2.278179448	5.747471481	*PRKAA2;WAC;GNAI3;RAB3GAP2;SUPT5H*
Biological Process	Positive Regulation Of Type I Interferon Production (GO:0032481)	0.0802318	0.5086981	0	0	2.278179448	5.747471481	*DDX3X;POLR3C;DHX36;POLR3F;XIAP*
Biological Process	Intracellular Protein Transport (GO:0006886)	0.0802702	0.5086981	0	0	1.489553121	3.757184284	*SMURF1;CSE1L;SURF4;STAT3;RABL2B;NUP153;ARL17A;SNX27;RAB14;RAB3GAP2;PHIP;STX3;TNPO1;ARL5A;TRIM23;APPL1;TMED4*
Biological Process	Positive Regulation Of GTPase Activity (GO:0043547)	0.0813432	0.5142677	0	0	1.585739246	3.978743184	*GSK3B;RALGAPA2;ARHGAP29;CXCL13;RASGRP1;TBC1D1;TBC1D30;ARHGEF7;SOS1;RAPGEF6;EVI5;RALGPS2;RGS7*
Biological Process	Positive Regulation Of Cellular Catabolic Process (GO:0031331)	0.0815457	0.5143205	0	0	1.768733253	4.433491696	*RBM27;GSK3B;PRKAA1;PRKAA2;PIK3CB;SLC4A4;MID2;TRIM23;MTDH*
Biological Process	Response To Cytokine (GO:0034097)	0.0821618	0.5160175	0	0	1.840295784	4.599019115	*NFAT5;NUB1;SLC22A5;CASP3;TRAF6;ADIPOQ;AFF3;MAP4K3*
Biological Process	Regulation Of Cysteine-Type Endopeptidase Activity Involved In Apoptotic Process (GO:0043281)	0.0822044	0.5160175	0	0	2.068735186	5.168831662	*DDX3X;DNAJB6;XIAP;BCL10;RAF1;FNIP1*
Biological Process	Antiviral Innate Immune Response (GO:0140374)	0.0828985	0.5179201	0	0	2.558925246	6.37207795	*UBE2W;TRIM7;TRAF6;NCK1*
Biological Process	Protein Sumoylation (GO:0016925)	0.0828985	0.5179201	0	0	2.558925246	6.37207795	*SUMO2;SMC6;PIAS2;SENP1*
Biological Process	Dephosphorylation (GO:0016311)	0.0842512	0.5198424	0	0	1.755824619	4.343826158	*PPP6C;DUSP5;PPM1A;PDP2;FBXW11;PPM1H;PPP4R3B;BTRC;PPM1G*
Biological Process	RNA Export From Nucleus (GO:0006405)	0.0844821	0.5198424	0	0	2.24009324	5.535752312	*FYTTD1;NXF1;SARNP;HNRNPA2B1;NUP153*
Biological Process	Canonical Wnt Signaling Pathway (GO:0060070)	0.0844821	0.5198424	0	0	2.24009324	5.535752312	*GSK3B;FZD3;SIAH1;CTNNB1;LRP6*
Biological Process	Regulation Of Cell-Matrix Adhesion (GO:0001952)	0.0844821	0.5198424	0	0	2.24009324	5.535752312	*GSK3B;PEAK1;RASA1;CASK;PIK3CB*
Biological Process	Intrinsic Apoptotic Signaling Pathway (GO:0097193)	0.0847168	0.5198424	0	0	1.921658986	4.743501546	*DDX3X;APAF1;PRKDC;CUL3;CASP3;CUL2;JMY*
Biological Process	Regulation Of Developmental Growth (GO:0048638)	0.0850171	0.5198424	0	0	3.098487287	7.637468773	*GSK3B;MBD5;SIX4*
Biological Process	RNA-mediated Gene Silencing (GO:0031047)	0.0850171	0.5198424	0	0	3.098487287	7.637468773	*AGO3;CELF1;TNRC6B*
Biological Process	Regulation Of Neuron Migration (GO:2001222)	0.0850171	0.5198424	0	0	3.098487287	7.637468773	*ZNF609;RELN;PLAA*
Biological Process	Lipid Droplet Organization (GO:0034389)	0.0850171	0.5198424	0	0	3.098487287	7.637468773	*PRKAA1;PRKAA2;CDS2*
Biological Process	Calcium-Mediated Signaling (GO:0019722)	0.0859072	0.5198424	0	0	2.042442293	5.013150427	*PRKAA1;PDPK1;CACNA1C;PPP1R9A;TPCN2;CAMKK2*
Biological Process	Macromolecule Catabolic Process (GO:0009057)	0.0859072	0.5198424	0	0	2.042442293	5.013150427	*DFFB;DIS3;SMURF1;CASP3;SIAH1;IDE*
Biological Process	Nuclear-Transcribed mRNA Catabolic Process (GO:0000956)	0.0859072	0.5198424	0	0	2.042442293	5.013150427	*CNOT6;XRN1;ERI1;DCP1A;PELO;DCP2*
Biological Process	Regulation Of Anatomical Structure Morphogenesis (GO:0022603)	0.0881505	0.5198424	0	0	1.809177604	4.393967578	*GSK3B;POGLUT1;ZMYM3;ADGRB3;CTNNB1;PHIP;CXCL13;CLASP2*
Biological Process	Regulation Of Signal Transduction By P53 Class Mediator (GO:1901796)	0.0881624	0.5198424	0	0	2.499285436	6.069702758	*KAT6A;EP300;RPF2;SOX4*
Biological Process	COPII-coated Vesicle Cargo Loading (GO:0090110)	0.0885047	0.5198424	0	0	4.472609099	10.84473444	*MIA3;SEC23B*

Biological Process	Contractile Actin Filament Bundle Assembly (GO:0030038)	0.0885047	0.5198424	0	0	4.472609099	10.84473444	*CUL3;SORBS1*
Biological Process	Response To Muscle Stretch (GO:0035994)	0.0885047	0.5198424	0	0	4.472609099	10.84473444	*CTNNB1;TTN*
Biological Process	Stress Fiber Assembly (GO:0043149)	0.0885047	0.5198424	0	0	4.472609099	10.84473444	*CUL3;SORBS1*
Biological Process	tRNA 5'-End Processing (GO:0099116)	0.0885047	0.5198424	0	0	4.472609099	10.84473444	*SSB;RPP14*
Biological Process	Telomere Capping (GO:0016233)	0.0885047	0.5198424	0	0	4.472609099	10.84473444	*PRKDC;TERF2IP*
Biological Process	Negative Regulation Of Transcription Elongation By RNA Polymerase II (GO:0034244)	0.0885047	0.5198424	0	0	4.472609099	10.84473444	*RNF8;HNRNPU*
Biological Process	Neuron Cell-Cell Adhesion (GO:0007158)	0.0885047	0.5198424	0	0	4.472609099	10.84473444	*CNTN4;ASTN2*
Biological Process	Positive Regulation Of Cytoplasmic Translation (GO:2000767)	0.0885047	0.5198424	0	0	4.472609099	10.84473444	*DHX36;HNRNPU*
Biological Process	Positive Regulation Of Histone Acetylation (GO:0035066)	0.0885047	0.5198424	0	0	4.472609099	10.84473444	*BAZ1B;PPARGC1A*
Biological Process	Positive Regulation Of Mitochondrial Translation (GO:0070131)	0.0885047	0.5198424	0	0	4.472609099	10.84473444	*MRPS27;UQCC2*
Biological Process	Positive Regulation Of Telomere Maintenance In Response To DNA Damage (GO:1904507)	0.0885047	0.5198424	0	0	4.472609099	10.84473444	*YY1;INO80D*
Biological Process	Regulation Of ATP Biosynthetic Process (GO:2001169)	0.0885047	0.5198424	0	0	4.472609099	10.84473444	*VCP;PPARGC1A*
Biological Process	Negative Regulation Of Protein Phosphorylation (GO:0001933)	0.0898219	0.5264121	0	0	1.730560579	4.170523413	*PRKDC;PDPK1;TERF2IP;SPRY2;ATG14;TARDBP;CRKL;NCK1;LRP6*
Biological Process	DNA Geometric Change (GO:0032392)	0.0920518	0.5382895	0	0	2.983573532	7.117028796	*RTEL1;DHX36;HNRNPA2B1*
Biological Process	Regulation Of Neurogenesis (GO:0050767)	0.0933303	0.5433672	0	0	2.167606587	5.140717577	*HES7;HEY1;CTNNB1;OPRM1;HOOK3*
Biological Process	tRNA Modification (GO:0006400)	0.0933303	0.5433672	0	0	2.167606587	5.140717577	*DTWD2;QTRT2;PUS10;SSB;TRDMT1*
Biological Process	Regulation Of Tumor Necrosis Factor-Mediated Signaling Pathway (GO:0010803)	0.0935833	0.5436453	0	0	2.442356526	5.785705447	*ADIPOQ;SPATA2;XIAP;HIPK1*
Biological Process	Negative Regulation Of Programmed Cell Death (GO:0043069)	0.0961653	0.5471729	0	0	1.416453212	3.316889804	*GSK3B;PRKAA1;DDX3X;PRKAA2;PRKDC;API5;XIAP;CBL;SMAD5;MTDH;SON;SIX4;RASA1;CTNNB1;SPRY2;PHIP;NAA15;RAF1;ZNF268*
Biological Process	DNA Metabolic Process (GO:0006259)	0.0984843	0.5471729	0	0	1.481334269	3.433522189	*DFFB;RTEL1;VCP;INO80D;PRKDC;ATRX;TSN;YY1;ORC1;ERCC4;PSME4;SMARCAD1;TERF2IP;FBXO6;KMT5B*
Biological Process	Positive Regulation Of NF-kappaB Transcription Factor Activity (GO:0051092)	0.098576	0.5471729	0	0	1.693989555	3.92485133	*FER;TRAF6;STAT3;EP300;TERF2IP;BCL10;CLOCK;MID2;MTDH*
Biological Process	Regulation Of Protein Phosphorylation (GO:0001932)	0.0989317	0.5471729	0	0	1.50336896	3.477781925	*PRKDC;ADIPOQ;ATG14;RASGRP1;CRKL;CAMKK2;LRP6;TTN;FER;RELN;TERF2IP;PHIP;TARDBP;FNIP1*
Biological Process	Negative Regulation Of DNA Metabolic Process (GO:0051053)	0.0991582	0.5471729	0	0	2.38795779	5.518662411	*RTEL1;ERCC4;ADIPOQ;TERF2IP*
Biological Process	Nuclear Export (GO:0051168)	0.0991582	0.5471729	0	0	2.38795779	5.518662411	*ATXN1;SMURF1;CSE1L;NUP153*
Biological Process	Regulation Of Centrosome Duplication (GO:0010824)	0.0993183	0.5471729	0	0	2.876867902	6.643913181	*PDCD6IP;CEP120;CEP76*
Biological Process	Regulation Of Extrinsic Apoptotic Signaling Pathway Via Death Domain Receptors (GO:1902041)	0.0993183	0.5471729	0	0	2.876867902	6.643913181	*GSK3B;DDX3X;FEM1B*
Biological Process	Regulation Of Long-Term Synaptic Potentiation (GO:1900271)	0.0993183	0.5471729	0	0	2.876867902	6.643913181	*GSK3B;YTHDF1;RELN*
Biological Process	Negative Regulation Of Cell-Matrix Adhesion (GO:0001953)	0.0993183	0.5471729	0	0	2.876867902	6.643913181	*RASA1;CASK;CLASP2*
Biological Process	Organic Cation Transport (GO:0015695)	0.0993183	0.5471729	0	0	2.876867902	6.643913181	*SLC7A6;SLC22A5;SLC19A2*
Biological Process	3'-UTR-mediated mRNA Destabilization (GO:0061158)	0.0997677	0.5471729	0	0	4.128347975	9.515475487	*DHX36;TARDBP*
Biological Process	Regulation Of Clathrin-Dependent Endocytosis (GO:2000369)	0.0997677	0.5471729	0	0	4.128347975	9.515475487	*PIK3CB;SNAP91*

Biological Process	DNA Alkylation (GO:0006305)	0.0997677	0.5471729	0	0	4.128347975	9.515475487	*ATRX;DNMT3A*
Biological Process	Golgi To Endosome Transport (GO:0006895)	0.0997677	0.5471729	0	0	4.128347975	9.515475487	*RAB14;VPS13A*
Biological Process	N-terminal Protein Amino Acid Acetylation (GO:0006474)	0.0997677	0.5471729	0	0	4.128347975	9.515475487	*EP300;NAA15*
Biological Process	Regulation Of miRNA-mediated Gene Silencing (GO:0060964)	0.0997677	0.5471729	0	0	4.128347975	9.515475487	*PUM1;PUM2*
Biological Process	Amyloid-Beta Metabolic Process (GO:0050435)	0.0997677	0.5471729	0	0	4.128347975	9.515475487	*DYRK1A;IDE*
Biological Process	Histone H4-K5 Acetylation (GO:0043981)	0.0997677	0.5471729	0	0	4.128347975	9.515475487	*PHF20;WDR5*
Biological Process	Histone H4-K8 Acetylation (GO:0043982)	0.0997677	0.5471729	0	0	4.128347975	9.515475487	*PHF20;WDR5*
Biological Process	Negative Regulation Of Epithelial Cell Differentiation (GO:0030857)	0.0997677	0.5471729	0	0	4.128347975	9.515475487	*SPRY2;SPRY1*
Biological Process	Negative Regulation Of Immune Effector Process (GO:0002698)	0.0997677	0.5471729	0	0	4.128347975	9.515475487	*FER;CD47*
Biological Process	Negative Regulation Of Phagocytosis (GO:0050765)	0.0997677	0.5471729	0	0	4.128347975	9.515475487	*CD47;APPL1*
Biological Process	Positive Regulation Of Transcription By RNA Polymerase III (GO:0045945)	0.0997677	0.5471729	0	0	4.128347975	9.515475487	*ELL;BAZ1B*
Biological Process	Protein Localization To Chromosome, Telomeric Region (GO:0070198)	0.0997677	0.5471729	0	0	4.128347975	9.515475487	*ATRX;TERF2IP*
Biological Process	Receptor Catabolic Process (GO:0032801)	0.0997677	0.5471729	0	0	4.128347975	9.515475487	*ZNRF3;SMURF1*
Biological Process	Regulation Of Rac Protein Signal Transduction (GO:0035020)	0.0997677	0.5471729	0	0	4.128347975	9.515475487	*KBTBD6;PIK3CB*
Biological Process	Protein Transport (GO:0015031)	0.1018683	0.5575398	0	0	1.452632384	3.317919936	*SURF4;RABL2B;MIA3;SCAMP1;ZDHHC17;LRP1B;ARL17A;SNX27;RAB14;KIF3A;RAB3GAP2;PHIP;STX3;ARL5A;TRIM23;TMED4*
Biological Process	Positive Regulation Of Hydrolase Activity (GO:0051345)	0.104105	0.5670134	0	0	1.621754624	3.668985271	*GSK3B;TBC1D30;MTMR9;ARHGEF7;SOS1;RASGRP1;EVI5;RAPGEF6;RGS7;RALGPS2*
Biological Process	Regulation Of Interferon-Beta Production (GO:0032648)	0.1048836	0.5670134	0	0	2.335924217	5.26728596	*YY1;DDX3X;POLR3C;POLR3F*
Biological Process	Ribosome Assembly (GO:0042255)	0.1048836	0.5670134	0	0	2.335924217	5.26728596	*DDX3X;MRPS11;RPF2;MDN1*
Biological Process	tRNA Processing (GO:0008033)	0.1048836	0.5670134	0	0	2.335924217	5.26728596	*DTWD2;SSB;TSEN2;TRDMT1*
Biological Process	Intracellular Iron Ion Homeostasis (GO:0006879)	0.1048836	0.5670134	0	0	2.335924217	5.26728596	*SLC22A17;ABCB7;SLC11A2;FBXL5*
Biological Process	Regulation Of TORC1 Signaling (GO:1903432)	0.1048836	0.5670134	0	0	2.335924217	5.26728596	*BMT2;RRAGD;WAC;GPR137C*
Biological Process	Double-Strand Break Repair Via Homologous Recombination (GO:0000724)	0.1058293	0.5709611	0	0	1.810227641	4.065640229	*YY1;MBTD1;WDR48;ERCC4;ZMYND8;ATRX;SMC6*
Biological Process	Positive Regulation Of Programmed Cell Death (GO:0043068)	0.1061874	0.5717285	0	0	1.50968151	3.385536009	*DDX3X;APAF1;SIAH1;DDX20;BCL10;TFAP4;STK17B;CASP3;JMY;CTNNB1;ARHGEF7;ZNF268;SOX4*
Biological Process	Mitochondrial Membrane Organization (GO:0007006)	0.106807	0.5727362	0	0	2.777521281	6.212570667	*CHCHD3;MTX3;IMMT*
Biological Process	Positive Regulation Of Telomere Maintenance Via Telomerase (GO:0032212)	0.106807	0.5727362	0	0	2.777521281	6.212570667	*MAPK1;CTNNB1;MAP3K4*
Biological Process	Protein K48-linked Ubiquitination (GO:0070936)	0.1074504	0.5738632	0	0	2.067240452	4.611446758	*KBTBD6;UBE2D4;CUL3;RNF8;UBE3A*
Biological Process	Regulation Of G Protein-Coupled Receptor Signaling Pathway (GO:0008277)	0.1074504	0.5738632	0	0	2.067240452	4.611446758	*KCTD8;GNG4;ZDHHC7;RPGRIP1L;RGS7*
Biological Process	Positive Regulation Of Apoptotic Process (GO:0043065)	0.1104775	0.5775006	0	0	1.47361898	3.246298839	*DDX3X;APAF1;SIAH1;ADIPOQ;DDX20;BCL10;TFAP4;STK17B;CASP3;JMY;CTNNB1;ARHGEF7;ZNF268;SOX4*
Biological Process	Positive Regulation Of DNA Recombination (GO:0045911)	0.1107558	0.5775006	0	0	2.286104838	5.030408883	*MBTD1;WDR48;ING3;KMT5B*
Biological Process	DNA Repair-Dependent Chromatin Remodeling (GO:0140861)	0.1114016	0.5775006	0	0	3.833267012	8.412540272	*RNF8;TRIP12*

Biological Process	Alternative mRNA Splicing, Via Spliceosome (GO:0000380)	0.1114016	0.5775006	0	0	3.833267012	8.412540272	*SRSF1;CELF4*
Biological Process	Regulation Of Nucleocytoplasmic Transport (GO:0046822)	0.1114016	0.5775006	0	0	3.833267012	8.412540272	*GSK3B;KHDRBS1*
Biological Process	Regulation Of Stem Cell Proliferation (GO:0072091)	0.1114016	0.5775006	0	0	3.833267012	8.412540272	*AGO3;HNRNPU*
Biological Process	Endoplasmic Reticulum Tubular Network Organization (GO:0071786)	0.1114016	0.5775006	0	0	3.833267012	8.412540272	*RTN1;ATL2*
Biological Process	Male Meiosis I (GO:0007141)	0.1114016	0.5775006	0	0	3.833267012	8.412540272	*ING2;FOXJ2*
Biological Process	Nuclear Migration (GO:0007097)	0.1114016	0.5775006	0	0	3.833267012	8.412540272	*FBXW11;HOOK3*
Biological Process	Positive Regulation By Host Of Viral Transcription (GO:0043923)	0.1114016	0.5775006	0	0	3.833267012	8.412540272	*TFAP4;EP300*
Biological Process	Positive Regulation Of Stem Cell Proliferation (GO:2000648)	0.1114016	0.5775006	0	0	3.833267012	8.412540272	*HNRNPU;N4BP2L2*
Biological Process	Protein Kinase A Signaling (GO:0010737)	0.1114016	0.5775006	0	0	3.833267012	8.412540272	*PRKACB;TTN*
Biological Process	Regulation Of DNA Strand Elongation (GO:0060382)	0.1114016	0.5775006	0	0	3.833267012	8.412540272	*YY1;INO80D*
Biological Process	Regulation Of Golgi Organization (GO:1903358)	0.1114016	0.5775006	0	0	3.833267012	8.412540272	*MYO5A;MAPK1*
Biological Process	Regulation Of NMDA Receptor Activity (GO:2000310)	0.1114016	0.5775006	0	0	3.833267012	8.412540272	*RELN;OPRM1*
Biological Process	Regulation Of Cilium Assembly (GO:1902017)	0.1123767	0.5791556	0	0	2.035812672	4.450079652	*GSK3B;TBC1D1;CEP120;TBC1D30;EVI5*
Biological Process	Regulation Of Double-Strand Break Repair Via Homologous Recombination (GO:0010569)	0.1123767	0.5791556	0	0	2.035812672	4.450079652	*MBTD1;RTEL1;WDR48;ING3;TERF2IP*
Biological Process	Negative Regulation Of Cellular Response To Growth Factor Stimulus (GO:0090288)	0.1123767	0.5791556	0	0	2.035812672	4.450079652	*SMURF1;VWC2;CASK;SPRY2;SPRY1*
Biological Process	Positive Regulation Of Protein Modification Process (GO:0031401)	0.1140474	0.5833308	0	0	1.51722766	3.294115203	*RELN;DDX3X;ADIPOQ;DYRK1A;RAB3GAP2;ATG14;FNIP1;ALG10B;SPPL3;RASGRP1;CRKL;CAMKK2*
Biological Process	Positive Regulation Of Protein Catabolic Process (GO:0045732)	0.1143168	0.5833308	0	0	1.875512996	4.067578517	*GSK3B;VCP;TMTC3;ASB11;IDE;VGLL4*
Biological Process	Positive Regulation Of Notch Signaling Pathway (GO:0045747)	0.1145081	0.5833308	0	0	2.684797768	5.818250628	*POGLUT1;STAT3;TSPAN5*
Biological Process	Positive Regulation Of Small Molecule Metabolic Process (GO:0062013)	0.1145081	0.5833308	0	0	2.684797768	5.818250628	*PRKAA1;PRKAA2;SLC4A4*
Biological Process	Positive Regulation Of Telomerase Activity (GO:0051973)	0.1145081	0.5833308	0	0	2.684797768	5.818250628	*MAPK1;CTNNB1;MAP3K4*
Biological Process	Regulation Of G0 To G1 Transition (GO:0070316)	0.1145081	0.5833308	0	0	2.684797768	5.818250628	*PBRM1;BCL7A;ARID1A*
Biological Process	Endosomal Transport (GO:0016197)	0.1159764	0.5896766	0	0	1.583264292	3.410934658	*DENND1B;SNX27;RAB14;VPS50;ITSN1;REPS2;PLEKHA3;TBC1D23;VAMP4;EVI5*
Biological Process	Negative Regulation Of Innate Immune Response (GO:0045824)	0.116771	0.5917499	0	0	2.238361266	4.806971048	*PPP6C;USP15;YTHDF3;PRKDC*
Biological Process	Regulation Of MAP Kinase Activity (GO:0043405)	0.1173531	0.5917499	0	0	1.759198333	3.769202514	*EPGN;ADIPOQ;SPRY2;SPRY1;PIK3CB;SOS1;RASGRP1*
Biological Process	Second-Messenger-Mediated Signaling (GO:0019932)	0.1174099	0.5917499	0	0	2.005323035	4.295571058	*DHX8;PDPK1;PPP1R9A;TPCN2;CAMKK2*
Biological Process	Negative Regulation Of Cell Adhesion (GO:0007162)	0.1174099	0.5917499	0	0	2.005323035	4.295571058	*RASA1;PDE3B;SPOCK1;MIA3;PLXNC1*
Biological Process	Peptidyl-Serine Phosphorylation (GO:0018105)	0.1175011	0.5917499	0	0	1.625265483	3.480193149	*GSK3B;PRKDC;PDPK1;DYRK1A;MAPK1;PKN2;HIPK1;PRKACB;MARK3*
Biological Process	Embryonic Limb Morphogenesis (GO:0030326)	0.1224117	0.5985248	0	0	2.598056418	5.456866708	*OSR2;CTNNB1;CACNA1C*
Biological Process	Smoothened Signaling Pathway (GO:0007224)	0.1224117	0.5985248	0	0	2.598056418	5.456866708	*TTC26;TCTN2;HIPK1*
Biological Process	Glial Cell Differentiation (GO:0010001)	0.1224117	0.5985248	0	0	2.598056418	5.456866708	*RELN;NFIB;STAT3*

Biological Process	Transcription Initiation-Coupled Chromatin Remodeling (GO:0045815)	0.1224117	0.5985248	0	0	2.598056418	5.456866708	*ATF2;WDR5;EP300*
Biological Process	Membrane Protein Proteolysis (GO:0033619)	0.1224117	0.5985248	0	0	2.598056418	5.456866708	*ERAP1;SPPL3;RBMX*
Biological Process	Endosome Organization (GO:0007032)	0.1229255	0.5985248	0	0	2.192566412	4.596007714	*USP8;ARFGEF2;PLEKHA3;HOOK3*
Biological Process	mRNA Stabilization (GO:0048255)	0.1229255	0.5985248	0	0	2.192566412	4.596007714	*CIRBP;HNRNPU;TARDBP;HNRNPA0*
Biological Process	Negative Regulation Of Metabolic Process (GO:0009892)	0.1229255	0.5985248	0	0	2.192566412	4.596007714	*ADIPOQ;ZDHHC7;LNPEP;LGR4*
Biological Process	Regulation Of Cellular Respiration (GO:0043457)	0.1233653	0.5985248	0	0	3.577530176	7.486360286	*VCP;IDE*
Biological Process	Regulation Of Hematopoietic Progenitor Cell Differentiation (GO:1901532)	0.1233653	0.5985248	0	0	3.577530176	7.486360286	*PRKDC;DHX36*
Biological Process	Regulation Of Oxidative Phosphorylation (GO:0002082)	0.1233653	0.5985248	0	0	3.577530176	7.486360286	*VCP;UQCC2*
Biological Process	Ribosomal Small Subunit Assembly (GO:0000028)	0.1233653	0.5985248	0	0	3.577530176	7.486360286	*PRKDC;MRPS11*
Biological Process	Intracellular Estrogen Receptor Signaling Pathway (GO:0030520)	0.1233653	0.5985248	0	0	3.577530176	7.486360286	*RBFOX2;ARID1A*
Biological Process	Negative Regulation Of Rho Protein Signal Transduction (GO:0035024)	0.1233653	0.5985248	0	0	3.577530176	7.486360286	*CUL3;SCAI*
Biological Process	Nuclear-Transcribed mRNA Catabolic Process, Exonucleolytic (GO:0000291)	0.1233653	0.5985248	0	0	3.577530176	7.486360286	*DIS3;DCP2*
Biological Process	Positive Regulation Of Dendrite Morphogenesis (GO:0050775)	0.1233653	0.5985248	0	0	3.577530176	7.486360286	*RELN;DHX36*
Biological Process	Positive Regulation Of Leukocyte Adhesion To Vascular Endothelial Cell (GO:1904996)	0.1233653	0.5985248	0	0	3.577530176	7.486360286	*NFAT5;TRAF6*
Biological Process	Positive Regulation Of Small GTPase Mediated Signal Transduction (GO:0051057)	0.1233653	0.5985248	0	0	3.577530176	7.486360286	*RELN;RASGRP1*
Biological Process	Receptor Recycling (GO:0001881)	0.1233653	0.5985248	0	0	3.577530176	7.486360286	*USP9X;PLEKHA3*
Biological Process	Regulation Of Adenylate Cyclase Activity (GO:0045761)	0.1233653	0.5985248	0	0	3.577530176	7.486360286	*GNAI3;CACNA1C*
Biological Process	Recombinational Repair (GO:0000725)	0.1277776	0.617723	0	0	1.812010197	3.728145334	*YY1;MBTD1;WDR48;ERCC4;ZMYND8;SMC6*
Biological Process	Import Into Nucleus (GO:0051170)	0.1277887	0.617723	0	0	1.946995034	4.005703553	*CSE1L;STAT3;NUP153;TNPO1;APPL1*
Biological Process	Negative Regulation Of Protein Modification By Small Protein Conjugation Or Removal (GO:1903321)	0.129215	0.6234799	0	0	2.148603352	4.396639332	*U2AF2;CTNNB1;SPRY2;SOX4*
Biological Process	Intrinsic Apoptotic Signaling Pathway By P53 Class Mediator (GO:0072332)	0.1305078	0.6274321	0	0	2.516736402	5.124887083	*JMY;EP300;HIPK1*
Biological Process	Positive Regulation Of Mitotic Nuclear Division (GO:0045840)	0.1305078	0.6274321	0	0	2.516736402	5.124887083	*EPGN;CUL3;PHIP*
Biological Process	Negative Regulation Of Intracellular Signal Transduction (GO:1902532)	0.1308163	0.6277761	0	0	1.475195418	3.000490126	*DUSP5;PPM1A;PRKAA1;DDX3X;PRKAA2;TMEM127;ADIPOQ;PDE3B;PIK3CB;FNIP1;MARK3;VGLL4*
Biological Process	Protein Stabilization (GO:0050821)	0.1316388	0.6291719	0	0	1.50288889	3.047398402	*USP13;ATF7IP;CREB1;USP9X;AAK1;MTMR9;EP300;NAA15;PPARGC1A;SOX4;HCFC1*
Biological Process	Regulation Of Macroautophagy (GO:0016241)	0.1324279	0.6291719	0	0	1.79178338	3.622478464	*PRKAA2;CASP3;WAC;GNAI3;ATG14;SUPT5H*
Biological Process	Regulation Of Fat Cell Differentiation (GO:0045598)	0.1331302	0.6291719	0	0	1.919080919	3.869688556	*ANKRD26;CREB1;ADIPOQ;TAF8;RUNX1T1*
Biological Process	Nucleosome Organization (GO:0034728)	0.1331302	0.6291719	0	0	1.919080919	3.869688556	*LIN54;SUPT16H;RBBP4;ATRX;ARID1A*
Biological Process	Positive Regulation Of Cell Cycle Process (GO:0090068)	0.1336838	0.6291719	0	0	1.695450008	3.411716231	*EPGN;CUL3;CEP120;ATRX;PHIP;PKN2;SMC6*

Biological Process	3'-UTR-mediated mRNA Stabilization (GO:0070935)	0.1356199	0.6291719	0	0	3.353760446	6.700474552	*TARDBP;HNRNPA0*
Biological Process	Regulation Of Mitochondrial Outer Membrane Permeabilization Involved In Apoptotic Signaling Pathway (GO:1901028)	0.1356199	0.6291719	0	0	3.353760446	6.700474552	*GSK3B;SLC35F6*
Biological Process	Antigen Processing And Presentation Of Peptide Antigen Via MHC Class I (GO:0002474)	0.1356199	0.6291719	0	0	3.353760446	6.700474552	*ERAP1;IDE*
Biological Process	Detection Of Mechanical Stimulus (GO:0050982)	0.1356199	0.6291719	0	0	3.353760446	6.700474552	*CTNNB1;TTN*
Biological Process	Energy Reserve Metabolic Process (GO:0006112)	0.1356199	0.6291719	0	0	3.353760446	6.700474552	*GSK3B;GFPT1*
Biological Process	Vesicle Transport Along Actin Filament (GO:0030050)	0.1356199	0.6291719	0	0	3.353760446	6.700474552	*MYO15A;MYO5A*
Biological Process	Maintenance Of Protein Localization In Organelle (GO:0072595)	0.1356199	0.6291719	0	0	3.353760446	6.700474552	*TAF8;HNRNPU*
Biological Process	Maintenance Of Protein Location In Nucleus (GO:0051457)	0.1356199	0.6291719	0	0	3.353760446	6.700474552	*TAF8;HNRNPU*
Biological Process	Melanosome Assembly (GO:1903232)	0.1356199	0.6291719	0	0	3.353760446	6.700474552	*AP3M1;AP1G1*
Biological Process	Mitotic Cytokinetic Process (GO:1902410)	0.1356199	0.6291719	0	0	3.353760446	6.700474552	*PDCD6IP;CNTROB*
Biological Process	Negative Regulation Of Lipid Catabolic Process (GO:0050995)	0.1356199	0.6291719	0	0	3.353760446	6.700474552	*PRKAA1;PDE3B*
Biological Process	Neural Crest Cell Differentiation (GO:0014033)	0.1356199	0.6291719	0	0	3.353760446	6.700474552	*NOLC1;LRP6*
Biological Process	Positive Regulation Of Neuroblast Proliferation (GO:0002052)	0.1356199	0.6291719	0	0	3.353760446	6.700474552	*FZD3;CTNNB1*
Biological Process	Positive Regulation Of Response To Endoplasmic Reticulum Stress (GO:1905898)	0.1356199	0.6291719	0	0	3.353760446	6.700474552	*USP13;NCK1*
Biological Process	Regulation Of Defense Response (GO:0031347)	0.1371577	0.6351938	0	0	1.772001108	3.520299197	*CELF1;ELMOD2;ERAP1;SPATA2;XIAP;APPL1*
Biological Process	Wnt Signaling Pathway (GO:0016055)	0.1385701	0.6371652	0	0	1.891953117	3.739216445	*FZD3;DDX3X;SIAH1;CTNNB1;LRP6*
Biological Process	Interstrand Cross-Link Repair (GO:0036297)	0.1387861	0.6371652	0	0	2.440344871	4.819245499	*VCP;ERCC4;RNF8*
Biological Process	Lytic Vacuole Organization (GO:0080171)	0.1387861	0.6371652	0	0	2.440344871	4.819245499	*CLVS2;HOOK3;TPCN2*
Biological Process	Positive Regulation Of Double-Strand Break Repair Via Homologous Recombination (GO:1905168)	0.1387861	0.6371652	0	0	2.440344871	4.819245499	*MBTD1;WDR48;ING3*
Biological Process	Positive Regulation Of Interferon-Beta Production (GO:0032728)	0.1387861	0.6371652	0	0	2.440344871	4.819245499	*DDX3X;POLR3C;POLR3F*
Biological Process	RNA Splicing (GO:0008380)	0.1419657	0.6384791	0	0	1.752648886	3.421467936	*DHX8;GEMIN2;SRSF2;TARDBP;PPARGC1A;SRSF10*
Biological Process	Negative Regulation Of Macromolecule Biosynthetic Process (GO:0010558)	0.1419657	0.6384791	0	0	1.752648886	3.421467936	*GSK3B;DDX3X;ADIPOQ;DHX36;CPEB3;EIF4E2*
Biological Process	Mitotic DNA Damage Checkpoint Signaling (GO:0044773)	0.1421825	0.6384791	0	0	2.065749893	4.029542563	*ATF2;PRKDC;WAC;FBXO31*
Biological Process	Regulation Of Immune Response (GO:0050776)	0.1441062	0.6384791	0	0	1.865578866	3.614008507	*DENND1B;ERAP1;XIAP;CXCL13;APPL1*
Biological Process	Positive Regulation Of Response To Stimulus (GO:0048584)	0.1441062	0.6384791	0	0	1.865578866	3.614008507	*YY1;INO80D;EYA3;ADIPOQ;RNF8*
Biological Process	Transmembrane Receptor Protein Tyrosine Kinase Signaling Pathway (GO:0007169)	0.1468659	0.6384791	0	0	1.396180883	2.678203764	*GSK3B;STAT3;IDE;SORBS1;PIK3CB;FER;RAB14;CASP3;RASA1;MAPK1;PHIP;ARHGEF7;EPHB1;APPL1*
Biological Process	Negative Regulation Of Transferase Activity (GO:0051348)	0.1472366	0.6384791	0	0	2.36844696	4.537268705	*GSK3B;ERCC4;HNRNPU*
Biological Process	COPII Vesicle Coating (GO:0048208)	0.1481292	0.6384791	0	0	3.156316566	6.02752398	*PPP6C;CUL3*

Biological Process	Regulation Of Fibroblast Growth Factor Receptor Signaling Pathway (GO:0040036)	0.1481292	0.6384791	0	0	3.156316566	6.02752398	*SPRY2;SPRY1*
Biological Process	Atrioventricular Valve Morphogenesis (GO:0003181)	0.1481292	0.6384791	0	0	3.156316566	6.02752398	*HEY1;SOX4*
Biological Process	Calcium-Independent Cell-Cell Adhesion Via Plasma Membrane Cell-Adhesion Molecules (GO:0016338)	0.1481292	0.6384791	0	0	3.156316566	6.02752398	*CLDN12;CLDN18*
Biological Process	Cardiac Epithelial To Mesenchymal Transition (GO:0060317)	0.1481292	0.6384791	0	0	3.156316566	6.02752398	*HEY1;SPRY1*
Biological Process	Cellular Response To Lectin (GO:1990858)	0.1481292	0.6384791	0	0	3.156316566	6.02752398	*TRAF6;EP300*
Biological Process	Regulation Of Vascular Endothelial Growth Factor Signaling Pathway (GO:1900746)	0.1481292	0.6384791	0	0	3.156316566	6.02752398	*SPRY2;PIK3CB*
Biological Process	Dopaminergic Neuron Differentiation (GO:0071542)	0.1481292	0.6384791	0	0	3.156316566	6.02752398	*CTNNB1;LRP6*
Biological Process	snRNA Processing (GO:0016180)	0.1481292	0.6384791	0	0	3.156316566	6.02752398	*INTS7;INTS6*
Biological Process	Stimulatory C-type Lectin Receptor Signaling Pathway (GO:0002223)	0.1481292	0.6384791	0	0	3.156316566	6.02752398	*TRAF6;EP300*
Biological Process	Glycosphingolipid Biosynthetic Process (GO:0006688)	0.1481292	0.6384791	0	0	3.156316566	6.02752398	*FUT9;ST3GAL5*
Biological Process	Vesicle Targeting, Rough ER To cis-Golgi (GO:0048207)	0.1481292	0.6384791	0	0	3.156316566	6.02752398	*PPP6C;CUL3*
Biological Process	Inositol Phosphate-Mediated Signaling (GO:0048016)	0.1481292	0.6384791	0	0	3.156316566	6.02752398	*NFAT5;DHX8*
Biological Process	Intrinsic Apoptotic Signaling Pathway In Response To DNA Damage By P53 Class Mediator (GO:0042771)	0.1481292	0.6384791	0	0	3.156316566	6.02752398	*EP300;HIPK1*
Biological Process	Cellular Response To Oxygen-Containing Compound (GO:1901701)	0.1481427	0.6384791	0	0	1.323200153	2.526755806	*MEF2A;SYT4;GSK3B;PRKAA1;VCP;PRKAA2;BCL10;CXCL13;CAMKK2;FER;CREB1;TRAF6;RRAGD;DHX36;ZNF703;LCOR;PLAA;MAPK1;CPEB3*
Biological Process	Ras Protein Signal Transduction (GO:0007265)	0.1487739	0.6384791	0	0	1.581625909	3.01351506	*USP8;ARHGAP29;ARHGAP5;SOS1;RASGRP1;RAPGEF6;CRKL;RALGPS2*
Biological Process	Regulation Of Cell Adhesion (GO:0030155)	0.1487739	0.6384791	0	0	1.581625909	3.01351506	*ATXN3;EPB41L4B;RASA1;PDE3B;MIA3;PLXNC1;STX3;TNPO1*
Biological Process	Endosome To Lysosome Transport (GO:0008333)	0.1488518	0.6384791	0	0	2.026668072	3.86040578	*VCP;HOOK3;ATG14;TPCN2*
Biological Process	Organonitrogen Compound Catabolic Process (GO:1901565)	0.1497361	0.6384791	0	0	1.839927196	3.493803161	*CASP3;ERAP1;SIAH1;LNPEP;IDE*
Biological Process	Regulation Of GTPase Activity (GO:0043087)	0.1508265	0.6384791	0	0	1.458009963	2.758008045	*GSK3B;TBC1D30;RAB3GAP2;PLXNC1;ARHGEF7;SOS1;RAPGEF6;RASGRP1;EVI5;RALGPS2;RGS7*
Biological Process	Regulation Of Organelle Organization (GO:0033043)	0.1518104	0.6384791	0	0	1.715179689	3.233324629	*YY1;INO80D;SURF4;EP300;MYO5A;MAPK1*
Biological Process	ncRNA Processing (GO:0034470)	0.1518104	0.6384791	0	0	1.715179689	3.233324629	*SSB;DIS3;NOLC1;INTS7;INTS6;TSEN2*
Biological Process	Protein Complex Oligomerization (GO:0051259)	0.1518104	0.6384791	0	0	1.715179689	3.233324629	*CPSF6;PDCD6IP;ATL2;TIFA;BCL10;RBMX*
Biological Process	Regulation Of Neuron Differentiation (GO:0045664)	0.1554573	0.6384791	0	0	1.814968815	3.378354702	*RELN;HEY1;VWC2;NREP;CNTN4*
Biological Process	Protein Import Into Nucleus (GO:0006606)	0.1554573	0.6384791	0	0	1.814968815	3.378354702	*CSE1L;STAT3;NUP153;TNPO1;APPL1*
Biological Process	mRNA Transport (GO:0051028)	0.1556389	0.6384791	0	0	1.989033726	3.700034122	*FYTTD1;NXF1;SARNP;HNRNPA2B1*
Biological Process	Maturation Of SSU-rRNA (GO:0030490)	0.1558491	0.6384791	0	0	2.300657501	4.27661686	*MRPS11;RIOK2;DDX52*
Biological Process	Negative Regulation Of Epithelial Cell Apoptotic Process (GO:1904036)	0.1558491	0.6384791	0	0	2.300657501	4.27661686	*PRKAA1;PRKAA2;PDPK1*
Biological Process	Positive Regulation Of Myoblast Differentiation (GO:0045663)	0.1558491	0.6384791	0	0	2.300657501	4.27661686	*PBRM1;ARID1A;SOX4*
Biological Process	rRNA Processing (GO:0006364)	0.1568441	0.6384791	0	0	1.697036709	3.143765817	*PRKDC;ERI1;DIS3;NOLC1;RIOK2;DDX52*
Biological Process	Vesicle-Mediated Transport (GO:0016192)	0.1601363	0.6384791	0	0	1.305976884	2.392197327	*DENND1B;SYT4;ITSN1;MYO5A;RAB39B;MIA3;HOOK3;ATG14;ARL17A;AP3M1;SNX27;RAB14;AP1G1;REPS2;EXOC6;STX3;TBC1D23;ARL5A;TRIM23*

Biological Process	Negative Regulation Of Growth (GO:0045926)	0.1601413	0.6384791	0	0	1.608000384	2.945372133	*ST7L;DDX3X;GNG4;SERTAD2;EI24;TMPRSS4;VGLL4*
Biological Process	DNA Methylation (GO:0006306)	0.160859	0.6384791	0	0	2.980810894	5.44661858	*ATRX;DNMT3A*
Biological Process	Regulation Of Fibroblast Migration (GO:0010762)	0.160859	0.6384791	0	0	2.980810894	5.44661858	*ARHGEF7;APPL1*
Biological Process	Actin Filament-Based Transport (GO:0099515)	0.160859	0.6384791	0	0	2.980810894	5.44661858	*MYO15A;MYO5A*
Biological Process	Ameboidal-Type Cell Migration (GO:0001667)	0.160859	0.6384791	0	0	2.980810894	5.44661858	*PKN2;AMOTL1*
Biological Process	Regulation Of Mitotic Spindle Assembly (GO:1901673)	0.160859	0.6384791	0	0	2.980810894	5.44661858	*PDCD6IP;HNRNPU*
Biological Process	Regulation Of Phosphatase Activity (GO:0010921)	0.160859	0.6384791	0	0	2.980810894	5.44661858	*PPP6R2;MTMR9*
Biological Process	Regulation Of Protein Localization To Cell Periphery (GO:1904375)	0.160859	0.6384791	0	0	2.980810894	5.44661858	*ZDHHC7;APPL1*
Biological Process	Cellular Response To Estradiol Stimulus (GO:0071392)	0.160859	0.6384791	0	0	2.980810894	5.44661858	*ZNF703;LCOR*
Biological Process	Negative Regulation Of Small GTPase Mediated Signal Transduction (GO:0051058)	0.160859	0.6384791	0	0	2.980810894	5.44661858	*SPRY2;SPRY1*
Biological Process	Receptor Metabolic Process (GO:0043112)	0.160859	0.6384791	0	0	2.980810894	5.44661858	*SMURF1;PLEKHA3*
Biological Process	Axonal Transport (GO:0098930)	0.1646135	0.6384791	0	0	2.236634124	4.035234327	*AP3M1;KIF3A;FBXW11*
Biological Process	Regulation Of Receptor-Mediated Endocytosis (GO:0048259)	0.1646135	0.6384791	0	0	2.236634124	4.035234327	*PIK3CB;CBL;SNAP91*
Biological Process	Heterochromatin Organization (GO:0070828)	0.1646135	0.6384791	0	0	2.236634124	4.035234327	*SETDB1;HP1BP3;TRIP12*
Biological Process	Internal Peptidyl-Lysine Acetylation (GO:0018393)	0.1646135	0.6384791	0	0	2.236634124	4.035234327	*MBTD1;ING3;EP300*
Biological Process	Negative Regulation Of Cell-Substrate Adhesion (GO:0010812)	0.1646135	0.6384791	0	0	2.236634124	4.035234327	*RASA1;SPOCK1;CASK*
Biological Process	Positive Regulation Of Lymphocyte Differentiation (GO:0045621)	0.1646135	0.6384791	0	0	2.236634124	4.035234327	*PBRM1;PRKDC;ARID1A*
Biological Process	Cytoskeleton-Dependent Cytokinesis (GO:0061640)	0.1671636	0.6384791	0	0	1.767022451	3.160818223	*USP8;PDCD6IP;SON;RASA1;EXOC6*
Biological Process	Histone Modification (GO:0016570)	0.1671636	0.6384791	0	0	1.767022451	3.160818223	*MBTD1;ING3;RBBP4;TBL1XR1;EP300*
Biological Process	5S Class rRNA Transcription By RNA Polymerase III (GO:0042791)	0.1675134	0.6384791	0	0	6.702364395	11.9750608	*GTF3C4*
Biological Process	B Cell Chemotaxis (GO:0035754)	0.1675134	0.6384791	0	0	6.702364395	11.9750608	*CXCL13*
Biological Process	DNA Replication-Dependent Chromatin Assembly (GO:0006335)	0.1675134	0.6384791	0	0	6.702364395	11.9750608	*RBBP4*
Biological Process	Regulation Of Endoplasmic Reticulum Tubular Network Organization (GO:1903371)	0.1675134	0.6384791	0	0	6.702364395	11.9750608	*RAB3GAP2*
Biological Process	Regulation Of Glycogen (Starch) Synthase Activity (GO:2000465)	0.1675134	0.6384791	0	0	6.702364395	11.9750608	*GSK3B*
Biological Process	R-loop Processing (GO:0062176)	0.1675134	0.6384791	0	0	6.702364395	11.9750608	*NFAT5*
Biological Process	Regulation Of Growth Hormone Receptor Signaling Pathway (GO:0060398)	0.1675134	0.6384791	0	0	6.702364395	11.9750608	*MBD5*
Biological Process	Regulation Of Hematopoietic Stem Cell Proliferation (GO:1902033)	0.1675134	0.6384791	0	0	6.702364395	11.9750608	*N4BP2L2*
Biological Process	acetyl-CoA Biosynthetic Process From Pyruvate (GO:0006086)	0.1675134	0.6384791	0	0	6.702364395	11.9750608	*PDHA1*
Biological Process	Adiponectin-Activated Signaling Pathway (GO:0033211)	0.1675134	0.6384791	0	0	6.702364395	11.9750608	*APPL1*
Biological Process	Regulation Of Macrophage Proliferation (GO:0120040)	0.1675134	0.6384791	0	0	6.702364395	11.9750608	*MAPK1*
Biological Process	Astrocyte Differentiation (GO:0048708)	0.1675134	0.6384791	0	0	6.702364395	11.9750608	*STAT3*

Biological Process	Bradykinin Catabolic Process (GO:0010815)	0.1675134	0.6384791	0	0	6.702364395	11.9750608	*IDE*
Biological Process	Regulation Of pro-B Cell Differentiation (GO:2000973)	0.1675134	0.6384791	0	0	6.702364395	11.9750608	*FNIP1*
Biological Process	Cardiolipin Acyl-Chain Remodeling (GO:0035965)	0.1675134	0.6384791	0	0	6.702364395	11.9750608	*LCLAT1*
Biological Process	Regulation Of T-Circle Formation (GO:1904429)	0.1675134	0.6384791	0	0	6.702364395	11.9750608	*RTEL1*
Biological Process	Regulation Of Translation In Response To Endoplasmic Reticulum Stress (GO:0036490)	0.1675134	0.6384791	0	0	6.702364395	11.9750608	*NCK1*
Biological Process	Cerebral Cortex Cell Migration (GO:0021795)	0.1675134	0.6384791	0	0	6.702364395	11.9750608	*RELN*
Biological Process	Retinal Ganglion Cell Axon Guidance (GO:0031290)	0.1675134	0.6384791	0	0	6.702364395	11.9750608	*EPHB1*
Biological Process	Embryonic Brain Development (GO:1990403)	0.1675134	0.6384791	0	0	6.702364395	11.9750608	*TBC1D23*
Biological Process	Embryonic Hindlimb Morphogenesis (GO:0035116)	0.1675134	0.6384791	0	0	6.702364395	11.9750608	*OSR2*
Biological Process	Embryonic Skeletal Joint Development (GO:0072498)	0.1675134	0.6384791	0	0	6.702364395	11.9750608	*OSR2*
Biological Process	Signal Complex Assembly (GO:0007172)	0.1675134	0.6384791	0	0	6.702364395	11.9750608	*NCK1*
Biological Process	Establishment Of Lymphocyte Polarity (GO:0001767)	0.1675134	0.6384791	0	0	6.702364395	11.9750608	*SNX27*
Biological Process	Striated Muscle Hypertrophy (GO:0014897)	0.1675134	0.6384791	0	0	6.702364395	11.9750608	*TTN*
Biological Process	tRNA Pseudouridine Synthesis (GO:0031119)	0.1675134	0.6384791	0	0	6.702364395	11.9750608	*PUS10*
Biological Process	tRNA Transport (GO:0051031)	0.1675134	0.6384791	0	0	6.702364395	11.9750608	*SSB*
Biological Process	Thiamine-Containing Compound Metabolic Process (GO:0042723)	0.1675134	0.6384791	0	0	6.702364395	11.9750608	*SLC19A2*
Biological Process	Tight Junction Organization (GO:0120193)	0.1675134	0.6384791	0	0	6.702364395	11.9750608	*CLDN12*
Biological Process	Tongue Development (GO:0043586)	0.1675134	0.6384791	0	0	6.702364395	11.9750608	*SIX4*
Biological Process	Ventricular Cardiac Muscle Cell Development (GO:0055015)	0.1675134	0.6384791	0	0	6.702364395	11.9750608	*MEF2A*
Biological Process	Zymogen Inhibition (GO:0097341)	0.1675134	0.6384791	0	0	6.702364395	11.9750608	*XIAP*
Biological Process	Immunoglobulin V(D)J Recombination (GO:0033152)	0.1675134	0.6384791	0	0	6.702364395	11.9750608	*PRKDC*
Biological Process	interleukin-17A-mediated Signaling Pathway (GO:0038173)	0.1675134	0.6384791	0	0	6.702364395	11.9750608	*TRAF6*
Biological Process	Iron Coordination Entity Transport (GO:1901678)	0.1675134	0.6384791	0	0	6.702364395	11.9750608	*SLC22A17*
Biological Process	Kidney Morphogenesis (GO:0060993)	0.1675134	0.6384791	0	0	6.702364395	11.9750608	*SOX4*
Biological Process	Morphogenesis Of An Endothelium (GO:0003159)	0.1675134	0.6384791	0	0	6.702364395	11.9750608	*CTNNB1*
Biological Process	Negative Regulation Of PERK-mediated Unfolded Protein Response (GO:1903898)	0.1675134	0.6384791	0	0	6.702364395	11.9750608	*NCK1*
Biological Process	Negative Regulation Of Cardiac Muscle Tissue Growth (GO:0055022)	0.1675134	0.6384791	0	0	6.702364395	11.9750608	*VGLL4*
Biological Process	Negative Regulation Of Glucocorticoid Receptor Signaling Pathway (GO:2000323)	0.1675134	0.6384791	0	0	6.702364395	11.9750608	*CLOCK*
Biological Process	Negative Regulation Of Glycogen Biosynthetic Process (GO:0045719)	0.1675134	0.6384791	0	0	6.702364395	11.9750608	*GSK3B*
Biological Process	Negative Regulation Of Lamellipodium Organization (GO:1902744)	0.1675134	0.6384791	0	0	6.702364395	11.9750608	*ARPIN*
Biological Process	Negative Regulation Of Mast Cell Activation (GO:0033004)	0.1675134	0.6384791	0	0	6.702364395	11.9750608	*FER*

Biological Process	Negative Regulation Of Protein Localization To Chromatin (GO:0120186)	0.1675134	0.6384791	0	0	6.702364395	11.9750608	*VCP*
Biological Process	Negative Regulation Of Protein Polyubiquitination (GO:1902915)	0.1675134	0.6384791	0	0	6.702364395	11.9750608	*PLAA*
Biological Process	Negative Regulation Of Transcription From RNA Polymerase II Promoter In Response To Stress (GO:0097201)	0.1675134	0.6384791	0	0	6.702364395	11.9750608	*NCK1*
Biological Process	Ornithine Transport (GO:0015822)	0.1675134	0.6384791	0	0	6.702364395	11.9750608	*SLC7A6*
Biological Process	Phosphatidylinositol Acyl-Chain Remodeling (GO:0036149)	0.1675134	0.6384791	0	0	6.702364395	11.9750608	*LCLAT1*
Biological Process	Photoreceptor Cell Differentiation (GO:0046530)	0.1675134	0.6384791	0	0	6.702364395	11.9750608	*STAT3*
Biological Process	Plus-End-Directed Organelle Transport Along Microtubule (GO:0072386)	0.1675134	0.6384791	0	0	6.702364395	11.9750608	*KIF3A*
Biological Process	Positive Regulation Of Wnt Signaling Pathway, Planar Cell Polarity Pathway (GO:2000096)	0.1675134	0.6384791	0	0	6.702364395	11.9750608	*MLLT3*
Biological Process	Positive Regulation Of Animal Organ Morphogenesis (GO:0110110)	0.1675134	0.6384791	0	0	6.702364395	11.9750608	*SIX4*
Biological Process	Positive Regulation Of Cell-Cell Adhesion Mediated By Integrin (GO:0033634)	0.1675134	0.6384791	0	0	6.702364395	11.9750608	*CXCL13*
Biological Process	Positive Regulation Of Cholesterol Biosynthetic Process (GO:0045542)	0.1675134	0.6384791	0	0	6.702364395	11.9750608	*PRKAA1*
Biological Process	Positive Regulation Of Mitochondrial Membrane Potential (GO:0010918)	0.1675134	0.6384791	0	0	6.702364395	11.9750608	*VCP*
Biological Process	Positive Regulation Of Protein Localization To Cilium (GO:1903566)	0.1675134	0.6384791	0	0	6.702364395	11.9750608	*GSK3B*
Biological Process	Positive Regulation Of Sterol Biosynthetic Process (GO:0106120)	0.1675134	0.6384791	0	0	6.702364395	11.9750608	*PRKAA1*
Biological Process	Positive Regulation Of Translation In Response To Stress (GO:0032056)	0.1675134	0.6384791	0	0	6.702364395	11.9750608	*NCK1*
Biological Process	Protein Targeting To Vacuole Involved In Autophagy (GO:0071211)	0.1675134	0.6384791	0	0	6.702364395	11.9750608	*SMURF1*
Biological Process	Radial Glial Cell Differentiation (GO:0060019)	0.1675134	0.6384791	0	0	6.702364395	11.9750608	*STAT3*
Biological Process	Regulation Of Apoptotic DNA Fragmentation (GO:1902510)	0.1675134	0.6384791	0	0	6.702364395	11.9750608	*DFFB*
Biological Process	Response To Light Stimulus (GO:0009416)	0.1695481	0.6403329	0	0	1.917797287	3.403358607	*ERCC4;CIRBP;CLOCK;MAP4K3*
Biological Process	Regulation Of Phosphorylation (GO:0042325)	0.1731436	0.6403329	0	0	1.743983289	3.058308325	*FER;PHIP;BCL10;ATG14;FNIP1*
Biological Process	Negative Regulation Of Apoptotic Signaling Pathway (GO:2001234)	0.1731436	0.6403329	0	0	1.743983289	3.058308325	*SLC35F6;DDX3X;CAAP1;PHIP;GRINA*
Biological Process	Regulation Of Small Molecule Metabolic Process (GO:0062012)	0.1735199	0.6403329	0	0	2.176071469	3.811308816	*ADIPOQ;EP300;FOXK1*
Biological Process	Cellular Response To Heat (GO:0034605)	0.1735199	0.6403329	0	0	2.176071469	3.811308816	*ATXN3;VCP;DHX36*
Biological Process	Establishment Or Maintenance Of Epithelial Cell Apical/Basal Polarity (GO:0045197)	0.1735199	0.6403329	0	0	2.176071469	3.811308816	*PDCD6IP;PARD6G;LIN7C*
Biological Process	mRNA 3'-End Processing (GO:0031124)	0.1735199	0.6403329	0	0	2.176071469	3.811308816	*CPSF6;RPRD1A;RPRD2*
Biological Process	Protein Deacetylation (GO:0006476)	0.1735199	0.6403329	0	0	2.176071469	3.811308816	*RBBP4;TBL1XR1;SIRT5*
Biological Process	Protein Localization To Chromosome (GO:0034502)	0.1735199	0.6403329	0	0	2.176071469	3.811308816	*ZMYND8;ATRX;TERF2IP*

Biological Process	Protein Localization To Cilium (GO:0061512)	0.1735199	0.6403329	0	0	2.176071469	3.811308816	*TTC26;TCTN2;TTC21B*
Biological Process	Anaphase-Promoting Complex-Dependent Catabolic Process (GO:0031145)	0.1737772	0.6403329	0	0	2.823779504	4.941560966	*CUL3;FBXO31*
Biological Process	snRNA Metabolic Process (GO:0016073)	0.1737772	0.6403329	0	0	2.823779504	4.941560966	*INTS7;INTS6*
Biological Process	Histone H2A Acetylation (GO:0043968)	0.1737772	0.6403329	0	0	2.823779504	4.941560966	*MBTD1;ING3*
Biological Process	Maturation Of 5.8S rRNA From Tricistronic rRNA Transcript (SSU-rRNA, 5.8S rRNA, LSU-rRNA) (GO:0000466)	0.1737772	0.6403329	0	0	2.823779504	4.941560966	*ERI1;ERI2*
Biological Process	Negative Regulation Of Reactive Oxygen Species Metabolic Process (GO:2000378)	0.1737772	0.6403329	0	0	2.823779504	4.941560966	*ABCB7;SIRT5*
Biological Process	poly(A)+ mRNA Export From Nucleus (GO:0016973)	0.1737772	0.6403329	0	0	2.823779504	4.941560966	*NXF1;SARNP*
Biological Process	Positive Regulation Of T Cell Cytokine Production (GO:0002726)	0.1737772	0.6403329	0	0	2.823779504	4.941560966	*DENND1B;TRAF6*
Biological Process	Positive Regulation Of Interferon-Alpha Production (GO:0032727)	0.1737772	0.6403329	0	0	2.823779504	4.941560966	*DDX3X;DHX36*
Biological Process	Positive Regulation Of Transcription Regulatory Region DNA Binding (GO:2000679)	0.1737772	0.6403329	0	0	2.823779504	4.941560966	*TRAF6;EP300*
Biological Process	Golgi Vesicle Transport (GO:0048193)	0.1742744	0.6403329	0	0	1.438050764	2.512453589	*COG6;CUL3;SURF4;ATL2;YIPF5;MYO5A;MIA3;SCAMP1;TMED4;BET1*
Biological Process	Negative Regulation Of Autophagy (GO:0010507)	0.176661	0.6403329	0	0	1.88405371	3.266049554	*RRAGD;STAT3;MTMR9;FOXK1*
Biological Process	Regulation Of Epithelial To Mesenchymal Transition (GO:0010717)	0.1792046	0.6403329	0	0	1.721534875	2.959709711	*ZNF703;CTNNB1;SPRY2;SPRY1;CLASP2*
Biological Process	DNA-templated Transcription (GO:0006351)	0.1819129	0.6403329	0	0	1.422684254	2.424577146	*MEF2A;GTF2A1;NFAT5;SUPT16H;CCNT2;GTF3C4;NCOA6;NFIC;ETV1;RBMX*
Biological Process	Double-Strand Break Repair Via Nonhomologous End Joining (GO:0006303)	0.1825582	0.6403329	0	0	2.118696322	3.603237274	*PRKDC;ERCC4;RNF8*
Biological Process	Positive Regulation Of Viral Process (GO:0048524)	0.1825582	0.6403329	0	0	2.118696322	3.603237274	*DDX3X;CD209;PKN2*
Biological Process	Protein Homooligomerization (GO:0051260)	0.1838483	0.6403329	0	0	1.541696365	2.611084924	*PDCD6IP;ATL2;TIFA;BCL10;TNPO1;RBMX;GLS*
Biological Process	Spliceosomal Complex Assembly (GO:0000245)	0.1838732	0.6403329	0	0	1.851473704	3.135487174	*CELF1;SRSF1;CELF4;SRSF10*
Biological Process	Fat Cell Differentiation (GO:0045444)	0.1838732	0.6403329	0	0	1.851473704	3.135487174	*ADIPOQ;ERAP1;EP300;PPARGC1A*
Biological Process	mRNA Export From Nucleus (GO:0006406)	0.1838732	0.6403329	0	0	1.851473704	3.135487174	*FYTTD1;NXF1;SARNP;HNRNPA2B1*
Biological Process	Phosphatidylinositol-Mediated Signaling (GO:0048015)	0.1838732	0.6403329	0	0	1.851473704	3.135487174	*PLCB4;STAT3;PLCH1;PIK3CB*
Biological Process	Regulation Of Histone Acetylation (GO:0035065)	0.1868539	0.6403329	0	0	2.682451253	4.499619662	*BAZ1B;PPARGC1A*
Biological Process	Regulation Of Intracellular Steroid Hormone Receptor Signaling Pathway (GO:0033143)	0.1868539	0.6403329	0	0	2.682451253	4.499619662	*EP300;UFSP2*
Biological Process	Regulation Of Mitochondrial Translation (GO:0070129)	0.1868539	0.6403329	0	0	2.682451253	4.499619662	*MRPS27;UQCC2*
Biological Process	Ribosomal Large Subunit Assembly (GO:0000027)	0.1868539	0.6403329	0	0	2.682451253	4.499619662	*RPF2;MDN1*
Biological Process	snRNA 3'-End Processing (GO:0034472)	0.1868539	0.6403329	0	0	2.682451253	4.499619662	*INTS7;INTS6*
Biological Process	Vesicle Transport Along Microtubule (GO:0047496)	0.1868539	0.6403329	0	0	2.682451253	4.499619662	*FBXW11;KIF3A*
Biological Process	Negative Regulation Of Cell Communication (GO:0010648)	0.1868539	0.6403329	0	0	2.682451253	4.499619662	*ADIPOQ;OPRM1*

Biological Process	Negative Regulation Of Cell Development (GO:0010721)	0.1868539	0.6403329	0	0	2.682451253	4.499619662	*GSK3B;HOOK3*
Biological Process	peptidyl-L-cysteine S-palmitoylation (GO:0018230)	0.1868539	0.6403329	0	0	2.682451253	4.499619662	*ZDHHC21;ZDHHC7*
Biological Process	peptidyl-S-diacylglycerol-L-cysteine Biosynthetic Process From Peptidyl-Cysteine (GO:0018231)	0.1868539	0.6403329	0	0	2.682451253	4.499619662	*ZDHHC21;ZDHHC7*
Biological Process	Platelet Dense Granule Organization (GO:0060155)	0.1868539	0.6403329	0	0	2.682451253	4.499619662	*AP3M1;AP1G1*
Biological Process	Positive Regulation Of Cilium Assembly (GO:0045724)	0.1868539	0.6403329	0	0	2.682451253	4.499619662	*GSK3B;CEP120*
Biological Process	Positive Regulation Of Viral Life Cycle (GO:1903902)	0.1868539	0.6403329	0	0	2.682451253	4.499619662	*CD209;TMPRSS4*
Biological Process	Establishment Of Protein Localization To Organelle (GO:0072594)	0.1915586	0.6403329	0	0	1.678321678	2.773529657	*CSE1L;STAT3;NUP153;TNPO1;APPL1*
Biological Process	Positive Regulation Of Protein Localization (GO:1903829)	0.1915586	0.6403329	0	0	1.678321678	2.773529657	*GSK3B;PRKAA1;PRKAA2;CEP120;STX3*
Biological Process	Cellular Response To Amino Acid Starvation (GO:0034198)	0.1917188	0.6403329	0	0	2.064263491	3.40959728	*BMT2;RRAGD;MAPK1*
Biological Process	Cellular Response To Glucose Starvation (GO:0042149)	0.1917188	0.6403329	0	0	2.064263491	3.40959728	*PRKAA1;PRKAA2;ATG14*
Biological Process	Regulation Of Ubiquitin-Dependent Protein Catabolic Process (GO:2000058)	0.1917188	0.6403329	0	0	2.064263491	3.40959728	*NUB1;SMURF1;UBE3A*
Biological Process	Stress-Activated MAPK Cascade (GO:0051403)	0.1917188	0.6403329	0	0	2.064263491	3.40959728	*MAPK1;MAP4K3;CRKL*
Biological Process	Modulation By Host Of Symbiont Process (GO:0051851)	0.1917188	0.6403329	0	0	2.064263491	3.40959728	*TFAP4;EP300;TARDBP*
Biological Process	Synapse Organization (GO:0050808)	0.1937185	0.6403329	0	0	1.516671945	2.489388199	*CACNB4;SPOCK2;PPFIA1;RAB39B;ARHGAP39;APPL1;ADGRL3*
Biological Process	Response To Tumor Necrosis Factor (GO:0034612)	0.1940066	0.6403329	0	0	1.579996705	2.590978206	*NUB1;SLC22A5;CASP3;ADIPOQ;AFF3;MAP4K3*
Biological Process	Regulation Of Centriole Elongation (GO:1903722)	0.1974904	0.6403329	0	0	5.361613352	8.696888062	*CEP120*
Biological Process	Regulation Of Glucocorticoid Receptor Signaling Pathway (GO:2000322)	0.1974904	0.6403329	0	0	5.361613352	8.696888062	*CLOCK*
Biological Process	Regulation Of Ketone Biosynthetic Process (GO:0010566)	0.1974904	0.6403329	0	0	5.361613352	8.696888062	*SIRT5*
Biological Process	Adenosine To Inosine Editing (GO:0006382)	0.1974904	0.6403329	0	0	5.361613352	8.696888062	*ADAT2*
Biological Process	Antigen Processing And Presentation Of Exogenous Peptide Antigen Via MHC Class I (GO:0042590)	0.1974904	0.6403329	0	0	5.361613352	8.696888062	*LNPEP*
Biological Process	Atrioventricular Valve Formation (GO:0003190)	0.1974904	0.6403329	0	0	5.361613352	8.696888062	*HEY1*
Biological Process	Cellular Hyperosmotic Response (GO:0071474)	0.1974904	0.6403329	0	0	5.361613352	8.696888062	*NFAT5*
Biological Process	Cellular Hypotonic Response (GO:0071476)	0.1974904	0.6403329	0	0	5.361613352	8.696888062	*SLC12A6*
Biological Process	Regulation Of Skeletal Muscle Satellite Cell Proliferation (GO:0014842)	0.1974904	0.6403329	0	0	5.361613352	8.696888062	*EPHB1*
Biological Process	Cellular Response To Hepatocyte Growth Factor Stimulus (GO:0035729)	0.1974904	0.6403329	0	0	5.361613352	8.696888062	*APPL1*
Biological Process	Cellular Response To Histamine (GO:0071420)	0.1974904	0.6403329	0	0	5.361613352	8.696888062	*DHX8*
Biological Process	Cellular Response To Interleukin-9 (GO:0071355)	0.1974904	0.6403329	0	0	5.361613352	8.696888062	*STAT3*
Biological Process	Clathrin-Coated Vesicle Cargo Loading (GO:0035652)	0.1974904	0.6403329	0	0	5.361613352	8.696888062	*AP3M1*
Biological Process	Clathrin-Coated Vesicle Cargo Loading, AP-3-mediated (GO:0035654)	0.1974904	0.6403329	0	0	5.361613352	8.696888062	*AP3M1*
Biological Process	Response To Hepatocyte Growth Factor (GO:0035728)	0.1974904	0.6403329	0	0	5.361613352	8.696888062	*APPL1*

Biological Process	Definitive Hemopoiesis (GO:0060216)	0.1974904	0.6403329	0	0	5.361613352	8.696888062	*HIPK1*
Biological Process	Dense Core Granule Cytoskeletal Transport (GO:0099519)	0.1974904	0.6403329	0	0	5.361613352	8.696888062	*SYT4*
Biological Process	Detection Of Stimulus Involved In Sensory Perception Of Pain (GO:0062149)	0.1974904	0.6403329	0	0	5.361613352	8.696888062	*EPHB1*
Biological Process	Retinal Rod Cell Differentiation (GO:0060221)	0.1974904	0.6403329	0	0	5.361613352	8.696888062	*RPGRIP1L*
Biological Process	Dorsal Aorta Development (GO:0035907)	0.1974904	0.6403329	0	0	5.361613352	8.696888062	*HEY1*
Biological Process	Dorsal Aorta Morphogenesis (GO:0035912)	0.1974904	0.6403329	0	0	5.361613352	8.696888062	*HEY1*
Biological Process	Skeletal Muscle Cell Differentiation (GO:0035914)	0.1974904	0.6403329	0	0	5.361613352	8.696888062	*SIX4*
Biological Process	Skeletal Muscle Thin Filament Assembly (GO:0030240)	0.1974904	0.6403329	0	0	5.361613352	8.696888062	*TTN*
Biological Process	Synaptic Vesicle Budding From Presynaptic Endocytic Zone Membrane (GO:0016185)	0.1974904	0.6403329	0	0	5.361613352	8.696888062	*SNAP91*
Biological Process	tRNA 3'-End Processing (GO:0042780)	0.1974904	0.6403329	0	0	5.361613352	8.696888062	*SSB*
Biological Process	Fatty Acid Elongation, Monounsaturated Fatty Acid (GO:0034625)	0.1974904	0.6403329	0	0	5.361613352	8.696888062	*ELOVL4*
Biological Process	Fatty Acid Elongation, Polyunsaturated Fatty Acid (GO:0034626)	0.1974904	0.6403329	0	0	5.361613352	8.696888062	*ELOVL4*
Biological Process	Fatty Acid Elongation, Saturated Fatty Acid (GO:0019367)	0.1974904	0.6403329	0	0	5.361613352	8.696888062	*ELOVL4*
Biological Process	Fatty Acid Elongation, Unsaturated Fatty Acid (GO:0019368)	0.1974904	0.6403329	0	0	5.361613352	8.696888062	*ELOVL4*
Biological Process	Heart Trabecula Formation (GO:0060347)	0.1974904	0.6403329	0	0	5.361613352	8.696888062	*HEY1*
Biological Process	Vesicle Fusion With Golgi Apparatus (GO:0048280)	0.1974904	0.6403329	0	0	5.361613352	8.696888062	*YIPF5*
Biological Process	Vitamin A Metabolic Process (GO:0006776)	0.1974904	0.6403329	0	0	5.361613352	8.696888062	*ARID4A*
Biological Process	Hindlimb Morphogenesis (GO:0035137)	0.1974904	0.6403329	0	0	5.361613352	8.696888062	*OSR2*
Biological Process	Histidine Catabolic Process (GO:0006548)	0.1974904	0.6403329	0	0	5.361613352	8.696888062	*CARNMT1*
Biological Process	Histidine Metabolic Process (GO:0006547)	0.1974904	0.6403329	0	0	5.361613352	8.696888062	*CARNMT1*
Biological Process	Zinc Ion Import Into Organelle (GO:0062111)	0.1974904	0.6403329	0	0	5.361613352	8.696888062	*SLC30A5*
Biological Process	Interleukin-9-Mediated Signaling Pathway (GO:0038113)	0.1974904	0.6403329	0	0	5.361613352	8.696888062	*STAT3*
Biological Process	Intraciliary Transport Involved In Cilium Assembly (GO:0035735)	0.1974904	0.6403329	0	0	5.361613352	8.696888062	*TTC26*
Biological Process	Lymphoid Progenitor Cell Differentiation (GO:0002320)	0.1974904	0.6403329	0	0	5.361613352	8.696888062	*SOX4*
Biological Process	Membrane Invagination (GO:0010324)	0.1974904	0.6403329	0	0	5.361613352	8.696888062	*SMURF1*
Biological Process	Negative Regulation Of Catecholamine Secretion (GO:0033604)	0.1974904	0.6403329	0	0	5.361613352	8.696888062	*SYT4*
Biological Process	Negative Regulation Of Glycogen Metabolic Process (GO:0070874)	0.1974904	0.6403329	0	0	5.361613352	8.696888062	*GSK3B*
Biological Process	Negative Regulation Of Hematopoietic Progenitor Cell Differentiation (GO:1901533)	0.1974904	0.6403329	0	0	5.361613352	8.696888062	*N4BP2L2*
Biological Process	Negative Regulation Of Low-Density Lipoprotein Receptor Activity (GO:1905598)	0.1974904	0.6403329	0	0	5.361613352	8.696888062	*ADIPOQ*
Biological Process	Negative Regulation Of Meiotic Nuclear Division (GO:0045835)	0.1974904	0.6403329	0	0	5.361613352	8.696888062	*ERCC4*
Biological Process	Negative Regulation Of miRNA Processing (GO:1903799)	0.1974904	0.6403329	0	0	5.361613352	8.696888062	*STAT3*

Biological Process	Negative Regulation Of Protein Sumoylation (GO:0033234)	0.1974904	0.6403329	0	0	5.361613352	8.696888062	*CTNNB1*
Biological Process	Negative Regulation Of Telomere Capping (GO:1904354)	0.1974904	0.6403329	0	0	5.361613352	8.696888062	*ERCC4*
Biological Process	Neurotrophin TRK Receptor Signaling Pathway (GO:0048011)	0.1974904	0.6403329	0	0	5.361613352	8.696888062	*CASP3*
Biological Process	Noradrenergic Neuron Differentiation (GO:0003357)	0.1974904	0.6403329	0	0	5.361613352	8.696888062	*SOX4*
Biological Process	Nose Development (GO:0043584)	0.1974904	0.6403329	0	0	5.361613352	8.696888062	*SIX4*
Biological Process	Nucleotide Catabolic Process (GO:0009166)	0.1974904	0.6403329	0	0	5.361613352	8.696888062	*VCP*
Biological Process	Nucleotide Transport (GO:0006862)	0.1974904	0.6403329	0	0	5.361613352	8.696888062	*SLC25A36*
Biological Process	Peptide Transport (GO:0015833)	0.1974904	0.6403329	0	0	5.361613352	8.696888062	*CD209*
Biological Process	Placenta Blood Vessel Development (GO:0060674)	0.1974904	0.6403329	0	0	5.361613352	8.696888062	*HEY1*
Biological Process	Positive Regulation Of Endoplasmic Reticulum Stress-Induced Intrinsic Apoptotic Signaling Pathway (GO:1902237)	0.1974904	0.6403329	0	0	5.361613352	8.696888062	*NCK1*
Biological Process	Positive Regulation Of Fibroblast Apoptotic Process (GO:2000271)	0.1974904	0.6403329	0	0	5.361613352	8.696888062	*STK17B*
Biological Process	Positive Regulation Of Lymphocyte Apoptotic Process (GO:0070230)	0.1974904	0.6403329	0	0	5.361613352	8.696888062	*FNIP1*
Biological Process	Positive Regulation Of Pinocytosis (GO:0048549)	0.1974904	0.6403329	0	0	5.361613352	8.696888062	*APPL1*
Biological Process	Positive Regulation Of Protein K63-linked Ubiquitination (GO:1902523)	0.1974904	0.6403329	0	0	5.361613352	8.696888062	*DDX3X*
Biological Process	Positive Regulation Of Protein Localization To Centrosome (GO:1904781)	0.1974904	0.6403329	0	0	5.361613352	8.696888062	*GSK3B*
Biological Process	Protein Catabolic Process In The Vacuole (GO:0007039)	0.1974904	0.6403329	0	0	5.361613352	8.696888062	*VPS13A*
Biological Process	Protein Import Into Peroxisome Matrix, Receptor Recycling (GO:0016562)	0.1974904	0.6403329	0	0	5.361613352	8.696888062	*USP9X*
Biological Process	Protein Localization To Basolateral Plasma Membrane (GO:1903361)	0.1974904	0.6403329	0	0	5.361613352	8.696888062	*LIN7C*
Biological Process	Protein Localization To Endoplasmic Reticulum Exit Site (GO:0070973)	0.1974904	0.6403329	0	0	5.361613352	8.696888062	*MIA3*
Biological Process	protein-DNA Covalent Cross-Linking Repair (GO:0106300)	0.1974904	0.6403329	0	0	5.361613352	8.696888062	*VCP*
Biological Process	Pyrimidine Nucleotide-Sugar Transmembrane Transport (GO:0090481)	0.1974904	0.6403329	0	0	5.361613352	8.696888062	*SLC35D1*
Biological Process	Regulation Of Rab Protein Signal Transduction (GO:0032483)	0.1974904	0.6403329	0	0	5.361613352	8.696888062	*DENND4C*
Biological Process	Regulation Of TORC2 Signaling (GO:1903939)	0.1974904	0.6403329	0	0	5.361613352	8.696888062	*USP9X*
Biological Process	Regulation Of Axon Guidance (GO:1902667)	0.1974904	0.6403329	0	0	5.361613352	8.696888062	*YTHDF1*
Biological Process	Regulation Of Cellular Response To Transforming Growth Factor Beta Stimulus (GO:1903844)	0.2000609	0.6430279	0	0	2.554582836	4.11066453	*ZNF703;NREP*
Biological Process	Regulation Of Lipid Catabolic Process (GO:0050994)	0.2000609	0.6430279	0	0	2.554582836	4.11066453	*PRKAA1;PDE3B*
Biological Process	Cell-Cell Signaling By Wnt (GO:0198738)	0.2000609	0.6430279	0	0	2.554582836	4.11066453	*DDX3X;LRP6*
Biological Process	Cellular Response To Interleukin-6 (GO:0071354)	0.2000609	0.6430279	0	0	2.554582836	4.11066453	*FER;STAT3*
Biological Process	Cytosolic Pattern Recognition Receptor Signaling Pathway (GO:0002753)	0.2000609	0.6430279	0	0	2.554582836	4.11066453	*TRAF6;TIFA*

Biological Process	Negative Regulation Of Extrinsic Apoptotic Signaling Pathway Via Death Domain Receptors (GO:1902042)	0.2000609	0.6430279	0	0	2.554582836	4.11066453	*GSK3B;DDX3X*
Biological Process	Positive Regulation Of Oxidoreductase Activity (GO:0051353)	0.2000609	0.6430279	0	0	2.554582836	4.11066453	*PDP2;PPM1H*
Biological Process	Ephrin Receptor Signaling Pathway (GO:0048013)	0.2009917	0.6430279	0	0	2.012552301	3.22912294	*RASA1;ARHGEF7;EPHB1*
Biological Process	Glycolipid Biosynthetic Process (GO:0009247)	0.2009917	0.6430279	0	0	2.012552301	3.22912294	*FUT9;SLC30A5;ST3GAL5*
Biological Process	Lysosome Organization (GO:0007040)	0.2009917	0.6430279	0	0	2.012552301	3.22912294	*CLVS2;HOOK3;TPCN2*
Biological Process	mRNA Catabolic Process (GO:0006402)	0.2009917	0.6430279	0	0	2.012552301	3.22912294	*AGO3;XRN1;DCP2*
Biological Process	Regulation Of Angiogenesis (GO:0045765)	0.205714	0.6436057	0	0	1.378475984	2.179740447	*ATF2;PDPK1;PDE3B;FOXJ2;CTNNB1;SPRY2;HIPK1;CXCL13;MTDH;GTF2I*
Biological Process	Cellular Response To Light Stimulus (GO:0071482)	0.2060581	0.6436057	0	0	1.76014287	2.780316808	*YY1;ERCC4;DHX36;EP300*
Biological Process	Positive Regulation Of Biosynthetic Process (GO:0009891)	0.2103677	0.6436057	0	0	1.963363609	3.060684488	*CREB1;SUCO;SORBS1*
Biological Process	Positive Regulation Of Leukocyte Cell-Cell Adhesion (GO:1903039)	0.2103677	0.6436057	0	0	1.963363609	3.060684488	*NFAT5;TRAF6;CD47*
Biological Process	Regulation Of DNA Recombination (GO:0000018)	0.2103677	0.6436057	0	0	1.963363609	3.060684488	*RTEL1;SMARCAD1;TERF2IP*
Biological Process	Regulation Of Cell-Substrate Adhesion (GO:0010810)	0.2103677	0.6436057	0	0	1.963363609	3.060684488	*ATXN3;SPOCK1;PIK3CB*
Biological Process	ERK1 And ERK2 Cascade (GO:0070371)	0.213372	0.6436057	0	0	2.43833882	3.766546197	*DUSP5;MAPK1*
Biological Process	RNA Splicing, Via Transesterification Reactions (GO:0000375)	0.213372	0.6436057	0	0	2.43833882	3.766546197	*GEMIN2;SRSF10*
Biological Process	Regulation Of Neuroblast Proliferation (GO:1902692)	0.213372	0.6436057	0	0	2.43833882	3.766546197	*FZD3;CTNNB1*
Biological Process	Regulation Of Proteolysis Involved In Protein Catabolic Process (GO:1903050)	0.213372	0.6436057	0	0	2.43833882	3.766546197	*NUB1;UBE3A*
Biological Process	Early Endosome To Late Endosome Transport (GO:0045022)	0.213372	0.6436057	0	0	2.43833882	3.766546197	*HOOK3;ATG14*
Biological Process	Gliogenesis (GO:0042063)	0.213372	0.6436057	0	0	2.43833882	3.766546197	*RELN;NFIB*
Biological Process	Photoperiodism (GO:0009648)	0.213372	0.6436057	0	0	2.43833882	3.766546197	*FBXL3;CLOCK*
Biological Process	Positive Regulation Of Lipid Metabolic Process (GO:0045834)	0.213372	0.6436057	0	0	2.43833882	3.766546197	*CREB1;SORBS1*
Biological Process	Protein K48-linked Deubiquitination (GO:0071108)	0.213372	0.6436057	0	0	2.43833882	3.766546197	*USP8;USP37*
Biological Process	Protein Exit From Endoplasmic Reticulum (GO:0032527)	0.213372	0.6436057	0	0	2.43833882	3.766546197	*VCP;SURF4*
Biological Process	Negative Regulation Of Cell Differentiation (GO:0045596)	0.2139269	0.6436057	0	0	1.364338314	2.103974471	*ANKRD26;HEY1;BCL7A;ADIPOQ;HNRNPU;SPRY2;SPRY1;CNTN4;SOX4;RUNX1T1*
Biological Process	Small GTPase Mediated Signal Transduction (GO:0007264)	0.2166016	0.6436057	0	0	1.520057079	2.325224415	*USP8;SOS1;RASGRP1;RAPGEF6;CRKL;RALGPS2*
Biological Process	ERAD Pathway (GO:0036503)	0.2171171	0.6436057	0	0	1.598068598	2.440759366	*USP13;ATXN3;VCP;JKAMP;FBXO6*
Biological Process	I-kappaB kinase/NF-kappaB Signaling (GO:0007249)	0.2198372	0.6436057	0	0	1.916517235	2.903270936	*TRAF6;TIFA;BCL10*
Biological Process	Regulation Of Stem Cell Differentiation (GO:2000736)	0.2198372	0.6436057	0	0	1.916517235	2.903270936	*RBBP4;PRKDC;HNRNPU*
Biological Process	Natural Killer Cell Activation (GO:0030101)	0.2198372	0.6436057	0	0	1.916517235	2.903270936	*NFIL3;SNX27;RASGRP1*
Biological Process	Extrinsic Apoptotic Signaling Pathway (GO:0097191)	0.2212574	0.6436057	0	0	1.704087967	2.570494896	*GSK3B;DDX3X;PDPK1;HIPK1*
Biological Process	Negative Regulation Of Cellular Catabolic Process (GO:0031330)	0.2212574	0.6436057	0	0	1.704087967	2.570494896	*RRAGD;STAT3;MTMR9;FOXK1*
Biological Process	Regulation Of Cell Migration (GO:0030334)	0.2216341	0.6436057	0	0	1.232095286	1.856431865	*YTHDF3;ZMYND8;STAT3;ADIPOQ;ARID4A;MIA3;ARID4B;ING2;FER;RELN;RBBP4;ZNF703;SPRY2;PLXNC1;AMOTL1;ZNF268;ARPIN;SCAI;APPL1*

Biological Process	Positive Regulation Of Cellular Component Organization (GO:0051130)	0.2223863	0.6436057	0	0	1.505772402	2.263686906	*GSK3B;VCP;RELN;ABCB7;SURF4;FNIP1*
Biological Process	Negative Regulation Of Cellular Process (GO:0048523)	0.2237307	0.6436057	0	0	1.20476863	1.803915013	*PBRM1;DDX3X;CELF1;EI24;PDE3B;MIA3;OPRM1;ST7L;TFAP4;BRINP1;RBBP4;BCL7A;SERTAD2;TMEM127;GNG4;RASA1;PHF14;CTNNB1;PLXNC1;RAF1;ZNF268;SOX4;VGLL4*
Biological Process	Regulation Of Chemokine (C-C Motif) Ligand 5 Production (GO:0071649)	0.2263893	0.6436057	0	0	4.467779323	6.636881855	*DDX3X*
Biological Process	Regulation Of Cytoplasmic Translational Elongation (GO:1900247)	0.2263893	0.6436057	0	0	4.467779323	6.636881855	*CPEB3*
Biological Process	ER Overload Response (GO:0006983)	0.2263893	0.6436057	0	0	4.467779323	6.636881855	*GSK3B*
Biological Process	Regulation Of Extent Of Cell Growth (GO:0061387)	0.2263893	0.6436057	0	0	4.467779323	6.636881855	*GSK3B*
Biological Process	N-terminal Peptidyl-Methionine Acetylation (GO:0017196)	0.2263893	0.6436057	0	0	4.467779323	6.636881855	*NAA15*
Biological Process	NADH Metabolic Process (GO:0006734)	0.2263893	0.6436057	0	0	4.467779323	6.636881855	*VCP*
Biological Process	T-helper 17 Cell Lineage Commitment (GO:0072540)	0.2263893	0.6436057	0	0	4.467779323	6.636881855	*STAT3*
Biological Process	Regulation Of Leukocyte Degranulation (GO:0043300)	0.2263893	0.6436057	0	0	4.467779323	6.636881855	*FER*
Biological Process	Adenylate Cyclase-Inhibiting G Protein-Coupled Acetylcholine Receptor Signaling Pathway (GO:0007197)	0.2263893	0.6436057	0	0	4.467779323	6.636881855	*OPRM1*
Biological Process	Regulation Of Mammary Gland Epithelial Cell Proliferation (GO:0033599)	0.2263893	0.6436057	0	0	4.467779323	6.636881855	*ZNF703*
Biological Process	Regulation Of Protein Glycosylation (GO:0060049)	0.2263893	0.6436057	0	0	4.467779323	6.636881855	*ALG10B*
Biological Process	Regulation Of Protein Localization To Cilium (GO:1903564)	0.2263893	0.6436057	0	0	4.467779323	6.636881855	*GSK3B*
Biological Process	Cardiac Muscle Hypertrophy (GO:0003300)	0.2263893	0.6436057	0	0	4.467779323	6.636881855	*TTN*
Biological Process	Regulation Of Stem Cell Division (GO:2000035)	0.2263893	0.6436057	0	0	4.467779323	6.636881855	*MLLT3*
Biological Process	Cellular Response To Interleukin-2 (GO:0071352)	0.2263893	0.6436057	0	0	4.467779323	6.636881855	*STAT3*
Biological Process	Cellular Response To Leptin Stimulus (GO:0044320)	0.2263893	0.6436057	0	0	4.467779323	6.636881855	*STAT3*
Biological Process	Regulation Of Wound Healing, Spreading Of Epidermal Cells (GO:1903689)	0.2263893	0.6436057	0	0	4.467779323	6.636881855	*CLASP2*
Biological Process	siRNA Processing (GO:0030422)	0.2263893	0.6436057	0	0	4.467779323	6.636881855	*TSN*
Biological Process	tRNA Transcription By RNA Polymerase III (GO:0042797)	0.2263893	0.6436057	0	0	4.467779323	6.636881855	*GTF3C4*
Biological Process	Folic Acid Transport (GO:0015884)	0.2263893	0.6436057	0	0	4.467779323	6.636881855	*SLC25A32*
Biological Process	Transcription Initiation At RNA Polymerase I Promoter (GO:0006361)	0.2263893	0.6436057	0	0	4.467779323	6.636881855	*RRN3*
Biological Process	Growth Hormone Receptor Signaling Pathway Via JAK-STAT (GO:0060397)	0.2263893	0.6436057	0	0	4.467779323	6.636881855	*STAT3*
Biological Process	Ventricular Cardiac Muscle Cell Membrane Repolarization (GO:0099625)	0.2263893	0.6436057	0	0	4.467779323	6.636881855	*KCNJ3*
Biological Process	Hyperosmotic Response (GO:0006972)	0.2263893	0.6436057	0	0	4.467779323	6.636881855	*NFAT5*
Biological Process	Inhibition Of Cysteine-Type Endopeptidase Activity (GO:0097340)	0.2263893	0.6436057	0	0	4.467779323	6.636881855	*XIAP*
Biological Process	Interleukin-2-Mediated Signaling Pathway (GO:0038110)	0.2263893	0.6436057	0	0	4.467779323	6.636881855	*STAT3*

Biological Process	Kidney Mesenchyme Development (GO:0072074)	0.2263893	0.6436057	0	0	4.467779323	6.636881855	*SIX4*
Biological Process	Mammary Gland Epithelial Cell Differentiation (GO:0060644)	0.2263893	0.6436057	0	0	4.467779323	6.636881855	*ZNF703*
Biological Process	Mitral Valve Development (GO:0003174)	0.2263893	0.6436057	0	0	4.467779323	6.636881855	*SOX4*
Biological Process	Natural Killer Cell Differentiation (GO:0001779)	0.2263893	0.6436057	0	0	4.467779323	6.636881855	*NFIL3*
Biological Process	Negative Regulation Of B Cell Apoptotic Process (GO:0002903)	0.2263893	0.6436057	0	0	4.467779323	6.636881855	*BCL10*
Biological Process	Negative Regulation Of Amyloid-Beta Clearance (GO:1900222)	0.2263893	0.6436057	0	0	4.467779323	6.636881855	*HMGCR*
Biological Process	Negative Regulation Of cAMP-dependent Protein Kinase Activity (GO:2000480)	0.2263893	0.6436057	0	0	4.467779323	6.636881855	*PRKAR2A*
Biological Process	Negative Regulation Of Inflammatory Response To Wounding (GO:0106015)	0.2263893	0.6436057	0	0	4.467779323	6.636881855	*STAT3*
Biological Process	Negative Regulation Of Neural Precursor Cell Proliferation (GO:2000178)	0.2263893	0.6436057	0	0	4.467779323	6.636881855	*KDM2B*
Biological Process	Neuroepithelial Cell Differentiation (GO:0060563)	0.2263893	0.6436057	0	0	4.467779323	6.636881855	*SOX4*
Biological Process	Peptidyl-Lysine Hydroxylation (GO:0017185)	0.2263893	0.6436057	0	0	4.467779323	6.636881855	*P3H4*
Biological Process	Positive Regulation Of Arp2/3 Complex-Mediated Actin Nucleation (GO:2000601)	0.2263893	0.6436057	0	0	4.467779323	6.636881855	*WASF3*
Biological Process	Positive Regulation Of DNA Demethylation (GO:1901537)	0.2263893	0.6436057	0	0	4.467779323	6.636881855	*USP9X*
Biological Process	Positive Regulation Of T-helper 2 Cell Cytokine Production (GO:2000553)	0.2263893	0.6436057	0	0	4.467779323	6.636881855	*DENND1B*
Biological Process	Positive Regulation Of Adipose Tissue Development (GO:1904179)	0.2263893	0.6436057	0	0	4.467779323	6.636881855	*PRKAA1*
Biological Process	Positive Regulation Of Attachment Of Mitotic Spindle Microtubules To Kinetochore (GO:1902425)	0.2263893	0.6436057	0	0	4.467779323	6.636881855	*HNRNPU*
Biological Process	Positive Regulation Of Epithelial Tube Formation (GO:1905278)	0.2263893	0.6436057	0	0	4.467779323	6.636881855	*SIX4*
Biological Process	Positive Regulation Of Extracellular Matrix Disassembly (GO:0090091)	0.2263893	0.6436057	0	0	4.467779323	6.636881855	*CLASP2*
Biological Process	Positive Regulation Of Glial Cell Migration (GO:1903977)	0.2263893	0.6436057	0	0	4.467779323	6.636881855	*CRKL*
Biological Process	Positive Regulation Of Gluconeogenesis (GO:0045722)	0.2263893	0.6436057	0	0	4.467779323	6.636881855	*PPARGC1A*
Biological Process	Positive Regulation Of Insulin-Like Growth Factor Receptor Signaling Pathway (GO:0043568)	0.2263893	0.6436057	0	0	4.467779323	6.636881855	*PHIP*
Biological Process	Positive Regulation Of Isomerase Activity (GO:0010912)	0.2263893	0.6436057	0	0	4.467779323	6.636881855	*HNRNPU*
Biological Process	Positive Regulation Of Leukocyte Differentiation (GO:1902107)	0.2263893	0.6436057	0	0	4.467779323	6.636881855	*PRKDC*
Biological Process	Positive Regulation Of Membrane Potential (GO:0045838)	0.2263893	0.6436057	0	0	4.467779323	6.636881855	*VCP*
Biological Process	Positive Regulation Of Myeloid Cell Apoptotic Process (GO:0033034)	0.2263893	0.6436057	0	0	4.467779323	6.636881855	*PIK3CB*

Biological Process	Post-Translational Protein Targeting To Membrane, Translocation (GO:0031204)	0.2263893	0.6436057	0	0	4.467779323	6.636881855	*SEC61A2*
Biological Process	Postsynapse Organization (GO:0099173)	0.2263893	0.6436057	0	0	4.467779323	6.636881855	*ARHGAP39*
Biological Process	Protein K27-linked Ubiquitination (GO:0044314)	0.2263893	0.6436057	0	0	4.467779323	6.636881855	*UBE2D4*
Biological Process	Protein Desumoylation (GO:0016926)	0.2263893	0.6436057	0	0	4.467779323	6.636881855	*SENP1*
Biological Process	Protein Deubiquitination Involved In Ubiquitin-Dependent Protein Catabolic Process (GO:0071947)	0.2263893	0.6436057	0	0	4.467779323	6.636881855	*USP9X*
Biological Process	Protein Localization To Ciliary Transition Zone (GO:1904491)	0.2263893	0.6436057	0	0	4.467779323	6.636881855	*TCTN2*
Biological Process	Protein Localization To Nucleolus (GO:1902570)	0.2263893	0.6436057	0	0	4.467779323	6.636881855	*RPF2*
Biological Process	protein-RNA Complex Disassembly (GO:0032988)	0.2263893	0.6436057	0	0	4.467779323	6.636881855	*VCP*
Biological Process	Regulation Of Attachment Of Mitotic Spindle Microtubules To Kinetochore (GO:1902423)	0.2263893	0.6436057	0	0	4.467779323	6.636881855	*HNRNPU*
Biological Process	Regulation Of Double-Strand Break Repair Via Nonhomologous End Joining (GO:2001032)	0.2267626	0.6436057	0	0	2.332202979	3.460643236	*PRKDC;KMT5B*
Biological Process	Regulation Of Glycogen Biosynthetic Process (GO:0005979)	0.2267626	0.6436057	0	0	2.332202979	3.460643236	*GSK3B;SORBS1*
Biological Process	Regulation Of Hippo Signaling (GO:0035330)	0.2267626	0.6436057	0	0	2.332202979	3.460643236	*MARK3;VGLL4*
Biological Process	Regulation Of Keratinocyte Proliferation (GO:0010837)	0.2267626	0.6436057	0	0	2.332202979	3.460643236	*EPB41L4B;CASK*
Biological Process	Cardiac Conduction System Development (GO:0003161)	0.2267626	0.6436057	0	0	2.332202979	3.460643236	*HEY1;SMAD5*
Biological Process	Cellular Response To Steroid Hormone Stimulus (GO:0071383)	0.2267626	0.6436057	0	0	2.332202979	3.460643236	*MAPK1;ZDHHC7*
Biological Process	Secretory Granule Organization (GO:0033363)	0.2267626	0.6436057	0	0	2.332202979	3.460643236	*AP3M1;AP1G1*
Biological Process	Vesicle Cargo Loading (GO:0035459)	0.2267626	0.6436057	0	0	2.332202979	3.460643236	*MIA3;SEC23B*
Biological Process	Vesicle-Mediated Transport Between Endosomal Compartments (GO:0098927)	0.2267626	0.6436057	0	0	2.332202979	3.460643236	*HOOK3;ATG14*
Biological Process	Metanephros Development (GO:0001656)	0.2267626	0.6436057	0	0	2.332202979	3.460643236	*OSR2;SIX4*
Biological Process	Negative Regulation Of Response To Wounding (GO:1903035)	0.2267626	0.6436057	0	0	2.332202979	3.460643236	*STAT3;CASK*
Biological Process	Negative Regulation Of Stress Fiber Assembly (GO:0051497)	0.2267626	0.6436057	0	0	2.332202979	3.460643236	*PPFIA1;CLASP2*
Biological Process	Positive Regulation Of Glucose Import (GO:0046326)	0.2267626	0.6436057	0	0	2.332202979	3.460643236	*SORBS1;APPL1*
Biological Process	Regulation Of Nervous System Development (GO:0051960)	0.229391	0.6436057	0	0	1.871849762	2.75597529	*HES7;HEY1;CTNNB1*
Biological Process	Cardiac Conduction (GO:0061337)	0.229391	0.6436057	0	0	1.871849762	2.75597529	*MEF2A;CACNA1C;KCNJ3*
Biological Process	Negative Regulation Of Cold-Induced Thermogenesis (GO:0120163)	0.229391	0.6436057	0	0	1.871849762	2.75597529	*ADIPOQ;LNPEP;LGR4*
Biological Process	Regulation Of Epithelial Cell Proliferation (GO:0050678)	0.2302761	0.6436057	0	0	1.560741584	2.291911874	*EPB41L4B;OSR2;EPGN;SIX4;PRKDC*
Biological Process	Positive Regulation Of Binding (GO:0051099)	0.2302761	0.6436057	0	0	1.560741584	2.291911874	*GSK3B;USP9X;IDE;SPPL3;MARK3*
Biological Process	Endoplasmic Reticulum To Golgi Vesicle-Mediated Transport (GO:0006888)	0.2341052	0.6436057	0	0	1.477989361	2.146017923	*CUL3;ATL2;YIPF5;MIA3;TMED4;BET1*

Biological Process	Cellular Response To Calcium Ion (GO:0071277)	0.23674	0.6436057	0	0	1.651482596	2.379444337	*MEF2A;SYT4;PRKAA1;PRKAA2*
Biological Process	Establishment Of Protein Localization To Membrane (GO:0090150)	0.23674	0.6436057	0	0	1.651482596	2.379444337	*GORASP1;RAB3GAP2;ZDHHC7;ZDHHC21*
Biological Process	Positive Regulation Of Neuron Projection Development (GO:0010976)	0.2369404	0.6436057	0	0	1.542721646	2.221436591	*RELN;FUT9;DHX36;ZNF804A;EP300*
Biological Process	Epithelial To Mesenchymal Transition (GO:0001837)	0.2390203	0.6436057	0	0	1.829212628	2.61798187	*GSK3B;HEY1;CTNNB1*
Biological Process	Positive Regulation Of Smooth Muscle Cell Proliferation (GO:0048661)	0.2390203	0.6436057	0	0	1.829212628	2.61798187	*DNMT1;FOXJ2;MEF2D*
Biological Process	Regulation Of Epidermal Growth Factor-Activated Receptor Activity (GO:0007176)	0.2402097	0.6436057	0	0	2.234911792	3.187526837	*EPGN;CBL*
Biological Process	Regulation Of Interferon-Alpha Production (GO:0032647)	0.2402097	0.6436057	0	0	2.234911792	3.187526837	*DDX3X;DHX36*
Biological Process	Wnt Signaling Pathway, Planar Cell Polarity Pathway (GO:0060071)	0.2402097	0.6436057	0	0	2.234911792	3.187526837	*FZD3;SMURF1*
Biological Process	Regulation Of Vasoconstriction (GO:0019229)	0.2402097	0.6436057	0	0	2.234911792	3.187526837	*AVPR1B;ZDHHC21*
Biological Process	Meiosis I (GO:0007127)	0.2402097	0.6436057	0	0	2.234911792	3.187526837	*ING2;FOXJ2*
Biological Process	Negative Regulation Of Lipid Metabolic Process (GO:0045833)	0.2402097	0.6436057	0	0	2.234911792	3.187526837	*PRKAA1;PDE3B*
Biological Process	Organelle Fusion (GO:0048284)	0.2402097	0.6436057	0	0	2.234911792	3.187526837	*CHCHD3;STX3*
Biological Process	Organelle Membrane Fusion (GO:0090174)	0.2402097	0.6436057	0	0	2.234911792	3.187526837	*SYT4;STX3*
Biological Process	Positive Regulation Of Adenylate Cyclase Activity (GO:0045762)	0.2402097	0.6436057	0	0	2.234911792	3.187526837	*CACNA1C;RAF1*
Biological Process	Positive Regulation Of Erythrocyte Differentiation (GO:0045648)	0.2402097	0.6436057	0	0	2.234911792	3.187526837	*PRKDC;STAT3*
Biological Process	Positive Regulation Of Nuclear Division (GO:0051785)	0.2402097	0.6436057	0	0	2.234911792	3.187526837	*EPGN;PHIP*
Biological Process	Protein Export From Nucleus (GO:0006611)	0.2402097	0.6436057	0	0	2.234911792	3.187526837	*SMURF1;CSE1L*
Biological Process	Protein Localization To Cell Surface (GO:0034394)	0.2402097	0.6436057	0	0	2.234911792	3.187526837	*SMURF1;CTNNB1*
Biological Process	Positive Regulation Of Macromolecule Metabolic Process (GO:0010604)	0.2408894	0.6436057	0	0	1.236415883	1.759935985	*ATF2;OSR2;GSK3B;DNMT1;PRKAA1;DDX3X;CELF1;STAT3;ZNF804A;PIK3CB;HCFC1;TTN;EPB41L4B;DHX36;SPRY2;PPARGC1A*
Biological Process	Negative Regulation Of Amide Metabolic Process (GO:0034249)	0.2436575	0.6436057	0	0	1.525111252	2.153444696	*DDX3X;RTN1;DHX36;EIF4E2;CPEB3*
Biological Process	Negative Regulation Of Extrinsic Apoptotic Signaling Pathway (GO:2001237)	0.2445757	0.6436057	0	0	1.626375487	2.29031132	*GSK3B;DDX3X;EYA3;PHIP*
Biological Process	Regulation Of Notch Signaling Pathway (GO:0008593)	0.2445757	0.6436057	0	0	1.626375487	2.29031132	*POGLUT1;HEY1;STAT3;TSPAN5*
Biological Process	Regulation Of Cellular Biosynthetic Process (GO:0031326)	0.2445757	0.6436057	0	0	1.626375487	2.29031132	*SIRT5;NOLC1;MSI2;CD47*
Biological Process	Cellular Response To Oxidative Stress (GO:0034599)	0.2460104	0.6436057	0	0	1.45120751	2.03514652	*FER;PRKAA1;PRKAA2;MAPK1;PPARGC1A;CAMKK2*
Biological Process	Cellular Response To Radiation (GO:0071478)	0.2487161	0.6436057	0	0	1.788470479	2.488555421	*ERCC4;INTS7;CLOCK*
Biological Process	Retrograde Transport, Endosome To Golgi (GO:0042147)	0.2504243	0.6436057	0	0	1.507896598	2.087831651	*VPS50;SURF4;PLEKHA3;TBC1D23;EVI5*
Biological Process	Vesicle Organization (GO:0016050)	0.2504243	0.6436057	0	0	1.507896598	2.087831651	*USP8;ARFGEF2;SYT4;PLEKHA3;HOOK3*
Biological Process	Regulation Of Small GTPase Mediated Signal Transduction (GO:0051056)	0.2520279	0.6436057	0	0	1.43817527	1.982115459	*ARHGEF12;RELN;ITSN1;ARHGAP29;ARHGAP39;ARHGAP5*
Biological Process	Cell-Cell Junction Assembly (GO:0007043)	0.2524682	0.6436057	0	0	1.602017844	2.205129525	*CLDN12;ZNF703;CTNNB1;PKN2*
Biological Process	Lipid Biosynthetic Process (GO:0008610)	0.2524682	0.6436057	0	0	1.602017844	2.205129525	*PRKAA1;PRKAA2;ELOVL4;HMGCR*

Biological Process	Protein Autoubiquitination (GO:0051865)	0.2524682	0.6436057	0	0	1.602017844	2.205129525	*TRAF6;CUL3;RNF8;UBE3A*
Biological Process	Cell Surface Receptor Signaling Pathway Involved In Cell-Cell Signaling (GO:1905114)	0.2536921	0.6436057	0	0	2.1454039	2.942709093	*DDX3X;LRP6*
Biological Process	Cellular Response To Acid Chemical (GO:0071229)	0.2536921	0.6436057	0	0	2.1454039	2.942709093	*RRAGD;CPEB3*
Biological Process	Regulation Of Transcription Regulatory Region DNA Binding (GO:2000677)	0.2536921	0.6436057	0	0	2.1454039	2.942709093	*HEY1;TRAF6*
Biological Process	Clathrin-Dependent Endocytosis (GO:0072583)	0.2536921	0.6436057	0	0	2.1454039	2.942709093	*ITSN1;SNAP91*
Biological Process	Ventricular Septum Morphogenesis (GO:0060412)	0.2536921	0.6436057	0	0	2.1454039	2.942709093	*HEY1;SOX4*
Biological Process	Negative Regulation Of NIK/NF-kappaB Signaling (GO:1901223)	0.2536921	0.6436057	0	0	2.1454039	2.942709093	*PPM1A;DDX3X*
Biological Process	Negative Regulation Of Neurogenesis (GO:0050768)	0.2536921	0.6436057	0	0	2.1454039	2.942709093	*GORASP1;HOOK3*
Biological Process	Positive Regulation Of Immune System Process (GO:0002684)	0.2536921	0.6436057	0	0	2.1454039	2.942709093	*MIA3;PIK3CB*
Biological Process	Positive Regulation Of Mitotic Cell Cycle (GO:0045931)	0.2536921	0.6436057	0	0	2.1454039	2.942709093	*CUL3;PKN2*
Biological Process	Positive Regulation Of Ubiquitin-Protein Transferase Activity (GO:0051443)	0.2536921	0.6436057	0	0	2.1454039	2.942709093	*DCUN1D5;BTRC*
Biological Process	Regulation Of Androgen Receptor Signaling Pathway (GO:0060765)	0.2536921	0.6436057	0	0	2.1454039	2.942709093	*EP300;PIAS2*
Biological Process	DNA Damage Response, Signal Transduction By P53 Class Mediator Resulting In Transcription Of P21 Class Mediator (GO:0006978)	0.254249	0.6436057	0	0	3.829326445	5.244036822	*TFAP4*
Biological Process	Regulation Of Endothelial Cell Development (GO:1901550)	0.254249	0.6436057	0	0	3.829326445	5.244036822	*ZDHHC21*
Biological Process	L-arginine Transmembrane Transport (GO:1903826)	0.254249	0.6436057	0	0	3.829326445	5.244036822	*SLC7A6*
Biological Process	L-fucose Catabolic Process (GO:0042355)	0.254249	0.6436057	0	0	3.829326445	5.244036822	*FUT9*
Biological Process	L-fucose Metabolic Process (GO:0042354)	0.254249	0.6436057	0	0	3.829326445	5.244036822	*FUT9*
Biological Process	Regulation Of Fibroblast Apoptotic Process (GO:2000269)	0.254249	0.6436057	0	0	3.829326445	5.244036822	*STK17B*
Biological Process	RISC Complex Assembly (GO:0070922)	0.254249	0.6436057	0	0	3.829326445	5.244036822	*AGO3*
Biological Process	Regulation Of Heme Biosynthetic Process (GO:0070453)	0.254249	0.6436057	0	0	3.829326445	5.244036822	*ABCB7*
Biological Process	T Cell Mediated Cytotoxicity (GO:0001913)	0.254249	0.6436057	0	0	3.829326445	5.244036822	*MICA*
Biological Process	T-helper 17 Cell Differentiation (GO:0072539)	0.254249	0.6436057	0	0	3.829326445	5.244036822	*STAT3*
Biological Process	UDP-N-acetylglucosamine Biosynthetic Process (GO:0006048)	0.254249	0.6436057	0	0	3.829326445	5.244036822	*GFPT1*
Biological Process	Regulation Of Lysosomal Protein Catabolic Process (GO:1905165)	0.254249	0.6436057	0	0	3.829326445	5.244036822	*USP8*
Biological Process	Adult Heart Development (GO:0007512)	0.254249	0.6436057	0	0	3.829326445	5.244036822	*MEF2D*
Biological Process	Amyloid-Beta Formation (GO:0034205)	0.254249	0.6436057	0	0	3.829326445	5.244036822	*DYRK1A*
Biological Process	Regulation Of Morphogenesis Of An Epithelium (GO:1905330)	0.254249	0.6436057	0	0	3.829326445	5.244036822	*BTBD7*

Biological Process	Apoptotic DNA Fragmentation (GO:0006309)	0.254249	0.6436057	0	0	3.829326445	5.244036822	*DFFB*
Biological Process	Regulation Of Nephron Tubule Epithelial Cell Differentiation (GO:0072182)	0.254249	0.6436057	0	0	3.829326445	5.244036822	*CTNNB1*
Biological Process	Regulation Of Nuclear Cell Cycle DNA Replication (GO:0033262)	0.254249	0.6436057	0	0	3.829326445	5.244036822	*ATRX*
Biological Process	Basic Amino Acid Transmembrane Transport (GO:1990822)	0.254249	0.6436057	0	0	3.829326445	5.244036822	*SLC7A6*
Biological Process	Regulation Of Pattern Recognition Receptor Signaling Pathway (GO:0062207)	0.254249	0.6436057	0	0	3.829326445	5.244036822	*APPL1*
Biological Process	Regulation Of Sister Chromatid Cohesion (GO:0007063)	0.254249	0.6436057	0	0	3.829326445	5.244036822	*CTNNB1*
Biological Process	Regulation Of Skeletal Muscle Cell Differentiation (GO:2001014)	0.254249	0.6436057	0	0	3.829326445	5.244036822	*UQCC2*
Biological Process	Regulation Of Tumor Necrosis Factor Superfamily Cytokine Production (GO:1903555)	0.254249	0.6436057	0	0	3.829326445	5.244036822	*CD47*
Biological Process	Commissural Neuron Axon Guidance (GO:0071679)	0.254249	0.6436057	0	0	3.829326445	5.244036822	*NFIB*
Biological Process	Response To Leptin (GO:0044321)	0.254249	0.6436057	0	0	3.829326445	5.244036822	*STAT3*
Biological Process	Response To Misfolded Protein (GO:0051788)	0.254249	0.6436057	0	0	3.829326445	5.244036822	*UBE2W*
Biological Process	Retrograde Transport, Vesicle Recycling Within Golgi (GO:0000301)	0.254249	0.6436057	0	0	3.829326445	5.244036822	*COG6*
Biological Process	Semaphorin-Plexin Signaling Pathway Involved In Axon Guidance (GO:1902287)	0.254249	0.6436057	0	0	3.829326445	5.244036822	*PLXNC1*
Biological Process	Embryonic Forelimb Morphogenesis (GO:0035115)	0.254249	0.6436057	0	0	3.829326445	5.244036822	*OSR2*
Biological Process	Skeletal Myofibril Assembly (GO:0014866)	0.254249	0.6436057	0	0	3.829326445	5.244036822	*TTN*
Biological Process	Skin Morphogenesis (GO:0043589)	0.254249	0.6436057	0	0	3.829326445	5.244036822	*AHDC1*
Biological Process	Endothelial Cell Chemotaxis (GO:0035767)	0.254249	0.6436057	0	0	3.829326445	5.244036822	*CXCL13*
Biological Process	Somatic Recombination Of Immunoglobulin Gene Segments (GO:0016447)	0.254249	0.6436057	0	0	3.829326445	5.244036822	*PRKDC*
Biological Process	Sphingosine-1-Phosphate Receptor Signaling Pathway (GO:0003376)	0.254249	0.6436057	0	0	3.829326445	5.244036822	*PIK3CB*
Biological Process	Exonucleolytic Trimming Involved In rRNA Processing (GO:0000459)	0.254249	0.6436057	0	0	3.829326445	5.244036822	*ERI2*
Biological Process	Exonucleolytic Trimming To Generate Mature 3'-End Of 5.8S rRNA From Tricistronic rRNA Transcript (SSU-rRNA, 5.8S rRNA, LSU-rRNA) (GO:0000467)	0.254249	0.6436057	0	0	3.829326445	5.244036822	*ERI2*
Biological Process	tRNA Transcription (GO:0009304)	0.254249	0.6436057	0	0	3.829326445	5.244036822	*GTF3C4*
Biological Process	Fucose Catabolic Process (GO:0019317)	0.254249	0.6436057	0	0	3.829326445	5.244036822	*FUT9*
Biological Process	Glucan Metabolic Process (GO:0044042)	0.254249	0.6436057	0	0	3.829326445	5.244036822	*GSK3B*
Biological Process	Internal Protein Amino Acid Acetylation (GO:0006475)	0.254249	0.6436057	0	0	3.829326445	5.244036822	*EP300*
Biological Process	Isoprenoid Biosynthetic Process (GO:0008299)	0.254249	0.6436057	0	0	3.829326445	5.244036822	*HMGCR*
Biological Process	Isoprenoid Metabolic Process (GO:0006720)	0.254249	0.6436057	0	0	3.829326445	5.244036822	*HMGCR*
Biological Process	Leptin-Mediated Signaling Pathway (GO:0033210)	0.254249	0.6436057	0	0	3.829326445	5.244036822	*STAT3*
Biological Process	Manganese Ion Transport (GO:0006828)	0.254249	0.6436057	0	0	3.829326445	5.244036822	*SLC11A2*

Biological Process	Multi-Pass Transmembrane Protein Insertion Into ER Membrane (GO:0160063)	0.254249	0.6436057	0	0	3.829326445	5.244036822	*NOMO3*
Biological Process	ncRNA Export From Nucleus (GO:0097064)	0.254249	0.6436057	0	0	3.829326445	5.244036822	*SSB*
Biological Process	Negative Regulation By Host Of Viral Genome Replication (GO:0044828)	0.254249	0.6436057	0	0	3.829326445	5.244036822	*SMC6*
Biological Process	Negative Regulation Of cAMP-mediated Signaling (GO:0043951)	0.254249	0.6436057	0	0	3.829326445	5.244036822	*PDE3B*
Biological Process	Negative Regulation Of Cytoplasmic Translation (GO:2000766)	0.254249	0.6436057	0	0	3.829326445	5.244036822	*CPEB3*
Biological Process	Negative Regulation Of Regulated Secretory Pathway (GO:1903306)	0.254249	0.6436057	0	0	3.829326445	5.244036822	*SYT4*
Biological Process	Nucleic Acid Transport (GO:0050657)	0.254249	0.6436057	0	0	3.829326445	5.244036822	*HNRNPA2B1*
Biological Process	Positive Regulation Of Calcium Ion-Dependent Exocytosis (GO:0045956)	0.254249	0.6436057	0	0	3.829326445	5.244036822	*SYT4*
Biological Process	Positive Regulation Of Centriole Replication (GO:0046601)	0.254249	0.6436057	0	0	3.829326445	5.244036822	*CEP120*
Biological Process	Positive Regulation Of Extracellular Matrix Assembly (GO:1901203)	0.254249	0.6436057	0	0	3.829326445	5.244036822	*CLASP2*
Biological Process	Positive Regulation Of Inositol Phosphate Biosynthetic Process (GO:0060732)	0.254249	0.6436057	0	0	3.829326445	5.244036822	*DHX8*
Biological Process	Positive Regulation Of Synapse Maturation (GO:0090129)	0.254249	0.6436057	0	0	3.829326445	5.244036822	*RELN*
Biological Process	Protein K29-linked Ubiquitination (GO:0035519)	0.254249	0.6436057	0	0	3.829326445	5.244036822	*UBE2D4*
Biological Process	Protein Localization To Perinuclear Region Of Cytoplasm (GO:1905719)	0.254249	0.6436057	0	0	3.829326445	5.244036822	*HOOK3*
Biological Process	Pyrimidine-Containing Compound Metabolic Process (GO:0072527)	0.254249	0.6436057	0	0	3.829326445	5.244036822	*SLC19A2*
Biological Process	Regulation Of DNA Demethylation (GO:1901535)	0.254249	0.6436057	0	0	3.829326445	5.244036822	*USP9X*
Biological Process	Regulation Of Wnt Signaling Pathway, Planar Cell Polarity Pathway (GO:2000095)	0.254249	0.6436057	0	0	3.829326445	5.244036822	*MLLT3*
Biological Process	T Cell Receptor Signaling Pathway (GO:0050852)	0.257238	0.6436057	0	0	1.491064491	2.024498224	*DENND1B;KHDRBS1;TRAF6;BCL10;SPPL3*
Biological Process	Intrinsic Apoptotic Signaling Pathway In Response To DNA Damage (GO:0008630)	0.2584698	0.6436057	0	0	1.749499727	2.367031717	*PRKDC;EP300;HIPK1*
Biological Process	Neurogenesis (GO:0022008)	0.2584698	0.6436057	0	0	1.749499727	2.367031717	*SIX4;SPOCK1;EPHB1*
Biological Process	Regulation Of Cellular Localization (GO:0060341)	0.2604128	0.6436057	0	0	1.578376602	2.123685549	*USP8;DNAJB6;RAB14;PARD6G*
Biological Process	Negative Regulation Of Catabolic Process (GO:0009895)	0.2604128	0.6436057	0	0	1.578376602	2.123685549	*PRKAA1;PDE3B;ZDHHC7;HMGCR*
Biological Process	Protein Targeting To Membrane (GO:0006612)	0.2604128	0.6436057	0	0	1.578376602	2.123685549	*PEX3;SSR1;ZDHHC7;ZDHHC21*
Biological Process	Regulation Of Chemotaxis (GO:0050920)	0.2671896	0.6436057	0	0	2.06278123	2.722451741	*YTHDF1;STX3*
Biological Process	RNA Phosphodiester Bond Hydrolysis (GO:0090501)	0.2671896	0.6436057	0	0	2.06278123	2.722451741	*XRN1;ZC3H12B*
Biological Process	Regulation Of Lipid Biosynthetic Process (GO:0046890)	0.2671896	0.6436057	0	0	2.06278123	2.722451741	*CREB1;SORBS1*
Biological Process	Regulation Of Nitric-Oxide Synthase Activity (GO:0050999)	0.2671896	0.6436057	0	0	2.06278123	2.722451741	*ZDHHC21;PIK3CB*
Biological Process	Regulation Of Plasma Membrane Bounded Cell Projection Organization (GO:0120035)	0.2671896	0.6436057	0	0	2.06278123	2.722451741	*GSK3B;ZNF804A*

Biological Process	Regulation Of Protein Localization To Membrane (GO:1905475)	0.2671896	0.6436057	0	0	2.06278123	2.722451741	*ZDHHC7;APPL1*
Biological Process	Cellular Response To Osmotic Stress (GO:0071470)	0.2671896	0.6436057	0	0	2.06278123	2.722451741	*NFAT5;DDX3X*
Biological Process	Transcription By RNA Polymerase III (GO:0006383)	0.2671896	0.6436057	0	0	2.06278123	2.722451741	*GTF3C4;ELL*
Biological Process	Vesicle Coating (GO:0006901)	0.2671896	0.6436057	0	0	2.06278123	2.722451741	*PPP6C;CUL3*
Biological Process	Negative Regulation Of Actin Filament Bundle Assembly (GO:0032232)	0.2671896	0.6436057	0	0	2.06278123	2.722451741	*PPFIA1;CLASP2*
Biological Process	Negative Regulation Of Signaling (GO:0023057)	0.2671896	0.6436057	0	0	2.06278123	2.722451741	*ADIPOQ;OPRM1*
Biological Process	Negative Regulation Of Wound Healing (GO:0061045)	0.2671896	0.6436057	0	0	2.06278123	2.722451741	*CASK;CLASP2*
Biological Process	Protein Localization To Golgi Apparatus (GO:0034067)	0.2671896	0.6436057	0	0	2.06278123	2.722451741	*VPS13A;ARL5A*
Biological Process	Regulation Of DNA Damage Response, Signal Transduction By P53 Class Mediator (GO:0043516)	0.2671896	0.6436057	0	0	2.06278123	2.722451741	*DYRK1A;SOX4*
Biological Process	Regulation Of Mitochondrion Organization (GO:0010821)	0.2682732	0.6436057	0	0	1.712187305	2.252809432	*GSK3B;EP300;PPARGC1A*
Biological Process	Negative Regulation Of Cell Projection Organization (GO:0031345)	0.2682732	0.6436057	0	0	1.712187305	2.252809432	*GORASP1;SPOCK1;SPRY2*
Biological Process	Positive Regulation Of Developmental Growth (GO:0048639)	0.2682732	0.6436057	0	0	1.712187305	2.252809432	*SYT4;SMURF1;PLAA*
Biological Process	Regulation Of Programmed Cell Death (GO:0043067)	0.2704716	0.6436057	0	0	1.299441949	1.699134805	*MBTD1;VCP;RBM25;ING3;APAF1;XIAP;TARDBP;RAF1;ZDHHC17*
Biological Process	Gene Expression (GO:0010467)	0.2705778	0.6436057	0	0	1.234305106	1.613478308	*FYTTD1;DHX8;DIS3;MRPS11;DDX20;HNRNPU;NXF1;ATXN1;SARNP;CASP3;HNRNPA2B1;TSPAN5;MRRF*
Biological Process	Negative Regulation Of Neuron Apoptotic Process (GO:0043524)	0.2764402	0.6436057	0	0	1.533120511	1.971226271	*SIX4;KDM2B;RASA1;PPARGC1A*
Biological Process	Regulation Of Cell Cycle G1/S Phase Transition (GO:1902806)	0.2764402	0.6436057	0	0	1.533120511	1.971226271	*PBRM1;BCL7A;ARID1A;APPL1*
Biological Process	Regulation Of Protein Catabolic Process (GO:0042176)	0.2764838	0.6436057	0	0	1.388293248	1.784793986	*GSK3B;VCP;ASB11;IDE;HMGCR;VGLL4*
Biological Process	MAPK Cascade (GO:0000165)	0.2779315	0.6436057	0	0	1.442740056	1.847256349	*MEF2A;DUSP5;MAPK1;MAP3K4;CAMKK2*
Biological Process	Positive Regulation Of Protein-Containing Complex Assembly (GO:0031334)	0.2779315	0.6436057	0	0	1.442740056	1.847256349	*GSK3B;VCP;DDX3X;CXCL13;FNIP1*
Biological Process	RNA Modification (GO:0009451)	0.278118	0.6436057	0	0	1.676429568	2.145343083	*DTWD2;SSB;TRDMT1*
Biological Process	Regulation Of Smoothened Signaling Pathway (GO:0008589)	0.278118	0.6436057	0	0	1.676429568	2.145343083	*VCP;PHIP;BTRC*
Biological Process	Regulation Of Stress-Activated MAPK Cascade (GO:0032872)	0.278118	0.6436057	0	0	1.676429568	2.145343083	*MAPK1;EPHB1;RASGRP1*
Biological Process	Negative Regulation Of MAP Kinase Activity (GO:0043407)	0.278118	0.6436057	0	0	1.676429568	2.145343083	*SPRY2;SPRY1;PIK3CB*
Biological Process	Positive Regulation Of Protein Localization To Cell Periphery (GO:1904377)	0.278118	0.6436057	0	0	1.676429568	2.145343083	*PDPK1;STX3;SORBS1*
Biological Process	Positive Regulation Of Protein Localization To Plasma Membrane (GO:1903078)	0.278118	0.6436057	0	0	1.676429568	2.145343083	*PDPK1;STX3;SORBS1*
Biological Process	Regulation Of Establishment Of Planar Polarity (GO:0090175)	0.2806839	0.6436057	0	0	1.986278758	2.523619178	*FZD3;SMURF1*
Biological Process	JNK Cascade (GO:0007254)	0.2806839	0.6436057	0	0	1.986278758	2.523619178	*MAP4K3;CRKL*
Biological Process	Regulation Of Protein Modification By Small Protein Conjugation Or Removal (GO:1903320)	0.2806839	0.6436057	0	0	1.986278758	2.523619178	*DCUN1D5;CTNNB1*
Biological Process	Regulation Of Spindle Assembly (GO:0090169)	0.2806839	0.6436057	0	0	1.986278758	2.523619178	*PDCD6IP;HNRNPU*

Biological Process	Chondrocyte Differentiation (GO:0002062)	0.2806839	0.6436057	0	0	1.986278758	2.523619178	*OSR2;NFIB*
Biological Process	Sarcomere Organization (GO:0045214)	0.2806839	0.6436057	0	0	1.986278758	2.523619178	*SIX4;TTN*
Biological Process	Modified Amino Acid Transport (GO:0072337)	0.2806839	0.6436057	0	0	1.986278758	2.523619178	*SLC7A6;SLC25A32*
Biological Process	Morphogenesis Of An Epithelium (GO:0002009)	0.2806839	0.6436057	0	0	1.986278758	2.523619178	*KDM2B;LIN7C*
Biological Process	Negative Regulation Of DNA Binding (GO:0043392)	0.2806839	0.6436057	0	0	1.986278758	2.523619178	*TFAP4;HEY1*
Biological Process	Phosphatidic Acid Biosynthetic Process (GO:0006654)	0.2806839	0.6436057	0	0	1.986278758	2.523619178	*GPAT3;LCLAT1*
Biological Process	Positive Regulation Of Dephosphorylation (GO:0035306)	0.2806839	0.6436057	0	0	1.986278758	2.523619178	*MTMR9;SPPL3*
Biological Process	Positive Regulation Of Neural Precursor Cell Proliferation (GO:2000179)	0.2806839	0.6436057	0	0	1.986278758	2.523619178	*FZD3;CTNNB1*
Biological Process	Positive Regulation Of Viral Genome Replication (GO:0045070)	0.2806839	0.6436057	0	0	1.986278758	2.523619178	*DDX3X;PKN2*
Biological Process	Protein K11-linked Ubiquitination (GO:0070979)	0.2806839	0.6436057	0	0	1.986278758	2.523619178	*UBE2W;UBE2D4*
Biological Process	DNA Damage Response, Signal Transduction Resulting In Transcription (GO:0042772)	0.2811068	0.6436057	0	0	3.350486787	4.251837124	*TFAP4*
Biological Process	ER-associated Misfolded Protein Catabolic Process (GO:0071712)	0.2811068	0.6436057	0	0	3.350486787	4.251837124	*VCP*
Biological Process	Regulation Of Hair Cycle (GO:0042634)	0.2811068	0.6436057	0	0	3.350486787	4.251837124	*CLOCK*
Biological Process	SNARE Complex Assembly (GO:0035493)	0.2811068	0.6436057	0	0	3.350486787	4.251837124	*VAMP4*
Biological Process	Regulation Of Inflammatory Response To Wounding (GO:0106014)	0.2811068	0.6436057	0	0	3.350486787	4.251837124	*STAT3*
Biological Process	Wnt Signaling Pathway Involved In Midbrain Dopaminergic Neuron Differentiation (GO:1904953)	0.2811068	0.6436057	0	0	3.350486787	4.251837124	*LRP6*
Biological Process	Regulation Of Mast Cell Activation Involved In Immune Response (GO:0033006)	0.2811068	0.6436057	0	0	3.350486787	4.251837124	*FER*
Biological Process	Amino Sugar Biosynthetic Process (GO:0046349)	0.2811068	0.6436057	0	0	3.350486787	4.251837124	*GFPT1*
Biological Process	Amino Sugar Metabolic Process (GO:0006040)	0.2811068	0.6436057	0	0	3.350486787	4.251837124	*GFPT1*
Biological Process	Regulation Of Myeloid Leukocyte Mediated Immunity (GO:0002886)	0.2811068	0.6436057	0	0	3.350486787	4.251837124	*FER*
Biological Process	Axon Regeneration (GO:0031103)	0.2811068	0.6436057	0	0	3.350486787	4.251837124	*NREP*
Biological Process	Regulation Of Phospholipid Metabolic Process (GO:1903725)	0.2811068	0.6436057	0	0	3.350486787	4.251837124	*MTMR9*
Biological Process	Calcium Ion Transport Into Cytosol (GO:0060402)	0.2811068	0.6436057	0	0	3.350486787	4.251837124	*CACNA1C*
Biological Process	Camera-Type Eye Morphogenesis (GO:0048593)	0.2811068	0.6436057	0	0	3.350486787	4.251837124	*KDM2B*
Biological Process	Regulation Of Protein Lipidation (GO:1903059)	0.2811068	0.6436057	0	0	3.350486787	4.251837124	*RAB3GAP2*
Biological Process	Regulation Of Protein Localization To Centrosome (GO:1904779)	0.2811068	0.6436057	0	0	3.350486787	4.251837124	*GSK3B*
Biological Process	Regulation Of Protein Localization To Chromatin (GO:1905634)	0.2811068	0.6436057	0	0	3.350486787	4.251837124	*VCP*
Biological Process	Regulation Of Receptor Binding (GO:1900120)	0.2811068	0.6436057	0	0	3.350486787	4.251837124	*ADIPOQ*

Biological Process	Cellular Response To Oxidised Low-Density Lipoprotein Particle Stimulus (GO:0140052)	0.2811068	0.6436057	0	0	3.350486787	4.251837124	*MIA3*
Biological Process	Regulation Of Viral-Induced Cytoplasmic Pattern Recognition Receptor Signaling Pathway (GO:0039531)	0.2811068	0.6436057	0	0	3.350486787	4.251837124	*XIAP*
Biological Process	Chloride Ion Homeostasis (GO:0055064)	0.2811068	0.6436057	0	0	3.350486787	4.251837124	*SLC12A6*
Biological Process	Response To Histamine (GO:0034776)	0.2811068	0.6436057	0	0	3.350486787	4.251837124	*DHX8*
Biological Process	Response To Platelet-Derived Growth Factor (GO:0036119)	0.2811068	0.6436057	0	0	3.350486787	4.251837124	*FER*
Biological Process	Dosage Compensation (GO:0007549)	0.2811068	0.6436057	0	0	3.350486787	4.251837124	*HNRNPU*
Biological Process	Ectoderm Development (GO:0007398)	0.2811068	0.6436057	0	0	3.350486787	4.251837124	*SMURF1*
Biological Process	Embryonic Eye Morphogenesis (GO:0048048)	0.2811068	0.6436057	0	0	3.350486787	4.251837124	*KDM2B*
Biological Process	Endothelial Tube Morphogenesis (GO:0061154)	0.2811068	0.6436057	0	0	3.350486787	4.251837124	*CTNNB1*
Biological Process	Somatic Recombination Of Immunoglobulin Genes Involved In Immune Response (GO:0002204)	0.2811068	0.6436057	0	0	3.350486787	4.251837124	*RNF8*
Biological Process	Synaptic Vesicle Fusion To Presynaptic Active Zone Membrane (GO:0031629)	0.2811068	0.6436057	0	0	3.350486787	4.251837124	*STX3*
Biological Process	Forelimb Morphogenesis (GO:0035136)	0.2811068	0.6436057	0	0	3.350486787	4.251837124	*OSR2*
Biological Process	Fructose 6-Phosphate Metabolic Process (GO:0006002)	0.2811068	0.6436057	0	0	3.350486787	4.251837124	*GFPT1*
Biological Process	Telomeric Loop Disassembly (GO:0090657)	0.2811068	0.6436057	0	0	3.350486787	4.251837124	*RTEL1*
Biological Process	Hematopoietic Stem Cell Differentiation (GO:0060218)	0.2811068	0.6436057	0	0	3.350486787	4.251837124	*MLLT3*
Biological Process	High-Density Lipoprotein Particle Assembly (GO:0034380)	0.2811068	0.6436057	0	0	3.350486787	4.251837124	*PRKACB*
Biological Process	Histone Ubiquitination (GO:0016574)	0.2811068	0.6436057	0	0	3.350486787	4.251837124	*WAC*
Biological Process	Leucine Transport (GO:0015820)	0.2811068	0.6436057	0	0	3.350486787	4.251837124	*SLC7A6*
Biological Process	Microtubule Anchoring (GO:0034453)	0.2811068	0.6436057	0	0	3.350486787	4.251837124	*CLASP2*
Biological Process	Mitotic DNA Replication Checkpoint Signaling (GO:0033314)	0.2811068	0.6436057	0	0	3.350486787	4.251837124	*ORC1*
Biological Process	Mitral Valve Morphogenesis (GO:0003183)	0.2811068	0.6436057	0	0	3.350486787	4.251837124	*SOX4*
Biological Process	Monoatomic Anion Homeostasis (GO:0055081)	0.2811068	0.6436057	0	0	3.350486787	4.251837124	*SLC12A6*
Biological Process	Morphogenesis Of An Epithelial Sheet (GO:0002011)	0.2811068	0.6436057	0	0	3.350486787	4.251837124	*LIN7C*
Biological Process	Negative Regulation Of Cell Adhesion Mediated By Integrin (GO:0033629)	0.2811068	0.6436057	0	0	3.350486787	4.251837124	*PDE3B*
Biological Process	Negative Regulation Of Muscle Cell Apoptotic Process (GO:0010656)	0.2811068	0.6436057	0	0	3.350486787	4.251837124	*LRP6*
Biological Process	Negative Regulation Of Receptor Binding (GO:1900121)	0.2811068	0.6436057	0	0	3.350486787	4.251837124	*ADIPOQ*
Biological Process	Negative Regulation Of Transcription By Competitive Promoter Binding (GO:0010944)	0.2811068	0.6436057	0	0	3.350486787	4.251837124	*CREB1*
Biological Process	Neuron Projection Regeneration (GO:0031102)	0.2811068	0.6436057	0	0	3.350486787	4.251837124	*NREP*
Biological Process	Neurotransmitter Receptor Transport To Postsynaptic Membrane (GO:0098969)	0.2811068	0.6436057	0	0	3.350486787	4.251837124	*STX3*

Biological Process	Nuclear mRNA Surveillance (GO:0071028)	0.2811068	0.6436057	0	0	3.350486787	4.251837124	*XRN1*
Biological Process	Peptidyl-Lysine Acetylation (GO:0018394)	0.2811068	0.6436057	0	0	3.350486787	4.251837124	*EP300*
Biological Process	Peptidyl-Serine Dephosphorylation (GO:0070262)	0.2811068	0.6436057	0	0	3.350486787	4.251837124	*NCK1*
Biological Process	Phosphatidylethanolamine Biosynthetic Process (GO:0006646)	0.2811068	0.6436057	0	0	3.350486787	4.251837124	*ETNK1*
Biological Process	Positive Regulation Of Keratinocyte Migration (GO:0051549)	0.2811068	0.6436057	0	0	3.350486787	4.251837124	*EPB41L4B*
Biological Process	Positive Regulation Of Leukocyte Apoptotic Process (GO:2000108)	0.2811068	0.6436057	0	0	3.350486787	4.251837124	*PIK3CB*
Biological Process	Positive Regulation Of Lipase Activity (GO:0060193)	0.2811068	0.6436057	0	0	3.350486787	4.251837124	*PDPK1*
Biological Process	Positive Regulation Of Mitochondrial Membrane Permeability (GO:0035794)	0.2811068	0.6436057	0	0	3.350486787	4.251837124	*GSK3B*
Biological Process	Positive Regulation Of Protein Kinase A Signaling (GO:0010739)	0.2811068	0.6436057	0	0	3.350486787	4.251837124	*ADIPOQ*
Biological Process	Positive Regulation Of rRNA Processing (GO:2000234)	0.2811068	0.6436057	0	0	3.350486787	4.251837124	*RIOK2*
Biological Process	Protein K11-linked Deubiquitination (GO:0035871)	0.2811068	0.6436057	0	0	3.350486787	4.251837124	*USP37*
Biological Process	Protein K6-linked Ubiquitination (GO:0085020)	0.2811068	0.6436057	0	0	3.350486787	4.251837124	*UBE2D4*
Biological Process	Protein O-linked Glycosylation Via Serine (GO:0018242)	0.2811068	0.6436057	0	0	3.350486787	4.251837124	*POGLUT1*
Biological Process	Protein Localization To Microtubule Cytoskeleton (GO:0072698)	0.2811068	0.6436057	0	0	3.350486787	4.251837124	*MID2*
Biological Process	Protein Localization To Nuclear Envelope (GO:0090435)	0.2811068	0.6436057	0	0	3.350486787	4.251837124	*TOR1AIP2*
Biological Process	Purine Nucleobase Metabolic Process (GO:0006144)	0.2811068	0.6436057	0	0	3.350486787	4.251837124	*SLC25A16*
Biological Process	rRNA 3'-End Processing (GO:0031125)	0.2811068	0.6436057	0	0	3.350486787	4.251837124	*ERI2*
Biological Process	Regulation Of ER To Golgi Vesicle-Mediated Transport (GO:0060628)	0.2811068	0.6436057	0	0	3.350486787	4.251837124	*YIPF5*
Biological Process	Regulation Of PERK-mediated Unfolded Protein Response (GO:1903897)	0.2811068	0.6436057	0	0	3.350486787	4.251837124	*NCK1*
Biological Process	Regulation Of RNA Polymerase II Regulatory Region Sequence-Specific DNA Binding (GO:1903025)	0.2811068	0.6436057	0	0	3.350486787	4.251837124	*EP300*
Biological Process	Regulation Of Animal Organ Morphogenesis (GO:2000027)	0.2811068	0.6436057	0	0	3.350486787	4.251837124	*BTBD7*
Biological Process	Regulation Of Cell-Cell Adhesion Mediated By Integrin (GO:0033632)	0.2811068	0.6436057	0	0	3.350486787	4.251837124	*CXCL13*
Biological Process	Regulation Of Cellular Extravasation (GO:0002691)	0.2811068	0.6436057	0	0	3.350486787	4.251837124	*FUT9*
Biological Process	Positive Regulation Of Neurogenesis (GO:0050769)	0.284514	0.6461364	0	0	1.51144858	1.899849838	*FZD3;CTNNB1;PLXNC1;OPRM1*
Biological Process	Regulation Of BMP Signaling Pathway (GO:0030510)	0.284514	0.6461364	0	0	1.51144858	1.899849838	*FSTL5;SMURF1;VWC2;XIAP*
Biological Process	Regulation Of DNA Replication (GO:0006275)	0.284514	0.6461364	0	0	1.51144858	1.899849838	*YY1;USP37;INO80D;BRPF3*
Biological Process	Cardiac Muscle Tissue Development (GO:0048738)	0.2879964	0.6461364	0	0	1.64213133	2.04413689	*HEY1;SMAD5;TTN*
Biological Process	Cellular Response To Ionizing Radiation (GO:0071479)	0.2879964	0.6461364	0	0	1.64213133	2.04413689	*ERCC4;INTS7;CLOCK*
Biological Process	Regulation Of DNA Metabolic Process (GO:0051052)	0.2889152	0.6461364	0	0	1.364620424	1.694342665	*YY1;USP37;INO80D;SMARCAD1;TERF2IP;SMC6*

Biological Process	Axon Guidance (GO:0007411)	0.2905018	0.6461364	0	0	1.323173261	1.635634894	*RELN;NFIB;SIAH1;PLXNC1;CNTN4;SOS1;EPHB1*
Biological Process	Regulation Of Dendritic Spine Morphogenesis (GO:0061001)	0.2941576	0.6461364	0	0	1.915240748	2.343564338	*RELN;DHX36*
Biological Process	Regulation Of Vasculature Development (GO:1901342)	0.2941576	0.6461364	0	0	1.915240748	2.343564338	*CTNNB1;CXCL13*
Biological Process	Response To Amino Acid (GO:0043200)	0.2941576	0.6461364	0	0	1.915240748	2.343564338	*RRAGD;CPEB3*
Biological Process	Response To Epidermal Growth Factor (GO:0070849)	0.2941576	0.6461364	0	0	1.915240748	2.343564338	*PDPK1;MAPK1*
Biological Process	Innate Immune Response Activating Cell Surface Receptor Signaling Pathway (GO:0002220)	0.2941576	0.6461364	0	0	1.915240748	2.343564338	*TRAF6;EP300*
Biological Process	Negative Regulation Of Carbohydrate Metabolic Process (GO:0045912)	0.2941576	0.6461364	0	0	1.915240748	2.343564338	*ADIPOQ;EP300*
Biological Process	Negative Regulation Of Epithelial To Mesenchymal Transition (GO:0010719)	0.2941576	0.6461364	0	0	1.915240748	2.343564338	*SPRY2;SPRY1*
Biological Process	Positive Regulation Of JUN Kinase Activity (GO:0043507)	0.2941576	0.6461364	0	0	1.915240748	2.343564338	*TRAF6;MAP3K4*
Biological Process	Positive Regulation Of Locomotion (GO:0040017)	0.2941576	0.6461364	0	0	1.915240748	2.343564338	*SPOCK2;STX3*
Biological Process	Positive Regulation Of Phosphate Metabolic Process (GO:0045937)	0.2941576	0.6461364	0	0	1.915240748	2.343564338	*AVPR1B;BCL10*
Biological Process	Negative Regulation Of Cell Growth (GO:0030308)	0.2951748	0.6461364	0	0	1.353082409	1.651014334	*ST7L;DDX3X;GNG4;SERTAD2;EI24;VGLL4*
Biological Process	Neuron Development (GO:0048666)	0.2962027	0.6461364	0	0	1.313851646	1.598578276	*GSK3B;PBX2P1;ETV1;TBC1D23;CNTN4;STX3;PPP1R9A*
Biological Process	Cellular Response To Alcohol (GO:0097306)	0.2979007	0.6461364	0	0	1.609205021	1.948739407	*CREB1;CTNNB1;LRP6*
Biological Process	Tight Junction Assembly (GO:0120192)	0.2979007	0.6461364	0	0	1.609205021	1.948739407	*PDCD6IP;CLDN12;CLDN18*
Biological Process	Protein Catabolic Process (GO:0030163)	0.306005	0.6461364	0	0	1.382957249	1.637634282	*VCP;CASP3;SIAH1;FBXO6;IDE*
Biological Process	CRD-mediated mRNA Stabilization (GO:0070934)	0.3069985	0.6461364	0	0	2.978055942	3.516822846	*HNRNPU*
Biological Process	Regulation Of Gene Expression By Genomic Imprinting (GO:0006349)	0.3069985	0.6461364	0	0	2.978055942	3.516822846	*DNMT3A*
Biological Process	Notch Signaling Involved In Heart Development (GO:0061314)	0.3069985	0.6461364	0	0	2.978055942	3.516822846	*HEY1*
Biological Process	SRP-dependent Cotranslational Protein Targeting To Membrane (GO:0006614)	0.3069985	0.6461364	0	0	2.978055942	3.516822846	*SEC61A2*
Biological Process	Regulation Of Inclusion Body Assembly (GO:0090083)	0.3069985	0.6461364	0	0	2.978055942	3.516822846	*DNAJB6*
Biological Process	T-helper Cell Lineage Commitment (GO:0002295)	0.3069985	0.6461364	0	0	2.978055942	3.516822846	*STAT3*
Biological Process	UDP-N-acetylglucosamine Metabolic Process (GO:0006047)	0.3069985	0.6461364	0	0	2.978055942	3.516822846	*GFPT1*
Biological Process	Regulation Of Lamellipodium Morphogenesis (GO:2000392)	0.3069985	0.6461364	0	0	2.978055942	3.516822846	*ARPIN*
Biological Process	Regulation Of Lipid Transport (GO:0032368)	0.3069985	0.6461364	0	0	2.978055942	3.516822846	*SURF4*
Biological Process	Ammonium Transmembrane Transport (GO:0072488)	0.3069985	0.6461364	0	0	2.978055942	3.516822846	*SLC12A6*
Biological Process	Atrial Septum Morphogenesis (GO:0060413)	0.3069985	0.6461364	0	0	2.978055942	3.516822846	*SOX4*
Biological Process	Branched-Chain Amino Acid Transport (GO:0015803)	0.3069985	0.6461364	0	0	2.978055942	3.516822846	*SLC7A6*
Biological Process	calcineurin-NFAT Signaling Cascade (GO:0033173)	0.3069985	0.6461364	0	0	2.978055942	3.516822846	*NFAT5*

Biological Process	Regulation Of Programmed Necrotic Cell Death (GO:0062098)	0.3069985	0.6461364	0	0	2.978055942	3.516822846	*SPATA2*
Biological Process	Cardiac Muscle Cell Membrane Repolarization (GO:0099622)	0.3069985	0.6461364	0	0	2.978055942	3.516822846	*KCNJ3*
Biological Process	Regulation Of Smooth Muscle Cell Apoptotic Process (GO:0034391)	0.3069985	0.6461364	0	0	2.978055942	3.516822846	*LRP6*
Biological Process	Cellular Response To Cholesterol (GO:0071397)	0.3069985	0.6461364	0	0	2.978055942	3.516822846	*LRP6*
Biological Process	Cellular Response To Forskolin (GO:1904322)	0.3069985	0.6461364	0	0	2.978055942	3.516822846	*CREB1*
Biological Process	Regulation Of Synapse Maturation (GO:0090128)	0.3069985	0.6461364	0	0	2.978055942	3.516822846	*RELN*
Biological Process	Cellular Response To Leucine (GO:0071233)	0.3069985	0.6461364	0	0	2.978055942	3.516822846	*RRAGD*
Biological Process	Coenzyme A Biosynthetic Process (GO:0015937)	0.3069985	0.6461364	0	0	2.978055942	3.516822846	*SLC25A16*
Biological Process	Columnar/Cuboidal Epithelial Cell Differentiation (GO:0002065)	0.3069985	0.6461364	0	0	2.978055942	3.516822846	*SOX4*
Biological Process	Copper Ion Transport (GO:0006825)	0.3069985	0.6461364	0	0	2.978055942	3.516822846	*SLC11A2*
Biological Process	Response To Forskolin (GO:1904321)	0.3069985	0.6461364	0	0	2.978055942	3.516822846	*CREB1*
Biological Process	Response To Leucine (GO:0043201)	0.3069985	0.6461364	0	0	2.978055942	3.516822846	*RRAGD*
Biological Process	Ribonucleoside Bisphosphate Biosynthetic Process (GO:0034030)	0.3069985	0.6461364	0	0	2.978055942	3.516822846	*SLC25A16*
Biological Process	snRNA Transcription By RNA Polymerase II (GO:0042795)	0.3069985	0.6461364	0	0	2.978055942	3.516822846	*ELL*
Biological Process	Epithelial Cell Apoptotic Process (GO:1904019)	0.3069985	0.6461364	0	0	2.978055942	3.516822846	*HIPK1*
Biological Process	Establishment Of Endothelial Intestinal Barrier (GO:0090557)	0.3069985	0.6461364	0	0	2.978055942	3.516822846	*RAPGEF6*
Biological Process	Urogenital System Development (GO:0001655)	0.3069985	0.6461364	0	0	2.978055942	3.516822846	*OSR2*
Biological Process	Histone H3-K14 Acetylation (GO:0044154)	0.3069985	0.6461364	0	0	2.978055942	3.516822846	*WDR5*
Biological Process	Kinetochore Organization (GO:0051383)	0.3069985	0.6461364	0	0	2.978055942	3.516822846	*CENPH*
Biological Process	Mesonephros Development (GO:0001823)	0.3069985	0.6461364	0	0	2.978055942	3.516822846	*OSR2*
Biological Process	Metanephric Mesenchyme Development (GO:0072075)	0.3069985	0.6461364	0	0	2.978055942	3.516822846	*SIX4*
Biological Process	Midbrain Dopaminergic Neuron Differentiation (GO:1904948)	0.3069985	0.6461364	0	0	2.978055942	3.516822846	*LRP6*
Biological Process	Negative Regulation Of Activin Receptor Signaling Pathway (GO:0032926)	0.3069985	0.6461364	0	0	2.978055942	3.516822846	*NOMO3*
Biological Process	Negative Regulation Of Adenylate Cyclase Activity (GO:0007194)	0.3069985	0.6461364	0	0	2.978055942	3.516822846	*GNAI3*
Biological Process	Negative Regulation Of Homotypic Cell-Cell Adhesion (GO:0034111)	0.3069985	0.6461364	0	0	2.978055942	3.516822846	*ZNF703*
Biological Process	Negative Regulation Of Inclusion Body Assembly (GO:0090084)	0.3069985	0.6461364	0	0	2.978055942	3.516822846	*DNAJB6*
Biological Process	Negative Regulation Of Interferon-Beta Production (GO:0032688)	0.3069985	0.6461364	0	0	2.978055942	3.516822846	*YY1*
Biological Process	Negative Regulation Of Lymphocyte Migration (GO:2000402)	0.3069985	0.6461364	0	0	2.978055942	3.516822846	*MIA3*
Biological Process	Negative Regulation Of Mitochondrial Outer Membrane Permeabilization Involved In Apoptotic Signaling Pathway (GO:1901029)	0.3069985	0.6461364	0	0	2.978055942	3.516822846	*SLC35F6*

Biological Process	Negative Regulation Of Natural Killer Cell Mediated Immunity (GO:0002716)	0.3069985	0.6461364	0	0	2.978055942	3.516822846	*MICA*
Biological Process	Negative Regulation Of Sprouting Angiogenesis (GO:1903671)	0.3069985	0.6461364	0	0	2.978055942	3.516822846	*PIK3CB*
Biological Process	Neuron Projection Fasciculation (GO:0106030)	0.3069985	0.6461364	0	0	2.978055942	3.516822846	*CNTN4*
Biological Process	Nuclear RNA Surveillance (GO:0071027)	0.3069985	0.6461364	0	0	2.978055942	3.516822846	*XRN1*
Biological Process	Nuclear Pore Complex Assembly (GO:0051292)	0.3069985	0.6461364	0	0	2.978055942	3.516822846	*NUP153*
Biological Process	Positive Regulation Of DNA Methylation-Dependent Heterochromatin Formation (GO:0090309)	0.3069985	0.6461364	0	0	2.978055942	3.516822846	*SETDB1*
Biological Process	Positive Regulation Of Actin Nucleation (GO:0051127)	0.3069985	0.6461364	0	0	2.978055942	3.516822846	*WASF3*
Biological Process	Positive Regulation Of Attachment Of Spindle Microtubules To Kinetochore (GO:0051987)	0.3069985	0.6461364	0	0	2.978055942	3.516822846	*HNRNPU*
Biological Process	Positive Regulation Of Autophagy Of Mitochondrion (GO:1903599)	0.3069985	0.6461364	0	0	2.978055942	3.516822846	*CAMKK2*
Biological Process	Positive Regulation Of Cellular Respiration (GO:1901857)	0.3069985	0.6461364	0	0	2.978055942	3.516822846	*VCP*
Biological Process	Positive Regulation Of Cholesterol Metabolic Process (GO:0090205)	0.3069985	0.6461364	0	0	2.978055942	3.516822846	*PRKAA1*
Biological Process	Positive Regulation Of Epidermal Growth Factor-Activated Receptor Activity (GO:0045741)	0.3069985	0.6461364	0	0	2.978055942	3.516822846	*EPGN*
Biological Process	Positive Regulation Of Integrin Activation (GO:0033625)	0.3069985	0.6461364	0	0	2.978055942	3.516822846	*CXCL13*
Biological Process	Positive Regulation Of Mitochondrial Outer Membrane Permeabilization Involved In Apoptotic Signaling Pathway (GO:1901030)	0.3069985	0.6461364	0	0	2.978055942	3.516822846	*GSK3B*
Biological Process	Positive Regulation Of Non-Canonical Wnt Signaling Pathway (GO:2000052)	0.3069985	0.6461364	0	0	2.978055942	3.516822846	*MLLT3*
Biological Process	Positive Regulation Of Voltage-Gated Potassium Channel Activity (GO:1903818)	0.3069985	0.6461364	0	0	2.978055942	3.516822846	*ALG10B*
Biological Process	Protein auto-ADP-ribosylation (GO:0070213)	0.3069985	0.6461364	0	0	2.978055942	3.516822846	*PARP11*
Biological Process	Purine Nucleoside Bisphosphate Biosynthetic Process (GO:0034033)	0.3069985	0.6461364	0	0	2.978055942	3.516822846	*SLC25A16*
Biological Process	rRNA Base Methylation (GO:0070475)	0.3069985	0.6461364	0	0	2.978055942	3.516822846	*METTL15*
Biological Process	Receptor-Mediated Endocytosis Of Virus By Host Cell (GO:0019065)	0.3069985	0.6461364	0	0	2.978055942	3.516822846	*TPCN2*
Biological Process	Regulation Of B Cell Apoptotic Process (GO:0002902)	0.3069985	0.6461364	0	0	2.978055942	3.516822846	*FNIP1*
Biological Process	Regulation Of T-helper 2 Cell Cytokine Production (GO:2000551)	0.3069985	0.6461364	0	0	2.978055942	3.516822846	*DENND1B*
Biological Process	Regulation Of Dendrite Morphogenesis (GO:0048814)	0.3075948	0.6461364	0	0	1.849101911	2.180039222	*GSK3B;GORASP1*
Biological Process	Regulation Of Gluconeogenesis (GO:0006111)	0.3075948	0.6461364	0	0	1.849101911	2.180039222	*EP300;PPARGC1A*
Biological Process	Regulation Of Protein Localization To Cell Surface (GO:2000008)	0.3075948	0.6461364	0	0	1.849101911	2.180039222	*CTNNB1;STX3*

Biological Process	Negative Regulation Of Intracellular Steroid Hormone Receptor Signaling Pathway (GO:0033144)	0.3075948	0.6461364	0	0	1.849101911	2.180039222	*CLOCK;PIAS2*
Biological Process	Negative Regulation Of Microtubule Polymerization Or Depolymerization (GO:0031111)	0.3075948	0.6461364	0	0	1.849101911	2.180039222	*DYRK1A;CLASP2*
Biological Process	Non-Motile Cilium Assembly (GO:1905515)	0.3075948	0.6461364	0	0	1.849101911	2.180039222	*TBC1D32;RPGRIP1L*
Biological Process	Positive Regulation Of Glucose Transmembrane Transport (GO:0010828)	0.3075948	0.6461364	0	0	1.849101911	2.180039222	*SORBS1;APPL1*
Biological Process	Positive Regulation Of Proteolysis Involved In Protein Catabolic Process (GO:1903052)	0.3075948	0.6461364	0	0	1.849101911	2.180039222	*SMURF1;TMTC3*
Biological Process	Regulation Of Cell Morphogenesis (GO:0022604)	0.3075948	0.6461364	0	0	1.849101911	2.180039222	*ZMYM3;PHIP*
Biological Process	Regulation Of Response To External Stimulus (GO:0032101)	0.3077717	0.6461364	0	0	1.330578512	1.567949915	*CELF1;ELMOD2;ERAP1;SPATA2;XIAP;APPL1*
Biological Process	Apical Junction Assembly (GO:0043297)	0.3078234	0.6461364	0	0	1.57756994	1.85873883	*PDCD6IP;CLDN18;PKN2*
Biological Process	Regulation Of Myoblast Differentiation (GO:0045661)	0.3078234	0.6461364	0	0	1.57756994	1.85873883	*PBRM1;ARID1A;SOX4*
Biological Process	Regulation Of Smooth Muscle Cell Proliferation (GO:0048660)	0.3078234	0.6461364	0	0	1.57756994	1.85873883	*PRKDC;CTNNB1;PPARGC1A*
Biological Process	Endoplasmic Reticulum Organization (GO:0007029)	0.3078234	0.6461364	0	0	1.57756994	1.85873883	*ATL2;TOR1AIP2;MIA3*
Biological Process	Mesenchymal Cell Differentiation (GO:0048762)	0.3078234	0.6461364	0	0	1.57756994	1.85873883	*GSK3B;CTNNB1;LRP6*
Biological Process	Negative Regulation Of Protein Ubiquitination (GO:0031397)	0.3078234	0.6461364	0	0	1.57756994	1.85873883	*U2AF2;SPRY2;SOX4*
Biological Process	Nucleosome Assembly (GO:0006334)	0.3078234	0.6461364	0	0	1.57756994	1.85873883	*SUPT16H;RBBP4;ATRX*
Biological Process	Peptidyl-Tyrosine Modification (GO:0018212)	0.3078234	0.6461364	0	0	1.57756994	1.85873883	*FER;RELN;DYRK1A*
Biological Process	Protein K63-linked Ubiquitination (GO:0070534)	0.3078234	0.6461364	0	0	1.57756994	1.85873883	*UBE2D4;TRAF6;RNF8*
Biological Process	Cellular Response To UV (GO:0034644)	0.3171081	0.662676	0	0	1.430540037	1.642993035	*YY1;ERCC4;DHX36;EP300*
Biological Process	Vesicle Fusion (GO:0006906)	0.3171081	0.662676	0	0	1.430540037	1.642993035	*SYT4;YIPF5;VAMP4;BET1*
Biological Process	NIK/NF-kappaB Signaling (GO:0038061)	0.3209807	0.662676	0	0	1.787372331	2.031124152	*TRAF6;BTRC*
Biological Process	Regulation Of Natural Killer Cell Mediated Cytotoxicity (GO:0042269)	0.3209807	0.662676	0	0	1.787372331	2.031124152	*RASGRP1;MICA*
Biological Process	Regulation Of Transcription By RNA Polymerase I (GO:0006356)	0.3209807	0.662676	0	0	1.787372331	2.031124152	*BAZ1B;PHF8*
Biological Process	Regulation Of Ubiquitin-Protein Transferase Activity (GO:0051438)	0.3209807	0.662676	0	0	1.787372331	2.031124152	*DCUN1D5;FEM1B*
Biological Process	Chromosome Condensation (GO:0030261)	0.3209807	0.662676	0	0	1.787372331	2.031124152	*DFFB;TTN*
Biological Process	Transition Metal Ion Transport (GO:0000041)	0.3209807	0.662676	0	0	1.787372331	2.031124152	*SLC30A5;SLC11A2*
Biological Process	Glycosphingolipid Metabolic Process (GO:0006687)	0.3209807	0.662676	0	0	1.787372331	2.031124152	*FUT9;ST3GAL5*
Biological Process	Negative Regulation Of Fat Cell Differentiation (GO:0045599)	0.3209807	0.662676	0	0	1.787372331	2.031124152	*ANKRD26;RUNX1T1*
Biological Process	Negative Regulation Of Proteasomal Ubiquitin-Dependent Protein Catabolic Process (GO:0032435)	0.3209807	0.662676	0	0	1.787372331	2.031124152	*USP9X;WAC*
Biological Process	Neural Tube Closure (GO:0001843)	0.3209807	0.662676	0	0	1.787372331	2.031124152	*KDM2B;BCL10*
Biological Process	Phosphatidylinositol 3-Kinase Signaling (GO:0014065)	0.3209807	0.662676	0	0	1.787372331	2.031124152	*STAT3;PIK3CB*
Biological Process	Positive Regulation Of Cell Differentiation (GO:0045597)	0.3223278	0.662676	0	0	1.188879855	1.346033477	*GSK3B;PBRM1;ZHX3;CREB1;DHX36;ZNF703;VWC2;SUCO;CTNNB1;ARID1A;ZNF268;SOX4*

Biological Process	Regulation Of Organelle Assembly (GO:1902115)	0.3253103	0.662676	0	0	1.411643634	1.585241692	*TBC1D1;PDCD6IP;CEP76;EVI5*
Biological Process	Regulation Of Cell Cycle Process (GO:0010564)	0.3268317	0.662676	0	0	1.29818379	1.451772001	*PBRM1;BCL7A;CTNNB1;PUM1;ARID1A;PUM2*
Biological Process	Regulation Of Neuron Apoptotic Process (GO:0043523)	0.3273233	0.662676	0	0	1.341258741	1.497926932	*KDM2B;SIX4;RASA1;CTNNB1;PPARGC1A*
Biological Process	Negative Regulation Of MAPK Cascade (GO:0043409)	0.3273233	0.662676	0	0	1.341258741	1.497926932	*DUSP5;ADIPOQ;SPRY2;SPRY1;PIK3CB*
Biological Process	Pattern Recognition Receptor Signaling Pathway (GO:0002221)	0.3276955	0.662676	0	0	1.517881108	1.693455142	*TRAF6;TIFA;BCL10*
Biological Process	Regulation Of Cytoplasmic Transport (GO:1903649)	0.331959	0.662676	0	0	2.680111266	2.955475831	*MAPK1*
Biological Process	Golgi To Lysosome Transport (GO:0090160)	0.331959	0.662676	0	0	2.680111266	2.955475831	*AP1G1*
Biological Process	RNA Splicing, Via Endonucleolytic Cleavage And Ligation (GO:0000394)	0.331959	0.662676	0	0	2.680111266	2.955475831	*TSEN2*
Biological Process	Regulation Of Homotypic Cell-Cell Adhesion (GO:0034110)	0.331959	0.662676	0	0	2.680111266	2.955475831	*ZNF703*
Biological Process	Regulation Of Keratinocyte Migration (GO:0051547)	0.331959	0.662676	0	0	2.680111266	2.955475831	*EPB41L4B*
Biological Process	Regulation Of Leukocyte Tethering Or Rolling (GO:1903236)	0.331959	0.662676	0	0	2.680111266	2.955475831	*FUT9*
Biological Process	Regulation Of mRNA 3'-End Processing (GO:0031440)	0.331959	0.662676	0	0	2.680111266	2.955475831	*DHX36*
Biological Process	Regulation Of Mesenchymal Cell Proliferation (GO:0010464)	0.331959	0.662676	0	0	2.680111266	2.955475831	*PHF14*
Biological Process	Regulation Of Microtubule-Based Movement (GO:0060632)	0.331959	0.662676	0	0	2.680111266	2.955475831	*TTC21B*
Biological Process	Brain Morphogenesis (GO:0048854)	0.331959	0.662676	0	0	2.680111266	2.955475831	*FBXW11*
Biological Process	Regulation Of Phospholipase C Activity (GO:1900274)	0.331959	0.662676	0	0	2.680111266	2.955475831	*RASGRP1*
Biological Process	Calcineurin-Mediated Signaling (GO:0097720)	0.331959	0.662676	0	0	2.680111266	2.955475831	*NFAT5*
Biological Process	Regulation Of Stress-Activated Protein Kinase Signaling Cascade (GO:0070302)	0.331959	0.662676	0	0	2.680111266	2.955475831	*MAPK1*
Biological Process	Cellular Response To Growth Hormone Stimulus (GO:0071378)	0.331959	0.662676	0	0	2.680111266	2.955475831	*STAT3*
Biological Process	Central Nervous System Neuron Axonogenesis (GO:0021955)	0.331959	0.662676	0	0	2.680111266	2.955475831	*EPHB1*
Biological Process	Regulation Of Ventricular Cardiac Muscle Cell Action Potential (GO:0098911)	0.331959	0.662676	0	0	2.680111266	2.955475831	*CACNA1C*
Biological Process	Respiratory Chain Complex III Assembly (GO:0017062)	0.331959	0.662676	0	0	2.680111266	2.955475831	*UQCC2*
Biological Process	Dosage Compensation By Inactivation Of X Chromosome (GO:0009048)	0.331959	0.662676	0	0	2.680111266	2.955475831	*HNRNPU*
Biological Process	Endoplasmic Reticulum Membrane Organization (GO:0090158)	0.331959	0.662676	0	0	2.680111266	2.955475831	*ATL2*
Biological Process	Smooth Muscle Cell Differentiation (GO:0051145)	0.331959	0.662676	0	0	2.680111266	2.955475831	*HEY1*
Biological Process	Exit From Mitosis (GO:0010458)	0.331959	0.662676	0	0	2.680111266	2.955475831	*CLASP2*
Biological Process	tRNA Splicing, Via Endonucleolytic Cleavage And Ligation (GO:0006388)	0.331959	0.662676	0	0	2.680111266	2.955475831	*TSEN2*
Biological Process	Glutamine Family Amino Acid Biosynthetic Process (GO:0009084)	0.331959	0.662676	0	0	2.680111266	2.955475831	*GLS*
Biological Process	Histone H2A Ubiquitination (GO:0033522)	0.331959	0.662676	0	0	2.680111266	2.955475831	*RNF8*

Biological Process	Isotype Switching (GO:0045190)	0.331959	0.662676	0	0	2.680111266	2.955475831	*RNF8*
Biological Process	Melanocyte Differentiation (GO:0030318)	0.331959	0.662676	0	0	2.680111266	2.955475831	*USP13*
Biological Process	Mitochondrial Respiratory Chain Complex III Assembly (GO:0034551)	0.331959	0.662676	0	0	2.680111266	2.955475831	*UQCC2*
Biological Process	Mitochondrion Distribution (GO:0048311)	0.331959	0.662676	0	0	2.680111266	2.955475831	*MEF2A*
Biological Process	Negative Regulation Of RNA Metabolic Process (GO:0051253)	0.331959	0.662676	0	0	2.680111266	2.955475831	*PTBP3*
Biological Process	Negative Regulation Of calcineurin-NFAT Signaling Cascade (GO:0070885)	0.331959	0.662676	0	0	2.680111266	2.955475831	*GSK3B*
Biological Process	Negative Regulation Of Calcineurin-Mediated Signaling (GO:0106057)	0.331959	0.662676	0	0	2.680111266	2.955475831	*GSK3B*
Biological Process	Negative Regulation Of Cyclase Activity (GO:0031280)	0.331959	0.662676	0	0	2.680111266	2.955475831	*GNAI3*
Biological Process	Negative Regulation Of Microtubule Polymerization (GO:0031115)	0.331959	0.662676	0	0	2.680111266	2.955475831	*DYRK1A*
Biological Process	Negative Regulation Of Nuclear-Transcribed mRNA Catabolic Process, Deadenylation-Dependent Decay (GO:1900152)	0.331959	0.662676	0	0	2.680111266	2.955475831	*HNRNPU*
Biological Process	Negative Regulation Of Platelet-Derived Growth Factor Receptor Signaling Pathway (GO:0010642)	0.331959	0.662676	0	0	2.680111266	2.955475831	*PHF14*
Biological Process	Phosphatidylinositol-3-Phosphate Biosynthetic Process (GO:0036092)	0.331959	0.662676	0	0	2.680111266	2.955475831	*PIK3CB*
Biological Process	Positive Regulation Of Branching Involved In Ureteric Bud Morphogenesis (GO:0090190)	0.331959	0.662676	0	0	2.680111266	2.955475831	*SIX4*
Biological Process	Positive Regulation Of Lipid Transport (GO:0032370)	0.331959	0.662676	0	0	2.680111266	2.955475831	*ADIPOQ*
Biological Process	Positive Regulation Of Metaphase/Anaphase Transition Of Cell Cycle (GO:1902101)	0.331959	0.662676	0	0	2.680111266	2.955475831	*CUL3*
Biological Process	Positive Regulation Of Mitotic Metaphase/Anaphase Transition (GO:0045842)	0.331959	0.662676	0	0	2.680111266	2.955475831	*CUL3*
Biological Process	Positive Regulation Of Protein Maturation (GO:1903319)	0.331959	0.662676	0	0	2.680111266	2.955475831	*SOX4*
Biological Process	pre-mRNA Cleavage Required For Polyadenylation (GO:0098789)	0.331959	0.662676	0	0	2.680111266	2.955475831	*CPSF6*
Biological Process	Regulation Of Adipose Tissue Development (GO:1904177)	0.331959	0.662676	0	0	2.680111266	2.955475831	*PRKAA1*
Biological Process	Regulation Of Cell Cycle Checkpoint (GO:1901976)	0.331959	0.662676	0	0	2.680111266	2.955475831	*FEM1B*
Biological Process	Activation Of Protein Kinase Activity (GO:0032147)	0.3335257	0.662676	0	0	1.393238047	1.529824668	*PRRC1;PDPK1;TRAF6;CAMKK2*
Biological Process	Regulation Of Sprouting Angiogenesis (GO:1903670)	0.3343015	0.662676	0	0	1.729625303	1.895171351	*PDPK1;PIK3CB*
Biological Process	Signal Transduction By P53 Class Mediator (GO:0072331)	0.3343015	0.662676	0	0	1.729625303	1.895171351	*ATRX;JMY*
Biological Process	Inositol Lipid-Mediated Signaling (GO:0048017)	0.3343015	0.662676	0	0	1.729625303	1.895171351	*PLCB4;PLCH1*
Biological Process	Monoubiquitinated Protein Deubiquitination (GO:0035520)	0.3343015	0.662676	0	0	1.729625303	1.895171351	*USP15;USP9X*
Biological Process	ncRNA Catabolic Process (GO:0034661)	0.3343015	0.662676	0	0	1.729625303	1.895171351	*XRN1;DIS3*

Biological Process	Negative Regulation Of Small Molecule Metabolic Process (GO:0062014)	0.3343015	0.662676	0	0	1.729625303	1.895171351	*ADIPOQ;EP300*
Biological Process	Phosphatidic Acid Metabolic Process (GO:0046473)	0.3343015	0.662676	0	0	1.729625303	1.895171351	*GPAT3;LCLAT1*
Biological Process	Positive Regulation Of Epidermal Growth Factor Receptor Signaling Pathway (GO:0045742)	0.3343015	0.662676	0	0	1.729625303	1.895171351	*EPGN;CBL*
Biological Process	Regulation Of Amyloid-Beta Formation (GO:1902003)	0.3343015	0.662676	0	0	1.729625303	1.895171351	*RTN1;CASP3*
Biological Process	Response To Calcium Ion (GO:0051592)	0.334465	0.662676	0	0	1.327909714	1.45435737	*MEF2A;SYT4;PRKAA1;PRKAA2;TTN*
Biological Process	Regulation Of G1/S Transition Of Mitotic Cell Cycle (GO:2000045)	0.334465	0.662676	0	0	1.327909714	1.45435737	*PBRM1;DDX3X;BCL7A;ARID1A;APPL1*
Biological Process	Myeloid Cell Differentiation (GO:0030099)	0.3376313	0.6684493	0	0	1.489694716	1.617511936	*NCOA6;CASP3;KAT6A*
Biological Process	Circulatory System Development (GO:0072359)	0.3396253	0.6712466	0	0	1.277444311	1.379527702	*MEF2A;HEY1;NCOA6;PHIP;CACNA1C;SOX4*
Biological Process	Positive Regulation Of T Cell Activation (GO:0050870)	0.34162	0.6712466	0	0	1.314822432	1.412193389	*PBRM1;CD209;CD47;ARID1A;NCK1*
Biological Process	Cellular Response To Organic Substance (GO:0071310)	0.3417504	0.6712466	0	0	1.375304398	1.476629392	*NFAT5;AVPR1B;TRAF6;STAT3*
Biological Process	Positive Regulation Of Tumor Necrosis Factor Superfamily Cytokine Production (GO:1903557)	0.3417504	0.6712466	0	0	1.375304398	1.476629392	*ARFGEF2;STAT3;BCL10;RASGRP1*
Biological Process	Establishment Of Mitotic Spindle Localization (GO:0040001)	0.3475446	0.6712466	0	0	1.675487465	1.770759611	*FBXW11;CLASP2*
Biological Process	Negative Regulation Of Mitotic Cell Cycle (GO:0045930)	0.3475446	0.6712466	0	0	1.675487465	1.770759611	*FZD3;CTNNB1*
Biological Process	Positive Regulation Of Transcription By RNA Polymerase I (GO:0045943)	0.3475446	0.6712466	0	0	1.675487465	1.770759611	*BAZ1B;PHF8*
Biological Process	Regulation Of calcineurin-NFAT Signaling Cascade (GO:0070884)	0.3475446	0.6712466	0	0	1.675487465	1.770759611	*GSK3B;SPPL3*
Biological Process	Response To Organic Cyclic Compound (GO:0014070)	0.3475581	0.6712466	0	0	1.462533283	1.545639413	*CREB1;STAT3;CTNNB1*
Biological Process	Eye Development (GO:0001654)	0.3475581	0.6712466	0	0	1.462533283	1.545639413	*PBX2P1;CACNA1C;HIPK1*
Biological Process	Memory (GO:0007613)	0.3475581	0.6712466	0	0	1.462533283	1.545639413	*YTHDF1;ATXN1;CPEB3*
Biological Process	Positive Regulation Of Mitochondrion Organization (GO:0010822)	0.3475581	0.6712466	0	0	1.462533283	1.545639413	*GSK3B;PPARGC1A;CAMKK2*
Biological Process	Positive Regulation Of Cysteine-Type Endopeptidase Activity Involved In Apoptotic Process (GO:0043280)	0.3559595	0.6712466	0	0	1.289402905	1.331873684	*VCP;DDX3X;APAF1;BCL10;SENP1*
Biological Process	MyD88-dependent Toll-Like Receptor Signaling Pathway (GO:0002755)	0.3560217	0.6712466	0	0	2.436338349	2.516161515	*TRAF6*
Biological Process	V(D)J Recombination (GO:0033151)	0.3560217	0.6712466	0	0	2.436338349	2.516161515	*PRKDC*
Biological Process	acetyl-CoA Biosynthetic Process (GO:0006085)	0.3560217	0.6712466	0	0	2.436338349	2.516161515	*PDHA1*
Biological Process	Regulation Of Lymphocyte Migration (GO:2000401)	0.3560217	0.6712466	0	0	2.436338349	2.516161515	*MIA3*
Biological Process	Adult Locomotory Behavior (GO:0008344)	0.3560217	0.6712466	0	0	2.436338349	2.516161515	*PUM1*
Biological Process	Regulation Of Monooxygenase Activity (GO:0032768)	0.3560217	0.6712466	0	0	2.436338349	2.516161515	*ZDHHC21*
Biological Process	Axonal Fasciculation (GO:0007413)	0.3560217	0.6712466	0	0	2.436338349	2.516161515	*CNTN4*
Biological Process	Regulation Of Platelet-Derived Growth Factor Receptor Signaling Pathway (GO:0010640)	0.3560217	0.6712466	0	0	2.436338349	2.516161515	*CBL*
Biological Process	Cardiac Right Ventricle Morphogenesis (GO:0003215)	0.3560217	0.6712466	0	0	2.436338349	2.516161515	*SOX4*

Biological Process	Cardiolipin Metabolic Process (GO:0032048)	0.3560217	0.6712466	0	0	2.436338349	2.516161515	*LCLAT1*
Biological Process	Cellular Response To Leucine Starvation (GO:1990253)	0.3560217	0.6712466	0	0	2.436338349	2.516161515	*RRAGD*
Biological Process	Regulation Of Vascular Associated Smooth Muscle Cell Migration (GO:1904752)	0.3560217	0.6712466	0	0	2.436338349	2.516161515	*ADIPOQ*
Biological Process	Response To Iron Ion (GO:0010039)	0.3560217	0.6712466	0	0	2.436338349	2.516161515	*SLC11A2*
Biological Process	Dendrite Self-Avoidance (GO:0070593)	0.3560217	0.6712466	0	0	2.436338349	2.516161515	*CNTN4*
Biological Process	Early Endosome To Golgi Transport (GO:0034498)	0.3560217	0.6712466	0	0	2.436338349	2.516161515	*SURF4*
Biological Process	Semaphorin-Plexin Signaling Pathway Involved In Neuron Projection Guidance (GO:1902285)	0.3560217	0.6712466	0	0	2.436338349	2.516161515	*PLXNC1*
Biological Process	snRNA Transcription (GO:0009301)	0.3560217	0.6712466	0	0	2.436338349	2.516161515	*ELL*
Biological Process	Sodium Ion Export Across Plasma Membrane (GO:0036376)	0.3560217	0.6712466	0	0	2.436338349	2.516161515	*SLC4A4*
Biological Process	Eye Photoreceptor Cell Differentiation (GO:0001754)	0.3560217	0.6712466	0	0	2.436338349	2.516161515	*STAT3*
Biological Process	Fatty Acid Elongation (GO:0030497)	0.3560217	0.6712466	0	0	2.436338349	2.516161515	*ELOVL4*
Biological Process	Folic Acid Metabolic Process (GO:0046655)	0.3560217	0.6712466	0	0	2.436338349	2.516161515	*SLC25A32*
Biological Process	Gamma-Delta T Cell Activation (GO:0046629)	0.3560217	0.6712466	0	0	2.436338349	2.516161515	*MICA*
Biological Process	Glucose Import (GO:0046323)	0.3560217	0.6712466	0	0	2.436338349	2.516161515	*ZDHHC7*
Biological Process	Very Long-Chain Fatty Acid Biosynthetic Process (GO:0042761)	0.3560217	0.6712466	0	0	2.436338349	2.516161515	*ELOVL4*
Biological Process	Histone Monoubiquitination (GO:0010390)	0.3560217	0.6712466	0	0	2.436338349	2.516161515	*WAC*
Biological Process	Lipid Droplet Formation (GO:0140042)	0.3560217	0.6712466	0	0	2.436338349	2.516161515	*CDS2*
Biological Process	mRNA Cleavage Involved In mRNA Processing (GO:0098787)	0.3560217	0.6712466	0	0	2.436338349	2.516161515	*CPSF6*
Biological Process	Mitotic DNA Integrity Checkpoint Signaling (GO:0044774)	0.3560217	0.6712466	0	0	2.436338349	2.516161515	*ORC1*
Biological Process	Morphogenesis Of A Polarized Epithelium (GO:0001738)	0.3560217	0.6712466	0	0	2.436338349	2.516161515	*ZDHHC7*
Biological Process	Multicellular Organismal-Level Iron Ion Homeostasis (GO:0060586)	0.3560217	0.6712466	0	0	2.436338349	2.516161515	*SLC11A2*
Biological Process	Negative Regulation By Host Of Viral Process (GO:0044793)	0.3560217	0.6712466	0	0	2.436338349	2.516161515	*SMC6*
Biological Process	Negative Regulation Of Intrinsic Apoptotic Signaling Pathway In Response To DNA Damage By P53 Class Mediator (GO:1902166)	0.3560217	0.6712466	0	0	2.436338349	2.516161515	*ING2*
Biological Process	Negative Regulation Of Macrophage Derived Foam Cell Differentiation (GO:0010745)	0.3560217	0.6712466	0	0	2.436338349	2.516161515	*ADIPOQ*
Biological Process	Negative Regulation Of Mitochondrial Membrane Permeability (GO:0035795)	0.3560217	0.6712466	0	0	2.436338349	2.516161515	*SLC35F6*
Biological Process	Negative Regulation Of Mononuclear Cell Migration (GO:0071676)	0.3560217	0.6712466	0	0	2.436338349	2.516161515	*MIA3*
Biological Process	Polysaccharide Biosynthetic Process (GO:0000271)	0.3560217	0.6712466	0	0	2.436338349	2.516161515	*FUT9*
Biological Process	Positive Regulation By Symbiont Of Entry Into Host (GO:0075294)	0.3560217	0.6712466	0	0	2.436338349	2.516161515	*TMPRSS4*

Biological Process	Positive Regulation Of ERAD Pathway (GO:1904294)	0.3560217	0.6712466	0	0	2.436338349	2.516161515	*USP13*
Biological Process	Positive Regulation Of T Cell Chemotaxis (GO:0010820)	0.3560217	0.6712466	0	0	2.436338349	2.516161515	*CXCL13*
Biological Process	Positive Regulation Of Cell Morphogenesis Involved In Differentiation (GO:0010770)	0.3560217	0.6712466	0	0	2.436338349	2.516161515	*PRKDC*
Biological Process	Positive Regulation Of Cellular Extravasation (GO:0002693)	0.3560217	0.6712466	0	0	2.436338349	2.516161515	*CD47*
Biological Process	Positive Regulation Of Fatty Acid Oxidation (GO:0046321)	0.3560217	0.6712466	0	0	2.436338349	2.516161515	*PPARGC1A*
Biological Process	Positive Regulation Of Insulin Receptor Signaling Pathway (GO:0046628)	0.3560217	0.6712466	0	0	2.436338349	2.516161515	*SORBS1*
Biological Process	Positive Regulation Of Protein Export From Nucleus (GO:0046827)	0.3560217	0.6712466	0	0	2.436338349	2.516161515	*GSK3B*
Biological Process	Positive Regulation Of Protein Localization To Cell Surface (GO:2000010)	0.3560217	0.6712466	0	0	2.436338349	2.516161515	*STX3*
Biological Process	Positive Regulation Of Regulated Secretory Pathway (GO:1903307)	0.3560217	0.6712466	0	0	2.436338349	2.516161515	*SYT4*
Biological Process	Positive Regulation Of Vascular Endothelial Cell Proliferation (GO:1905564)	0.3560217	0.6712466	0	0	2.436338349	2.516161515	*PDPK1*
Biological Process	Positive Regulation Of Vasoconstriction (GO:0045907)	0.3560217	0.6712466	0	0	2.436338349	2.516161515	*AVPR1B*
Biological Process	Positive Regulation Of Viral Entry Into Host Cell (GO:0046598)	0.3560217	0.6712466	0	0	2.436338349	2.516161515	*TMPRSS4*
Biological Process	pre-miRNA Processing (GO:0031054)	0.3560217	0.6712466	0	0	2.436338349	2.516161515	*AGO3*
Biological Process	Protein Heterotetramerization (GO:0051290)	0.3560217	0.6712466	0	0	2.436338349	2.516161515	*CPSF6*
Biological Process	Protein Insertion Into Mitochondrial Inner Membrane (GO:0045039)	0.3560217	0.6712466	0	0	2.436338349	2.516161515	*TIMM29*
Biological Process	Pseudouridine Synthesis (GO:0001522)	0.3560217	0.6712466	0	0	2.436338349	2.516161515	*PUS10*
Biological Process	Quaternary Ammonium Group Transport (GO:0015697)	0.3560217	0.6712466	0	0	2.436338349	2.516161515	*SLC22A5*
Biological Process	DNA Repair (GO:0006281)	0.3567912	0.6720636	0	0	1.154304113	1.189630967	*ATXN3;VCP;PRKDC;ERCC4;EYA3;PSME4;NSD2;ATRX;RNF8;FBXO6;KMT5B;PMS1*
Biological Process	Regulation Of miRNA Transcription (GO:1902893)	0.3574698	0.6720636	0	0	1.436341901	1.477571091	*POU2F1;NFIB;STAT3*
Biological Process	Regulation Of Type I Interferon Production (GO:0032479)	0.3574698	0.6720636	0	0	1.436341901	1.477571091	*DDX3X;CUL3;XIAP*
Biological Process	protein-DNA Complex Assembly (GO:0065004)	0.3574698	0.6720636	0	0	1.436341901	1.477571091	*SUPT16H;RBBP4;ATRX*
Biological Process	Adherens Junction Organization (GO:0034332)	0.3606983	0.6752578	0	0	1.624630708	1.65665762	*ZNF703;CTNNB1*
Biological Process	Regulation Of mRNA Processing (GO:0050684)	0.3606983	0.6752578	0	0	1.624630708	1.65665762	*SON;SRSF10*
Biological Process	Cellular Response To Amino Acid Stimulus (GO:0071230)	0.3606983	0.6752578	0	0	1.624630708	1.65665762	*RRAGD;CPEB3*
Biological Process	Dendrite Morphogenesis (GO:0048813)	0.3606983	0.6752578	0	0	1.624630708	1.65665762	*MEF2A;EPHB1*
Biological Process	Response To Nutrient Levels (GO:0031667)	0.3606983	0.6752578	0	0	1.624630708	1.65665762	*PRKAA1;PRKAA2*
Biological Process	Positive Regulation Of Substrate Adhesion-Dependent Cell Spreading (GO:1900026)	0.3606983	0.6752578	0	0	1.624630708	1.65665762	*ARHGEF7;CRKL*
Biological Process	Regulation Of Extrinsic Apoptotic Signaling Pathway (GO:2001236)	0.3673604	0.6760098	0	0	1.411069515	1.413061612	*FEM1B;PHIP;BCL10*
Biological Process	T Cell Activation (GO:0042110)	0.3703214	0.6760098	0	0	1.264942605	1.2565736	*MFAP3;RASGRP1;SOX4;MICA;NCK1*
Biological Process	Negative Regulation Of Cellular Biosynthetic Process (GO:0031327)	0.371803	0.6760098	0	0	1.228366156	1.215334504	*DDX3X;ADIPOQ;DHX36;EP300;CPEB3;EIF4E2*

Biological Process	DNA Damage Response, Signal Transduction By P53 Class Mediator (GO:0030330)	0.373752	0.6760098	0	0	1.576765525	1.551794071	*TFAP4;ATRX*
Biological Process	Regulation Of Erythrocyte Differentiation (GO:0045646)	0.373752	0.6760098	0	0	1.576765525	1.551794071	*PRKDC;STAT3*
Biological Process	Regulation Of Glucose Import (GO:0046324)	0.373752	0.6760098	0	0	1.576765525	1.551794071	*SORBS1;APPL1*
Biological Process	Regulation Of Peptidyl-Threonine Phosphorylation (GO:0010799)	0.373752	0.6760098	0	0	1.576765525	1.551794071	*MAPK1;SPRY2*
Biological Process	Substrate Adhesion-Dependent Cell Spreading (GO:0034446)	0.373752	0.6760098	0	0	1.576765525	1.551794071	*FER;PEAK1*
Biological Process	Histone H3 Acetylation (GO:0043966)	0.373752	0.6760098	0	0	1.576765525	1.551794071	*DR1;WDR5*
Biological Process	Histone Deacetylation (GO:0016575)	0.373752	0.6760098	0	0	1.576765525	1.551794071	*RBBP4;TBL1XR1*
Biological Process	Negative Regulation Of Sequestering Of Calcium Ion (GO:0051283)	0.373752	0.6760098	0	0	1.576765525	1.551794071	*PLCB4;PLCH1*
Biological Process	Positive Regulation Of Myeloid Cell Differentiation (GO:0045639)	0.373752	0.6760098	0	0	1.576765525	1.551794071	*PRKDC;STAT3*
Biological Process	Positive Regulation Of Signaling Receptor Activity (GO:2000273)	0.373752	0.6760098	0	0	1.576765525	1.551794071	*EPGN;RELN*
Biological Process	Receptor Signaling Pathway Via JAK-STAT (GO:0007259)	0.373752	0.6760098	0	0	1.576765525	1.551794071	*FER;STAT3*
Biological Process	Organelle Assembly (GO:0070925)	0.3761898	0.6760098	0	0	1.128900546	1.103682704	*TTC26;YTHDF1;PDCD6IP;YTHDF3;WDR45B;RABL2B;ATG14;AP3M1;KIF3A;AP1G1;TCTN2;TTC21B;PHIP*
Biological Process	Regulation Of Protein Serine/Threonine Kinase Activity (GO:0071900)	0.3775046	0.6760098	0	0	1.253055356	1.220692065	*CCNT2;DDX3X;CCNG2;SOS1;LRP6*
Biological Process	ATP Transport (GO:0015867)	0.3792188	0.6760098	0	0	2.233194251	2.165398769	*CD47*
Biological Process	GTP Metabolic Process (GO:0046039)	0.3792188	0.6760098	0	0	2.233194251	2.165398769	*GNAI3*
Biological Process	N-terminal Protein Amino Acid Modification (GO:0031365)	0.3792188	0.6760098	0	0	2.233194251	2.165398769	*NAA15*
Biological Process	Regulation Of Humoral Immune Response (GO:0002920)	0.3792188	0.6760098	0	0	2.233194251	2.165398769	*CXCL13*
Biological Process	Regulation Of Intrinsic Apoptotic Signaling Pathway In Response To DNA Damage By P53 Class Mediator (GO:1902165)	0.3792188	0.6760098	0	0	2.233194251	2.165398769	*ING2*
Biological Process	Regulation Of Lamellipodium Organization (GO:1902743)	0.3792188	0.6760098	0	0	2.233194251	2.165398769	*FER*
Biological Process	Regulation Of Leukocyte Migration (GO:0002685)	0.3792188	0.6760098	0	0	2.233194251	2.165398769	*MIA3*
Biological Process	Amyloid Precursor Protein Catabolic Process (GO:0042987)	0.3792188	0.6760098	0	0	2.233194251	2.165398769	*DYRK1A*
Biological Process	Antigen Processing And Presentation Of Endogenous Peptide Antigen Via MHC Class I Via ER Pathway (GO:0002484)	0.3792188	0.6760098	0	0	2.233194251	2.165398769	*MICA*
Biological Process	Antigen Processing And Presentation Of Endogenous Peptide Antigen Via MHC Class I Via ER Pathway, TAP-independent (GO:0002486)	0.3792188	0.6760098	0	0	2.233194251	2.165398769	*MICA*
Biological Process	Antigen Processing And Presentation Of Endogenous Peptide Antigen Via MHC Class Ib (GO:0002476)	0.3792188	0.6760098	0	0	2.233194251	2.165398769	*MICA*
Biological Process	Antigen Processing And Presentation Of Peptide Antigen Via MHC Class Ib (GO:0002428)	0.3792188	0.6760098	0	0	2.233194251	2.165398769	*MICA*

Biological Process	Branching Involved In Blood Vessel Morphogenesis (GO:0001569)	0.3792188	0.6760098	0	0	2.233194251	2.165398769	*CTNNB1*
Biological Process	Regulation Of Response To Stress (GO:0080134)	0.3792188	0.6760098	0	0	2.233194251	2.165398769	*OPRM1*
Biological Process	Regulation Of Trophoblast Cell Migration (GO:1901163)	0.3792188	0.6760098	0	0	2.233194251	2.165398769	*YTHDF3*
Biological Process	Regulation Of Vasculogenesis (GO:2001212)	0.3792188	0.6760098	0	0	2.233194251	2.165398769	*HEY1*
Biological Process	Cotranslational Protein Targeting To Membrane (GO:0006613)	0.3792188	0.6760098	0	0	2.233194251	2.165398769	*SSR1*
Biological Process	Response To Progesterone (GO:0032570)	0.3792188	0.6760098	0	0	2.233194251	2.165398769	*UBE3A*
Biological Process	Signal Peptide Processing (GO:0006465)	0.3792188	0.6760098	0	0	2.233194251	2.165398769	*SPPL3*
Biological Process	Endoplasmic Reticulum Mannose Trimming (GO:1904380)	0.3792188	0.6760098	0	0	2.233194251	2.165398769	*EDEM3*
Biological Process	Suppression Of Viral Release By Host (GO:0044790)	0.3792188	0.6760098	0	0	2.233194251	2.165398769	*MID2*
Biological Process	Eye Photoreceptor Cell Development (GO:0042462)	0.3792188	0.6760098	0	0	2.233194251	2.165398769	*RPGRIP1L*
Biological Process	Glutamine Metabolic Process (GO:0006541)	0.3792188	0.6760098	0	0	2.233194251	2.165398769	*GLS*
Biological Process	Intraciliary Retrograde Transport (GO:0035721)	0.3792188	0.6760098	0	0	2.233194251	2.165398769	*TTC21B*
Biological Process	Mature B Cell Differentiation Involved In Immune Response (GO:0002313)	0.3792188	0.6760098	0	0	2.233194251	2.165398769	*PHF14*
Biological Process	Membrane Depolarization During Action Potential (GO:0086010)	0.3792188	0.6760098	0	0	2.233194251	2.165398769	*CACNA1C*
Biological Process	Microvillus Organization (GO:0032528)	0.3792188	0.6760098	0	0	2.233194251	2.165398769	*RAPGEF6*
Biological Process	Negative Regulation Of DNA Damage Response, Signal Transduction By P53 Class Mediator (GO:0043518)	0.3792188	0.6760098	0	0	2.233194251	2.165398769	*DYRK1A*
Biological Process	Negative Regulation Of Endoplasmic Reticulum Unfolded Protein Response (GO:1900102)	0.3792188	0.6760098	0	0	2.233194251	2.165398769	*NCK1*
Biological Process	Negative Regulation Of Lyase Activity (GO:0051350)	0.3792188	0.6760098	0	0	2.233194251	2.165398769	*GNAI3*
Biological Process	Negative Regulation Of Myoblast Differentiation (GO:0045662)	0.3792188	0.6760098	0	0	2.233194251	2.165398769	*SOX4*
Biological Process	Negative Regulation Of Toll-Like Receptor Signaling Pathway (GO:0034122)	0.3792188	0.6760098	0	0	2.233194251	2.165398769	*LGR4*
Biological Process	Negative Regulation Of Viral Transcription (GO:0032897)	0.3792188	0.6760098	0	0	2.233194251	2.165398769	*MID2*
Biological Process	Nucleotide-Sugar Metabolic Process (GO:0009225)	0.3792188	0.6760098	0	0	2.233194251	2.165398769	*GFPT1*
Biological Process	Peptidyl-Methionine Modification (GO:0018206)	0.3792188	0.6760098	0	0	2.233194251	2.165398769	*NAA15*
Biological Process	Peptidyl-Tyrosine Autophosphorylation (GO:0038083)	0.3792188	0.6760098	0	0	2.233194251	2.165398769	*DYRK1A*
Biological Process	Positive Regulation Of Brown Fat Cell Differentiation (GO:0090336)	0.3792188	0.6760098	0	0	2.233194251	2.165398769	*HNRNPU*
Biological Process	Positive Regulation Of Cellular Response To Insulin Stimulus (GO:1900078)	0.3792188	0.6760098	0	0	2.233194251	2.165398769	*SORBS1*
Biological Process	Positive Regulation Of Cyclase Activity (GO:0031281)	0.3792188	0.6760098	0	0	2.233194251	2.165398769	*CACNA1C*

Biological Process	Positive Regulation Of Glycogen Biosynthetic Process (GO:0045725)	0.3792188	0.6760098	0	0	2.233194251	2.165398769	*SORBS1*
Biological Process	Positive Regulation Of Glycoprotein Biosynthetic Process (GO:0010560)	0.3792188	0.6760098	0	0	2.233194251	2.165398769	*ALG10B*
Biological Process	Positive Regulation Of Granulocyte Macrophage Colony-Stimulating Factor Production (GO:0032725)	0.3792188	0.6760098	0	0	2.233194251	2.165398769	*RASGRP1*
Biological Process	Positive Regulation Of Heterochromatin Formation (GO:0031453)	0.3792188	0.6760098	0	0	2.233194251	2.165398769	*SETDB1*
Biological Process	Positive Regulation Of Histone Deacetylation (GO:0031065)	0.3792188	0.6760098	0	0	2.233194251	2.165398769	*ING2*
Biological Process	Positive Regulation Of Macrophage Chemotaxis (GO:0010759)	0.3792188	0.6760098	0	0	2.233194251	2.165398769	*MAPK1*
Biological Process	Positive Regulation Of Type 2 Immune Response (GO:0002830)	0.3792188	0.6760098	0	0	2.233194251	2.165398769	*DENND1B*
Biological Process	Protein Hydroxylation (GO:0018126)	0.3792188	0.6760098	0	0	2.233194251	2.165398769	*P3H4*
Biological Process	Protein Import Into Peroxisome Matrix (GO:0016558)	0.3792188	0.6760098	0	0	2.233194251	2.165398769	*USP9X*
Biological Process	Protein Localization To Phagophore Assembly Site (GO:0034497)	0.3792188	0.6760098	0	0	2.233194251	2.165398769	*WDR45B*
Biological Process	Regulation Of ERBB Signaling Pathway (GO:1901184)	0.3792188	0.6760098	0	0	2.233194251	2.165398769	*FER*
Biological Process	Regulation Of T Cell Chemotaxis (GO:0010819)	0.3792188	0.6760098	0	0	2.233194251	2.165398769	*CXCL13*
Biological Process	Regulation Of cAMP-dependent Protein Kinase Activity (GO:2000479)	0.3792188	0.6760098	0	0	2.233194251	2.165398769	*PRKAR2A*
Biological Process	Regulation Of Cell Fate Commitment (GO:0010453)	0.3792188	0.6760098	0	0	2.233194251	2.165398769	*RBBP4*
Biological Process	Regulation Of Cell Projection Organization (GO:0031344)	0.3792188	0.6760098	0	0	2.233194251	2.165398769	*SPRY2*
Biological Process	Regulation Of Cellular Ketone Metabolic Process (GO:0010565)	0.3792188	0.6760098	0	0	2.233194251	2.165398769	*SIRT5*
Biological Process	Regulation Of Intracellular Signal Transduction (GO:1902531)	0.3830112	0.6823111	0	0	1.129646149	1.084111355	*ARHGEF12;TMEM127;PDPK1;KAT6A;TRAF6;ITSN1;EP300;ARHGAP29;GPAT3;ARHGAP39;ARHGAP5;RPF2*
Biological Process	Regulation Of p38MAPK Cascade (GO:1900744)	0.3866957	0.6827915	0	0	1.531635495	1.455233177	*RASGRP1;MAP3K4*
Biological Process	Regulation Of Phosphoprotein Phosphatase Activity (GO:0043666)	0.3866957	0.6827915	0	0	1.531635495	1.455233177	*GSK3B;PPP6R2*
Biological Process	Ventricular Septum Development (GO:0003281)	0.3866957	0.6827915	0	0	1.531635495	1.455233177	*HEY1;SOX4*
Biological Process	Melanosome Organization (GO:0032438)	0.3866957	0.6827915	0	0	1.531635495	1.455233177	*AP3M1;AP1G1*
Biological Process	Muscle Tissue Development (GO:0060537)	0.3866957	0.6827915	0	0	1.531635495	1.455233177	*POGLUT1;SIX4*
Biological Process	Positive Regulation Of Exocytosis (GO:0045921)	0.3866957	0.6827915	0	0	1.531635495	1.455233177	*PDCD6IP;CLASP2*
Biological Process	Negative Regulation Of Kinase Activity (GO:0033673)	0.3870556	0.6827915	0	0	1.363094816	1.293831728	*PDPK1;HNRNPU;LRP6*
Biological Process	Protein-Containing Complex Disassembly (GO:0032984)	0.3870556	0.6827915	0	0	1.363094816	1.293831728	*GSK3B;VCP;ATG14*
Biological Process	Positive Regulation Of Cell Migration (GO:0030335)	0.3902452	0.6827915	0	0	1.130560014	1.063834463	*ZNF609;FER;ZNF703;STAT3;PLAA;SPRY2;MIA3;ARHGEF7;ZNF268;CRKL;CLASP2*
Biological Process	Negative Regulation Of Neuron Death (GO:1901215)	0.391084	0.6827915	0	0	1.276669327	1.198579072	*SIX4;KDM2B;RASA1;PPARGC1A*
Biological Process	Positive Regulation Of Cell Adhesion (GO:0045785)	0.391084	0.6827915	0	0	1.276669327	1.198579072	*EPB41L4B;CD47;STX3;TNPO1*

Biological Process	Negative Regulation Of Wnt Signaling Pathway (GO:0030178)	0.3911686	0.6827915	0	0	1.200677286	1.12697568	*TLE4;GSK3B;ZNRF3;TMEM170B;MLLT3;VGLL4*
Biological Process	Negative Regulation Of Canonical Wnt Signaling Pathway (GO:0090090)	0.3918633	0.6827915	0	0	1.229935202	1.152255285	*TLE4;GSK3B;ZNRF3;TMEM170B;MLLT3*
Biological Process	Regulation Of Signal Transduction (GO:0009966)	0.393311	0.6827915	0	0	1.159732766	1.082210007	*KCTD8;GNG4;ADIPOQ;ZDHHC7;PHIP;ZDHHC17;RGS7;NCK1*
Biological Process	Cellular Response To Chemical Stress (GO:0062197)	0.3992707	0.6827915	0	0	1.261583963	1.158279952	*PRKAA1;DDX3X;PRKAA2;PPARGC1A*
Biological Process	Regulation Of Intracellular Transport (GO:0032386)	0.3995205	0.6827915	0	0	1.48901269	1.366154466	*TTC21B;YIPF5*
Biological Process	Amino Acid Catabolic Process (GO:0009063)	0.3995205	0.6827915	0	0	1.48901269	1.366154466	*CARNMT1;DBT*
Biological Process	mRNA Transcription By RNA Polymerase II (GO:0042789)	0.3995205	0.6827915	0	0	1.48901269	1.366154466	*CREB1;TAF8*
Biological Process	Negative Regulation Of Type I Interferon Production (GO:0032480)	0.3995205	0.6827915	0	0	1.48901269	1.366154466	*YY1;CUL3*
Biological Process	Positive Regulation Of Protein Tyrosine Kinase Activity (GO:0061098)	0.3995205	0.6827915	0	0	1.48901269	1.366154466	*EPGN;RELN*
Biological Process	2-Oxoglutarate Metabolic Process (GO:0006103)	0.4015814	0.6827915	0	0	2.061303092	1.880619508	*L2HGDH*
Biological Process	Regulation Of Establishment Of Protein Localization (GO:0070201)	0.4015814	0.6827915	0	0	2.061303092	1.880619508	*CEP120*
Biological Process	Regulation Of Extracellular Matrix Disassembly (GO:0010715)	0.4015814	0.6827915	0	0	2.061303092	1.880619508	*CLASP2*
Biological Process	Regulation Of Feeding Behavior (GO:0060259)	0.4015814	0.6827915	0	0	2.061303092	1.880619508	*STAT3*
Biological Process	RNA Phosphodiester Bond Hydrolysis, Endonucleolytic (GO:0090502)	0.4015814	0.6827915	0	0	2.061303092	1.880619508	*ZC3H12B*
Biological Process	T Cell Proliferation (GO:0042098)	0.4015814	0.6827915	0	0	2.061303092	1.880619508	*RASGRP1*
Biological Process	Regulation Of Mast Cell Degranulation (GO:0043304)	0.4015814	0.6827915	0	0	2.061303092	1.880619508	*FER*
Biological Process	Regulation Of Non-Canonical Wnt Signaling Pathway (GO:2000050)	0.4015814	0.6827915	0	0	2.061303092	1.880619508	*ZNRF3*
Biological Process	Bone Morphogenesis (GO:0060349)	0.4015814	0.6827915	0	0	2.061303092	1.880619508	*OSR2*
Biological Process	Calcium Ion-Regulated Exocytosis Of Neurotransmitter (GO:0048791)	0.4015814	0.6827915	0	0	2.061303092	1.880619508	*SYT4*
Biological Process	Cardiac Cell Development (GO:0055006)	0.4015814	0.6827915	0	0	2.061303092	1.880619508	*TTN*
Biological Process	Renal Water Homeostasis (GO:0003091)	0.4015814	0.6827915	0	0	2.061303092	1.880619508	*PRKACB*
Biological Process	Resolution Of Meiotic Recombination Intermediates (GO:0000712)	0.4015814	0.6827915	0	0	2.061303092	1.880619508	*ERCC4*
Biological Process	Respiratory Electron Transport Chain (GO:0022904)	0.4015814	0.6827915	0	0	2.061303092	1.880619508	*PPARGC1A*
Biological Process	Copper Ion Homeostasis (GO:0055070)	0.4015814	0.6827915	0	0	2.061303092	1.880619508	*XIAP*
Biological Process	Electron Transport Chain (GO:0022900)	0.4015814	0.6827915	0	0	2.061303092	1.880619508	*PPARGC1A*
Biological Process	Endoderm Development (GO:0007492)	0.4015814	0.6827915	0	0	2.061303092	1.880619508	*DUSP5*
Biological Process	Smooth Muscle Contraction (GO:0006939)	0.4015814	0.6827915	0	0	2.061303092	1.880619508	*TPCN2*
Biological Process	Establishment Of Spindle Localization (GO:0051293)	0.4015814	0.6827915	0	0	2.061303092	1.880619508	*CLASP2*
Biological Process	Synaptic Vesicle Membrane Organization (GO:0048499)	0.4015814	0.6827915	0	0	2.061303092	1.880619508	*STX3*

Biological Process	Synaptonemal Complex Assembly (GO:0007130)	0.4015814	0.6827915	0	0	2.061303092	1.880619508	*P3H4*
Biological Process	Synaptonemal Complex Organization (GO:0070193)	0.4015814	0.6827915	0	0	2.061303092	1.880619508	*P3H4*
Biological Process	Lipoprotein Biosynthetic Process (GO:0042158)	0.4015814	0.6827915	0	0	2.061303092	1.880619508	*WDR45B*
Biological Process	Mammary Gland Epithelium Development (GO:0061180)	0.4015814	0.6827915	0	0	2.061303092	1.880619508	*ZNF703*
Biological Process	Maturation Of LSU-rRNA From Tricistronic rRNA Transcript (SSU-rRNA, 5.8S rRNA, LSU-rRNA) (GO:0000463)	0.4015814	0.6827915	0	0	2.061303092	1.880619508	*RPF2*
Biological Process	Membrane Repolarization During Cardiac Muscle Cell Action Potential (GO:0086013)	0.4015814	0.6827915	0	0	2.061303092	1.880619508	*KCNJ3*
Biological Process	Microvillus Assembly (GO:0030033)	0.4015814	0.6827915	0	0	2.061303092	1.880619508	*RAPGEF6*
Biological Process	Mitotic Recombination (GO:0006312)	0.4015814	0.6827915	0	0	2.061303092	1.880619508	*SMC6*
Biological Process	Negative Regulation Of Androgen Receptor Signaling Pathway (GO:0060766)	0.4015814	0.6827915	0	0	2.061303092	1.880619508	*PIAS2*
Biological Process	Negative Regulation Of Cilium Assembly (GO:1902018)	0.4015814	0.6827915	0	0	2.061303092	1.880619508	*TBC1D30*
Biological Process	Negative Regulation Of Leukocyte Mediated Cytotoxicity (GO:0001911)	0.4015814	0.6827915	0	0	2.061303092	1.880619508	*MICA*
Biological Process	Negative Regulation Of Nucleocytoplasmic Transport (GO:0046823)	0.4015814	0.6827915	0	0	2.061303092	1.880619508	*NUP153*
Biological Process	Negative Regulation Of Peptidyl-Threonine Phosphorylation (GO:0010801)	0.4015814	0.6827915	0	0	2.061303092	1.880619508	*SPRY2*
Biological Process	Negative Regulation Of Single Stranded Viral RNA Replication Via Double Stranded DNA Intermediate (GO:0045869)	0.4015814	0.6827915	0	0	2.061303092	1.880619508	*SETDB1*
Biological Process	Negative Regulation Of Transcription Regulatory Region DNA Binding (GO:2000678)	0.4015814	0.6827915	0	0	2.061303092	1.880619508	*HEY1*
Biological Process	Nuclear Pore Organization (GO:0006999)	0.4015814	0.6827915	0	0	2.061303092	1.880619508	*NUP153*
Biological Process	Pathway-Restricted SMAD Protein Phosphorylation (GO:0060389)	0.4015814	0.6827915	0	0	2.061303092	1.880619508	*USP15*
Biological Process	Phagolysosome Assembly (GO:0001845)	0.4015814	0.6827915	0	0	2.061303092	1.880619508	*RAB14*
Biological Process	Polarized Epithelial Cell Differentiation (GO:0030859)	0.4015814	0.6827915	0	0	2.061303092	1.880619508	*ZDHHC7*
Biological Process	Positive Regulation Of Exosomal Secretion (GO:1903543)	0.4015814	0.6827915	0	0	2.061303092	1.880619508	*PDCD6IP*
Biological Process	Positive Regulation Of Glycogen Metabolic Process (GO:0070875)	0.4015814	0.6827915	0	0	2.061303092	1.880619508	*SORBS1*
Biological Process	Positive Regulation Of Long-Term Synaptic Potentiation (GO:1900273)	0.4015814	0.6827915	0	0	2.061303092	1.880619508	*RELN*
Biological Process	Positive Regulation Of Lyase Activity (GO:0051349)	0.4015814	0.6827915	0	0	2.061303092	1.880619508	*CACNA1C*
Biological Process	Positive Regulation Of Transcription From RNA Polymerase II Promoter Involved In Cellular Response To Chemical Stimulus (GO:1901522)	0.4015814	0.6827915	0	0	2.061303092	1.880619508	*SMAD5*
Biological Process	Positive Regulation Of Transporter Activity (GO:0032411)	0.4015814	0.6827915	0	0	2.061303092	1.880619508	*PPARGC1A*

Biological Process	Post-Translational Protein Targeting To Endoplasmic Reticulum Membrane (GO:0006620)	0.4015814	0.6827915	0	0	2.061303092	1.880619508	*SEC61A2*
Biological Process	Primary Alcohol Metabolic Process (GO:0034308)	0.4015814	0.6827915	0	0	2.061303092	1.880619508	*GDPD1*
Biological Process	Regulation Of Bone Remodeling (GO:0046850)	0.4015814	0.6827915	0	0	2.061303092	1.880619508	*SUCO*
Biological Process	Regulation Of Branching Involved In Ureteric Bud Morphogenesis (GO:0090189)	0.4015814	0.6827915	0	0	2.061303092	1.880619508	*SIX4*
Biological Process	Regulation Of Carbohydrate Catabolic Process (GO:0043470)	0.4015814	0.6827915	0	0	2.061303092	1.880619508	*EP300*
Biological Process	Regulation Of Cell Fate Specification (GO:0042659)	0.4015814	0.6827915	0	0	2.061303092	1.880619508	*RBBP4*
Biological Process	Positive Regulation Of T Cell Differentiation (GO:0045582)	0.4066005	0.686544	0	0	1.318265999	1.18633934	*PBRM1;ARID1A;SOX4*
Biological Process	Positive Regulation Of Cysteine-Type Endopeptidase Activity (GO:2001056)	0.4066005	0.686544	0	0	1.318265999	1.18633934	*DDX3X;APAF1;BCL10*
Biological Process	Protein N-linked Glycosylation (GO:0006487)	0.4066005	0.686544	0	0	1.318265999	1.18633934	*FUT9;GFPT1;ALG10B*
Biological Process	Mitochondrial Respiratory Chain Complex Assembly (GO:0033108)	0.4074383	0.686544	0	0	1.246849422	1.11950341	*UQCC2;NDUFAF7;NDUFC2;COA5*
Biological Process	Positive Regulation Of Transferase Activity (GO:0051347)	0.41052	0.686544	0	0	1.174201067	1.045427161	*DCUN1D5;ADIPOQ;HNRNPA2B1;MAPK1;CTNNB1;MAP3K4*
Biological Process	RNA Transport (GO:0050658)	0.4122182	0.686544	0	0	1.448693819	1.283836041	*HNRNPA2B1;NUP153*
Biological Process	Regulation Of Mitochondrial Membrane Potential (GO:0051881)	0.4122182	0.686544	0	0	1.448693819	1.283836041	*VCP;SLC25A36*
Biological Process	Anterograde Axonal Transport (GO:0008089)	0.4122182	0.686544	0	0	1.448693819	1.283836041	*AP3M1;KIF3A*
Biological Process	Regulation Of Mitotic Spindle Organization (GO:0060236)	0.4122182	0.686544	0	0	1.448693819	1.283836041	*PDCD6IP;HNRNPU*
Biological Process	Release Of Sequestered Calcium Ion Into Cytosol (GO:0051209)	0.4122182	0.686544	0	0	1.448693819	1.283836041	*PLCB4;PLCH1*
Biological Process	Vitamin Transport (GO:0051180)	0.4122182	0.686544	0	0	1.448693819	1.283836041	*SLC25A32;SLC19A2*
Biological Process	Negative Regulation Of Proteasomal Protein Catabolic Process (GO:1901799)	0.4122182	0.686544	0	0	1.448693819	1.283836041	*USP9X;WAC*
Biological Process	Negative Regulation Of Protein Secretion (GO:0050709)	0.4122182	0.686544	0	0	1.448693819	1.283836041	*HMGCR;OPRM1*
Biological Process	Negative Regulation Of Ubiquitin-Dependent Protein Catabolic Process (GO:2000059)	0.4122182	0.686544	0	0	1.448693819	1.283836041	*USP9X;WAC*
Biological Process	Neuron Projection Extension (GO:1990138)	0.4122182	0.686544	0	0	1.448693819	1.283836041	*USP9X;CTNNB1*
Biological Process	Positive Regulation Of ATP-dependent Activity (GO:0032781)	0.4122182	0.686544	0	0	1.448693819	1.283836041	*HNRNPU;TOR1AIP2*
Biological Process	Positive Regulation Of Cytokinesis (GO:0032467)	0.4122182	0.686544	0	0	1.448693819	1.283836041	*CUL3;PKN2*
Biological Process	Positive Regulation Of Intrinsic Apoptotic Signaling Pathway (GO:2001244)	0.4122182	0.686544	0	0	1.448693819	1.283836041	*SIAH1;NCK1*
Biological Process	Positive Regulation Of Lipid Biosynthetic Process (GO:0046889)	0.4122182	0.686544	0	0	1.448693819	1.283836041	*CREB1;SORBS1*
Biological Process	Positive Regulation Of MAP Kinase Activity (GO:0043406)	0.4155837	0.686544	0	0	1.232453606	1.082182105	*EPGN;TRAF6;RASGRP1;MAP3K4*
Biological Process	Autophagosome Assembly (GO:0000045)	0.4163042	0.686544	0	0	1.296936159	1.136555832	*WDR45B;SMURF1;ATG14*
Biological Process	Positive Regulation Of DNA Biosynthetic Process (GO:2000573)	0.4163042	0.686544	0	0	1.296936159	1.136555832	*MAPK1;CTNNB1;MAP3K4*
Biological Process	Positive Regulation Of Cell Cycle (GO:0045787)	0.4163042	0.686544	0	0	1.296936159	1.136555832	*PKN2;HCFC1;LRP6*

Biological Process	Actin Filament Organization (GO:0007015)	0.4169594	0.686544	0	0	1.16563147	1.019655172	*MARCKS;NEBL;MYO15A;MYO5A;PPP1R9A;TTN*
Biological Process	Regulation Of Protein Binding (GO:0043393)	0.4204854	0.686544	0	0	1.186150133	1.027615921	*GSK3B;USP9X;IDE;MARK3;SPPL3*
Biological Process	Positive Regulation Of Cell Projection Organization (GO:0031346)	0.4204854	0.686544	0	0	1.186150133	1.027615921	*RELN;FUT9;ZNF804A;EP300;PLXNC1*
Biological Process	Regulation Of Cholesterol Biosynthetic Process (GO:0045540)	0.4231395	0.686544	0	0	1.913967812	1.64611427	*PRKAA1*
Biological Process	DNA Double-Strand Break Processing (GO:0000729)	0.4231395	0.686544	0	0	1.913967812	1.64611427	*SMARCAD1*
Biological Process	Regulation Of Dephosphorylation (GO:0035303)	0.4231395	0.686544	0	0	1.913967812	1.64611427	*MTMR9*
Biological Process	Regulation Of Granulocyte Macrophage Colony-Stimulating Factor Production (GO:0032645)	0.4231395	0.686544	0	0	1.913967812	1.64611427	*RASGRP1*
Biological Process	Regulation Of Isotype Switching (GO:0045191)	0.4231395	0.686544	0	0	1.913967812	1.64611427	*KMT5B*
Biological Process	Activation Of NF-kappaB-inducing Kinase Activity (GO:0007250)	0.4231395	0.686544	0	0	1.913967812	1.64611427	*TRAF6*
Biological Process	Anterograde Synaptic Vesicle Transport (GO:0048490)	0.4231395	0.686544	0	0	1.913967812	1.64611427	*AP3M1*
Biological Process	Regulation Of Multicellular Organism Growth (GO:0040014)	0.4231395	0.686544	0	0	1.913967812	1.64611427	*MBD5*
Biological Process	Branched-Chain Amino Acid Metabolic Process (GO:0009081)	0.4231395	0.686544	0	0	1.913967812	1.64611427	*DBT*
Biological Process	Regulation Of rRNA Processing (GO:2000232)	0.4231395	0.686544	0	0	1.913967812	1.64611427	*RIOK2*
Biological Process	Cellular Response To Corticosteroid Stimulus (GO:0071384)	0.4231395	0.686544	0	0	1.913967812	1.64611427	*HNRNPU*
Biological Process	Regulation Of Systemic Arterial Blood Pressure By Hormone (GO:0001990)	0.4231395	0.686544	0	0	1.913967812	1.64611427	*AVPR1B*
Biological Process	Cellular Response To Sterol (GO:0036315)	0.4231395	0.686544	0	0	1.913967812	1.64611427	*LRP6*
Biological Process	Coenzyme A Metabolic Process (GO:0015936)	0.4231395	0.686544	0	0	1.913967812	1.64611427	*SLC25A16*
Biological Process	Dendritic Spine Morphogenesis (GO:0060997)	0.4231395	0.686544	0	0	1.913967812	1.64611427	*EPHB1*
Biological Process	Retrograde Axonal Transport (GO:0008090)	0.4231395	0.686544	0	0	1.913967812	1.64611427	*FBXW11*
Biological Process	Endoplasmic Reticulum To Cytosol Transport (GO:1903513)	0.4231395	0.686544	0	0	1.913967812	1.64611427	*VCP*
Biological Process	Endosome To Lysosome Transport Via Multivesicular Body Sorting Pathway (GO:0032510)	0.4231395	0.686544	0	0	1.913967812	1.64611427	*VCP*
Biological Process	Stem Cell Development (GO:0048864)	0.4231395	0.686544	0	0	1.913967812	1.64611427	*NOLC1*
Biological Process	Synaptic Vesicle Transport Along Microtubule (GO:0099517)	0.4231395	0.686544	0	0	1.913967812	1.64611427	*AP3M1*
Biological Process	Folic Acid-Containing Compound Metabolic Process (GO:0006760)	0.4231395	0.686544	0	0	1.913967812	1.64611427	*SLC25A32*
Biological Process	Ganglioside Biosynthetic Process (GO:0001574)	0.4231395	0.686544	0	0	1.913967812	1.64611427	*ST3GAL5*
Biological Process	Glutamine Family Amino Acid Catabolic Process (GO:0009065)	0.4231395	0.686544	0	0	1.913967812	1.64611427	*GLS*
Biological Process	mRNA Methylation (GO:0080009)	0.4231395	0.686544	0	0	1.913967812	1.64611427	*WTAP*
Biological Process	Melanosome Transport (GO:0032402)	0.4231395	0.686544	0	0	1.913967812	1.64611427	*MYO5A*
Biological Process	Midbody Abscission (GO:0061952)	0.4231395	0.686544	0	0	1.913967812	1.64611427	*PDCD6IP*
Biological Process	Mitotic Chromosome Condensation (GO:0007076)	0.4231395	0.686544	0	0	1.913967812	1.64611427	*TTN*

Biological Process	Negative Regulation Of Cell-Substrate Junction Organization (GO:0150118)	0.4231395	0.686544	0	0	1.913967812	1.64611427	*CLASP2*
Biological Process	Negative Regulation Of Focal Adhesion Assembly (GO:0051895)	0.4231395	0.686544	0	0	1.913967812	1.64611427	*CLASP2*
Biological Process	Negative Regulation Of Gluconeogenesis (GO:0045721)	0.4231395	0.686544	0	0	1.913967812	1.64611427	*EP300*
Biological Process	Negative Regulation Of Synaptic Transmission (GO:0050805)	0.4231395	0.686544	0	0	1.913967812	1.64611427	*ADIPOQ*
Biological Process	Negative Regulation Of Type I Interferon-Mediated Signaling Pathway (GO:0060339)	0.4231395	0.686544	0	0	1.913967812	1.64611427	*YTHDF3*
Biological Process	Neurotrophin Signaling Pathway (GO:0038179)	0.4231395	0.686544	0	0	1.913967812	1.64611427	*CASP3*
Biological Process	Nitric Oxide Biosynthetic Process (GO:0006809)	0.4231395	0.686544	0	0	1.913967812	1.64611427	*SLC7A6*
Biological Process	Nucleotide-Sugar Biosynthetic Process (GO:0009226)	0.4231395	0.686544	0	0	1.913967812	1.64611427	*GFPT1*
Biological Process	Phagosome Maturation (GO:0090382)	0.4231395	0.686544	0	0	1.913967812	1.64611427	*RAB14*
Biological Process	Pigment Granule Transport (GO:0051904)	0.4231395	0.686544	0	0	1.913967812	1.64611427	*MYO5A*
Biological Process	Platelet-Derived Growth Factor Receptor Signaling Pathway (GO:0048008)	0.4231395	0.686544	0	0	1.913967812	1.64611427	*FER*
Biological Process	Positive Regulation Of Collagen Biosynthetic Process (GO:0032967)	0.4231395	0.686544	0	0	1.913967812	1.64611427	*SUCO*
Biological Process	Positive Regulation Of Glucose Metabolic Process (GO:0010907)	0.4231395	0.686544	0	0	1.913967812	1.64611427	*PPARGC1A*
Biological Process	Positive Regulation Of Immunoglobulin Mediated Immune Response (GO:0002891)	0.4231395	0.686544	0	0	1.913967812	1.64611427	*KMT5B*
Biological Process	Positive Regulation Of Isotype Switching (GO:0045830)	0.4231395	0.686544	0	0	1.913967812	1.64611427	*KMT5B*
Biological Process	Positive Regulation Of Mitotic Sister Chromatid Separation (GO:1901970)	0.4231395	0.686544	0	0	1.913967812	1.64611427	*CUL3*
Biological Process	Positive Regulation Of Sprouting Angiogenesis (GO:1903672)	0.4231395	0.686544	0	0	1.913967812	1.64611427	*PDPK1*
Biological Process	Protein Localization To Microtubule Organizing Center (GO:1905508)	0.4231395	0.686544	0	0	1.913967812	1.64611427	*HOOK3*
Biological Process	Regulation Of Amyloid Fibril Formation (GO:1905906)	0.4231395	0.686544	0	0	1.913967812	1.64611427	*USP8*
Biological Process	Regulation Of Amyloid-Beta Clearance (GO:1900221)	0.4231395	0.686544	0	0	1.913967812	1.64611427	*HMGCR*
Biological Process	Regulation Of Carbohydrate Metabolic Process (GO:0006109)	0.4231395	0.686544	0	0	1.913967812	1.64611427	*FOXK1*
Biological Process	Regulation Of Cytokine-Mediated Signaling Pathway (GO:0001959)	0.4247813	0.686544	0	0	1.410496995	1.207640621	*SPATA2;HIPK1*
Biological Process	Regulation Of Protein Dephosphorylation (GO:0035304)	0.4247813	0.686544	0	0	1.410496995	1.207640621	*PPP6R2;SPPL3*
Biological Process	Synaptic Vesicle Endocytosis (GO:0048488)	0.4247813	0.686544	0	0	1.410496995	1.207640621	*ITSN1;SNAP91*
Biological Process	Water-Soluble Vitamin Metabolic Process (GO:0006767)	0.4247813	0.686544	0	0	1.410496995	1.207640621	*SLC25A32;SLC19A2*
Biological Process	Negative Regulation Of Cell-Cell Adhesion (GO:0022408)	0.4247813	0.686544	0	0	1.410496995	1.207640621	*ZNF703;MIA3*
Biological Process	Negative Regulation Of Protein Localization (GO:1903828)	0.4247813	0.686544	0	0	1.410496995	1.207640621	*GSK3B;VCP*
Biological Process	Negative Regulation Of Secretion By Cell (GO:1903531)	0.4247813	0.686544	0	0	1.410496995	1.207640621	*SYT4;HMGCR*
Biological Process	Response To Reactive Oxygen Species (GO:0000302)	0.4259558	0.6876033	0	0	1.276283456	1.089205406	*FER;MAPK1;CAMKK2*
Biological Process	Negative Regulation Of Immune Response (GO:0050777)	0.4259558	0.6876033	0	0	1.276283456	1.089205406	*PPP6C;FER;PRKDC*

Biological Process	Regulation Of Protein Kinase Activity (GO:0045859)	0.4276082	0.6898502	0	0	1.175683965	0.998799996	*RELN;PDPK1;CAMKK2;LRP6;TTN*
Biological Process	Regulation Of Peptidyl-Serine Phosphorylation (GO:0033135)	0.4317958	0.6937755	0	0	1.204632478	1.011653349	*SPRY2;RAF1;FNIP1;NCK1*
Biological Process	Positive Regulation Of Cellular Component Biogenesis (GO:0044089)	0.4317958	0.6937755	0	0	1.204632478	1.011653349	*GSK3B;VCP;PDCD6IP;FNIP1*
Biological Process	Activation Of Cysteine-Type Endopeptidase Activity Involved In Apoptotic Process (GO:0006919)	0.435551	0.6937755	0	0	1.256276151	1.044145481	*VCP;APAF1;SENP1*
Biological Process	Peptidyl-Tyrosine Phosphorylation (GO:0018108)	0.435551	0.6937755	0	0	1.256276151	1.044145481	*FER;RELN;DYRK1A*
Biological Process	Regulation Of Neuron Projection Development (GO:0010975)	0.4369445	0.6937755	0	0	1.12362372	0.930303291	*GSK3B;YTHDF1;RELN;FUT9;ZNF804A;EP300;SPOCK1*
Biological Process	Regulation Of Heart Rate By Cardiac Conduction (GO:0086091)	0.4372029	0.6937755	0	0	1.374258982	1.137003856	*CACNA1C;KCNJ3*
Biological Process	Cellular Response To Retinoic Acid (GO:0071300)	0.4372029	0.6937755	0	0	1.374258982	1.137003856	*GSK3B;CREB1*
Biological Process	Response To Amino Acid Starvation (GO:1990928)	0.4372029	0.6937755	0	0	1.374258982	1.137003856	*BMT2;MAPK1*
Biological Process	Synaptic Vesicle Exocytosis (GO:0016079)	0.4372029	0.6937755	0	0	1.374258982	1.137003856	*SYT4;STX3*
Biological Process	Visual System Development (GO:0150063)	0.4372029	0.6937755	0	0	1.374258982	1.137003856	*PBX2P1;HIPK1*
Biological Process	Negative Regulation Of DNA Recombination (GO:0045910)	0.4372029	0.6937755	0	0	1.374258982	1.137003856	*RTEL1;TERF2IP*
Biological Process	Negative Regulation Of mRNA Catabolic Process (GO:1902373)	0.4372029	0.6937755	0	0	1.374258982	1.137003856	*CIRBP;HNRNPU*
Biological Process	Non-Canonical Wnt Signaling Pathway (GO:0035567)	0.4372029	0.6937755	0	0	1.374258982	1.137003856	*FZD3;SMURF1*
Biological Process	Positive Regulation Of Actin Filament Polymerization (GO:0030838)	0.4372029	0.6937755	0	0	1.374258982	1.137003856	*FER;NCK1*
Biological Process	Positive Regulation Of Response To Biotic Stimulus (GO:0002833)	0.4372029	0.6937755	0	0	1.374258982	1.137003856	*POLR3C;POLR3F*
Biological Process	Protein Localization To Lysosome (GO:0061462)	0.4372029	0.6937755	0	0	1.374258982	1.137003856	*AP3M1;ATG14*
Biological Process	Regulation Of Amide Metabolic Process (GO:0034248)	0.4372029	0.6937755	0	0	1.374258982	1.137003856	*NOLC1;MSI2*
Biological Process	B Cell Activation Involved In Immune Response (GO:0002312)	0.4439221	0.6937755	0	0	1.786277237	1.450646926	*RNF8*
Biological Process	Regulation Of Establishment Of Endothelial Barrier (GO:1903140)	0.4439221	0.6937755	0	0	1.786277237	1.450646926	*ZDHHC21*
Biological Process	Regulation Of Fatty Acid Oxidation (GO:0046320)	0.4439221	0.6937755	0	0	1.786277237	1.450646926	*PPARGC1A*
Biological Process	Apoptotic Nuclear Changes (GO:0030262)	0.4439221	0.6937755	0	0	1.786277237	1.450646926	*DFFB*
Biological Process	Regulation Of Natural Killer Cell Activation (GO:0032814)	0.4439221	0.6937755	0	0	1.786277237	1.450646926	*MICA*
Biological Process	Autonomic Nervous System Development (GO:0048483)	0.4439221	0.6937755	0	0	1.786277237	1.450646926	*SOX4*
Biological Process	Regulation Of Protein Kinase A Signaling (GO:0010738)	0.4439221	0.6937755	0	0	1.786277237	1.450646926	*ADIPOQ*
Biological Process	Cell Communication By Electrical Coupling Involved In Cardiac Conduction (GO:0086064)	0.4439221	0.6937755	0	0	1.786277237	1.450646926	*CACNA1C*
Biological Process	Cell Surface Receptor Signaling Pathway Involved In Heart Development (GO:0061311)	0.4439221	0.6937755	0	0	1.786277237	1.450646926	*HEY1*
Biological Process	Cellular Response To Nitrogen Compound (GO:1901699)	0.4439221	0.6937755	0	0	1.786277237	1.450646926	*ATRX*

Biological Process	Establishment Of Melanosome Localization (GO:0032401)	0.4439221	0.6937755	0	0	1.786277237	1.450646926	*MYO5A*
Biological Process	Establishment Of Protein Localization To Endoplasmic Reticulum (GO:0072599)	0.4439221	0.6937755	0	0	1.786277237	1.450646926	*RAB3GAP2*
Biological Process	Extrinsic Apoptotic Signaling Pathway In Absence Of Ligand (GO:0097192)	0.4439221	0.6937755	0	0	1.786277237	1.450646926	*GSK3B*
Biological Process	Glycoprotein Catabolic Process (GO:0006516)	0.4439221	0.6937755	0	0	1.786277237	1.450646926	*FBXO6*
Biological Process	Intracellular Cholesterol Transport (GO:0032367)	0.4439221	0.6937755	0	0	1.786277237	1.450646926	*LRP6*
Biological Process	Intraciliary Anterograde Transport (GO:0035720)	0.4439221	0.6937755	0	0	1.786277237	1.450646926	*TTC26*
Biological Process	Maturation Of 5.8S rRNA (GO:0000460)	0.4439221	0.6937755	0	0	1.786277237	1.450646926	*PRKDC*
Biological Process	Membrane Depolarization During Cardiac Muscle Cell Action Potential (GO:0086012)	0.4439221	0.6937755	0	0	1.786277237	1.450646926	*CACNA1C*
Biological Process	Mitophagy (GO:0000423)	0.4439221	0.6937755	0	0	1.786277237	1.450646926	*ATG14*
Biological Process	Negative Regulation Of RNA Biosynthetic Process (GO:1902679)	0.4439221	0.6937755	0	0	1.786277237	1.450646926	*SETDB1*
Biological Process	Negative Regulation Of Amyloid-Beta Formation (GO:1902430)	0.4439221	0.6937755	0	0	1.786277237	1.450646926	*RTN1*
Biological Process	Negative Regulation Of Intrinsic Apoptotic Signaling Pathway By P53 Class Mediator (GO:1902254)	0.4439221	0.6937755	0	0	1.786277237	1.450646926	*ING2*
Biological Process	Negative Regulation Of Natural Killer Cell Mediated Cytotoxicity (GO:0045953)	0.4439221	0.6937755	0	0	1.786277237	1.450646926	*MICA*
Biological Process	Negative Regulation Of Phosphatase Activity (GO:0010923)	0.4439221	0.6937755	0	0	1.786277237	1.450646926	*GSK3B*
Biological Process	Neuron Projection Organization (GO:0106027)	0.4439221	0.6937755	0	0	1.786277237	1.450646926	*GSK3B*
Biological Process	Nitric Oxide Metabolic Process (GO:0046209)	0.4439221	0.6937755	0	0	1.786277237	1.450646926	*SLC7A6*
Biological Process	Pharyngeal System Development (GO:0060037)	0.4439221	0.6937755	0	0	1.786277237	1.450646926	*SIX4*
Biological Process	Positive Regulation Of Autophagosome Assembly (GO:2000786)	0.4439221	0.6937755	0	0	1.786277237	1.450646926	*RAB3GAP2*
Biological Process	Positive Regulation Of Collagen Metabolic Process (GO:0010714)	0.4439221	0.6937755	0	0	1.786277237	1.450646926	*SUCO*
Biological Process	Positive Regulation Of Muscle Hypertrophy (GO:0014742)	0.4439221	0.6937755	0	0	1.786277237	1.450646926	*MEF2A*
Biological Process	Prostanoid Metabolic Process (GO:0006692)	0.4439221	0.6937755	0	0	1.786277237	1.450646926	*PLAA*
Biological Process	Protein O-linked Mannosylation (GO:0035269)	0.4439221	0.6937755	0	0	1.786277237	1.450646926	*TMTC3*
Biological Process	Protein Alpha-1,2-Demannosylation (GO:0036508)	0.4439221	0.6937755	0	0	1.786277237	1.450646926	*EDEM3*
Biological Process	Protein Localization To Endoplasmic Reticulum (GO:0070972)	0.4439221	0.6937755	0	0	1.786277237	1.450646926	*MIA3*
Biological Process	Protein Targeting To Peroxisome (GO:0006625)	0.4439221	0.6937755	0	0	1.786277237	1.450646926	*PEX3*
Biological Process	Regulation Of Cardiac Muscle Contraction By Regulation Of The Release Of Sequestered Calcium Ion (GO:0010881)	0.4439221	0.6937755	0	0	1.786277237	1.450646926	*CACNA1C*
Biological Process	Positive Regulation Of Transmembrane Receptor Protein Serine/Threonine Kinase Signaling Pathway (GO:0090100)	0.447884	0.6995546	0	0	1.178034256	0.946221993	*ING2;EP300;RNF111;SH2B1*
Biological Process	DNA Recombination (GO:0006310)	0.4494772	0.6995681	0	0	1.339832869	1.071424487	*RTEL1;TSN*

Biological Process	Regulation Of Neurotransmitter Receptor Activity (GO:0099601)	0.4494772	0.6995681	0	0	1.339832869	1.071424487	*RELN;OPRM1*
Biological Process	Cardiac Ventricle Morphogenesis (GO:0003208)	0.4494772	0.6995681	0	0	1.339832869	1.071424487	*HEY1;SOX4*
Biological Process	Translational Elongation (GO:0006414)	0.4494772	0.6995681	0	0	1.339832869	1.071424487	*EEF2K;PELO*
Biological Process	Potassium Ion Import Across Plasma Membrane (GO:1990573)	0.4494772	0.6995681	0	0	1.339832869	1.071424487	*SLC12A6;KCNJ3*
Biological Process	Regulation Of Axonogenesis (GO:0050770)	0.4494772	0.6995681	0	0	1.339832869	1.071424487	*GSK3B;PLXNC1*
Biological Process	Regulation Of Heart Contraction (GO:0008016)	0.454556	0.6999004	0	0	1.218080385	0.960376158	*MEF2A;ATP2B2;CACNA1C*
Biological Process	Regulation Of Cell Motility (GO:2000145)	0.455906	0.6999004	0	0	1.13559322	0.891972792	*SPOCK2;PKN2;PLXNC1;AMOTL1;RAF1*
Biological Process	DNA-templated DNA Replication Maintenance Of Fidelity (GO:0045005)	0.4615984	0.6999004	0	0	1.307086079	1.010456035	*RTEL1;ATRX*
Biological Process	Energy Derivation By Oxidation Of Organic Compounds (GO:0015980)	0.4615984	0.6999004	0	0	1.307086079	1.010456035	*GFPT1;PPARGC1A*
Biological Process	Learning (GO:0007612)	0.4615984	0.6999004	0	0	1.307086079	1.010456035	*YTHDF1;ATXN1*
Biological Process	mRNA Transcription (GO:0009299)	0.4615984	0.6999004	0	0	1.307086079	1.010456035	*CREB1;TAF8*
Biological Process	Negative Regulation Of BMP Signaling Pathway (GO:0030514)	0.4615984	0.6999004	0	0	1.307086079	1.010456035	*SMURF1;VWC2*
Biological Process	Positive Regulation Of T Cell Mediated Immunity (GO:0002711)	0.4615984	0.6999004	0	0	1.307086079	1.010456035	*TRAF6;MICA*
Biological Process	Positive Regulation Of Cell Division (GO:0051781)	0.4615984	0.6999004	0	0	1.307086079	1.010456035	*CUL3;PKN2*
Biological Process	Positive Regulation Of miRNA Transcription (GO:1902895)	0.4615984	0.6999004	0	0	1.307086079	1.010456035	*POU2F1;STAT3*
Biological Process	Regulation Of JUN Kinase Activity (GO:0043506)	0.4615984	0.6999004	0	0	1.307086079	1.010456035	*TRAF6;MAP3K4*
Biological Process	Regulation Of Cell-Cell Adhesion (GO:0022407)	0.4615984	0.6999004	0	0	1.307086079	1.010456035	*FUT9;CD47*
Biological Process	Regulation Of Cellular Component Size (GO:0032535)	0.4615984	0.6999004	0	0	1.307086079	1.010456035	*HP1BP3;ARHGAP5*
Biological Process	Cellular Response To Lipopolysaccharide (GO:0071222)	0.4629224	0.6999004	0	0	1.125991655	0.86723399	*TRAF6;PLAA;BCL10;CXCL13;MTDH*
Biological Process	Neuron Projection Guidance (GO:0097485)	0.4629224	0.6999004	0	0	1.125991655	0.86723399	*RELN;SIAH1;CNTN4;SOS1;EPHB1*
Biological Process	DNA Replication Checkpoint Signaling (GO:0000076)	0.4639569	0.6999004	0	0	1.674547983	1.285992079	*ORC1*
Biological Process	G Protein-Coupled Acetylcholine Receptor Signaling Pathway (GO:0007213)	0.4639569	0.6999004	0	0	1.674547983	1.285992079	*OPRM1*
Biological Process	Golgi To Vacuole Transport (GO:0006896)	0.4639569	0.6999004	0	0	1.674547983	1.285992079	*AP1G1*
Biological Process	Aorta Morphogenesis (GO:0035909)	0.4639569	0.6999004	0	0	1.674547983	1.285992079	*HEY1*
Biological Process	Branched-Chain Amino Acid Catabolic Process (GO:0009083)	0.4639569	0.6999004	0	0	1.674547983	1.285992079	*DBT*
Biological Process	Carbohydrate Derivative Catabolic Process (GO:1901136)	0.4639569	0.6999004	0	0	1.674547983	1.285992079	*FBXO6*
Biological Process	Regulation Of Protein Sumoylation (GO:0033233)	0.4639569	0.6999004	0	0	1.674547983	1.285992079	*CTNNB1*
Biological Process	Cell Migration Involved In Sprouting Angiogenesis (GO:0002042)	0.4639569	0.6999004	0	0	1.674547983	1.285992079	*MIA3*
Biological Process	Regulation Of Single Stranded Viral RNA Replication Via Double Stranded DNA Intermediate (GO:0045091)	0.4639569	0.6999004	0	0	1.674547983	1.285992079	*SETDB1*
Biological Process	Cellular Response To Alkaloid (GO:0071312)	0.4639569	0.6999004	0	0	1.674547983	1.285992079	*CASP3*
Biological Process	Cellular Response To Glucocorticoid Stimulus (GO:0071385)	0.4639569	0.6999004	0	0	1.674547983	1.285992079	*HNRNPU*

Biological Process	Regulation Of Telomerase RNA Localization To Cajal Body (GO:1904872)	0.4639569	0.6999004	0	0	1.674547983	1.285992079	*DCP2*
Biological Process	Regulation Of Ubiquitin Protein Ligase Activity (GO:1904666)	0.4639569	0.6999004	0	0	1.674547983	1.285992079	*BTRC*
Biological Process	Regulation Of Viral Transcription (GO:0046782)	0.4639569	0.6999004	0	0	1.674547983	1.285992079	*MID2*
Biological Process	Retrograde Protein Transport, ER To Cytosol (GO:0030970)	0.4639569	0.6999004	0	0	1.674547983	1.285992079	*VCP*
Biological Process	Embryonic Digit Morphogenesis (GO:0042733)	0.4639569	0.6999004	0	0	1.674547983	1.285992079	*OSR2*
Biological Process	Transcription By RNA Polymerase I (GO:0006360)	0.4639569	0.6999004	0	0	1.674547983	1.285992079	*RRN3*
Biological Process	Triglyceride Biosynthetic Process (GO:0019432)	0.4639569	0.6999004	0	0	1.674547983	1.285992079	*GPAT3*
Biological Process	Growth Hormone Receptor Signaling Pathway (GO:0060396)	0.4639569	0.6999004	0	0	1.674547983	1.285992079	*STAT3*
Biological Process	Hippo Signaling (GO:0035329)	0.4639569	0.6999004	0	0	1.674547983	1.285992079	*AMOTL1*
Biological Process	Intracellular Zinc Ion Homeostasis (GO:0006882)	0.4639569	0.6999004	0	0	1.674547983	1.285992079	*SLC30A5*
Biological Process	Mesenchyme Development (GO:0060485)	0.4639569	0.6999004	0	0	1.674547983	1.285992079	*SOX4*
Biological Process	Muscle Tissue Morphogenesis (GO:0060415)	0.4639569	0.6999004	0	0	1.674547983	1.285992079	*TTN*
Biological Process	Neuron Maturation (GO:0042551)	0.4639569	0.6999004	0	0	1.674547983	1.285992079	*ADGRB3*
Biological Process	Peroxisomal Membrane Transport (GO:0015919)	0.4639569	0.6999004	0	0	1.674547983	1.285992079	*PEX3*
Biological Process	Positive Regulation Of SMAD Protein Signal Transduction (GO:0060391)	0.4639569	0.6999004	0	0	1.674547983	1.285992079	*SH2B1*
Biological Process	Positive Regulation Of Amyloid-Beta Formation (GO:1902004)	0.4639569	0.6999004	0	0	1.674547983	1.285992079	*CASP3*
Biological Process	Positive Regulation Of Cardiac Muscle Hypertrophy (GO:0010613)	0.4639569	0.6999004	0	0	1.674547983	1.285992079	*MEF2A*
Biological Process	Positive Regulation Of Cell Adhesion Mediated By Integrin (GO:0033630)	0.4639569	0.6999004	0	0	1.674547983	1.285992079	*CXCL13*
Biological Process	Positive Regulation Of Cell Cycle Phase Transition (GO:1901989)	0.4639569	0.6999004	0	0	1.674547983	1.285992079	*CUL3*
Biological Process	Positive Regulation Of Fatty Acid Metabolic Process (GO:0045923)	0.4639569	0.6999004	0	0	1.674547983	1.285992079	*PPARGC1A*
Biological Process	Positive Regulation Of Leukocyte Proliferation (GO:0070665)	0.4639569	0.6999004	0	0	1.674547983	1.285992079	*MAPK1*
Biological Process	Positive Regulation Of Lymphocyte Chemotaxis (GO:0140131)	0.4639569	0.6999004	0	0	1.674547983	1.285992079	*CXCL13*
Biological Process	Positive Regulation Of Nitric-Oxide Synthase Activity (GO:0051000)	0.4639569	0.6999004	0	0	1.674547983	1.285992079	*PIK3CB*
Biological Process	Protein Localization To Centrosome (GO:0071539)	0.4639569	0.6999004	0	0	1.674547983	1.285992079	*HOOK3*
Biological Process	rRNA Transcription (GO:0009303)	0.4639569	0.6999004	0	0	1.674547983	1.285992079	*GTF3C4*
Biological Process	Regulation Of Brown Fat Cell Differentiation (GO:0090335)	0.4639569	0.6999004	0	0	1.674547983	1.285992079	*HNRNPU*
Biological Process	Sensory Organ Development (GO:0007423)	0.4639581	0.6999004	0	0	1.199837632	0.921428522	*PBX2P1;SIX4;HIPK1*
Biological Process	Regulation Of Actin Filament Polymerization (GO:0030833)	0.4639581	0.6999004	0	0	1.199837632	0.921428522	*FER;RASA1;NCK1*
Biological Process	Cellular Response To Organic Cyclic Compound (GO:0071407)	0.4680477	0.7056679	0	0	1.101300794	0.836091045	*CREB1;DHX8;CASP3;ZNF703;LCOR;CTNNB1*

Biological Process	Regulation Of Hydrolase Activity (GO:0051336)	0.4732885	0.7076751	0	0	1.18213143	0.884293474	*RAB3GAP2;PLXNC1;RAPGEF6*
Biological Process	Kidney Development (GO:0001822)	0.4732885	0.7076751	0	0	1.18213143	0.884293474	*OSR2;PYGO2;SOX4*
Biological Process	Negative Regulation Of Supramolecular Fiber Organization (GO:1902904)	0.4732885	0.7076751	0	0	1.18213143	0.884293474	*DYRK1A;ARPIN;CLASP2*
Biological Process	Positive Regulation Of Cell-Substrate Adhesion (GO:0010811)	0.4732885	0.7076751	0	0	1.18213143	0.884293474	*GSK3B;ARHGEF7;CRKL*
Biological Process	Regulation Of Insulin Receptor Signaling Pathway (GO:0046626)	0.4735618	0.7076751	0	0	1.27589866	0.953699749	*SORBS1;NCK1*
Biological Process	Regulation Of Viral Entry Into Host Cell (GO:0046596)	0.4735618	0.7076751	0	0	1.27589866	0.953699749	*TMPRSS4;MID2*
Biological Process	Nucleobase-Containing Compound Catabolic Process (GO:0034655)	0.4735618	0.7076751	0	0	1.27589866	0.953699749	*DFFB;DIS3*
Biological Process	Phosphatidylinositol Phosphate Biosynthetic Process (GO:0046854)	0.4735618	0.7076751	0	0	1.27589866	0.953699749	*EFR3A;PIK3CB*
Biological Process	Positive Regulation Of Osteoblast Differentiation (GO:0045669)	0.4735618	0.7076751	0	0	1.27589866	0.953699749	*ZHX3;SUCO*
Biological Process	Regulation Of Cellular Component Organization (GO:0051128)	0.4743581	0.7076751	0	0	1.093751786	0.815712273	*SETD5;DCUN1D5;HNRNPU;SPOCK1;MLLT3;HCFC1*
Biological Process	Regulation Of Collagen Biosynthetic Process (GO:0032965)	0.4832708	0.7076751	0	0	1.575963348	1.146006105	*SUCO*
Biological Process	Regulation Of Defense Response To Virus (GO:0050688)	0.4832708	0.7076751	0	0	1.575963348	1.146006105	*ELMOD2*
Biological Process	Regulation Of Exosomal Secretion (GO:1903541)	0.4832708	0.7076751	0	0	1.575963348	1.146006105	*PDCD6IP*
Biological Process	Regulation Of Histone Modification (GO:0031056)	0.4832708	0.7076751	0	0	1.575963348	1.146006105	*WDR5*
Biological Process	Regulation Of Lymphocyte Proliferation (GO:0050670)	0.4832708	0.7076751	0	0	1.575963348	1.146006105	*CD209*
Biological Process	Adrenergic Receptor Signaling Pathway (GO:0071875)	0.4832708	0.7076751	0	0	1.575963348	1.146006105	*ZDHHC21*
Biological Process	Regulation Of Mitochondrial Membrane Permeability (GO:0046902)	0.4832708	0.7076751	0	0	1.575963348	1.146006105	*THEM4*
Biological Process	Autophagy Of Nucleus (GO:0044804)	0.4832708	0.7076751	0	0	1.575963348	1.146006105	*WDR45B*
Biological Process	Cardiac Muscle Cell Contraction (GO:0086003)	0.4832708	0.7076751	0	0	1.575963348	1.146006105	*CACNA1C*
Biological Process	Regulation Of Protein Neddylation (GO:2000434)	0.4832708	0.7076751	0	0	1.575963348	1.146006105	*DCUN1D5*
Biological Process	Cellular Response To Inorganic Substance (GO:0071241)	0.4832708	0.7076751	0	0	1.575963348	1.146006105	*ATRX*
Biological Process	Regulation Of Vascular Endothelial Cell Proliferation (GO:1905562)	0.4832708	0.7076751	0	0	1.575963348	1.146006105	*PDPK1*
Biological Process	Response To Axon Injury (GO:0048678)	0.4832708	0.7076751	0	0	1.575963348	1.146006105	*NREP*
Biological Process	Response To Cholesterol (GO:0070723)	0.4832708	0.7076751	0	0	1.575963348	1.146006105	*LRP6*
Biological Process	Response To Ethanol (GO:0045471)	0.4832708	0.7076751	0	0	1.575963348	1.146006105	*OPRM1*
Biological Process	Excitatory Postsynaptic Potential (GO:0060079)	0.4832708	0.7076751	0	0	1.575963348	1.146006105	*GSK3B*
Biological Process	Glycerolipid Catabolic Process (GO:0046503)	0.4832708	0.7076751	0	0	1.575963348	1.146006105	*GDPD1*
Biological Process	Heart Trabecula Morphogenesis (GO:0061384)	0.4832708	0.7076751	0	0	1.575963348	1.146006105	*HEY1*
Biological Process	Intracellular Sodium Ion Homeostasis (GO:0006883)	0.4832708	0.7076751	0	0	1.575963348	1.146006105	*TMPRSS4*
Biological Process	Melanosome Localization (GO:0032400)	0.4832708	0.7076751	0	0	1.575963348	1.146006105	*MYO5A*

Biological Process	Mesenchyme Morphogenesis (GO:0072132)	0.4832708	0.7076751	0	0	1.575963348	1.146006105	*HEY1*
Biological Process	Mitochondrial Fusion (GO:0008053)	0.4832708	0.7076751	0	0	1.575963348	1.146006105	*CHCHD3*
Biological Process	Myoblast Fusion (GO:0007520)	0.4832708	0.7076751	0	0	1.575963348	1.146006105	*ADGRB3*
Biological Process	Negative Regulation Of ERBB Signaling Pathway (GO:1901185)	0.4832708	0.7076751	0	0	1.575963348	1.146006105	*CBL*
Biological Process	Negative Regulation Of Amyloid Precursor Protein Catabolic Process (GO:1902992)	0.4832708	0.7076751	0	0	1.575963348	1.146006105	*RTN1*
Biological Process	Negative Regulation Of Nervous System Development (GO:0051961)	0.4832708	0.7076751	0	0	1.575963348	1.146006105	*HOOK3*
Biological Process	Negative Regulation Of Signal Transduction By P53 Class Mediator (GO:1901797)	0.4832708	0.7076751	0	0	1.575963348	1.146006105	*DYRK1A*
Biological Process	Negative Regulation Of Viral Entry Into Host Cell (GO:0046597)	0.4832708	0.7076751	0	0	1.575963348	1.146006105	*MID2*
Biological Process	Nucleosome Disassembly (GO:0006337)	0.4832708	0.7076751	0	0	1.575963348	1.146006105	*SUPT16H*
Biological Process	Positive Regulation Of CREB Transcription Factor Activity (GO:0032793)	0.4832708	0.7076751	0	0	1.575963348	1.146006105	*RELN*
Biological Process	Positive Regulation Of DNA Replication (GO:0045740)	0.4832708	0.7076751	0	0	1.575963348	1.146006105	*BRPF3*
Biological Process	Positive Regulation Of NLRP3 Inflammasome Complex Assembly (GO:1900227)	0.4832708	0.7076751	0	0	1.575963348	1.146006105	*DDX3X*
Biological Process	Positive Regulation Of Chromosome Segregation (GO:0051984)	0.4832708	0.7076751	0	0	1.575963348	1.146006105	*SMC6*
Biological Process	Positive Regulation Of Cytokine Production Involved In Inflammatory Response (GO:1900017)	0.4832708	0.7076751	0	0	1.575963348	1.146006105	*STAT3*
Biological Process	Positive Regulation Of Establishment Of Protein Localization (GO:1904951)	0.4832708	0.7076751	0	0	1.575963348	1.146006105	*CEP120*
Biological Process	Positive Regulation Of Extracellular Matrix Organization (GO:1903055)	0.4832708	0.7076751	0	0	1.575963348	1.146006105	*CLASP2*
Biological Process	Positive Regulation Of Macrophage Migration (GO:1905523)	0.4832708	0.7076751	0	0	1.575963348	1.146006105	*MAPK1*
Biological Process	Positive Regulation Of Osteoclast Differentiation (GO:0045672)	0.4832708	0.7076751	0	0	1.575963348	1.146006105	*TRAF6*
Biological Process	Pulmonary Valve Morphogenesis (GO:0003184)	0.4832708	0.7076751	0	0	1.575963348	1.146006105	*HEY1*
Biological Process	Regulation Of DNA Damage Checkpoint (GO:2000001)	0.4832708	0.7076751	0	0	1.575963348	1.146006105	*FEM1B*
Biological Process	Regulation Of T Cell Cytokine Production (GO:0002724)	0.4832708	0.7076751	0	0	1.575963348	1.146006105	*TRAF6*
Biological Process	Regulation Of cAMP-mediated Signaling (GO:0043949)	0.4832708	0.7076751	0	0	1.575963348	1.146006105	*PDE3B*
Biological Process	Regulation Of Cellular Metabolic Process (GO:0031323)	0.4853629	0.7091706	0	0	1.246161819	0.900798591	*ING2;EP300*
Biological Process	Chromosome Organization (GO:0051276)	0.4853629	0.7091706	0	0	1.246161819	0.900798591	*CENPH;KAT6A*
Biological Process	Negative Regulation Of Response To Biotic Stimulus (GO:0002832)	0.4853629	0.7091706	0	0	1.246161819	0.900798591	*PPP6C;PRKDC*
Biological Process	Positive Regulation Of Innate Immune Response (GO:0045089)	0.4853629	0.7091706	0	0	1.246161819	0.900798591	*POLR3C;POLR3F*
Biological Process	Cellular Response To Reactive Oxygen Species (GO:0034614)	0.4917214	0.7151727	0	0	1.148236701	0.815067789	*FER;MAPK1;CAMKK2*
Biological Process	Ubiquitin-Dependent ERAD Pathway (GO:0030433)	0.4917214	0.7151727	0	0	1.148236701	0.815067789	*VCP;JKAMP;FBXO6*

Biological Process	Protein Tetramerization (GO:0051262)	0.4917214	0.7151727	0	0	1.148236701	0.815067789	*CPSF6;TNPO1;GLS*
Biological Process	Regulation Of Rho Protein Signal Transduction (GO:0035023)	0.4917214	0.7151727	0	0	1.148236701	0.815067789	*CUL3;RAF1;SCAI*
Biological Process	Regulation Of Cytokine Production Involved In Inflammatory Response (GO:1900015)	0.496998	0.7151727	0	0	1.217776652	0.851432099	*STAT3;APPL1*
Biological Process	Regulation Of Epidermal Growth Factor Receptor Signaling Pathway (GO:0042058)	0.496998	0.7151727	0	0	1.217776652	0.851432099	*FER;CBL*
Biological Process	Regulation Of Interleukin-10 Production (GO:0032653)	0.496998	0.7151727	0	0	1.217776652	0.851432099	*STAT3;CD47*
Biological Process	Replication Fork Processing (GO:0031297)	0.496998	0.7151727	0	0	1.217776652	0.851432099	*RTEL1;ATRX*
Biological Process	Vacuolar Transport (GO:0007034)	0.496998	0.7151727	0	0	1.217776652	0.851432099	*AP1G1;VPS13A*
Biological Process	Male Gonad Development (GO:0008584)	0.496998	0.7151727	0	0	1.217776652	0.851432099	*ING2;SIX4*
Biological Process	Myofibril Assembly (GO:0030239)	0.496998	0.7151727	0	0	1.217776652	0.851432099	*SIX4;TTN*
Biological Process	Positive Regulation Of Cell-Cell Adhesion (GO:0022409)	0.496998	0.7151727	0	0	1.217776652	0.851432099	*CD47;CXCL13*
Biological Process	Negative Regulation Of Cell Population Proliferation (GO:0008285)	0.4993082	0.7151727	0	0	1.027630098	0.713721751	*PBRM1;KDM2B;CELF1;OPRM1;TFAP4;RBBP4;TMEM127;PHF14;CTNNB1;RAF1;PPARGC1A;ZNF268;SOX4;VGLL4*
Biological Process	Regulation Of Centriole Replication (GO:0046599)	0.5018898	0.7151727	0	0	1.488332561	1.026018955	*CEP76*
Biological Process	Arp2/3 Complex-Mediated Actin Nucleation (GO:0034314)	0.5018898	0.7151727	0	0	1.488332561	1.026018955	*JMY*
Biological Process	DNA Catabolic Process (GO:0006308)	0.5018898	0.7151727	0	0	1.488332561	1.026018955	*DFFB*
Biological Process	Regulation Of Early Endosome To Late Endosome Transport (GO:2000641)	0.5018898	0.7151727	0	0	1.488332561	1.026018955	*MAPK1*
Biological Process	Regulation Of Granulocyte Chemotaxis (GO:0071622)	0.5018898	0.7151727	0	0	1.488332561	1.026018955	*MAPK1*
Biological Process	RNA-templated DNA Biosynthetic Process (GO:0006278)	0.5018898	0.7151727	0	0	1.488332561	1.026018955	*TERF2IP*
Biological Process	Regulation Of Insulin-Like Growth Factor Receptor Signaling Pathway (GO:0043567)	0.5018898	0.7151727	0	0	1.488332561	1.026018955	*PHIP*
Biological Process	Regulation Of Lymphocyte Differentiation (GO:0045619)	0.5018898	0.7151727	0	0	1.488332561	1.026018955	*PRKDC*
Biological Process	Bicarbonate Transport (GO:0015701)	0.5018898	0.7151727	0	0	1.488332561	1.026018955	*SLC4A4*
Biological Process	Cardiac Ventricle Development (GO:0003231)	0.5018898	0.7151727	0	0	1.488332561	1.026018955	*HEY1*
Biological Process	Regulation Of Tissue Remodeling (GO:0034103)	0.5018898	0.7151727	0	0	1.488332561	1.026018955	*SUCO*
Biological Process	Eye Morphogenesis (GO:0048592)	0.5018898	0.7151727	0	0	1.488332561	1.026018955	*STAT3*
Biological Process	tRNA Metabolic Process (GO:0006399)	0.5018898	0.7151727	0	0	1.488332561	1.026018955	*SSB*
Biological Process	Fluid Transport (GO:0042044)	0.5018898	0.7151727	0	0	1.488332561	1.026018955	*TNPO1*
Biological Process	Telomere Maintenance Via Telomerase (GO:0007004)	0.5018898	0.7151727	0	0	1.488332561	1.026018955	*TERF2IP*
Biological Process	Ganglioside Metabolic Process (GO:0001573)	0.5018898	0.7151727	0	0	1.488332561	1.026018955	*ST3GAL5*
Biological Process	Glycogen Metabolic Process (GO:0005977)	0.5018898	0.7151727	0	0	1.488332561	1.026018955	*GSK3B*
Biological Process	Vesicle Docking (GO:0048278)	0.5018898	0.7151727	0	0	1.488332561	1.026018955	*STX3*
Biological Process	Water Transport (GO:0006833)	0.5018898	0.7151727	0	0	1.488332561	1.026018955	*TNPO1*
Biological Process	Interleukin-1-Mediated Signaling Pathway (GO:0070498)	0.5018898	0.7151727	0	0	1.488332561	1.026018955	*TRAF6*

Biological Process	Mesoderm Development (GO:0007498)	0.5018898	0.7151727	0	0	1.488332561	1.026018955	*HES7*
Biological Process	Multicellular Organismal-Level Water Homeostasis (GO:0050891)	0.5018898	0.7151727	0	0	1.488332561	1.026018955	*PRKACB*
Biological Process	Negative Regulation Of Cyclin-Dependent Protein Serine/Threonine Kinase Activity (GO:0045736)	0.5018898	0.7151727	0	0	1.488332561	1.026018955	*TFAP4*
Biological Process	Negative Regulation Of Execution Phase Of Apoptosis (GO:1900118)	0.5018898	0.7151727	0	0	1.488332561	1.026018955	*FZD3*
Biological Process	Negative Regulation Of Leukocyte Cell-Cell Adhesion (GO:1903038)	0.5018898	0.7151727	0	0	1.488332561	1.026018955	*MIA3*
Biological Process	Negative Regulation Of Peptidyl-Serine Phosphorylation (GO:0033137)	0.5018898	0.7151727	0	0	1.488332561	1.026018955	*NCK1*
Biological Process	Negative Regulation Of Protein Dephosphorylation (GO:0035308)	0.5018898	0.7151727	0	0	1.488332561	1.026018955	*GSK3B*
Biological Process	Neuron Apoptotic Process (GO:0051402)	0.5018898	0.7151727	0	0	1.488332561	1.026018955	*SIAH1*
Biological Process	Pore Complex Assembly (GO:0046931)	0.5018898	0.7151727	0	0	1.488332561	1.026018955	*NUP153*
Biological Process	Positive Regulation Of calcineurin-NFAT Signaling Cascade (GO:0070886)	0.5018898	0.7151727	0	0	1.488332561	1.026018955	*SPPL3*
Biological Process	Positive Regulation Of Calcineurin-Mediated Signaling (GO:0106058)	0.5018898	0.7151727	0	0	1.488332561	1.026018955	*SPPL3*
Biological Process	Protein Heterooligomerization (GO:0051291)	0.5018898	0.7151727	0	0	1.488332561	1.026018955	*CPSF6*
Biological Process	Regulation Of Autophagy Of Mitochondrion (GO:1903146)	0.5018898	0.7151727	0	0	1.488332561	1.026018955	*CAMKK2*
Biological Process	Regulation Of Protein Secretion (GO:0050708)	0.5029188	0.7162537	0	0	1.09348991	0.751584692	*UQCC2;HMGCR;CLOCK;TTN*
Biological Process	Golgi To Plasma Membrane Transport (GO:0006893)	0.5084636	0.7214356	0	0	1.190653049	0.805311969	*ARFGEF2;EXOC6*
Biological Process	Bicellular Tight Junction Assembly (GO:0070830)	0.5084636	0.7214356	0	0	1.190653049	0.805311969	*PDCD6IP;CLDN18*
Biological Process	Response To Organonitrogen Compound (GO:0010243)	0.5084636	0.7214356	0	0	1.190653049	0.805311969	*VCP;CREB1*
Biological Process	Positive Regulation Of Epithelial To Mesenchymal Transition (GO:0010718)	0.5084636	0.7214356	0	0	1.190653049	0.805311969	*ZNF703;CTNNB1*
Biological Process	Positive Regulation Of Lymphocyte Activation (GO:0051251)	0.5084636	0.7214356	0	0	1.190653049	0.805311969	*PRKDC;CD47*
Biological Process	Protein Monoubiquitination (GO:0006513)	0.5084636	0.7214356	0	0	1.190653049	0.805311969	*UBE2W;WAC*
Biological Process	Regulation Of T Cell Differentiation (GO:0045580)	0.5084636	0.7214356	0	0	1.190653049	0.805311969	*PBRM1;ARID1A*
Biological Process	Regulation Of Plasma Membrane Bounded Cell Projection Assembly (GO:0120032)	0.5098304	0.7221042	0	0	1.116225012	0.751975233	*TBC1D1;FER;EVI5*
Biological Process	Regulation Of Stress Fiber Assembly (GO:0051492)	0.5098304	0.7221042	0	0	1.116225012	0.751975233	*PPFIA1;CD47;CLASP2*
Biological Process	Response To Lipopolysaccharide (GO:0032496)	0.5117237	0.7221042	0	0	1.050530016	0.703824045	*FER;TRAF6;PLAA;BCL10;CXCL13;HNRNPA0*
Biological Process	Axonogenesis (GO:0007409)	0.5178833	0.7221042	0	0	1.03595422	0.681663448	*RELN;USP9X;SIAH1;CNTN4;SOS1;EPHB1;ZDHHC17*
Biological Process	Plasma Membrane Bounded Cell Projection Organization (GO:0120036)	0.5179424	0.7221042	0	0	1.054622543	0.693826925	*GSK3B;TBC1D23;CNTN4;STX3;PPP1R9A*
Biological Process	Regulation Of ERK1 And ERK2 Cascade (GO:0070372)	0.5181322	0.7221042	0	0	1.025855104	0.674525274	*MFAP3;ADIPOQ;SPRY2;SPRY1;OPRM1;EPHB1;ZDHHC17;RASGRP1;CRKL*
Biological Process	Macroautophagy (GO:0016236)	0.5182042	0.7221042	0	0	1.07150838	0.70439443	*VCP;EI24;PLAA;ATG14*

Biological Process	Regulation Of Intrinsic Apoptotic Signaling Pathway (GO:2001242)	0.519757	0.7221042	0	0	1.164708732	0.762178257	*DDX3X;SIAH1*
Biological Process	Negative Regulation Of ERK1 And ERK2 Cascade (GO:0070373)	0.519757	0.7221042	0	0	1.164708732	0.762178257	*SPRY2;SPRY1*
Biological Process	Post-Translational Protein Modification (GO:0043687)	0.519757	0.7221042	0	0	1.164708732	0.762178257	*PARP11;BAZ1B*
Biological Process	T Cell Mediated Immunity (GO:0002456)	0.5198387	0.7221042	0	0	1.409926067	0.922425299	*MICA*
Biological Process	Activation Of Adenylate Cyclase Activity (GO:0007190)	0.5198387	0.7221042	0	0	1.409926067	0.922425299	*RAF1*
Biological Process	Regulation Of Macrophage Chemotaxis (GO:0010758)	0.5198387	0.7221042	0	0	1.409926067	0.922425299	*MAPK1*
Biological Process	Regulation Of Necroptotic Process (GO:0060544)	0.5198387	0.7221042	0	0	1.409926067	0.922425299	*SPATA2*
Biological Process	Regulation Of Smooth Muscle Contraction (GO:0006940)	0.5198387	0.7221042	0	0	1.409926067	0.922425299	*ZDHHC21*
Biological Process	Regulation Of Toll-Like Receptor Signaling Pathway (GO:0034121)	0.5198387	0.7221042	0	0	1.409926067	0.922425299	*LGR4*
Biological Process	Response To Ketone (GO:1901654)	0.5198387	0.7221042	0	0	1.409926067	0.922425299	*UBE3A*
Biological Process	Sensory Perception Of Pain (GO:0019233)	0.5198387	0.7221042	0	0	1.409926067	0.922425299	*OPRM1*
Biological Process	Stress-Activated Protein Kinase Signaling Cascade (GO:0031098)	0.5198387	0.7221042	0	0	1.409926067	0.922425299	*MAPK1*
Biological Process	Striated Muscle Cell Differentiation (GO:0051146)	0.5198387	0.7221042	0	0	1.409926067	0.922425299	*MYEF2*
Biological Process	Glutamate Metabolic Process (GO:0006536)	0.5198387	0.7221042	0	0	1.409926067	0.922425299	*GLS*
Biological Process	Lipopolysaccharide-Mediated Signaling Pathway (GO:0031663)	0.5198387	0.7221042	0	0	1.409926067	0.922425299	*MTDH*
Biological Process	Long-Term Memory (GO:0007616)	0.5198387	0.7221042	0	0	1.409926067	0.922425299	*CPEB3*
Biological Process	Maturation Of LSU-rRNA (GO:0000470)	0.5198387	0.7221042	0	0	1.409926067	0.922425299	*RPF2*
Biological Process	Negative Regulation Of Cyclin-Dependent Protein Kinase Activity (GO:1904030)	0.5198387	0.7221042	0	0	1.409926067	0.922425299	*TFAP4*
Biological Process	Negative Regulation Of miRNA Transcription (GO:1902894)	0.5198387	0.7221042	0	0	1.409926067	0.922425299	*NFIB*
Biological Process	Negative Regulation Of Microtubule Depolymerization (GO:0007026)	0.5198387	0.7221042	0	0	1.409926067	0.922425299	*CLASP2*
Biological Process	Negative Regulation Of Phosphoprotein Phosphatase Activity (GO:0032515)	0.5198387	0.7221042	0	0	1.409926067	0.922425299	*GSK3B*
Biological Process	Negative Regulation Of Protein Localization To Cell Periphery (GO:1904376)	0.5198387	0.7221042	0	0	1.409926067	0.922425299	*PPFIA1*
Biological Process	Negative Regulation Of Protein Localization To Plasma Membrane (GO:1903077)	0.5198387	0.7221042	0	0	1.409926067	0.922425299	*PPFIA1*
Biological Process	Negative Regulation Of Viral Life Cycle (GO:1903901)	0.5198387	0.7221042	0	0	1.409926067	0.922425299	*MID2*
Biological Process	Nuclear-Transcribed mRNA poly(A) Tail Shortening (GO:0000289)	0.5198387	0.7221042	0	0	1.409926067	0.922425299	*CNOT6*
Biological Process	Phosphatidylglycerol Metabolic Process (GO:0046471)	0.5198387	0.7221042	0	0	1.409926067	0.922425299	*CDS2*
Biological Process	Positive Regulation Of Embryonic Development (GO:0040019)	0.5198387	0.7221042	0	0	1.409926067	0.922425299	*NR2C2*
Biological Process	Protein Deacylation (GO:0035601)	0.5198387	0.7221042	0	0	1.409926067	0.922425299	*SIRT5*
Biological Process	protein-DNA Complex Disassembly (GO:0032986)	0.5198387	0.7221042	0	0	1.409926067	0.922425299	*SUPT16H*
Biological Process	Pulmonary Valve Development (GO:0003177)	0.5198387	0.7221042	0	0	1.409926067	0.922425299	*HEY1*

Biological Process	Regulation Of Calcium Ion Import (GO:0090279)	0.5198387	0.7221042	0	0	1.409926067	0.922425299	*CTNNB1*
Biological Process	Regulation Of Cardiac Muscle Cell Proliferation (GO:0060043)	0.5198387	0.7221042	0	0	1.409926067	0.922425299	*VGLL4*
Biological Process	Regulation Of Cardiac Muscle Contraction By Calcium Ion Signaling (GO:0010882)	0.5198387	0.7221042	0	0	1.409926067	0.922425299	*CACNA1C*
Biological Process	Cellular Response To Organonitrogen Compound (GO:0071417)	0.5257639	0.7291877	0	0	1.060844073	0.682019919	*DHX8;RRAGD;CTNNB1;CPEB3*
Biological Process	Lysosomal Transport (GO:0007041)	0.5257639	0.7291877	0	0	1.060844073	0.682019919	*AP3M1;AP1G1;HOOK3;ATG14*
Biological Process	Positive Regulation Of Stress-Activated MAPK Cascade (GO:0032874)	0.5257639	0.7291877	0	0	1.060844073	0.682019919	*TRAF6;XIAP;RASGRP1;MAP3K4*
Biological Process	Cell Morphogenesis Involved In Neuron Differentiation (GO:0048667)	0.5275945	0.7298161	0	0	1.085943684	0.694382004	*MEF2A;CNTN4;ZDHHC17*
Biological Process	Embryonic Organ Development (GO:0048568)	0.5275945	0.7298161	0	0	1.085943684	0.694382004	*PBX2P1;HEY1;TBC1D23*
Biological Process	Sphingolipid Biosynthetic Process (GO:0030148)	0.5275945	0.7298161	0	0	1.085943684	0.694382004	*ELOVL4;FUT9;ST3GAL5*
Biological Process	Monoatomic Cation Homeostasis (GO:0055080)	0.5275945	0.7298161	0	0	1.085943684	0.694382004	*SLC11A2;XIAP;SLC12A6*
Biological Process	Positive Regulation Of JNK Cascade (GO:0046330)	0.5275945	0.7298161	0	0	1.085943684	0.694382004	*TRAF6;XIAP;RASGRP1*
Biological Process	Intracellular Monoatomic Cation Homeostasis (GO:0030003)	0.5291278	0.7315551	0	0	1.024525019	0.652136064	*ABCB7;SLC22A17;SLC30A5;SLC11A2;TMPRSS4;FBXL5;TPCN2*
Biological Process	Mitochondrion Organization (GO:0007005)	0.5300329	0.7323081	0	0	1.030165913	0.653966063	*MEF2A;CHCHD3;MTX3;SIRT5;PPARGC1A;SLC25A36*
Biological Process	Autophagy Of Mitochondrion (GO:0000422)	0.5308756	0.7323081	0	0	1.139868429	0.721796096	*WDR45B;ATG14*
Biological Process	Positive Regulation Of miRNA Metabolic Process (GO:2000630)	0.5308756	0.7323081	0	0	1.139868429	0.721796096	*POU2F1;STAT3*
Biological Process	Positive Regulation Of Nervous System Development (GO:0051962)	0.5308756	0.7323081	0	0	1.139868429	0.721796096	*OPRM1;EPHB1*
Biological Process	Positive Regulation Of Multicellular Organismal Process (GO:0051240)	0.5311004	0.7323081	0	0	1.005164465	0.636072335	*PRKAA1;AVPR1B;ADIPOQ;ARID4A;ARID4B;ARID1A;NR2C2;ING2;RBBP4;BCL7A;SIX4;ZNF703;CTNNB1;PPARGC1A*
Biological Process	Antigen Receptor-Mediated Signaling Pathway (GO:0050851)	0.5313311	0.7323081	0	0	1.038163387	0.656503229	*KHDRBS1;DENND1B;TRAF6;BCL10;SPPL3*
Biological Process	Positive Regulation Of Cell Growth (GO:0030307)	0.5332658	0.7323153	0	0	1.050388871	0.66041658	*SYT4;DDX3X;SMURF1;PLAA*
Biological Process	Nitrogen Compound Transport (GO:0071705)	0.5360731	0.7323153	0	0	1.023550822	0.63816836	*SLC22A5;KIF3A;MIA3;SCAMP1;LRP1B;SLC19A2*
Biological Process	Positive Regulation Of Tumor Necrosis Factor Production (GO:0032760)	0.5363414	0.7323153	0	0	1.071408647	0.667470949	*ARFGEF2;STAT3;RASGRP1*
Biological Process	DNA Replication Initiation (GO:0006270)	0.5371418	0.7323153	0	0	1.339360223	0.832403219	*ORC1*
Biological Process	Regulation Of Dendritic Spine Development (GO:0060998)	0.5371418	0.7323153	0	0	1.339360223	0.832403219	*CPEB3*
Biological Process	Regulation Of Neurotransmitter Transport (GO:0051588)	0.5371418	0.7323153	0	0	1.339360223	0.832403219	*CASK*
Biological Process	Regulation Of Transferase Activity (GO:0051338)	0.5371418	0.7323153	0	0	1.339360223	0.832403219	*FEM1B*
Biological Process	Response To Cadmium Ion (GO:0046686)	0.5371418	0.7323153	0	0	1.339360223	0.832403219	*MAPK1*
Biological Process	Endocardial Cushion Morphogenesis (GO:0003203)	0.5371418	0.7323153	0	0	1.339360223	0.832403219	*HEY1*
Biological Process	Sulfur Compound Transport (GO:0072348)	0.5371418	0.7323153	0	0	1.339360223	0.832403219	*SLC19A2*
Biological Process	Zinc Ion Transmembrane Transport (GO:0071577)	0.5371418	0.7323153	0	0	1.339360223	0.832403219	*SLC30A5*

Biological Process	Modulation By Host Of Viral Genome Replication (GO:0044827)	0.5371418	0.7323153	0	0	1.339360223	0.832403219	*SMC6*
Biological Process	Negative Regulation Of Biomineral Tissue Development (GO:0070168)	0.5371418	0.7323153	0	0	1.339360223	0.832403219	*HEY1*
Biological Process	Negative Regulation Of miRNA Metabolic Process (GO:2000629)	0.5371418	0.7323153	0	0	1.339360223	0.832403219	*NFIB*
Biological Process	Negative Regulation Of Tumor Necrosis Factor-Mediated Signaling Pathway (GO:0010804)	0.5371418	0.7323153	0	0	1.339360223	0.832403219	*XIAP*
Biological Process	Positive Regulation Of Amyloid Precursor Protein Catabolic Process (GO:1902993)	0.5371418	0.7323153	0	0	1.339360223	0.832403219	*CASP3*
Biological Process	Positive Regulation Of Natural Killer Cell Mediated Cytotoxicity (GO:0045954)	0.5371418	0.7323153	0	0	1.339360223	0.832403219	*RASGRP1*
Biological Process	Positive Regulation Of Vacuole Organization (GO:0044090)	0.5371418	0.7323153	0	0	1.339360223	0.832403219	*RAB3GAP2*
Biological Process	Protein Mannosylation (GO:0035268)	0.5371418	0.7323153	0	0	1.339360223	0.832403219	*TMTC3*
Biological Process	Regulation Of Cardiac Conduction (GO:1903779)	0.5371418	0.7323153	0	0	1.339360223	0.832403219	*ATP2B2*
Biological Process	Regulation Of Cell-Substrate Junction Assembly (GO:0090109)	0.5371418	0.7323153	0	0	1.339360223	0.832403219	*PEAK1*
Biological Process	Cellular Response To Metal Ion (GO:0071248)	0.5379628	0.7326804	0	0	1.030123722	0.638641582	*MEF2A;SYT4;PRKAA1;PRKAA2;MAPK1*
Biological Process	Non-Membrane-Bounded Organelle Assembly (GO:0140694)	0.5379628	0.7326804	0	0	1.030123722	0.638641582	*YTHDF1;DDX3X;YTHDF3;CIRBP;CDS2*
Biological Process	G Protein-Coupled Receptor Signaling Pathway, Coupled To Cyclic Nucleotide Second Messenger (GO:0007187)	0.5418174	0.7356608	0	0	1.116063138	0.683952845	*DHX8;OPRM1*
Biological Process	Dicarboxylic Acid Metabolic Process (GO:0043648)	0.5418174	0.7356608	0	0	1.116063138	0.683952845	*SLC25A32;L2HGDH*
Biological Process	Mitotic Metaphase Plate Congression (GO:0007080)	0.5418174	0.7356608	0	0	1.116063138	0.683952845	*PDCD6IP;CUL3*
Biological Process	Nucleocytoplasmic Transport (GO:0006913)	0.5418174	0.7356608	0	0	1.116063138	0.683952845	*ATXN1;NUP153*
Biological Process	Positive Regulation Of Fat Cell Differentiation (GO:0045600)	0.5418174	0.7356608	0	0	1.116063138	0.683952845	*CREB1;HNRNPU*
Biological Process	Positive Regulation Of Transport (GO:0051050)	0.5418174	0.7356608	0	0	1.116063138	0.683952845	*GSK3B;PLAA*
Biological Process	Regulation Of Cyclin-Dependent Protein Serine/Threonine Kinase Activity (GO:0000079)	0.5449953	0.7372236	0	0	1.057256111	0.641731335	*CCNT2;TFAP4;CCNG2*
Biological Process	Positive Regulation Of Protein Localization To Membrane (GO:1905477)	0.5449953	0.7372236	0	0	1.057256111	0.641731335	*PDPK1;STX3;SORBS1*
Biological Process	Glycerophospholipid Biosynthetic Process (GO:0046474)	0.54809	0.7372236	0	0	1.03008165	0.619404388	*ETNK1;GPAT3;LCLAT1;CDS2*
Biological Process	Regulation Of Substrate Adhesion-Dependent Cell Spreading (GO:1900024)	0.5525806	0.7372236	0	0	1.093229492	0.648455614	*ARHGEF7;CRKL*
Biological Process	Regulation Of Synapse Assembly (GO:0051963)	0.5525806	0.7372236	0	0	1.093229492	0.648455614	*SETD5;EPHB1*
Biological Process	Metaphase Plate Congression (GO:0051310)	0.5525806	0.7372236	0	0	1.093229492	0.648455614	*PDCD6IP;CUL3*
Biological Process	Positive Regulation Of Ras Protein Signal Transduction (GO:0046579)	0.5525806	0.7372236	0	0	1.093229492	0.648455614	*PIK3CB;RASGRP1*
Biological Process	Positive Regulation Of Leukocyte Mediated Cytotoxicity (GO:0001912)	0.5525806	0.7372236	0	0	1.093229492	0.648455614	*RASGRP1;MICA*
Biological Process	Response To Type II Interferon (GO:0034341)	0.5535544	0.7372236	0	0	1.043471173	0.617103817	*NUB1;SLC22A5;CD47*

Biological Process	Glycoprotein Biosynthetic Process (GO:0009101)	0.5535544	0.7372236	0	0	1.043471173	0.617103817	*FUT9;ST3GAL5;ALG10B*
Biological Process	Wound Healing (GO:0042060)	0.5535544	0.7372236	0	0	1.043471173	0.617103817	*EPB41L4B;MIA3;RAF1*
Biological Process	Regulation Of Dopamine Secretion (GO:0014059)	0.5538222	0.7372236	0	0	1.275514935	0.753716627	*SYT4*
Biological Process	NAD Metabolic Process (GO:0019674)	0.5538222	0.7372236	0	0	1.275514935	0.753716627	*VCP*
Biological Process	Acylglycerol Biosynthetic Process (GO:0046463)	0.5538222	0.7372236	0	0	1.275514935	0.753716627	*GPAT3*
Biological Process	Regulation Of Mononuclear Cell Migration (GO:0071675)	0.5538222	0.7372236	0	0	1.275514935	0.753716627	*MAPK1*
Biological Process	Regulation Of Neural Precursor Cell Proliferation (GO:2000177)	0.5538222	0.7372236	0	0	1.275514935	0.753716627	*KDM2B*
Biological Process	Blood Vessel Endothelial Cell Migration (GO:0043534)	0.5538222	0.7372236	0	0	1.275514935	0.753716627	*MIA3*
Biological Process	Regulation Of Protein Export From Nucleus (GO:0046825)	0.5538222	0.7372236	0	0	1.275514935	0.753716627	*GSK3B*
Biological Process	Regulation Of Release Of Sequestered Calcium Ion Into Cytosol By Sarcoplasmic Reticulum (GO:0010880)	0.5538222	0.7372236	0	0	1.275514935	0.753716627	*CACNA1C*
Biological Process	Cellular Response To Cadmium Ion (GO:0071276)	0.5538222	0.7372236	0	0	1.275514935	0.753716627	*MAPK1*
Biological Process	Cellular Response To Low-Density Lipoprotein Particle Stimulus (GO:0071404)	0.5538222	0.7372236	0	0	1.275514935	0.753716627	*MIA3*
Biological Process	Cellular Response To Topologically Incorrect Protein (GO:0035967)	0.5538222	0.7372236	0	0	1.275514935	0.753716627	*UBE2W*
Biological Process	Response To Gamma Radiation (GO:0010332)	0.5538222	0.7372236	0	0	1.275514935	0.753716627	*PRKAA1*
Biological Process	Cytoskeleton-Dependent Intracellular Transport (GO:0030705)	0.5538222	0.7372236	0	0	1.275514935	0.753716627	*HOOK3*
Biological Process	Mitochondrial Cytochrome C Oxidase Assembly (GO:0033617)	0.5538222	0.7372236	0	0	1.275514935	0.753716627	*COA5*
Biological Process	Natural Killer Cell Mediated Immunity (GO:0002228)	0.5538222	0.7372236	0	0	1.275514935	0.753716627	*MICA*
Biological Process	Negative Regulation Of G Protein-Coupled Receptor Signaling Pathway (GO:0045744)	0.5538222	0.7372236	0	0	1.275514935	0.753716627	*RPGRIP1L*
Biological Process	Negative Regulation Of Extrinsic Apoptotic Signaling Pathway In Absence Of Ligand (GO:2001240)	0.5538222	0.7372236	0	0	1.275514935	0.753716627	*EYA3*
Biological Process	Negative Regulation Of Protein Localization To Nucleus (GO:1900181)	0.5538222	0.7372236	0	0	1.275514935	0.753716627	*GSK3B*
Biological Process	Negative Regulation Of Signal Transduction In Absence Of Ligand (GO:1901099)	0.5538222	0.7372236	0	0	1.275514935	0.753716627	*EYA3*
Biological Process	Oligosaccharide Biosynthetic Process (GO:0009312)	0.5538222	0.7372236	0	0	1.275514935	0.753716627	*FUT9*
Biological Process	Positive Regulation Of Cholesterol Efflux (GO:0010875)	0.5538222	0.7372236	0	0	1.275514935	0.753716627	*ADIPOQ*
Biological Process	Positive Regulation Of Excitatory Postsynaptic Potential (GO:2000463)	0.5538222	0.7372236	0	0	1.275514935	0.753716627	*RELN*
Biological Process	Positive Regulation Of Phosphatase Activity (GO:0010922)	0.5538222	0.7372236	0	0	1.275514935	0.753716627	*MTMR9*
Biological Process	Positive Regulation Of Protein Autophosphorylation (GO:0031954)	0.5538222	0.7372236	0	0	1.275514935	0.753716627	*DDX3X*
Biological Process	Positive Regulation Of Synaptic Transmission, Glutamatergic (GO:0051968)	0.5538222	0.7372236	0	0	1.275514935	0.753716627	*RELN*
Biological Process	Postreplication Repair (GO:0006301)	0.5538222	0.7372236	0	0	1.275514935	0.753716627	*VCP*

Biological Process	Protein Neddylation (GO:0045116)	0.5538222	0.7372236	0	0	1.275514935	0.753716627	*DCUN1D5*
Biological Process	rRNA Methylation (GO:0031167)	0.5538222	0.7372236	0	0	1.275514935	0.753716627	*METTL15*
Biological Process	Cell-Matrix Adhesion (GO:0007160)	0.5554092	0.7385939	0	0	1.020218143	0.59993937	*PEAK1;PPFIA1;SORBS1;ARHGEF7*
Biological Process	Cellular Response To Salt (GO:1902075)	0.5554092	0.7385939	0	0	1.020218143	0.59993937	*MEF2A;SYT4;PRKAA1;PRKAA2*
Biological Process	Cellular Response To Interleukin-1 (GO:0071347)	0.5620171	0.7451655	0	0	1.030039695	0.593532603	*TRAF6;CD47;RBMX*
Biological Process	Response To Lipid (GO:0033993)	0.5626647	0.7451655	0	0	1.01054074	0.581133053	*FER;STAT3;CTNNB1;HNRNPA0*
Biological Process	Regulation Of JNK Cascade (GO:0046328)	0.5626647	0.7451655	0	0	1.01054074	0.581133053	*TRAF6;XIAP;EPHB1;RASGRP1*
Biological Process	Rho Protein Signal Transduction (GO:0007266)	0.563164	0.7451655	0	0	1.071309192	0.615129107	*ARHGAP29;ARHGAP5*
Biological Process	Adenylate Cyclase-Inhibiting G Protein-Coupled Receptor Signaling Pathway (GO:0007193)	0.563164	0.7451655	0	0	1.071309192	0.615129107	*GNAI3;OPRM1*
Biological Process	Regulation Of Peptide Hormone Secretion (GO:0090276)	0.563164	0.7451655	0	0	1.071309192	0.615129107	*UQCC2;CLOCK*
Biological Process	Cartilage Development (GO:0051216)	0.563164	0.7451655	0	0	1.071309192	0.615129107	*OSR2;NFIB*
Biological Process	Regulation Of Proteolysis (GO:0030162)	0.563164	0.7451655	0	0	1.071309192	0.615129107	*FBXW11;BTRC*
Biological Process	Fatty Acid Oxidation (GO:0019395)	0.563164	0.7451655	0	0	1.071309192	0.615129107	*ADIPOQ;PPARGC1A*
Biological Process	Negative Regulation Of Response To Stimulus (GO:0048585)	0.563164	0.7451655	0	0	1.071309192	0.615129107	*RTEL1;CASK*
Biological Process	Cytoskeleton Organization (GO:0007010)	0.5698551	0.7455165	0	0	1.001044223	0.562960395	*ZMYM3;PHIP;BRWD3;WASF3*
Biological Process	Mitotic Sister Chromatid Segregation (GO:0000070)	0.5698551	0.7455165	0	0	1.001044223	0.562960395	*PDCD6IP;CUL3;TTN;CLASP2*
Biological Process	Regulation Of Centrosome Cycle (GO:0046605)	0.5699022	0.7455165	0	0	1.217473764	0.68457392	*PDCD6IP*
Biological Process	Actin Nucleation (GO:0045010)	0.5699022	0.7455165	0	0	1.217473764	0.68457392	*JMY*
Biological Process	Adaptive Immune Response Based On Somatic Recombination Of Immune Receptors Built From Immunoglobulin Superfamily Domains (GO:0002460)	0.5699022	0.7455165	0	0	1.217473764	0.68457392	*STAT3*
Biological Process	Axon Extension (GO:0048675)	0.5699022	0.7455165	0	0	1.217473764	0.68457392	*USP9X*
Biological Process	Regulation Of Toll-Like Receptor 4 Signaling Pathway (GO:0034143)	0.5699022	0.7455165	0	0	1.217473764	0.68457392	*APPL1*
Biological Process	Rescue Of Stalled Ribosome (GO:0072344)	0.5699022	0.7455165	0	0	1.217473764	0.68457392	*PELO*
Biological Process	Endocardial Cushion Development (GO:0003197)	0.5699022	0.7455165	0	0	1.217473764	0.68457392	*HEY1*
Biological Process	Entrainment Of Circadian Clock By Photoperiod (GO:0043153)	0.5699022	0.7455165	0	0	1.217473764	0.68457392	*FBXL3*
Biological Process	Translesion Synthesis (GO:0019985)	0.5699022	0.7455165	0	0	1.217473764	0.68457392	*VCP*
Biological Process	Heparan Sulfate Proteoglycan Biosynthetic Process (GO:0015012)	0.5699022	0.7455165	0	0	1.217473764	0.68457392	*GLCE*
Biological Process	Zinc Ion Transport (GO:0006829)	0.5699022	0.7455165	0	0	1.217473764	0.68457392	*SLC30A5*
Biological Process	Microtubule Nucleation (GO:0007020)	0.5699022	0.7455165	0	0	1.217473764	0.68457392	*CLASP2*
Biological Process	Negative Regulation Of Lymphocyte Activation (GO:0051250)	0.5699022	0.7455165	0	0	1.217473764	0.68457392	*MICA*
Biological Process	Negative Regulation Of Protein Depolymerization (GO:1901880)	0.5699022	0.7455165	0	0	1.217473764	0.68457392	*CLASP2*
Biological Process	Negative Regulation Of Protein Tyrosine Kinase Activity (GO:0061099)	0.5699022	0.7455165	0	0	1.217473764	0.68457392	*CBL*

Biological Process	Positive Regulation Of Morphogenesis Of An Epithelium (GO:1905332)	0.5699022	0.7455165	0	0	1.217473764	0.68457392	*SIX4*
Biological Process	Positive Regulation Of Neuron Apoptotic Process (GO:0043525)	0.5699022	0.7455165	0	0	1.217473764	0.68457392	*CTNNB1*
Biological Process	Potassium Ion Homeostasis (GO:0055075)	0.5699022	0.7455165	0	0	1.217473764	0.68457392	*SLC12A6*
Biological Process	Protein Localization To Cell Junction (GO:1902414)	0.5699022	0.7455165	0	0	1.217473764	0.68457392	*KIF3A*
Biological Process	Regulation Of Cardiac Muscle Hypertrophy (GO:0010611)	0.5699022	0.7455165	0	0	1.217473764	0.68457392	*MEF2A*
Biological Process	Regulation Of Cell Junction Assembly (GO:1901888)	0.5699022	0.7455165	0	0	1.217473764	0.68457392	*SETD5*
Biological Process	Positive Regulation Of Cell Population Proliferation (GO:0008284)	0.5731194	0.7477251	0	0	0.976312432	0.543475185	*SLC35F6;OSR2;EGR4;CUL3;HNRNPU;CRKL;CNOT6;EPGN;FER;BCL7A;ZNF703;SSR1;PHIP;STX3;CD47;ZNF268;SOX4*
Biological Process	Regulation Of Cyclin-Dependent Protein Kinase Activity (GO:1904029)	0.5735664	0.7477251	0	0	1.050248512	0.583813736	*CCNT2;CCNG2*
Biological Process	NADH Dehydrogenase Complex Assembly (GO:0010257)	0.5735664	0.7477251	0	0	1.050248512	0.583813736	*NDUFAF7;NDUFC2*
Biological Process	Regulation Of Macromolecule Biosynthetic Process (GO:0010556)	0.5735664	0.7477251	0	0	1.050248512	0.583813736	*NOLC1;MSI2*
Biological Process	Mitochondrial Respiratory Chain Complex I Assembly (GO:0032981)	0.5735664	0.7477251	0	0	1.050248512	0.583813736	*NDUFAF7;NDUFC2*
Biological Process	Positive Regulation Of Chemokine Production (GO:0032722)	0.5735664	0.7477251	0	0	1.050248512	0.583813736	*DDX3X;ADIPOQ*
Biological Process	Protein Homotetramerization (GO:0051289)	0.5735664	0.7477251	0	0	1.050248512	0.583813736	*TNPO1;GLS*
Biological Process	Regulation Of Cell Growth (GO:0001558)	0.5746172	0.7487265	0	0	0.977938536	0.541828044	*ST7L;DDX3X;DCUN1D5;SERTAD2;GNG4;EI24;SPOCK1;VGLL4*
Biological Process	Calcium Ion Transport (GO:0006816)	0.5769792	0.7498713	0	0	0.991723567	0.545397457	*ORAI3;ORAI2;ATP2B2;CACNA1C*
Biological Process	Negative Regulation Of Defense Response (GO:0031348)	0.5769792	0.7498713	0	0	0.991723567	0.545397457	*PPP6C;PRKDC;ADIPOQ;STAT3*
Biological Process	Anterograde Trans-Synaptic Signaling (GO:0098916)	0.5783704	0.7498713	0	0	0.976040206	0.534421894	*ATXN3;CACNB4;DHX8;KCND2;MAPK1;SLC12A6;LRP6*
Biological Process	Calcium Ion Transmembrane Import Into Cytosol (GO:0097553)	0.5786467	0.7498713	0	0	1.0041841	0.549352205	*PLCB4;PLCH1;CACNA1C*
Biological Process	Vascular Transport (GO:0010232)	0.5786467	0.7498713	0	0	1.0041841	0.549352205	*SLC22A5;AVPR1B;SLC4A4*
Biological Process	Regulation Of Cell Division (GO:0051302)	0.5786467	0.7498713	0	0	1.0041841	0.549352205	*DR1;WDR5;MLLT3*
Biological Process	Cellular Response To Lipid (GO:0071396)	0.5796171	0.7498713	0	0	0.973442288	0.53090327	*GSK3B;CREB1;TRAF6;ZNF703;PLAA;LCOR;BCL10;CXCL13*
Biological Process	Heart Development (GO:0007507)	0.5831233	0.7498713	0	0	0.973516679	0.525072653	*MEF2A;NCOA6;PHIP;CACNA1C;MEF2D;SOX4*
Biological Process	Regulation Of Exocytosis (GO:0017157)	0.5837873	0.7498713	0	0	1.029997857	0.554363963	*TPCN2;CLASP2*
Biological Process	Regulation Of Focal Adhesion Assembly (GO:0051893)	0.5837873	0.7498713	0	0	1.029997857	0.554363963	*PEAK1;CLASP2*
Biological Process	Ribosomal Large Subunit Biogenesis (GO:0042273)	0.5837873	0.7498713	0	0	1.029997857	0.554363963	*RPF2;MDN1*
Biological Process	Epidermal Cell Differentiation (GO:0009913)	0.5837873	0.7498713	0	0	1.029997857	0.554363963	*CASP3;CTNNB1*
Biological Process	Regulation Of Endoplasmic Reticulum Stress-Induced Intrinsic Apoptotic Signaling Pathway (GO:1902235)	0.5854035	0.7498713	0	0	1.164479652	0.623525171	*NCK1*
Biological Process	Regulation Of Execution Phase Of Apoptosis (GO:1900117)	0.5854035	0.7498713	0	0	1.164479652	0.623525171	*FZD3*
Biological Process	Regulation Of Microtubule Depolymerization (GO:0031114)	0.5854035	0.7498713	0	0	1.164479652	0.623525171	*CLASP2*

Biological Process	Cell Morphogenesis Involved In Differentiation (GO:0000904)	0.5854035	0.7498713	0	0	1.164479652	0.623525171	*RELN*
Biological Process	Regulation Of Viral Life Cycle (GO:1903900)	0.5854035	0.7498713	0	0	1.164479652	0.623525171	*CD209*
Biological Process	Response To Purine-Containing Compound (GO:0014074)	0.5854035	0.7498713	0	0	1.164479652	0.623525171	*CREB1*
Biological Process	Dicarboxylic Acid Transport (GO:0006835)	0.5854035	0.7498713	0	0	1.164479652	0.623525171	*SLC25A32*
Biological Process	Secondary Alcohol Biosynthetic Process (GO:1902653)	0.5854035	0.7498713	0	0	1.164479652	0.623525171	*HMGCR*
Biological Process	Ear Morphogenesis (GO:0042471)	0.5854035	0.7498713	0	0	1.164479652	0.623525171	*OSR2*
Biological Process	Endosome Transport Via Multivesicular Body Sorting Pathway (GO:0032509)	0.5854035	0.7498713	0	0	1.164479652	0.623525171	*VCP*
Biological Process	Fat-Soluble Vitamin Metabolic Process (GO:0006775)	0.5854035	0.7498713	0	0	1.164479652	0.623525171	*ARID4A*
Biological Process	Iron Ion Transport (GO:0006826)	0.5854035	0.7498713	0	0	1.164479652	0.623525171	*SLC11A2*
Biological Process	Natural Killer Cell Mediated Cytotoxicity (GO:0042267)	0.5854035	0.7498713	0	0	1.164479652	0.623525171	*MICA*
Biological Process	Negative Regulation Of Cell Junction Assembly (GO:1901889)	0.5854035	0.7498713	0	0	1.164479652	0.623525171	*CLASP2*
Biological Process	Neuromuscular Junction Development (GO:0007528)	0.5854035	0.7498713	0	0	1.164479652	0.623525171	*CACNB4*
Biological Process	Peroxisome Organization (GO:0007031)	0.5854035	0.7498713	0	0	1.164479652	0.623525171	*PEX3*
Biological Process	Positive Regulation Of ERBB Signaling Pathway (GO:1901186)	0.5854035	0.7498713	0	0	1.164479652	0.623525171	*CBL*
Biological Process	Positive Regulation Of Natural Killer Cell Mediated Immunity (GO:0002717)	0.5854035	0.7498713	0	0	1.164479652	0.623525171	*RASGRP1*
Biological Process	Positive Regulation Of p38MAPK Cascade (GO:1900745)	0.5854035	0.7498713	0	0	1.164479652	0.623525171	*MAP3K4*
Biological Process	Positive Regulation Of Peptidyl-Threonine Phosphorylation (GO:0010800)	0.5854035	0.7498713	0	0	1.164479652	0.623525171	*MAPK1*
Biological Process	Protein Kinase B Signaling (GO:0043491)	0.5854035	0.7498713	0	0	1.164479652	0.623525171	*THEM4*
Biological Process	Regulation Of AMPA Receptor Activity (GO:2000311)	0.5854035	0.7498713	0	0	1.164479652	0.623525171	*RELN*
Biological Process	Regulation Of Actin Nucleation (GO:0051125)	0.5854035	0.7498713	0	0	1.164479652	0.623525171	*ARPIN*
Biological Process	Ribosomal Small Subunit Biogenesis (GO:0042274)	0.5868109	0.7513108	0	0	0.99173511	0.528647051	*MRPS11;RIOK2;DDX52*
Biological Process	Supramolecular Fiber Organization (GO:0097435)	0.5883042	0.7528588	0	0	0.965224629	0.512062435	*MARCKS;DNAJB6;USP9X;MYO15A;KRT37;SIAH1;MYO5A;P3H4;HOOK3;TARDBP;PPP1R9A*
Biological Process	Organic Substance Transport (GO:0071702)	0.5889756	0.7533541	0	0	0.965876722	0.511306628	*SLC22A5;KIF3A;MIA3;ATP2B2;SCAMP1;TNPO1;LRP1B*
Biological Process	Regulation Of Cytoskeleton Organization (GO:0051493)	0.5910239	0.7552447	0	0	0.973590655	0.512010168	*GSK3B;PRKAA1;PRKAA2;MAPK1*
Biological Process	Response To Endoplasmic Reticulum Stress (GO:0034976)	0.5910239	0.7552447	0	0	0.973590655	0.512010168	*GSK3B;VCP;TMTC3;NCK1*
Biological Process	Response To Peptide Hormone (GO:0043434)	0.5938262	0.7565742	0	0	1.010511379	0.526646846	*CREB1;STAT3*
Biological Process	Positive Regulation Of Phosphorylation (GO:0042327)	0.5944505	0.7565742	0	0	0.960195496	0.499414793	*RELN;ADIPOQ;BCL10;ATG14;FNIP1;RASGRP1;CRKL;CAMKK2*
Biological Process	Regulation Of Kinase Activity (GO:0043549)	0.5948732	0.7565742	0	0	0.979589754	0.508805856	*HNRNPU;CAMKK2;TTN*
Biological Process	Response To Interleukin-1 (GO:0070555)	0.5948732	0.7565742	0	0	0.979589754	0.508805856	*TRAF6;CD47;RBMX*
Biological Process	Transport Across Blood-Brain Barrier (GO:0150104)	0.5948732	0.7565742	0	0	0.979589754	0.508805856	*SLC22A5;AVPR1B;SLC4A4*
Biological Process	Mitotic Spindle Organization (GO:0007052)	0.5948732	0.7565742	0	0	0.979589754	0.508805856	*VCP;CENPH;CLASP2*
Biological Process	Cellular Component Assembly (GO:0022607)	0.5950016	0.7565742	0	0	0.959655051	0.498244501	*TTC26;YTHDF1;TFAP4;RTN1;YTHDF3;REPS2;P3H4;PPARGC1A;WASF3*

Biological Process	Regulation Of Intracellular Estrogen Receptor Signaling Pathway (GO:0033146)	0.6003469	0.7565742	0	0	1.115901715	0.569386244	*UFSP2*
Biological Process	Activation Of Protein Kinase B Activity (GO:0032148)	0.6003469	0.7565742	0	0	1.115901715	0.569386244	*PDPK1*
Biological Process	Carbohydrate Biosynthetic Process (GO:0016051)	0.6003469	0.7565742	0	0	1.115901715	0.569386244	*FUT9*
Biological Process	Regulation Of Synapse Organization (GO:0050807)	0.6003469	0.7565742	0	0	1.115901715	0.569386244	*SETD5*
Biological Process	Chemical Synaptic Transmission, Postsynaptic (GO:0099565)	0.6003469	0.7565742	0	0	1.115901715	0.569386244	*GSK3B*
Biological Process	Cholesterol Biosynthetic Process (GO:0006695)	0.6003469	0.7565742	0	0	1.115901715	0.569386244	*HMGCR*
Biological Process	Sodium Ion Homeostasis (GO:0055078)	0.6003469	0.7565742	0	0	1.115901715	0.569386244	*TMPRSS4*
Biological Process	Lamellipodium Assembly (GO:0030032)	0.6003469	0.7565742	0	0	1.115901715	0.569386244	*ARHGEF7*
Biological Process	Mismatch Repair (GO:0006298)	0.6003469	0.7565742	0	0	1.115901715	0.569386244	*PMS1*
Biological Process	Negative Regulation Of TORC1 Signaling (GO:1904262)	0.6003469	0.7565742	0	0	1.115901715	0.569386244	*BMT2*
Biological Process	Negative Regulation Of Intrinsic Apoptotic Signaling Pathway In Response To DNA Damage (GO:1902230)	0.6003469	0.7565742	0	0	1.115901715	0.569386244	*ING2*
Biological Process	Negative Regulation Of Protein Localization To Membrane (GO:1905476)	0.6003469	0.7565742	0	0	1.115901715	0.569386244	*PPFIA1*
Biological Process	Negative Regulation Of Protein Transport (GO:0051224)	0.6003469	0.7565742	0	0	1.115901715	0.569386244	*HMGCR*
Biological Process	Negative Regulation Of Signaling Receptor Activity (GO:2000272)	0.6003469	0.7565742	0	0	1.115901715	0.569386244	*CBL*
Biological Process	Negative Regulation Of Smoothened Signaling Pathway (GO:0045879)	0.6003469	0.7565742	0	0	1.115901715	0.569386244	*VCP*
Biological Process	Phosphatidylethanolamine Metabolic Process (GO:0046337)	0.6003469	0.7565742	0	0	1.115901715	0.569386244	*ETNK1*
Biological Process	Positive Regulation Of T Cell Migration (GO:2000406)	0.6003469	0.7565742	0	0	1.115901715	0.569386244	*CXCL13*
Biological Process	Positive Regulation Of Interleukin-2 Production (GO:0032743)	0.6003469	0.7565742	0	0	1.115901715	0.569386244	*TRAF6*
Biological Process	Positive Regulation Of Peptidase Activity (GO:0010952)	0.6003469	0.7565742	0	0	1.115901715	0.569386244	*VCP*
Biological Process	Regulation Of Biomineral Tissue Development (GO:0070167)	0.6003469	0.7565742	0	0	1.115901715	0.569386244	*HEY1*
Biological Process	Regulation Of Cell Cycle Phase Transition (GO:1901987)	0.6003469	0.7565742	0	0	1.115901715	0.569386244	*TFAP4*
Biological Process	Regulation Of Cell Size (GO:0008361)	0.6003469	0.7565742	0	0	1.115901715	0.569386244	*ARHGAP5*
Biological Process	Exocytosis (GO:0006887)	0.6028324	0.7589844	0	0	0.967737057	0.489787226	*MIA3;EXOC6;STX3*
Biological Process	Negative Regulation Of Binding (GO:0051100)	0.6028324	0.7589844	0	0	0.967737057	0.489787226	*GSK3B;TFAP4;GEMIN2*
Biological Process	Regulation Of Mitotic Nuclear Division (GO:0007088)	0.6036829	0.7593333	0	0	0.991746621	0.500540761	*EPGN;PHIP*
Biological Process	Nucleotide-Excision Repair (GO:0006289)	0.6036829	0.7593333	0	0	0.991746621	0.500540761	*ERCC4;RNF111*
Biological Process	Regulation Of MAPK Cascade (GO:0043408)	0.6046303	0.7601641	0	0	0.951018432	0.478493565	*DUSP5;AVPR1B;MAPK1;RAF1;EPHB1;ZDHHC17;RASGRP1*
Biological Process	Negative Regulation Of Response To External Stimulus (GO:0032102)	0.6078018	0.7616833	0	0	0.949213907	0.472619681	*PPP6C;PRKDC;ADIPOQ;STAT3;CASK*
Biological Process	Neuron Projection Morphogenesis (GO:0048812)	0.6078018	0.7616833	0	0	0.949213907	0.472619681	*MEF2A;CTNNB1;CNTN4;EPHB1;ZDHHC17*
Biological Process	Regulation Of Insulin Secretion (GO:0050796)	0.6106878	0.7616833	0	0	0.956166567	0.471552088	*UQCC2;CLOCK;SOX4*
Biological Process	Cellular Response To Molecule Of Bacterial Origin (GO:0071219)	0.6115673	0.7616833	0	0	0.947594799	0.465961088	*TRAF6;PLAA;BCL10;CXCL13*

Biological Process	Positive Regulation Of Protein Serine/Threonine Kinase Activity (GO:0071902)	0.6115673	0.7616833	0	0	0.947594799	0.465961088	*EPGN;DDX3X;ADIPOQ;RASGRP1*
Biological Process	Protein Maturation (GO:0051604)	0.6115673	0.7616833	0	0	0.947594799	0.465961088	*CASP3;TMPRSS4;TSPAN5;NAA15*
Biological Process	RNA Catabolic Process (GO:0006401)	0.6133575	0.7616833	0	0	0.973664219	0.47593427	*DIS3;DCP2*
Biological Process	Negative Regulation Of Protein Metabolic Process (GO:0051248)	0.6138434	0.7616833	0	0	0.942480055	0.459944866	*DDX3X;DHX36;HMGCR;CPEB3;EIF4E2*
Biological Process	Protein Glycosylation (GO:0006486)	0.6138434	0.7616833	0	0	0.942480055	0.459944866	*POGLUT1;FUT9;GFPT1;ST3GAL5;ALG10B*
Biological Process	cAMP-mediated Signaling (GO:0019933)	0.6147523	0.7616833	0	0	1.071210014	0.521181995	*CREB1*
Biological Process	Calcium-Ion Regulated Exocytosis (GO:0017156)	0.6147523	0.7616833	0	0	1.071210014	0.521181995	*SYT4*
Biological Process	Camera-Type Eye Development (GO:0043010)	0.6147523	0.7616833	0	0	1.071210014	0.521181995	*CACNA1C*
Biological Process	Cardiac Muscle Cell Development (GO:0055013)	0.6147523	0.7616833	0	0	1.071210014	0.521181995	*TTN*
Biological Process	Cell-Cell Adhesion Mediated By Cadherin (GO:0044331)	0.6147523	0.7616833	0	0	1.071210014	0.521181995	*FER*
Biological Process	Cellular Response To Xenobiotic Stimulus (GO:0071466)	0.6147523	0.7616833	0	0	1.071210014	0.521181995	*ADIPOQ*
Biological Process	Respiratory Chain Complex IV Assembly (GO:0008535)	0.6147523	0.7616833	0	0	1.071210014	0.521181995	*COA5*
Biological Process	Response To Glucocorticoid (GO:0051384)	0.6147523	0.7616833	0	0	1.071210014	0.521181995	*HNRNPU*
Biological Process	Dendritic Spine Organization (GO:0097061)	0.6147523	0.7616833	0	0	1.071210014	0.521181995	*EPHB1*
Biological Process	Establishment Or Maintenance Of Apical/Basal Cell Polarity (GO:0035088)	0.6147523	0.7616833	0	0	1.071210014	0.521181995	*PARD6G*
Biological Process	Telomere Maintenance Via Telomere Lengthening (GO:0010833)	0.6147523	0.7616833	0	0	1.071210014	0.521181995	*TERF2IP*
Biological Process	Negative Regulation Of Catalytic Activity (GO:0043086)	0.6147523	0.7616833	0	0	1.071210014	0.521181995	*CAND1*
Biological Process	Negative Regulation Of Endothelial Cell Apoptotic Process (GO:2000352)	0.6147523	0.7616833	0	0	1.071210014	0.521181995	*PDPK1*
Biological Process	Negative Regulation Of Insulin Receptor Signaling Pathway (GO:0046627)	0.6147523	0.7616833	0	0	1.071210014	0.521181995	*NCK1*
Biological Process	Negative Regulation Of Protein Polymerization (GO:0032272)	0.6147523	0.7616833	0	0	1.071210014	0.521181995	*DYRK1A*
Biological Process	Negative Regulation Of Receptor-Mediated Endocytosis (GO:0048261)	0.6147523	0.7616833	0	0	1.071210014	0.521181995	*ADIPOQ*
Biological Process	Nuclear Membrane Organization (GO:0071763)	0.6147523	0.7616833	0	0	1.071210014	0.521181995	*TARDBP*
Biological Process	Nucleic Acid Phosphodiester Bond Hydrolysis (GO:0090305)	0.6147523	0.7616833	0	0	1.071210014	0.521181995	*DCP1A*
Biological Process	Positive Regulation Of Monooxygenase Activity (GO:0032770)	0.6147523	0.7616833	0	0	1.071210014	0.521181995	*PIK3CB*
Biological Process	Positive Regulation Of Phosphatidylinositol 3-Kinase Activity (GO:0043552)	0.6147523	0.7616833	0	0	1.071210014	0.521181995	*ATG14*
Biological Process	Protein Targeting To Lysosome (GO:0006622)	0.6147523	0.7616833	0	0	1.071210014	0.521181995	*ATG14*
Biological Process	Regulation Of Cardiac Muscle Cell Contraction (GO:0086004)	0.6147523	0.7616833	0	0	1.071210014	0.521181995	*CACNA1C*
Biological Process	Regulation Of Phagocytosis (GO:0050764)	0.6228503	0.7709955	0	0	0.956227616	0.452725118	*ADIPOQ;CD47*
Biological Process	Muscle Organ Development (GO:0007517)	0.6228503	0.7709955	0	0	0.956227616	0.452725118	*MEF2A;MEF2D*

Biological Process	Regulation Of Vesicle-Mediated Transport (GO:0060627)	0.6260839	0.7713133	0	0	0.933832831	0.437286715	*YIPF5;MAPK1;TPCN2*
Biological Process	Positive Regulation Of Peptidyl-Serine Phosphorylation (GO:0033138)	0.6260839	0.7713133	0	0	0.933832831	0.437286715	*SPRY2;RAF1;FNIP1*
Biological Process	DNA Synthesis Involved In DNA Repair (GO:0000731)	0.6286393	0.7713133	0	0	1.029956136	0.478103249	*VCP*
Biological Process	GPI Anchor Metabolic Process (GO:0006505)	0.6286393	0.7713133	0	0	1.029956136	0.478103249	*SLC30A5*
Biological Process	Regulation Of Extrinsic Apoptotic Signaling Pathway In Absence Of Ligand (GO:2001239)	0.6286393	0.7713133	0	0	1.029956136	0.478103249	*EYA3*
Biological Process	Activation Of Phospholipase C Activity (GO:0007202)	0.6286393	0.7713133	0	0	1.029956136	0.478103249	*AVPR1B*
Biological Process	Regulation Of Microtubule Polymerization Or Depolymerization (GO:0031110)	0.6286393	0.7713133	0	0	1.029956136	0.478103249	*CLASP2*
Biological Process	Cardiac Muscle Cell Differentiation (GO:0055007)	0.6286393	0.7713133	0	0	1.029956136	0.478103249	*TTN*
Biological Process	Cellular Response To Epidermal Growth Factor Stimulus (GO:0071364)	0.6286393	0.7713133	0	0	1.029956136	0.478103249	*PDPK1*
Biological Process	Establishment Of Protein Localization To Vacuole (GO:0072666)	0.6286393	0.7713133	0	0	1.029956136	0.478103249	*VPS13A*
Biological Process	Glycerophospholipid Catabolic Process (GO:0046475)	0.6286393	0.7713133	0	0	1.029956136	0.478103249	*GDPD1*
Biological Process	Iron-Sulfur Cluster Assembly (GO:0016226)	0.6286393	0.7713133	0	0	1.029956136	0.478103249	*IBA57*
Biological Process	mRNA Modification (GO:0016556)	0.6286393	0.7713133	0	0	1.029956136	0.478103249	*WTAP*
Biological Process	Metallo-Sulfur Cluster Assembly (GO:0031163)	0.6286393	0.7713133	0	0	1.029956136	0.478103249	*IBA57*
Biological Process	Negative Regulation Of Notch Signaling Pathway (GO:0045746)	0.6286393	0.7713133	0	0	1.029956136	0.478103249	*HEY1*
Biological Process	Negative Regulation Of Cellular Response To Insulin Stimulus (GO:1900077)	0.6286393	0.7713133	0	0	1.029956136	0.478103249	*NCK1*
Biological Process	Regulation Of SMAD Protein Signal Transduction (GO:0060390)	0.6286393	0.7713133	0	0	1.029956136	0.478103249	*SH2B1*
Biological Process	Regulation Of Actin Polymerization Or Depolymerization (GO:0008064)	0.6286393	0.7713133	0	0	1.029956136	0.478103249	*RASA1*
Biological Process	Regulation Of Catecholamine Secretion (GO:0050433)	0.6286393	0.7713133	0	0	1.029956136	0.478103249	*SYT4*
Biological Process	Anterior/Posterior Pattern Specification (GO:0009952)	0.6321619	0.7738432	0	0	0.939402825	0.430819344	*HES7;HEY1*
Biological Process	Heart Morphogenesis (GO:0003007)	0.6321619	0.7738432	0	0	0.939402825	0.430819344	*HEY1;TTN*
Biological Process	Microtubule Cytoskeleton Organization Involved In Mitosis (GO:1902850)	0.6321619	0.7738432	0	0	0.939402825	0.430819344	*CENPH;CLASP2*
Biological Process	Negative Regulation Of Cytokine-Mediated Signaling Pathway (GO:0001960)	0.6321619	0.7738432	0	0	0.939402825	0.430819344	*YTHDF3;XIAP*
Biological Process	Positive Regulation Of Proteolysis (GO:0045862)	0.6321619	0.7738432	0	0	0.939402825	0.430819344	*FBXW11;BTRC*
Biological Process	Lymphocyte Differentiation (GO:0030098)	0.6410566	0.7787214	0	0	0.912514264	0.40573812	*NFIL3;FNIP1;SOX4*
Biological Process	rRNA Metabolic Process (GO:0016072)	0.6410566	0.7787214	0	0	0.912514264	0.40573812	*XRN1;DIS3;NOLC1*
Biological Process	Response To Retinoic Acid (GO:0032526)	0.6412929	0.7787214	0	0	0.923158198	0.410130497	*GSK3B;CREB1*
Biological Process	Negative Regulation Of Peptidase Activity (GO:0010466)	0.6412929	0.7787214	0	0	0.923158198	0.410130497	*SPOCK2;SPOCK1*
Biological Process	protein-DNA Complex Organization (GO:0071824)	0.6412929	0.7787214	0	0	0.923158198	0.410130497	*ATRX;HNRNPU*
Biological Process	Regulation Of Glucose Metabolic Process (GO:0010906)	0.6420263	0.7787214	0	0	0.9917581	0.439473834	*FOXK1*

Biological Process	Regulation Of Monoatomic Ion Transport (GO:0043269)	0.6420263	0.7787214	0	0	0.9917581	0.439473834	*KCNJ3*
Biological Process	Establishment Of Endothelial Barrier (GO:0061028)	0.6420263	0.7787214	0	0	0.9917581	0.439473834	*RAPGEF6*
Biological Process	Sterol Biosynthetic Process (GO:0016126)	0.6420263	0.7787214	0	0	0.9917581	0.439473834	*HMGCR*
Biological Process	Hematopoietic Progenitor Cell Differentiation (GO:0002244)	0.6420263	0.7787214	0	0	0.9917581	0.439473834	*MLLT3*
Biological Process	Limb Development (GO:0060173)	0.6420263	0.7787214	0	0	0.9917581	0.439473834	*ZNRF3*
Biological Process	Membrane Fusion (GO:0061025)	0.6420263	0.7787214	0	0	0.9917581	0.439473834	*STX3*
Biological Process	Negative Regulation Of Cytoskeleton Organization (GO:0051494)	0.6420263	0.7787214	0	0	0.9917581	0.439473834	*ARPIN*
Biological Process	Phospholipid Catabolic Process (GO:0009395)	0.6420263	0.7787214	0	0	0.9917581	0.439473834	*GDPD1*
Biological Process	Positive Regulation Of Protein Import Into Nucleus (GO:0042307)	0.6420263	0.7787214	0	0	0.9917581	0.439473834	*EP300*
Biological Process	Primary Neural Tube Formation (GO:0014020)	0.6420263	0.7787214	0	0	0.9917581	0.439473834	*BCL10*
Biological Process	Protein Insertion Into Mitochondrial Membrane (GO:0051204)	0.6420263	0.7787214	0	0	0.9917581	0.439473834	*TIMM29*
Biological Process	Pyroptosis (GO:0070269)	0.6420263	0.7787214	0	0	0.9917581	0.439473834	*CASP3*
Biological Process	Regulation Of Calcium Ion-Dependent Exocytosis (GO:0017158)	0.6420263	0.7787214	0	0	0.9917581	0.439473834	*SYT4*
Biological Process	Regulation Of Cardiac Muscle Cell Action Potential (GO:0098901)	0.6420263	0.7787214	0	0	0.9917581	0.439473834	*CACNA1C*
Biological Process	Fatty Acid Metabolic Process (GO:0006631)	0.6443557	0.7808317	0	0	0.907205757	0.39872095	*ELOVL4;ADIPOQ;THEM4;PPARGC1A*
Biological Process	Receptor-Mediated Endocytosis (GO:0006898)	0.6443557	0.7808317	0	0	0.907205757	0.39872095	*LRP1B;SNAP91;TPCN2;LRP6*
Biological Process	Autophagosome Organization (GO:1905037)	0.6502444	0.786452	0	0	0.907464237	0.390578927	*WDR45B;ATG14*
Biological Process	Positive Regulation Of Interleukin-8 Production (GO:0032757)	0.6502444	0.786452	0	0	0.907464237	0.390578927	*STAT3;BCL10*
Biological Process	Modulation Of Chemical Synaptic Transmission (GO:0050804)	0.6506901	0.786452	0	0	0.899535233	0.386549886	*GSK3B;RELN;ADIPOQ;CASK*
Biological Process	Cilium Assembly (GO:0060271)	0.6510563	0.786452	0	0	0.910590485	0.390788234	*TTC26;TBC1D32;KIF3A;TCTN2;TTC21B;RABL2B;PHIP;RPGRIP1L*
Biological Process	Phosphate-Containing Compound Metabolic Process (GO:0006796)	0.653985	0.786452	0	0	0.901803418	0.382969659	*DUSP5;PPM1A;GFPT1;STAT3;PLAA;PIK3CB*
Biological Process	RNA Biosynthetic Process (GO:0032774)	0.6549314	0.786452	0	0	0.956288496	0.404725051	*NCOA6*
Biological Process	TOR Signaling (GO:0031929)	0.6549314	0.786452	0	0	0.956288496	0.404725051	*FNIP1*
Biological Process	Alpha-Amino Acid Metabolic Process (GO:1901605)	0.6549314	0.786452	0	0	0.956288496	0.404725051	*RIMKLB*
Biological Process	Regulation Of Monoatomic Ion Transmembrane Transport (GO:0034765)	0.6549314	0.786452	0	0	0.956288496	0.404725051	*KCNJ3*
Biological Process	Cardiac Muscle Cell Action Potential (GO:0086001)	0.6549314	0.786452	0	0	0.956288496	0.404725051	*CACNA1C*
Biological Process	Regulation Of Voltage-Gated Calcium Channel Activity (GO:1901385)	0.6549314	0.786452	0	0	0.956288496	0.404725051	*CACNB4*
Biological Process	Response To Steroid Hormone (GO:0048545)	0.6549314	0.786452	0	0	0.956288496	0.404725051	*UBE3A*
Biological Process	Establishment Of Mitotic Spindle Orientation (GO:0000132)	0.6549314	0.786452	0	0	0.956288496	0.404725051	*FBXW11*
Biological Process	Tube Closure (GO:0060606)	0.6549314	0.786452	0	0	0.956288496	0.404725051	*BCL10*
Biological Process	Maintenance Of Blood-Brain Barrier (GO:0035633)	0.6549314	0.786452	0	0	0.956288496	0.404725051	*CLDN12*
Biological Process	Multivesicular Body Assembly (GO:0036258)	0.6549314	0.786452	0	0	0.956288496	0.404725051	*PDCD6IP*

Biological Process	Negative Regulation Of Plasma Membrane Bounded Cell Projection Assembly (GO:0120033)	0.6549314	0.786452	0	0	0.956288496	0.404725051	*TBC1D30*
Biological Process	Peptidyl-Tyrosine Dephosphorylation (GO:0035335)	0.6549314	0.786452	0	0	0.956288496	0.404725051	*DUSP5*
Biological Process	Positive Regulation Of Endopeptidase Activity (GO:0010950)	0.6549314	0.786452	0	0	0.956288496	0.404725051	*STAT3*
Biological Process	Protein Insertion Into ER Membrane (GO:0045048)	0.6549314	0.786452	0	0	0.956288496	0.404725051	*NOMO3*
Biological Process	Positive Regulation Of Protein Kinase B Signaling (GO:0051897)	0.6556026	0.786545	0	0	0.892143189	0.376663222	*SPRY2;PIK3CB;MTDH*
Biological Process	Protein O-linked Glycosylation (GO:0006493)	0.6556026	0.786545	0	0	0.892143189	0.376663222	*POGLUT1;FUT9;TMTC3*
Biological Process	RNA Methylation (GO:0001510)	0.6590174	0.789212	0	0	0.892293408	0.372091162	*WTAP;TRDMT1*
Biological Process	Cell Junction Organization (GO:0034330)	0.6590174	0.789212	0	0	0.892293408	0.372091162	*PPFIA1;RAB39B*
Biological Process	Positive Regulation Of Apoptotic Signaling Pathway (GO:2001235)	0.6590174	0.789212	0	0	0.892293408	0.372091162	*SIAH1;BCL10*
Biological Process	Regulation Of T Cell Activation (GO:0050863)	0.6590174	0.789212	0	0	0.892293408	0.372091162	*CD209;CD47*
Biological Process	Cellular Response To Growth Factor Stimulus (GO:0071363)	0.660159	0.790222	0	0	0.891841492	0.370359081	*PDPK1;CTNNB1;SPRY2;CRKL;APPL1*
Biological Process	Regulation Of Mitotic Cell Cycle (GO:0007346)	0.6631321	0.7934082	0	0	0.884574542	0.363366527	*TFAP4;HNRNPU;PKN2;ZNF268*
Biological Process	GPI Anchor Biosynthetic Process (GO:0006506)	0.6673718	0.7934082	0	0	0.923265071	0.373375722	*SLC30A5*
Biological Process	Regulation Of Macrophage Derived Foam Cell Differentiation (GO:0010743)	0.6673718	0.7934082	0	0	0.923265071	0.373375722	*ADIPOQ*
Biological Process	Cardiac Muscle Cell Action Potential Involved In Contraction (GO:0086002)	0.6673718	0.7934082	0	0	0.923265071	0.373375722	*CACNA1C*
Biological Process	Embryonic Skeletal System Morphogenesis (GO:0048704)	0.6673718	0.7934082	0	0	0.923265071	0.373375722	*OSR2*
Biological Process	Extrinsic Apoptotic Signaling Pathway Via Death Domain Receptors (GO:0008625)	0.6673718	0.7934082	0	0	0.923265071	0.373375722	*DDX3X*
Biological Process	Homologous Chromosome Pairing At Meiosis (GO:0007129)	0.6673718	0.7934082	0	0	0.923265071	0.373375722	*P3H4*
Biological Process	Lamellipodium Organization (GO:0097581)	0.6673718	0.7934082	0	0	0.923265071	0.373375722	*ARHGEF7*
Biological Process	Maturation Of SSU-rRNA From Tricistronic rRNA Transcript (SSU-rRNA, 5.8S rRNA, LSU-rRNA) (GO:0000462)	0.6673718	0.7934082	0	0	0.923265071	0.373375722	*MRPS11*
Biological Process	Multivesicular Body Organization (GO:0036257)	0.6673718	0.7934082	0	0	0.923265071	0.373375722	*PDCD6IP*
Biological Process	Nucleic Acid Metabolic Process (GO:0090304)	0.6673718	0.7934082	0	0	0.923265071	0.373375722	*RBFOX2*
Biological Process	Positive Regulation Of Interleukin-10 Production (GO:0032733)	0.6673718	0.7934082	0	0	0.923265071	0.373375722	*STAT3*
Biological Process	Positive Regulation Of Lipid Kinase Activity (GO:0090218)	0.6673718	0.7934082	0	0	0.923265071	0.373375722	*ATG14*
Biological Process	Prostaglandin Metabolic Process (GO:0006693)	0.6673718	0.7934082	0	0	0.923265071	0.373375722	*PLAA*
Biological Process	Regulation Of Arp2/3 Complex-Mediated Actin Nucleation (GO:0034315)	0.6673718	0.7934082	0	0	0.923265071	0.373375722	*WASF3*
Biological Process	Positive Regulation Of Mitotic Cell Cycle Phase Transition (GO:1901992)	0.667613	0.7934082	0	0	0.877619983	0.354599343	*DDX3X;CUL3*
Biological Process	Protein-Containing Complex Organization (GO:0043933)	0.6692389	0.7949838	0	0	0.877278139	0.352327321	*TFAP4;REPS2;PPARGC1A;WASF3*

Biological Process	Positive Regulation Of Epithelial Cell Migration (GO:0010634)	0.6697205	0.7951993	0	0	0.872657813	0.349844009	*EPB41L4B;PIK3CB;CLASP2*
Biological Process	Cellular Response To Cytokine Stimulus (GO:0071345)	0.6747851	0.7984189	0	0	0.897154741	0.352905726	*GSK3B;NFAT5;FER;TRAF6;STAT3;HNRNPU;CD47;SOS1;CBL;RBMX*
Biological Process	Regulation Of Tumor Necrosis Factor Production (GO:0032680)	0.6752693	0.7984189	0	0	0.870100377	0.341639378	*ARFGEF2;STAT3;CD47;RASGRP1*
Biological Process	Regulation Of Secretion By Cell (GO:1903530)	0.6760328	0.7984189	0	0	0.863419894	0.338040728	*CASK;TPCN2*
Biological Process	Response To Metal Ion (GO:0010038)	0.6760328	0.7984189	0	0	0.863419894	0.338040728	*SLC11A2;TTN*
Biological Process	Positive Regulation Of Supramolecular Fiber Organization (GO:1902905)	0.6766187	0.7984189	0	0	0.863229406	0.337218268	*USP8;FER;NCK1*
Biological Process	Defense Response To Virus (GO:0051607)	0.6791734	0.7984189	0	0	0.87693438	0.339267308	*UBE2W;TRIM7;TRAF6;MID2;MICA;NCK1*
Biological Process	B Cell Proliferation (GO:0042100)	0.6793644	0.7984189	0	0	0.892443208	0.345016456	*RASGRP1*
Biological Process	Actin Filament Polymerization (GO:0030041)	0.6793644	0.7984189	0	0	0.892443208	0.345016456	*WASF3*
Biological Process	Regulation Of Protein Autophosphorylation (GO:0031952)	0.6793644	0.7984189	0	0	0.892443208	0.345016456	*DDX3X*
Biological Process	Cardiac Muscle Tissue Morphogenesis (GO:0055008)	0.6793644	0.7984189	0	0	0.892443208	0.345016456	*TTN*
Biological Process	Central Nervous System Neuron Differentiation (GO:0021953)	0.6793644	0.7984189	0	0	0.892443208	0.345016456	*SPOCK1*
Biological Process	Toll-Like Receptor Signaling Pathway (GO:0002224)	0.6793644	0.7984189	0	0	0.892443208	0.345016456	*TRAF6*
Biological Process	Glutamine Family Amino Acid Metabolic Process (GO:0009064)	0.6793644	0.7984189	0	0	0.892443208	0.345016456	*RIMKLB*
Biological Process	Leukocyte Cell-Cell Adhesion (GO:0007159)	0.6793644	0.7984189	0	0	0.892443208	0.345016456	*CD209*
Biological Process	Modulation Of Excitatory Postsynaptic Potential (GO:0098815)	0.6793644	0.7984189	0	0	0.892443208	0.345016456	*RELN*
Biological Process	Positive Regulation Of G1/S Transition Of Mitotic Cell Cycle (GO:1900087)	0.6793644	0.7984189	0	0	0.892443208	0.345016456	*DDX3X*
Biological Process	Positive Regulation Of Cholesterol Transport (GO:0032376)	0.6793644	0.7984189	0	0	0.892443208	0.345016456	*ADIPOQ*
Biological Process	Positive Regulation Of Extrinsic Apoptotic Signaling Pathway (GO:2001238)	0.6793644	0.7984189	0	0	0.892443208	0.345016456	*BCL10*
Biological Process	Positive Regulation Of Lamellipodium Organization (GO:1902745)	0.6793644	0.7984189	0	0	0.892443208	0.345016456	*ARHGEF7*
Biological Process	Positive Regulation Of Leukocyte Migration (GO:0002687)	0.6793644	0.7984189	0	0	0.892443208	0.345016456	*MIA3*
Biological Process	Positive Regulation Of Mononuclear Cell Migration (GO:0071677)	0.6793644	0.7984189	0	0	0.892443208	0.345016456	*CD47*
Biological Process	Protein Localization To Chromatin (GO:0071168)	0.6793644	0.7984189	0	0	0.892443208	0.345016456	*ZMYND8*
Biological Process	Receptor Signaling Pathway Via STAT (GO:0097696)	0.6793644	0.7984189	0	0	0.892443208	0.345016456	*STAT3*
Biological Process	Positive Regulation Of T Cell Proliferation (GO:0042102)	0.6842781	0.8038371	0	0	0.849670602	0.322357234	*CD209;NCK1*
Biological Process	Mitochondrial Translation (GO:0032543)	0.6900944	0.8059272	0	0	0.844968069	0.313421407	*MRPL2;MRPS27;MRPS11*
Biological Process	Phosphatidylinositol Biosynthetic Process (GO:0006661)	0.6900944	0.8059272	0	0	0.844968069	0.313421407	*EFR3A;SLC30A5;PIK3CB*
Biological Process	Regulation Of Cholesterol Efflux (GO:0010874)	0.6909251	0.8059272	0	0	0.863609852	0.319297128	*ADIPOQ*
Biological Process	Amide Transport (GO:0042886)	0.6909251	0.8059272	0	0	0.863609852	0.319297128	*SLC25A32*
Biological Process	Cellular Nitrogen Compound Biosynthetic Process (GO:0044271)	0.6909251	0.8059272	0	0	0.863609852	0.319297128	*SLC7A6*

Biological Process	Regulation Of Transmembrane Transport (GO:0034762)	0.6909251	0.8059272	0	0	0.863609852	0.319297128	*KCNJ3*
Biological Process	Female Gamete Generation (GO:0007292)	0.6909251	0.8059272	0	0	0.863609852	0.319297128	*USP9X*
Biological Process	Gluconeogenesis (GO:0006094)	0.6909251	0.8059272	0	0	0.863609852	0.319297128	*PPARGC1A*
Biological Process	Very Long-Chain Fatty Acid Metabolic Process (GO:0000038)	0.6909251	0.8059272	0	0	0.863609852	0.319297128	*ELOVL4*
Biological Process	Heterochromatin Formation (GO:0031507)	0.6909251	0.8059272	0	0	0.863609852	0.319297128	*TRIP12*
Biological Process	Intracellular pH Reduction (GO:0051452)	0.6909251	0.8059272	0	0	0.863609852	0.319297128	*TPCN2*
Biological Process	Muscle Cell Development (GO:0055001)	0.6909251	0.8059272	0	0	0.863609852	0.319297128	*TTN*
Biological Process	Positive Regulation Of Synapse Assembly (GO:0051965)	0.6909251	0.8059272	0	0	0.863609852	0.319297128	*EPHB1*
Biological Process	Proteoglycan Biosynthetic Process (GO:0030166)	0.6909251	0.8059272	0	0	0.863609852	0.319297128	*GLCE*
Biological Process	Purine Ribonucleoside Triphosphate Metabolic Process (GO:0009205)	0.6909251	0.8059272	0	0	0.863609852	0.319297128	*GNAI3*
Biological Process	Regulation Of Calcium Ion Transmembrane Transporter Activity (GO:1901019)	0.6909251	0.8059272	0	0	0.863609852	0.319297128	*CACNB4*
Biological Process	Negative Regulation Of Endopeptidase Activity (GO:0010951)	0.6923507	0.8072346	0	0	0.836350975	0.307495038	*SPOCK2;SPOCK1*
Biological Process	Neuron Projection Development (GO:0031175)	0.6937049	0.8084577	0	0	0.862654739	0.315480218	*GSK3B;TBC1D23;CNTN4;STX3;PPP1R9A;EPHB1*
Biological Process	Axon Development (GO:0061564)	0.6966722	0.8103627	0	0	0.836122734	0.302208439	*NREP;CNTN4;ZDHHC17*
Biological Process	Regulation Of Viral Genome Replication (GO:0045069)	0.7002522	0.8103627	0	0	0.823441183	0.293404208	*DDX3X;PKN2*
Biological Process	Protein Secretion (GO:0009306)	0.7002522	0.8103627	0	0	0.823441183	0.293404208	*AP1G1;MIA3*
Biological Process	Regulation Of Lamellipodium Assembly (GO:0010591)	0.7020696	0.8103627	0	0	0.836578581	0.295916857	*FER*
Biological Process	Carboxylic Acid Metabolic Process (GO:0019752)	0.7020696	0.8103627	0	0	0.836578581	0.295916857	*CARNMT1*
Biological Process	Cardiac Muscle Contraction (GO:0060048)	0.7020696	0.8103627	0	0	0.836578581	0.295916857	*TTN*
Biological Process	Regulation Of Protein Polymerization (GO:0032271)	0.7020696	0.8103627	0	0	0.836578581	0.295916857	*RASA1*
Biological Process	Regulation Of Type I Interferon-Mediated Signaling Pathway (GO:0060338)	0.7020696	0.8103627	0	0	0.836578581	0.295916857	*YTHDF3*
Biological Process	Epithelial Tube Morphogenesis (GO:0060562)	0.7020696	0.8103627	0	0	0.836578581	0.295916857	*CTNNB1*
Biological Process	Ubiquitin-Dependent Protein Catabolic Process Via The Multivesicular Body Sorting Pathway (GO:0043162)	0.7020696	0.8103627	0	0	0.836578581	0.295916857	*PLAA*
Biological Process	Vasculogenesis (GO:0001570)	0.7020696	0.8103627	0	0	0.836578581	0.295916857	*RASA1*
Biological Process	intra-Golgi Vesicle-Mediated Transport (GO:0006891)	0.7020696	0.8103627	0	0	0.836578581	0.295916857	*COG6*
Biological Process	Lung Development (GO:0030324)	0.7020696	0.8103627	0	0	0.836578581	0.295916857	*PHF14*
Biological Process	Negative Regulation Of Endocytosis (GO:0045806)	0.7020696	0.8103627	0	0	0.836578581	0.295916857	*ADIPOQ*
Biological Process	Positive Regulation Of DNA Binding (GO:0043388)	0.7020696	0.8103627	0	0	0.836578581	0.295916857	*TRAF6*
Biological Process	Positive Regulation Of T Cell Mediated Cytotoxicity (GO:0001916)	0.7020696	0.8103627	0	0	0.836578581	0.295916857	*MICA*
Biological Process	Positive Regulation Of Cytokine Production Involved In Immune Response (GO:0002720)	0.7020696	0.8103627	0	0	0.836578581	0.295916857	*TRAF6*

Biological Process	Positive Regulation Of Ion Transmembrane Transporter Activity (GO:0032414)	0.7020696	0.8103627	0	0	0.836578581	0.295916857 *RELN*
Biological Process	Positive Regulation Of Protein Dephosphorylation (GO:0035307)	0.7020696	0.8103627	0	0	0.836578581	0.295916857 *SPPL3*
Biological Process	Positive Regulation Of Release Of Sequestered Calcium Ion Into Cytosol (GO:0051281)	0.7020696	0.8103627	0	0	0.836578581	0.295916857 *PDPK1*
Biological Process	Regulation Of NLRP3 Inflammasome Complex Assembly (GO:1900225)	0.7020696	0.8103627	0	0	0.836578581	0.295916857 *DDX3X*
Biological Process	Regulation Of Cell Adhesion Mediated By Integrin (GO:0033628)	0.7020696	0.8103627	0	0	0.836578581	0.295916857 *PDE3B*
Biological Process	B Cell Differentiation (GO:0030183)	0.7079845	0.8161231	0	0	0.810922596	0.280038378 *IRF2BP2;FNIP1*
Biological Process	Skin Development (GO:0043588)	0.7079845	0.8161231	0	0	0.810922596	0.280038378 *CASP3;AHDC1*
Biological Process	Intermediate Filament Organization (GO:0045109)	0.7079845	0.8161231	0	0	0.810922596	0.280038378 *DNAJB6;KRT37*
Biological Process	Hemopoiesis (GO:0030097)	0.7095095	0.8167133	0	0	0.818973615	0.28105651 *NCOA6;KAT6A;HIPK1*
Biological Process	Cytokine-Mediated Signaling Pathway (GO:0019221)	0.7109464	0.8167133	0	0	0.858760886	0.292973353 *FER;TRAF6;MFAP3;STAT3;CXCL13;SOS1;CBL;APPL1*
Biological Process	Apoptotic Mitochondrial Changes (GO:0008637)	0.7128128	0.8167133	0	0	0.811185569	0.274615907 *THEM4*
Biological Process	Regulation Of Phosphatidylinositol 3-Kinase Activity (GO:0043551)	0.7128128	0.8167133	0	0	0.811185569	0.274615907 *ATG14*
Biological Process	Cellular Response To Amyloid-Beta (GO:1904646)	0.7128128	0.8167133	0	0	0.811185569	0.274615907 *GSK3B*
Biological Process	Cellular Response To Vascular Endothelial Growth Factor Stimulus (GO:0035924)	0.7128128	0.8167133	0	0	0.811185569	0.274615907 *SPRY2*
Biological Process	Response To Estrogen (GO:0043627)	0.7128128	0.8167133	0	0	0.811185569	0.274615907 *EP300*
Biological Process	Embryonic Skeletal System Development (GO:0048706)	0.7128128	0.8167133	0	0	0.811185569	0.274615907 *OSR2*
Biological Process	Microtubule Polymerization (GO:0046785)	0.7128128	0.8167133	0	0	0.811185569	0.274615907 *CLASP2*
Biological Process	Mitochondrial Electron Transport, NADH To Ubiquinone (GO:0006120)	0.7128128	0.8167133	0	0	0.811185569	0.274615907 *NDUFC2*
Biological Process	Negative Regulation Of Cell Cycle Process (GO:0010948)	0.7128128	0.8167133	0	0	0.811185569	0.274615907 *BAZ1B*
Biological Process	Positive Regulation Of Immunoglobulin Production (GO:0002639)	0.7128128	0.8167133	0	0	0.811185569	0.274615907 *KMT5B*
Biological Process	Positive Regulation Of Neuron Death (GO:1901216)	0.7128128	0.8167133	0	0	0.811185569	0.274615907 *CTNNB1*
Biological Process	Regulation Of DNA Biosynthetic Process (GO:2000278)	0.7128128	0.8167133	0	0	0.811185569	0.274615907 *ADIPOQ*
Biological Process	Regulation Of Endopeptidase Activity (GO:0052548)	0.7155495	0.8187865	0	0	0.798777699	0.267354448 *SPOCK2;SPOCK1*
Biological Process	Regulation Of Monoatomic Ion Transmembrane Transporter Activity (GO:0032412)	0.7155495	0.8187865	0	0	0.798777699	0.267354448 *RELN;OPRM1*
Biological Process	Response To Molecule Of Bacterial Origin (GO:0002237)	0.7155495	0.8187865	0	0	0.798777699	0.267354448 *FER;HNRNPA0*
Biological Process	Inorganic Cation Import Across Plasma Membrane (GO:0098659)	0.7219252	0.8214729	0	0	0.80251046	0.261484941 *CACNA1C;SLC12A6;KCNJ3*
Biological Process	Mitochondrial Gene Expression (GO:0140053)	0.7219252	0.8214729	0	0	0.80251046	0.261484941 *MRPL2;MRPS27;MRPS11*
Biological Process	Negative Regulation Of Epithelial Cell Proliferation (GO:0050680)	0.7229493	0.8214729	0	0	0.786990005	0.255312319 *NFIB;CASK*

Biological Process	Positive Regulation Of Protein Polymerization (GO:0032273)	0.7229493	0.8214729	0	0	0.786990005	0.255312319	*FER;NCK1*
Biological Process	Regulation Of Cellular Senescence (GO:2000772)	0.7231691	0.8214729	0	0	0.787286264	0.255169113	*ING2*
Biological Process	Action Potential (GO:0001508)	0.7231691	0.8214729	0	0	0.787286264	0.255169113	*KCND2*
Biological Process	Regulation Of Postsynaptic Membrane Potential (GO:0060078)	0.7231691	0.8214729	0	0	0.787286264	0.255169113	*GSK3B*
Biological Process	Regulation Of Regulated Secretory Pathway (GO:1903305)	0.7231691	0.8214729	0	0	0.787286264	0.255169113	*SYT4*
Biological Process	Endoderm Formation (GO:0001706)	0.7231691	0.8214729	0	0	0.787286264	0.255169113	*DUSP5*
Biological Process	Skeletal Muscle Organ Development (GO:0060538)	0.7231691	0.8214729	0	0	0.787286264	0.255169113	*SIX4*
Biological Process	Establishment Of Spindle Orientation (GO:0051294)	0.7231691	0.8214729	0	0	0.787286264	0.255169113	*FBXW11*
Biological Process	Hexose Biosynthetic Process (GO:0019319)	0.7231691	0.8214729	0	0	0.787286264	0.255169113	*PPARGC1A*
Biological Process	Neutral Amino Acid Transport (GO:0015804)	0.7231691	0.8214729	0	0	0.787286264	0.255169113	*SLC7A6*
Biological Process	One-Carbon Compound Transport (GO:0019755)	0.7231691	0.8214729	0	0	0.787286264	0.255169113	*SLC4A4*
Biological Process	Platelet Aggregation (GO:0070527)	0.7231691	0.8214729	0	0	0.787286264	0.255169113	*PIK3CB*
Biological Process	Positive Regulation Of Cell Development (GO:0010720)	0.7231691	0.8214729	0	0	0.787286264	0.255169113	*OPRM1*
Biological Process	Regulation Of Axon Extension (GO:0030516)	0.7231691	0.8214729	0	0	0.787286264	0.255169113	*GSK3B*
Biological Process	Positive Regulation Of Vasculature Development (GO:1904018)	0.7279764	0.8265792	0	0	0.794523385	0.252250615	*PDPK1;HIPK1;MTDH*
Biological Process	Synapse Assembly (GO:0007416)	0.7301858	0.8283778	0	0	0.775543983	0.243874646	*SPOCK2;ADGRL3*
Biological Process	Negative Regulation Of Protein Binding (GO:0032091)	0.7301858	0.8283778	0	0	0.775543983	0.243874646	*GSK3B;ADIPOQ*
Biological Process	Regulation Of Intracellular Protein Transport (GO:0033157)	0.7331524	0.8292574	0	0	0.764752633	0.237380503	*GSK3B*
Biological Process	Regulation Of Trans-Synaptic Signaling (GO:0099177)	0.7331524	0.8292574	0	0	0.764752633	0.237380503	*RELN*
Biological Process	tRNA Methylation (GO:0030488)	0.7331524	0.8292574	0	0	0.764752633	0.237380503	*TRDMT1*
Biological Process	Negative Regulation Of Organelle Assembly (GO:1902116)	0.7331524	0.8292574	0	0	0.764752633	0.237380503	*TBC1D30*
Biological Process	Negative Regulation Of Protein Kinase B Signaling (GO:0051898)	0.7331524	0.8292574	0	0	0.764752633	0.237380503	*PIK3CB*
Biological Process	Positive Regulation Of Phospholipase C Activity (GO:0010863)	0.7331524	0.8292574	0	0	0.764752633	0.237380503	*AVPR1B*
Biological Process	Regulation Of T Cell Mediated Cytotoxicity (GO:0001914)	0.7331524	0.8292574	0	0	0.764752633	0.237380503	*MICA*
Biological Process	Cellular Response To Hypoxia (GO:0071456)	0.7372613	0.8335489	0	0	0.76442499	0.233006619	*KCND2;HP1BP3*
Biological Process	Aortic Valve Morphogenesis (GO:0003180)	0.7427762	0.8372826	0	0	0.74347087	0.221078875	*HEY1*
Biological Process	Calcium Ion Import Across Plasma Membrane (GO:0098703)	0.7427762	0.8372826	0	0	0.74347087	0.221078875	*CACNA1C*
Biological Process	Mitochondrion Disassembly (GO:0061726)	0.7427762	0.8372826	0	0	0.74347087	0.221078875	*WDR45B*
Biological Process	Negative Regulation Of Smooth Muscle Cell Proliferation (GO:0048662)	0.7427762	0.8372826	0	0	0.74347087	0.221078875	*PPARGC1A*
Biological Process	Positive Regulation Of Receptor Signaling Pathway Via JAK-STAT (GO:0046427)	0.7427762	0.8372826	0	0	0.74347087	0.221078875	*EP300*
Biological Process	Positive Regulation Of Receptor Signaling Pathway Via STAT (GO:1904894)	0.7427762	0.8372826	0	0	0.74347087	0.221078875	*EP300*
Biological Process	Regulation Of Cellular Component Biogenesis (GO:0044087)	0.7427762	0.8372826	0	0	0.74347087	0.221078875	*HCFC1*

Biological Process	Cellular Response To Decreased Oxygen Levels (GO:0036294)	0.7441779	0.8381493	0	0	0.753619208	0.222675765	*KCND2;HP1BP3*
Biological Process	Positive Regulation Of Secretion By Cell (GO:1903532)	0.7441779	0.8381493	0	0	0.753619208	0.222675765	*TTN;CLASP2*
Biological Process	Positive Regulation Of Signal Transduction (GO:0009967)	0.7456852	0.83949	0	0	0.828412159	0.243099007	*MBD5;RELN;POGLUT1;STAT3;ADIPOQ;TSPAN5;PHIP;SORBS1*
Biological Process	Cell-Cell Adhesion Via Plasma-Membrane Adhesion Molecules (GO:0098742)	0.7460292	0.8395205	0	0	0.800343369	0.234493068	*CD209;CLDN12;CLDN18;CNTN4;ADGRL3*
Biological Process	Positive Regulation Of Lymphocyte Proliferation (GO:0050671)	0.7509379	0.840941	0	0	0.743113587	0.212851764	*CD209;NCK1*
Biological Process	G2/M Transition Of Mitotic Cell Cycle (GO:0000086)	0.7520534	0.840941	0	0	0.723339473	0.206114141	*KHDRBS1*
Biological Process	Regulation Of Neurotransmitter Secretion (GO:0046928)	0.7520534	0.840941	0	0	0.723339473	0.206114141	*CASK*
Biological Process	Regulation Of Protein Import Into Nucleus (GO:0042306)	0.7520534	0.840941	0	0	0.723339473	0.206114141	*EP300*
Biological Process	Response To Peptide (GO:1901652)	0.7520534	0.840941	0	0	0.723339473	0.206114141	*STAT3*
Biological Process	Response To Transforming Growth Factor Beta (GO:0071559)	0.7520534	0.840941	0	0	0.723339473	0.206114141	*CRKL*
Biological Process	Signal Release From Synapse (GO:0099643)	0.7520534	0.840941	0	0	0.723339473	0.206114141	*LIN7C*
Biological Process	Establishment Of Protein Localization To Plasma Membrane (GO:0061951)	0.7520534	0.840941	0	0	0.723339473	0.206114141	*GORASP1*
Biological Process	Fatty Acid Transport (GO:0015908)	0.7520534	0.840941	0	0	0.723339473	0.206114141	*ARID4A*
Biological Process	Tumor Necrosis Factor-Mediated Signaling Pathway (GO:0033209)	0.7520534	0.840941	0	0	0.723339473	0.206114141	*TRAF6*
Biological Process	Import Into Cell (GO:0098657)	0.7520534	0.840941	0	0	0.723339473	0.206114141	*SLC11A2*
Biological Process	Lymphocyte Proliferation (GO:0046651)	0.7520534	0.840941	0	0	0.723339473	0.206114141	*RASGRP1*
Biological Process	Membrane Fission (GO:0090148)	0.7520534	0.840941	0	0	0.723339473	0.206114141	*EXOC6*
Biological Process	Positive Regulation Of Axonogenesis (GO:0050772)	0.7520534	0.840941	0	0	0.723339473	0.206114141	*PLXNC1*
Biological Process	Positive Regulation Of Bone Mineralization (GO:0030501)	0.7520534	0.840941	0	0	0.723339473	0.206114141	*OSR2*
Biological Process	Proteolysis (GO:0006508)	0.7538674	0.8426138	0	0	0.834507042	0.235780646	*CASP3;TMPRSS4;ERAP1;FBXO6;LNPEP;UBE3A;UFSP2;IDE;SPPL3;SENP1*
Biological Process	Extracellular Structure Organization (GO:0043062)	0.7566907	0.8450564	0	0	0.756848504	0.211009886	*SPOCK2;COL22A1;ST7*
Biological Process	Regulation Of Wnt Signaling Pathway (GO:0030111)	0.7566907	0.8450564	0	0	0.756848504	0.211009886	*ZNF703;BTRC;VGLL4*
Biological Process	Regulation Of Nitric Oxide Biosynthetic Process (GO:0045428)	0.7609964	0.8470081	0	0	0.704267623	0.192354283	*CD47*
Biological Process	Carboxylic Acid Transmembrane Transport (GO:1905039)	0.7609964	0.8470081	0	0	0.704267623	0.192354283	*SLC7A6*
Biological Process	Regulation Of Protein Tyrosine Kinase Activity (GO:0061097)	0.7609964	0.8470081	0	0	0.704267623	0.192354283	*RELN*
Biological Process	Fibroblast Growth Factor Receptor Signaling Pathway (GO:0008543)	0.7609964	0.8470081	0	0	0.704267623	0.192354283	*RAB14*
Biological Process	Negative Regulation Of Mitochondrion Organization (GO:0010823)	0.7609964	0.8470081	0	0	0.704267623	0.192354283	*SLC35F6*
Biological Process	Nuclear Transport (GO:0051169)	0.7609964	0.8470081	0	0	0.704267623	0.192354283	*NUP153*
Biological Process	Odontogenesis (GO:0042476)	0.7609964	0.8470081	0	0	0.704267623	0.192354283	*OSR2*
Biological Process	Organelle Transport Along Microtubule (GO:0072384)	0.7609964	0.8470081	0	0	0.704267623	0.192354283	*FBXW11*
Biological Process	External Encapsulating Structure Organization (GO:0045229)	0.7621307	0.8479144	0	0	0.74973605	0.203656235	*SPOCK2;COL22A1;ST7*

Biological Process	Positive Regulation Of Interleukin-6 Production (GO:0032755)	0.7639972	0.8492777	0	0	0.722954152	0.194612818	*STAT3;BCL10*
Biological Process	Positive Regulation Of Protein Secretion (GO:0050714)	0.7639972	0.8492777	0	0	0.722954152	0.194612818	*SOX4;TTN*
Biological Process	Regulation Of Cytosolic Calcium Ion Concentration (GO:0051480)	0.7696173	0.8508832	0	0	0.686173817	0.179682815	*ATP2B2*
Biological Process	L-alpha-amino Acid Transmembrane Transport (GO:1902475)	0.7696173	0.8508832	0	0	0.686173817	0.179682815	*SLC7A6*
Biological Process	Notch Signaling Pathway (GO:0007219)	0.7696173	0.8508832	0	0	0.686173817	0.179682815	*HEY1*
Biological Process	Regulation Of Microtubule Polymerization (GO:0031113)	0.7696173	0.8508832	0	0	0.686173817	0.179682815	*DYRK1A*
Biological Process	Aortic Valve Development (GO:0003176)	0.7696173	0.8508832	0	0	0.686173817	0.179682815	*HEY1*
Biological Process	Cell Cycle G2/M Phase Transition (GO:0044839)	0.7696173	0.8508832	0	0	0.686173817	0.179682815	*KHDRBS1*
Biological Process	Cyclic-Nucleotide-Mediated Signaling (GO:0019935)	0.7696173	0.8508832	0	0	0.686173817	0.179682815	*CREB1*
Biological Process	Retinoid Metabolic Process (GO:0001523)	0.7696173	0.8508832	0	0	0.686173817	0.179682815	*ARID4A*
Biological Process	Nucleic Acid-Templated Transcription (GO:0097659)	0.7696173	0.8508832	0	0	0.686173817	0.179682815	*MEF2A*
Biological Process	Positive Regulation Of Cell Cycle G1/S Phase Transition (GO:1902808)	0.7696173	0.8508832	0	0	0.686173817	0.179682815	*DDX3X*
Biological Process	Positive Regulation Of Insulin Secretion (GO:0032024)	0.7696173	0.8508832	0	0	0.686173817	0.179682815	*SOX4*
Biological Process	Positive Regulation Of Myeloid Leukocyte Differentiation (GO:0002763)	0.7696173	0.8508832	0	0	0.686173817	0.179682815	*TRAF6*
Biological Process	Regulation Of Autophagosome Assembly (GO:2000785)	0.7696173	0.8508832	0	0	0.686173817	0.179682815	*RAB3GAP2*
Biological Process	Regulation Of T Cell Proliferation (GO:0042129)	0.7703013	0.8510571	0	0	0.713277623	0.186146577	*CD209;NCK1*
Biological Process	Chemical Synaptic Transmission (GO:0007268)	0.7706236	0.8510571	0	0	0.806232775	0.210068121	*ATXN3;CACNB4;DHX8;KCND2;MAPK1;SLC12A6;LIN7C;LRP6*
Biological Process	Positive Regulation Of Protein Kinase Activity (GO:0045860)	0.7707384	0.8510571	0	0	0.758310551	0.197468806	*DDX3X;RELN;TRAF6;CAMKK2*
Biological Process	Regulation Of Cold-Induced Thermogenesis (GO:0120161)	0.7753282	0.8557685	0	0	0.752930994	0.191597519	*ADIPOQ;LNPEP;PPARGC1A;LGR4*
Biological Process	Positive Regulation Of ERK1 And ERK2 Cascade (GO:0070374)	0.7763501	0.8561405	0	0	0.76786432	0.194386123	*MFAP3;SPRY2;OPRM1;RASGRP1;CRKL*
Biological Process	Plasma Membrane Bounded Cell Projection Assembly (GO:0120031)	0.7774151	0.8561405	0	0	0.800109414	0.201452254	*TTC26;KIF3A;TCTN2;TTC21B;RABL2B;PHIP;ARHGEF7;RAPGEF6*
Biological Process	DNA Duplex Unwinding (GO:0032508)	0.7779276	0.8561405	0	0	0.668984701	0.16799665	*RTEL1*
Biological Process	Regulation Of Dendrite Development (GO:0050773)	0.7779276	0.8561405	0	0	0.668984701	0.16799665	*GSK3B*
Biological Process	Axoneme Assembly (GO:0035082)	0.7779276	0.8561405	0	0	0.668984701	0.16799665	*TTC26*
Biological Process	Heart Contraction (GO:0060047)	0.7779276	0.8561405	0	0	0.668984701	0.16799665	*TTN*
Biological Process	Positive Regulation Of Ossification (GO:0045778)	0.7779276	0.8561405	0	0	0.668984701	0.16799665	*OSR2*
Biological Process	Positive Regulation Of Cytoskeleton Organization (GO:0051495)	0.7824702	0.8596	0	0	0.69467858	0.170404305	*FER;NCK1*
Biological Process	Positive Regulation Of Endocytosis (GO:0045807)	0.7824702	0.8596	0	0	0.69467858	0.170404305	*CD47;CBL*
Biological Process	Regulation Of Interleukin-6 Production (GO:0032675)	0.78291	0.8596	0	0	0.722567756	0.176839481	*STAT3;BCL10;CD47*

Biological Process	Amino Acid Transmembrane Transport (GO:0003333)	0.7859386	0.8596	0	0	0.652634078	0.157204299	*SLC7A6*
Biological Process	Regulation Of Osteoclast Differentiation (GO:0045670)	0.7859386	0.8596	0	0	0.652634078	0.157204299	*TRAF6*
Biological Process	Branching Morphogenesis Of An Epithelial Tube (GO:0048754)	0.7859386	0.8596	0	0	0.652634078	0.157204299	*CTNNB1*
Biological Process	Ceramide Biosynthetic Process (GO:0046513)	0.7859386	0.8596	0	0	0.652634078	0.157204299	*ST3GAL5*
Biological Process	Collagen Fibril Organization (GO:0030199)	0.7859386	0.8596	0	0	0.652634078	0.157204299	*P3H4*
Biological Process	Skeletal Muscle Tissue Development (GO:0007519)	0.7859386	0.8596	0	0	0.652634078	0.157204299	*SIX4*
Biological Process	Substantia Nigra Development (GO:0021762)	0.7859386	0.8596	0	0	0.652634078	0.157204299	*ERCC4*
Biological Process	Keratinocyte Differentiation (GO:0030216)	0.7859386	0.8596	0	0	0.652634078	0.157204299	*CASP3*
Biological Process	Negative Regulation Of I-kappaB kinase/NF-kappaB Signaling (GO:0043124)	0.7859386	0.8596	0	0	0.652634078	0.157204299	*PPM1A*
Biological Process	Organic Hydroxy Compound Biosynthetic Process (GO:1901617)	0.7859386	0.8596	0	0	0.652634078	0.157204299	*HMGCR*
Biological Process	Positive Regulation Of Calcium Ion Transmembrane Transport (GO:1904427)	0.7859386	0.8596	0	0	0.652634078	0.157204299	*PDPK1*
Biological Process	Positive Regulation Of Neuron Differentiation (GO:0045666)	0.7859386	0.8596	0	0	0.652634078	0.157204299	*VWC2*
Biological Process	Regulation Of Cytokinesis (GO:0032465)	0.7883398	0.8615149	0	0	0.685736733	0.163086084	*CUL3;PKN2*
Biological Process	Positive Regulation Of Immune Response (GO:0050778)	0.7883398	0.8615149	0	0	0.685736733	0.163086084	*POLR3C;POLR3F*
Biological Process	Regulation Of Endothelial Cell Apoptotic Process (GO:2000351)	0.793661	0.8644769	0	0	0.637062057	0.147224354	*PDPK1*
Biological Process	Response To Amyloid-Beta (GO:1904645)	0.793661	0.8644769	0	0	0.637062057	0.147224354	*GSK3B*
Biological Process	Skeletal System Morphogenesis (GO:0048705)	0.793661	0.8644769	0	0	0.637062057	0.147224354	*OSR2*
Biological Process	Neurotransmitter Secretion (GO:0007269)	0.793661	0.8644769	0	0	0.637062057	0.147224354	*LIN7C*
Biological Process	Positive Regulation Of Biomineral Tissue Development (GO:0070169)	0.793661	0.8644769	0	0	0.637062057	0.147224354	*OSR2*
Biological Process	Positive Regulation Of Cell-Matrix Adhesion (GO:0001954)	0.793661	0.8644769	0	0	0.637062057	0.147224354	*GSK3B*
Biological Process	Positive Regulation Of Receptor-Mediated Endocytosis (GO:0048260)	0.793661	0.8644769	0	0	0.637062057	0.147224354	*CBL*
Biological Process	Positive Regulation Of Stress Fiber Assembly (GO:0051496)	0.793661	0.8644769	0	0	0.637062057	0.147224354	*CD47*
Biological Process	Regulation Of Interleukin-8 Production (GO:0032677)	0.7940695	0.8645664	0	0	0.677021262	0.1561105	*STAT3;BCL10*
Biological Process	Sodium Ion Transmembrane Transport (GO:0035725)	0.7996616	0.8675909	0	0	0.668523677	0.149459553	*SLC4A4;TPCN2*
Biological Process	Autophagosome Maturation (GO:0097352)	0.8011051	0.8675909	0	0	0.622214316	0.137984189	*VCP*
Biological Process	Bone Development (GO:0060348)	0.8011051	0.8675909	0	0	0.622214316	0.137984189	*OSR2*
Biological Process	Cellular Response To Ketone (GO:1901655)	0.8011051	0.8675909	0	0	0.622214316	0.137984189	*CREB1*
Biological Process	Regulation Of Transport (GO:0051049)	0.8011051	0.8675909	0	0	0.622214316	0.137984189	*SURF4*
Biological Process	Response To Unfolded Protein (GO:0006986)	0.8011051	0.8675909	0	0	0.622214316	0.137984189	*HSPA4L*
Biological Process	Embryonic Organ Morphogenesis (GO:0048562)	0.8011051	0.8675909	0	0	0.622214316	0.137984189	*OSR2*

Biological Process	Lymphocyte Chemotaxis (GO:0048247)	0.8011051	0.8675909	0	0	0.622214316	0.137984189	*CXCL13*
Biological Process	Mitotic Spindle Assembly (GO:0090307)	0.8011051	0.8675909	0	0	0.622214316	0.137984189	*CLASP2*
Biological Process	Negative Regulation Of Immune System Process (GO:0002683)	0.8011051	0.8675909	0	0	0.622214316	0.137984189	*LGR4*
Biological Process	Neural Crest Cell Development (GO:0014032)	0.8011051	0.8675909	0	0	0.622214316	0.137984189	*NOLC1*
Biological Process	Nucleus Organization (GO:0006997)	0.8011051	0.8675909	0	0	0.622214316	0.137984189	*PDCD6IP*
Biological Process	Positive Regulation Of Chemotaxis (GO:0050921)	0.8011051	0.8675909	0	0	0.622214316	0.137984189	*STX3*
Biological Process	Positive Regulation Of Protein Phosphorylation (GO:0001934)	0.8015833	0.867754	0	0	0.801768056	0.177324181	*RELN;DDX3X;ADIPOQ;MAPK1;SPRY2;ATG14;RAF1;FNIP1;RASGRP1;CRKL;CAMKK2*
Biological Process	Phospholipase C-activating G Protein-Coupled Receptor Signaling Pathway (GO:0007200)	0.8051188	0.8712254	0	0	0.660235909	0.143116325	*DHX8;OPRM1*
Biological Process	Positive Regulation Of Angiogenesis (GO:0045766)	0.8067518	0.872636	0	0	0.691242245	0.148436813	*PDPK1;HIPK1;MTDH*
Biological Process	Establishment Of Protein Localization To Extracellular Region (GO:0035592)	0.8082811	0.8728645	0	0	0.608041472	0.129418857	*MIA3*
Biological Process	Triglyceride Metabolic Process (GO:0006641)	0.8082811	0.8728645	0	0	0.608041472	0.129418857	*GPAT3*
Biological Process	Mitotic Nuclear Division (GO:0140014)	0.8082811	0.8728645	0	0	0.608041472	0.129418857	*CLASP2*
Biological Process	Regulation Of Apoptotic Signaling Pathway (GO:2001233)	0.8082811	0.8728645	0	0	0.608041472	0.129418857	*GRINA*
Biological Process	Positive Regulation Of Cell Motility (GO:2000147)	0.8112431	0.8736856	0	0	0.745163182	0.155878821	*FER;SPOCK2;ZNF703;STAT3;SPRY2;ZNF268*
Biological Process	Ribosome Biogenesis (GO:0042254)	0.8134295	0.8736856	0	0	0.707721336	0.146141681	*DIS3;NOLC1;RIOK2;RPF2*
Biological Process	Positive Regulation Of Response To External Stimulus (GO:0032103)	0.8134295	0.8736856	0	0	0.707721336	0.146141681	*POLR3C;POLR3F;CD47;STX3*
Biological Process	Endocytosis (GO:0006897)	0.8147413	0.8736856	0	0	0.725752508	0.148695506	*DENND1B;AP3M1;REPS2;MAPK1;LRP1B*
Biological Process	Regulation Of Fibroblast Proliferation (GO:0048145)	0.8151984	0.8736856	0	0	0.594498532	0.121470145	*CTNNB1*
Biological Process	T Cell Differentiation (GO:0030217)	0.8151984	0.8736856	0	0	0.594498532	0.121470145	*SOX4*
Biological Process	Amino Acid Transport (GO:0006865)	0.8151984	0.8736856	0	0	0.594498532	0.121470145	*SLC7A6*
Biological Process	Development Of Primary Male Sexual Characteristics (GO:0046546)	0.8151984	0.8736856	0	0	0.594498532	0.121470145	*SIX4*
Biological Process	Epithelial Cell Migration (GO:0010631)	0.8151984	0.8736856	0	0	0.594498532	0.121470145	*PKN2*
Biological Process	Homotypic Cell-Cell Adhesion (GO:0034109)	0.8151984	0.8736856	0	0	0.594498532	0.121470145	*PIK3CB*
Biological Process	Intracellular Protein Transmembrane Transport (GO:0065002)	0.8151984	0.8736856	0	0	0.594498532	0.121470145	*SEC61A2*
Biological Process	Mitotic G2/M Transition Checkpoint (GO:0044818)	0.8151984	0.8736856	0	0	0.594498532	0.121470145	*ORC1*
Biological Process	Negative Regulation Of Cellular Metabolic Process (GO:0031324)	0.8151984	0.8736856	0	0	0.594498532	0.121470145	*SIRT5*
Biological Process	Positive Regulation Of Blood Vessel Endothelial Cell Migration (GO:0043536)	0.8151984	0.8736856	0	0	0.594498532	0.121470145	*PDPK1*
Biological Process	Positive Regulation Of Peptide Hormone Secretion (GO:0090277)	0.8151984	0.8736856	0	0	0.594498532	0.121470145	*SOX4*
Biological Process	Regulation Of Osteoblast Differentiation (GO:0045667)	0.8156378	0.8736856	0	0	0.644259489	0.131290332	*ZHX3;SUCO*
Biological Process	Cellular Respiration (GO:0045333)	0.8156378	0.8736856	0	0	0.644259489	0.131290332	*NDUFC2;PPARGC1A*
Biological Process	Integrin-Mediated Signaling Pathway (GO:0007229)	0.8156378	0.8736856	0	0	0.644259489	0.131290332	*CUL3;CD47*
Biological Process	Negative Regulation Of Cell Cycle (GO:0045786)	0.8156378	0.8736856	0	0	0.644259489	0.131290332	*BRINP1;TFAP4*

Biological Process	Positive Regulation Of Endothelial Cell Migration (GO:0010595)	0.8156378	0.8736856	0	0	0.644259489	0.131290332	*PDPK1;PIK3CB*
Biological Process	Potassium Ion Transport (GO:0006813)	0.8199707	0.8779719	0	0	0.673710488	0.13372252	*KCND2;SLC12A6;KCNJ3*
Biological Process	Regulation Of Interleukin-2 Production (GO:0032663)	0.8218666	0.878936	0	0	0.581544416	0.114085763	*TRAF6*
Biological Process	Regulation Of Wound Healing (GO:0061041)	0.8218666	0.878936	0	0	0.581544416	0.114085763	*CASK*
Biological Process	Cognition (GO:0050890)	0.8218666	0.878936	0	0	0.581544416	0.114085763	*SETD5*
Biological Process	Positive Regulation Of Epithelial Cell Proliferation (GO:0050679)	0.8242027	0.8810787	0	0	0.668061367	0.129162134	*OSR2;EPGN;ZNF703*
Biological Process	Regulation Of Type II Interferon Production (GO:0032649)	0.8256462	0.8822657	0	0	0.629034901	0.120516141	*CD47;RASGRP1*
Biological Process	Cellular Response To Mechanical Stimulus (GO:0071260)	0.8282944	0.8830173	0	0	0.569141538	0.10721864	*BCL10*
Biological Process	Chloride Transmembrane Transport (GO:1902476)	0.8282944	0.8830173	0	0	0.569141538	0.10721864	*SLC12A6*
Biological Process	Cytoplasmic Microtubule Organization (GO:0031122)	0.8282944	0.8830173	0	0	0.569141538	0.10721864	*HOOK3*
Biological Process	Oligosaccharide Metabolic Process (GO:0009311)	0.8282944	0.8830173	0	0	0.569141538	0.10721864	*FUT9*
Biological Process	Regulation Of DNA Binding (GO:0051101)	0.8282944	0.8830173	0	0	0.569141538	0.10721864	*TFAP4*
Biological Process	Positive Regulation Of Defense Response (GO:0031349)	0.8283495	0.8830173	0	0	0.662505619	0.124763112	*POLR3C;POLR3F;CD47*
Biological Process	Negative Regulation Of Protein Kinase Activity (GO:0006469)	0.8304649	0.8845604	0	0	0.621688152	0.115490733	*PDPK1;LRP6*
Biological Process	Positive Regulation Of Plasma Membrane Bounded Cell Projection Assembly (GO:0120034)	0.8304649	0.8845604	0	0	0.621688152	0.115490733	*GSK3B;CEP120*
Biological Process	Apoptotic Process (GO:0006915)	0.8335714	0.8870649	0	0	0.721402074	0.131321125	*APAF1;FEM1B;CASP3;SIAH1;EP300;RAF1*
Biological Process	Cilium Organization (GO:0044782)	0.8335714	0.8870649	0	0	0.721402074	0.131321125	*TTC26;KIF3A;TCTN2;TTC21B;RABL2B;PHIP*
Biological Process	Erythrocyte Differentiation (GO:0030218)	0.8344906	0.8870649	0	0	0.557255447	0.10082632	*CASP3*
Biological Process	Fatty Acid Beta-Oxidation (GO:0006635)	0.8344906	0.8870649	0	0	0.557255447	0.10082632	*ADIPOQ*
Biological Process	Heterophilic Cell-Cell Adhesion Via Plasma Membrane Cell Adhesion Molecules (GO:0007157)	0.8344906	0.8870649	0	0	0.557255447	0.10082632	*CD209*
Biological Process	Sodium Ion Transport (GO:0006814)	0.8351634	0.8874239	0	0	0.614510294	0.110690474	*SLC4A4;TPCN2*
Biological Process	Protein-Containing Complex Assembly (GO:0065003)	0.8390081	0.8909115	0	0	0.752390778	0.132070813	*PPP6C;TFAP4;CAND1;CUL3;REPS2;VAMP4;PPARGC1A;WASF3;NCK1*
Biological Process	Regulation Of Epithelial Cell Migration (GO:0010632)	0.8404635	0.8909115	0	0	0.545854503	0.094870438	*CLASP2*
Biological Process	Regulation Of Interleukin-12 Production (GO:0032655)	0.8404635	0.8909115	0	0	0.545854503	0.094870438	*CD47*
Biological Process	Cell Chemotaxis (GO:0060326)	0.8404635	0.8909115	0	0	0.545854503	0.094870438	*EPHB1*
Biological Process	Steroid Biosynthetic Process (GO:0006694)	0.8404635	0.8909115	0	0	0.545854503	0.094870438	*HMGCR*
Biological Process	Pyruvate Metabolic Process (GO:0006090)	0.8404635	0.8909115	0	0	0.545854503	0.094870438	*PDHA1*
Biological Process	Adenylate Cyclase-Modulating G Protein-Coupled Receptor Signaling Pathway (GO:0007188)	0.8427006	0.8929256	0	0	0.671831629	0.114979619	*ADGRB3;GNAI3;PRKACB;ADGRL3*
Biological Process	SMAD Protein Signal Transduction (GO:0060395)	0.8462212	0.8941524	0	0	0.534909597	0.089316259	*SMAD5*
Biological Process	Amide Biosynthetic Process (GO:0043604)	0.8462212	0.8941524	0	0	0.534909597	0.089316259	*GLS*
Biological Process	Regulation Of Release Of Sequestered Calcium Ion Into Cytosol (GO:0051279)	0.8462212	0.8941524	0	0	0.534909597	0.089316259	*PDPK1*
Biological Process	Sensory Perception (GO:0007600)	0.8462212	0.8941524	0	0	0.534909597	0.089316259	*OPRM1*

Biological Process	Microtubule Bundle Formation (GO:0001578)	0.8462212	0.8941524	0	0	0.534909597	0.089316259	*TTC26*
Biological Process	Negative Regulation Of Gene Expression, Epigenetic (GO:0045814)	0.8462212	0.8941524	0	0	0.534909597	0.089316259	*DNMT1*
Biological Process	Positive Regulation Of Actin Filament Bundle Assembly (GO:0032233)	0.8462212	0.8941524	0	0	0.534909597	0.089316259	*CD47*
Biological Process	Protein Targeting (GO:0006605)	0.8478461	0.8955121	0	0	0.636049678	0.104983933	*VPS13A;ZDHHC7;ZDHHC21*
Biological Process	B Cell Activation (GO:0042113)	0.8485603	0.8959093	0	0	0.593933767	0.097532289	*FNIP1;RASGRP1*
Biological Process	Regulation Of Protein Localization To Nucleus (GO:1900180)	0.8517713	0.897868	0	0	0.524393902	0.084132283	*GSK3B*
Biological Process	Sprouting Angiogenesis (GO:0002040)	0.8517713	0.897868	0	0	0.524393902	0.084132283	*MIA3*
Biological Process	Neurotransmitter Transport (GO:0006836)	0.8517713	0.897868	0	0	0.524393902	0.084132283	*LIN7C*
Biological Process	Positive Regulation Of B Cell Activation (GO:0050871)	0.8517713	0.897868	0	0	0.524393902	0.084132283	*KMT5B*
Biological Process	Regulation Of Cytokine Production (GO:0001817)	0.8558281	0.8981466	0	0	0.655207869	0.102006555	*ZBTB6;ZBTB34;CD47;LGR4*
Biological Process	Membrane Organization (GO:0061024)	0.8558281	0.8981466	0	0	0.655207869	0.102006555	*PDCD6IP;AAK1;TOR1AIP2;EXOC6*
Biological Process	Cellular Response To Hormone Stimulus (GO:0032870)	0.8569335	0.8981466	0	0	0.580961608	0.089697575	*AVPR1B;STAT3*
Biological Process	Muscle Contraction (GO:0006936)	0.8569335	0.8981466	0	0	0.580961608	0.089697575	*TPCN2;TTN*
Biological Process	Actin Cytoskeleton Reorganization (GO:0031532)	0.8571214	0.8981466	0	0	0.514282658	0.079289893	*FER*
Biological Process	Actin Polymerization Or Depolymerization (GO:0008154)	0.8571214	0.8981466	0	0	0.514282658	0.079289893	*WASF3*
Biological Process	Blood Vessel Morphogenesis (GO:0048514)	0.8571214	0.8981466	0	0	0.514282658	0.079289893	*RASA1*
Biological Process	Regulation Of Proteasomal Protein Catabolic Process (GO:0061136)	0.8571214	0.8981466	0	0	0.514282658	0.079289893	*TMTC3*
Biological Process	Regulation Of Receptor Signaling Pathway Via JAK-STAT (GO:0046425)	0.8571214	0.8981466	0	0	0.514282658	0.079289893	*EP300*
Biological Process	Spermatid Development (GO:0007286)	0.8571214	0.8981466	0	0	0.514282658	0.079289893	*ING2*
Biological Process	Glycoprotein Metabolic Process (GO:0009100)	0.8571214	0.8981466	0	0	0.514282658	0.079289893	*FBXO6*
Biological Process	Gonad Development (GO:0008406)	0.8571214	0.8981466	0	0	0.514282658	0.079289893	*SIX4*
Biological Process	Mitochondrial Transport (GO:0006839)	0.8571214	0.8981466	0	0	0.514282658	0.079289893	*SLC25A16*
Biological Process	Negative Regulation Of Viral Genome Replication (GO:0045071)	0.8571214	0.8981466	0	0	0.514282658	0.079289893	*SETDB1*
Biological Process	Proton Motive Force-Driven Mitochondrial ATP Synthesis (GO:0042776)	0.8571214	0.8981466	0	0	0.514282658	0.079289893	*NDUFC2*
Biological Process	Epithelial Cell Differentiation (GO:0030855)	0.8585968	0.8993369	0	0	0.62116052	0.094699556	*KRT37;ZNF703;ZDHHC7*
Biological Process	Cell Junction Assembly (GO:0034329)	0.8609595	0.9010557	0	0	0.574684758	0.086034779	*SPOCK2;ADGRL3*
Biological Process	Monoatomic Anion Transmembrane Transport (GO:0098656)	0.8622786	0.9010557	0	0	0.504552969	0.074763051	*SLC12A6*
Biological Process	Negative Regulation Of Neuron Projection Development (GO:0010977)	0.8622786	0.9010557	0	0	0.504552969	0.074763051	*SPOCK1*
Biological Process	Negative Regulation Of Protein Catabolic Process (GO:0042177)	0.8622786	0.9010557	0	0	0.504552969	0.074763051	*HMGCR*
Biological Process	Positive Regulation Of Leukocyte Chemotaxis (GO:0002690)	0.8622786	0.9010557	0	0	0.504552969	0.074763051	*MAPK1*
Biological Process	Regulation Of Blood Vessel Endothelial Cell Migration (GO:0043535)	0.8622786	0.9010557	0	0	0.504552969	0.074763051	*PDPK1*
Biological Process	Regulation Of Protein Metabolic Process (GO:0051246)	0.8648817	0.9034194	0	0	0.568541457	0.082530948	*NOLC1;MSI2*

Biological Process	Regulation Of Inflammatory Response (GO:0050727)	0.866871	0.9044666	0	0	0.683976155	0.097716358	*CELF1;STAT3;ADIPOQ;XIAP;SPATA2;CD47*
Biological Process	Cellular Response To Fibroblast Growth Factor Stimulus (GO:0044344)	0.86725	0.9044666	0	0	0.49518364	0.070528031	*RAB14*
Biological Process	Regulation Of Synaptic Transmission, Glutamatergic (GO:0051966)	0.86725	0.9044666	0	0	0.49518364	0.070528031	*RELN*
Biological Process	Purine Ribonucleotide Biosynthetic Process (GO:0009152)	0.86725	0.9044666	0	0	0.49518364	0.070528031	*SLC25A16*
Biological Process	Metal Ion Transport (GO:0030001)	0.8680124	0.9049055	0	0	0.639380457	0.090503833	*SLC11A2;ATP2B2;SLC4A4;KCNJ3*
Biological Process	Regulation Of Protein Kinase B Signaling (GO:0051896)	0.8718927	0.9073211	0	0	0.602353163	0.082575909	*SPRY2;PIK3CB;MTDH*
Biological Process	Regulation Of Reactive Oxygen Species Metabolic Process (GO:2000377)	0.8720421	0.9073211	0	0	0.486155013	0.066563175	*SIRT5*
Biological Process	Cellular Response To Peptide (GO:1901653)	0.8720421	0.9073211	0	0	0.486155013	0.066563175	*GSK3B*
Biological Process	Positive Regulation Of Cellular Metabolic Process (GO:0031325)	0.8720421	0.9073211	0	0	0.486155013	0.066563175	*ADIPOQ*
Biological Process	Regulation Of Cation Channel Activity (GO:2001257)	0.8720421	0.9073211	0	0	0.486155013	0.066563175	*CACNB4*
Biological Process	Positive Regulation Of Cold-Induced Thermogenesis (GO:0120162)	0.8724231	0.9073611	0	0	0.556638812	0.075970493	*ADIPOQ;PPARGC1A*
Biological Process	Positive Regulation Of MAPK Cascade (GO:0043410)	0.8733983	0.9080189	0	0	0.706079321	0.095577406	*EPGN;AVPR1B;MFAP3;SPRY2;OPRM1;RAF1;RASGRP1;CRKL*
Biological Process	Potassium Ion Transmembrane Transport (GO:0071805)	0.8750392	0.9092702	0	0	0.597826766	0.079801843	*KCND2;SLC12A6;KCNJ3*
Biological Process	Chemokine-Mediated Signaling Pathway (GO:0070098)	0.8766614	0.9092702	0	0	0.477448838	0.062848694	*CXCL13*
Biological Process	Striated Muscle Contraction (GO:0006941)	0.8766614	0.9092702	0	0	0.477448838	0.062848694	*TTN*
Biological Process	Membrane Lipid Biosynthetic Process (GO:0046467)	0.8766614	0.9092702	0	0	0.477448838	0.062848694	*ELOVL4*
Biological Process	Positive Regulation Of Interleukin-1 Beta Production (GO:0032731)	0.8766614	0.9092702	0	0	0.477448838	0.062848694	*STAT3*
Biological Process	Regulation Of Calcium Ion Transport (GO:0051924)	0.8766614	0.9092702	0	0	0.477448838	0.062848694	*CTNNB1*
Biological Process	Renal System Development (GO:0072001)	0.8811143	0.912103	0	0	0.469048142	0.059366474	*PYGO2*
Biological Process	Selective Autophagy (GO:0061912)	0.8811143	0.912103	0	0	0.469048142	0.059366474	*ATG14*
Biological Process	Glycosaminoglycan Biosynthetic Process (GO:0006024)	0.8811143	0.912103	0	0	0.469048142	0.059366474	*GLCE*
Biological Process	Positive Regulation Of Type II Interferon Production (GO:0032729)	0.8811143	0.912103	0	0	0.469048142	0.059366474	*RASGRP1*
Biological Process	Regulation Of Bone Mineralization (GO:0030500)	0.8811143	0.912103	0	0	0.469048142	0.059366474	*OSR2*
Biological Process	Extracellular Matrix Organization (GO:0030198)	0.8819899	0.9126529	0	0	0.620631415	0.077935568	*SPOCK2;COL22A1;ST7;P3H4*
Biological Process	Monoatomic Cation Transmembrane Transport (GO:0098655)	0.8832266	0.9135756	0	0	0.681002447	0.084562486	*KCND2;SLC30A5;SLC11A2;CACNA1C;SLC12A6;SLC4A4;TPCN2*
Biological Process	Aerobic Respiration (GO:0009060)	0.8854065	0.9136899	0	0	0.460937125	0.056099916	*NDUFC2*
Biological Process	Cellular Response To Chemokine (GO:1990869)	0.8854065	0.9136899	0	0	0.460937125	0.056099916	*CXCL13*
Biological Process	Chordate Embryonic Development (GO:0043009)	0.8854065	0.9136899	0	0	0.460937125	0.056099916	*SIX4*
Biological Process	Glucose Metabolic Process (GO:0006006)	0.8854065	0.9136899	0	0	0.460937125	0.056099916	*PPARGC1A*
Biological Process	Protein Targeting To Mitochondrion (GO:0006626)	0.8854065	0.9136899	0	0	0.460937125	0.056099916	*TIMM29*
Biological Process	Regulation Of Cell Death (GO:0010941)	0.8854065	0.9136899	0	0	0.460937125	0.056099916	*ZDHHC17*

Biological Process	Inorganic Cation Transmembrane Transport (GO:0098662)	0.8894836	0.916175	0	0	0.67352064	0.078878863	*KCND2;SLC30A5;SLC11A2;CACNA1C;SLC12A6;SLC4A4;TPCN2*
Biological Process	Sterol Metabolic Process (GO:0016125)	0.889544	0.916175	0	0	0.453101058	0.053033792	*HMGCR*
Biological Process	Homophilic Cell Adhesion Via Plasma Membrane Adhesion Molecules (GO:0007156)	0.889544	0.916175	0	0	0.453101058	0.053033792	*CNTN4*
Biological Process	Positive Regulation Of Phagocytosis (GO:0050766)	0.889544	0.916175	0	0	0.453101058	0.053033792	*CD47*
Biological Process	Proton Motive Force-Driven ATP Synthesis (GO:0015986)	0.889544	0.916175	0	0	0.453101058	0.053033792	*NDUFC2*
Biological Process	Fatty Acid Catabolic Process (GO:0009062)	0.8935323	0.9185454	0	0	0.445526194	0.050154108	*ADIPOQ*
Biological Process	Negative Regulation Of Intrinsic Apoptotic Signaling Pathway (GO:2001243)	0.8935323	0.9185454	0	0	0.445526194	0.050154108	*DDX3X*
Biological Process	Negative Regulation Of Viral Process (GO:0048525)	0.8935323	0.9185454	0	0	0.445526194	0.050154108	*MID2*
Biological Process	Positive Regulation Of Cell Junction Assembly (GO:1901890)	0.8935323	0.9185454	0	0	0.445526194	0.050154108	*EPHB1*
Biological Process	Positive Regulation Of Cytokine Production (GO:0001819)	0.8935792	0.9185454	0	0	0.683088447	0.076861285	*DDX3X;TRAF6;STAT3;ADIPOQ;XIAP;BCL10;RASGRP1;APPL1*
Biological Process	Glycerophospholipid Metabolic Process (GO:0006650)	0.8973768	0.9220912	0	0	0.438199685	0.047447998	*GDPD1*
Biological Process	Macromolecule Biosynthetic Process (GO:0009059)	0.8993887	0.9238002	0	0	0.596142442	0.063214932	*EEF2K;FUT9;MRPS11;MRRF*
Biological Process	Inorganic Anion Transmembrane Transport (GO:0098661)	0.9010827	0.9241069	0	0	0.431109516	0.044903612	*SLC12A6*
Biological Process	Oxidative Phosphorylation (GO:0006119)	0.9010827	0.9241069	0	0	0.431109516	0.044903612	*NDUFC2*
Biological Process	Phosphatidylinositol Metabolic Process (GO:0046488)	0.9010827	0.9241069	0	0	0.431109516	0.044903612	*LCLAT1*
Biological Process	Protein Polymerization (GO:0051258)	0.9010827	0.9241069	0	0	0.431109516	0.044903612	*WASF3*
Biological Process	Positive Regulation Of Metabolic Process (GO:0009893)	0.9046311	0.9266941	0	0	0.503862932	0.050501218	*ADIPOQ;PPARGC1A*
Biological Process	Cellular Response To Virus (GO:0098586)	0.9046549	0.9266941	0	0	0.424244431	0.042510028	*DDX3X*
Biological Process	Positive Regulation Of Interleukin-1 Production (GO:0032732)	0.9046549	0.9266941	0	0	0.424244431	0.042510028	*STAT3*
Biological Process	Antimicrobial Humoral Immune Response Mediated By Antimicrobial Peptide (GO:0061844)	0.9080983	0.9291434	0	0	0.41759388	0.040257168	*CXCL13*
Biological Process	Fatty Acid Biosynthetic Process (GO:0006633)	0.9080983	0.9291434	0	0	0.41759388	0.040257168	*ELOVL4*
Biological Process	Monocarboxylic Acid Biosynthetic Process (GO:0072330)	0.9080983	0.9291434	0	0	0.41759388	0.040257168	*ELOVL4*
Biological Process	Adenylate Cyclase-Activating G Protein-Coupled Receptor Signaling Pathway (GO:0007189)	0.9100864	0.9308181	0	0	0.494480553	0.046587859	*ADGRB3;ADGRL3*
Biological Process	Cellular Response To Type II Interferon (GO:0071346)	0.9114175	0.9318197	0	0	0.411147962	0.038135721	*CD47*
Biological Process	Negative Regulation Of Inflammatory Response (GO:0050728)	0.9127032	0.9327742	0	0	0.489918479	0.044751397	*ADIPOQ;STAT3*
Biological Process	Cholesterol Metabolic Process (GO:0008203)	0.9146169	0.9343695	0	0	0.404897374	0.03613708	*HMGCR*
Biological Process	Aerobic Electron Transport Chain (GO:0019646)	0.917701	0.9367976	0	0	0.398833371	0.03425328	*NDUFC2*
Biological Process	Neuropeptide Signaling Pathway (GO:0007218)	0.917701	0.9367976	0	0	0.398833371	0.03425328	*OPRM1*
Biological Process	DNA-templated DNA Replication (GO:0006261)	0.9206738	0.938747	0	0	0.392947722	0.03247694	*ORC1*
Biological Process	Phospholipid Metabolic Process (GO:0006644)	0.9206738	0.938747	0	0	0.392947722	0.03247694	*PLAA*

Biological Process	Positive Regulation Of Phosphatidylinositol 3-Kinase Signaling (GO:0014068)	0.9206738	0.938747	0	0	0.392947722	0.03247694	*CBL*
Biological Process	Sulfur Compound Biosynthetic Process (GO:0044272)	0.9235393	0.9402212	0	0	0.38723267	0.03080122	*GLCE*
Biological Process	Mitochondrial ATP Synthesis Coupled Electron Transport (GO:0042775)	0.9235393	0.9402212	0	0	0.38723267	0.03080122	*NDUFC2*
Biological Process	Neutrophil Chemotaxis (GO:0030593)	0.9235393	0.9402212	0	0	0.38723267	0.03080122	*CXCL13*
Biological Process	Positive Regulation Of Protein Transport (GO:0051222)	0.9235393	0.9402212	0	0	0.38723267	0.03080122	*TTN*
Biological Process	Protein Processing (GO:0016485)	0.9269616	0.9430701	0	0	0.464212184	0.035207293	*TMPRSS4;SPPL3*
Biological Process	Translation (GO:0006412)	0.9270497	0.9430701	0	0	0.581763215	0.044067462	*MRPL2;MRPS27;EEF2K;MRPS11;MRRF*
Biological Process	Positive Regulation Of Protein Localization To Nucleus (GO:1900182)	0.928964	0.944444	0	0	0.37628553	0.027726695	*EP300*
Biological Process	Ribonucleoprotein Complex Biogenesis (GO:0022613)	0.9291132	0.944444	0	0	0.460186341	0.033835037	*RIOK2;RPF2*
Biological Process	Granulocyte Chemotaxis (GO:0071621)	0.9315305	0.946175	0	0	0.371040025	0.026316515	*CXCL13*
Biological Process	Monocarboxylic Acid Transport (GO:0015718)	0.9315305	0.946175	0	0	0.371040025	0.026316515	*ARID4A*
Biological Process	Chloride Transport (GO:0006821)	0.9409031	0.9542313	0	0	0.351438401	0.021407918	*SLC12A6*
Biological Process	Neutrophil Migration (GO:1990266)	0.9409031	0.9542313	0	0	0.351438401	0.021407918	*CXCL13*
Biological Process	Positive Regulation Of Endothelial Cell Proliferation (GO:0001938)	0.9409031	0.9542313	0	0	0.351438401	0.021407918	*PDPK1*
Biological Process	Positive Regulation Of Synaptic Transmission (GO:0050806)	0.9409031	0.9542313	0	0	0.351438401	0.021407918	*RELN*
Biological Process	Positive Regulation Of Intracellular Protein Transport (GO:0090316)	0.9442909	0.9573007	0	0	0.430317189	0.024666199	*GSK3B;EP300*
Biological Process	Calcium Ion Transmembrane Transport (GO:0070588)	0.9470815	0.9597624	0	0	0.338039823	0.018379254	*CACNA1C*
Biological Process	Spindle Assembly (GO:0051225)	0.9489942	0.9605982	0	0	0.33379694	0.017475142	*CLASP2*
Biological Process	Macromolecule Modification (GO:0043412)	0.9489942	0.9605982	0	0	0.33379694	0.017475142	*PRKDC*
Biological Process	Positive Regulation Of Growth (GO:0045927)	0.9489942	0.9605982	0	0	0.33379694	0.017475142	*DDX3X*
Biological Process	Regulation Of Interleukin-1 Beta Production (GO:0032651)	0.9543277	0.9652593	0	0	0.32168172	0.015038022	*STAT3*
Biological Process	Purine Ribonucleotide Metabolic Process (GO:0009150)	0.9543277	0.9652593	0	0	0.32168172	0.015038022	*GNAI3*
Biological Process	Regulation Of Endothelial Cell Migration (GO:0010594)	0.9605828	0.9712152	0	0	0.30682781	0.012339103	*PIK3CB*
Biological Process	Sensory Perception Of Mechanical Stimulus (GO:0050954)	0.962008	0.9719143	0	0	0.303325326	0.011748542	*ATP2B2*
Biological Process	Organic Anion Transport (GO:0015711)	0.962008	0.9719143	0	0	0.303325326	0.011748542	*SLC4A4*
Biological Process	Intracellular Calcium Ion Homeostasis (GO:0006874)	0.9625641	0.9721053	0	0	0.389219852	0.014850544	*ATP2B2;TPCN2*
Biological Process	Regulation Of Peptidyl-Tyrosine Phosphorylation (GO:0050730)	0.9633818	0.9721898	0	0	0.299901549	0.011187978	*RELN*
Biological Process	Regulation Of Phosphatidylinositol 3-Kinase Signaling (GO:0014066)	0.9633818	0.9721898	0	0	0.299901549	0.011187978	*CBL*
Biological Process	Sensory Perception Of Sound (GO:0007605)	0.9659822	0.9744428	0	0	0.293279738	0.010150367	*ATP2B2*
Biological Process	Sphingolipid Metabolic Process (GO:0006665)	0.9683982	0.9765081	0	0	0.286942737	0.009214273	*ELOVL4*
Biological Process	Monocarboxylic Acid Metabolic Process (GO:0032787)	0.9717047	0.9794695	0	0	0.277932313	0.007977577	*THEM4*
Biological Process	Inflammatory Response (GO:0006954)	0.9725019	0.9799002	0	0	0.458678482	0.012789443	*DHX8;STAT3;CXCL13;HNRNPA0*
Biological Process	Antimicrobial Humoral Response (GO:0019730)	0.9746657	0.981707	0	0	0.269467976	0.00691476	*CXCL13*

Biological Process	Visual Perception (GO:0007601)	0.9755822	0.9822566	0	0	0.266759388	0.006594524	*EYA3*
Biological Process	Calcium Ion Homeostasis (GO:0055074)	0.9764656	0.9823993	0	0	0.264104435	0.006289846	*TPCN2*
Biological Process	Sensory Perception Of Light Stimulus (GO:0050953)	0.9764656	0.9823993	0	0	0.264104435	0.006289846	*EYA3*
Biological Process	Positive Regulation Of Inflammatory Response (GO:0050729)	0.9781378	0.9833348	0	0	0.258949188	0.005723992	*CD47*
Biological Process	Positive Regulation Of Kinase Activity (GO:0033674)	0.9781378	0.9833348	0	0	0.258949188	0.005723992	*RELN*
Biological Process	Peptide Biosynthetic Process (GO:0043043)	0.9792974	0.984127	0	0	0.341475609	0.007143627	*MRPS11;MRRF*
Biological Process	Monoatomic Ion Transport (GO:0006811)	0.9804263	0.9848878	0	0	0.251581074	0.004973208	*SLC12A6*
Biological Process	Lipid Transport (GO:0006869)	0.9811346	0.9852258	0	0	0.249216851	0.004746479	*ARID4A*
Biological Process	Sensory Perception Of Chemical Stimulus (GO:0007606)	0.9824755	0.9861984	0	0	0.244618545	0.004324832	*OR5B2*
Biological Process	Cellular Response To Tumor Necrosis Factor (GO:0071356)	0.9874244	0.9907907	0	0	0.225855119	0.002858263	*TRAF6*
Biological Process	Negative Regulation Of Cytokine Production (GO:0001818)	0.9890363	0.9917701	0	0	0.302355027	0.003333236	*CUL3;LGR4*
Biological Process	Organonitrogen Compound Biosynthetic Process (GO:1901566)	0.9891493	0.9917701	0	0	0.218404432	0.002382782	*ELOVL4*
Biological Process	Enzyme-Linked Receptor Protein Signaling Pathway (GO:0007167)	0.9895423	0.9917887	0	0	0.216617479	0.00227725	*PIK3CB*
Biological Process	Positive Regulation Of Peptidyl-Tyrosine Phosphorylation (GO:0050731)	0.9916188	0.993494	0	0	0.206477558	0.001737821	*RELN*
Biological Process	Defense Response To Bacterium (GO:0042742)	0.9952733	0.9965957	0	0	0.263079511	0.001246439	*CXCL13;MICA*
Biological Process	Defense Response To Symbiont (GO:0140546)	0.9956869	0.9965957	0	0	0.181024287	7.82E-04	*MICA*
Biological Process	Negative Regulation Of DNA-binding Transcription Factor Activity (GO:0043433)	0.9958432	0.9965957	0	0	0.179791753	7.49E-04	*BTRC*
Biological Process	Epithelium Development (GO:0060429)	0.9965439	0.9969203	0	0	0.173870754	6.02E-04	*KRT37*
Biological Process	Sensory Perception Of Smell (GO:0007608)	0.9997898	0.9997898	0	0	0.11570534	2.43E-05	*OR5B2*
Molecular Function	mRNA Binding (GO:0003729)	3.64E-08	1.84E-05	0	0	3.41102354	58.42895857	*FYTTD1;KHDRBS1;RBM25;DDX3X;CELF1;MRPS11;SRSF1;CELF4;HNRNPU;MSI2;HNRNPLL;ZC3H12B;DHX36;YTHDF1;RBFOX2;MYEF2;CPSF6;SSB;YTHDF3;CIRBP;PUM1;SUPT5H;PUM2;PTBP3;ZNF638;HNRNPA2B1;CPEB3;DCP1A;TARDBP;LSM14B;RBMX*
Molecular Function	DNA Binding (GO:0003677)	8.69E-06	0.0012175	0	0	1.973921	23.00243197	*KDM5B;WDR48;DDX3X;ZNF292;HP1BP3;CHD6;HNRNPU;ARID4A;PRDM2;SMC6;YY1;ING2;ZMYM3;ZNF84;SMARCAD1;EP300;MEF2A;HES7;OSR2;KLF12;KDM2B;DNMT3A;ATRX;ARID1A;TSN;XRN1;KAT6A;ZNF638;CLOCK;CGGBP1;KHDRBS1;GTF2A1;DNMT1;GTF3C4;PRKDC;MAX;LIN9;NR2C2;SBNO1;DHX36;PLAGL2;PPARGC1A;ZNF268;PMS1;MBD5;SSB;POU2F1;EGR4;STAT3;TFAP4;NFIB;POLR3C;ERCC4;SP4;POLR3F;TARDBP*
Molecular Function	Ribosome Binding (GO:0043022)	1.04E-05	0.0012175	0	0	4.905390539	56.29489799	*NOMO3;YTHDF1;DDX3X;MRPS27;CPSF6;YTHDF3;SEC61A2;ERI1;NAA15;CPEB3;PELO;EIF1B;MRRF*
Molecular Function	RNA Binding (GO:0003723)	1.11E-05	0.0012175	0	0	1.736028456	19.80315963	*RBM27;PUS10;RBM25;DDX3X;CELF1;MRPS11;CELF4;HNRNPU;PSIP1;MSI2;CRKL;AFF2;YY1;MRPL2;ZC3H12B;SUMO2;TNPO1;SUPT16H;MBNL2;RBFOX2;MRPS27;ZFR;CIRBP;DDX52;TSN;PTBP3;XRN1;SARNP;ZNF638;SRSF2;SREK1;EIF4E2;EIF1B;SRSF8;DCP2;FYTTD1;KHDRBS1;VCP;NUFIP2;DHX8;PRKDC;SRSF1;DDX20;NOLC1;IBA57;HNRNPLL;RPF2;MTDH;NXF1;U2AF2;ERI1;DHX36;TRA2A;RPP14;PPARGC1A;SRSF10;HNRNPA0;YTHDF1;SSB;MYEF2;CPSF6;YTHDF3;API5;STAT3;IMMT;MYO5A;PUM1;SUPT5H;U2SURP;PUM2;SON;AGO3;HNRNPA2B1;PKN2;NAA15;CPEB3;DCP1A;TARDBP;LSM14B;RBMX;TNRC6A;TNRC6B*
Molecular Function	DNA-binding Transcription Factor Binding (GO:0140297)	1.20E-05	0.0012175	0	0	2.784041787	31.54328016	*ATF2;GSK3B;PRKDC;MAX;ZMYND8;DDX20;CRKL;MTDH;YY1;HEY1;EP300;PPARGC1A;MEF2D;RUNX1T1;GTF2I;MEF2A;RBFOX2;PBX2P1;STAT3;DNMT3A;PIAS2;HCFC1;CREB1;KAT6A;CTNNB1;CGGBP1*
Molecular Function	Ubiquitin-Protein Transferase Activity (GO:0004842)	1.69E-05	0.0014271	0	0	2.395536404	26.32429399	*RNFT2;UBE2D4;CUL3;CUL2;FBXO25;RNF8;XIAP;UBE3A;UBE3B;CBL;IRF2BPL;HERC3;TRIM7;C18ORF25;ZNRF3;RNF217;FBXO6;ARIH1;BTRC;LONRF2;RNF111;TRIM23;RNF44;FBXW11;SMURF1;SIAH1;MSL2;UBE2W;RNF149;TRAF6;FBXL3;TRIP12;FBXL5*
Molecular Function	Protein Kinase B Binding (GO:0043422)	5.34E-05	0.0038713	0	0	35.89757914	353.1165259	*TRAF6;PDE3B;BCL10;APPL1*
Molecular Function	DNA-binding Transcription Activator Activity, RNA Polymerase II-specific (GO:0001228)	6.69E-05	0.0042391	0	0	2.397398844	23.04493672	*ATF2;NFAT5;GMEB1;PRDM2;NR2C2;YY1;PLAGL2;ZNF544;ZNF268;SOX4;GTF2I;MEF2A;ZNF484;OSR2;ZNF384;PBX2P1;POU2F1;EGR4;STAT3;FOXJ2;ETV1;ZFX;TFAP4;CREB1;NFIB;NFIC;ZNF780B;CLOCK*
Molecular Function	Ubiquitin-Like Protein Ligase Activity (GO:0061659)	9.77E-05	0.005501	0	0	2.427742424	22.41805074	*RNFT2;CUL3;RNF8;XIAP;UBE3A;UBE3B;CBL;IRF2BPL;HERC3;TRIM7;C18ORF25;ZNRF3;RNF217;FBXO6;ARIH1;LONRF2;RNF111;TRIM23;RNF44;SMURF1;SIAH1;MSL2;PIAS2;RNF149;TRAF6;TRIP12*

Molecular Function	Ubiquitin Protein Ligase Activity (GO:0061630)	1.64E-04	0.0076725	0	0	2.388941993	20.82298647	*RNFT2;CUL3;RNF8;XIAP;UBE3A;UBE3B;CBL;IRF2BPL;HERC3;TRIM7;C18ORF25;ZNRF3;RNF217;FBXO6;ARIH1;LONRF2;RNF111;TRIM23;RNF44;SMURF1;SIAH1;MSL2;RNF149;TRAF6;TRIP12*
Molecular Function	Ubiquitin-Like Protein Transferase Activity (GO:0019787)	1.69E-04	0.0076725	0	0	2.614831364	22.70703965	*UBE2D4;FBXW11;SMURF1;CUL3;SIAH1;CUL2;FBXO25;RNF8;XIAP;UBE3A;CBL;PIAS2;UBE2W;ZNRF3;TRAF6;RNF217;FBXL3;ARIH1;BTRC;FBXL5;TRIM23*
Molecular Function	Histone H3K36 Demethylase Activity (GO:0051864)	1.82E-04	0.0076725	0	0	21.53631285	185.5076579	*KDM4B;KDM2B;KDM7A;PHF8*
Molecular Function	Acyltransferase Activity, Transferring Groups Other Than Amino-Acyl Groups (GO:0016747)	4.52E-04	0.0176397	0	0	4.424626007	34.07477404	*KAT6A;DBT;EP300;ZDHHC13;ZDHHC7;ZDHHC21;NAA15;LCLAT1;ZDHHC17*
Molecular Function	Histone Demethylase Activity (GO:0032452)	4.98E-04	0.0180448	0	0	7.035805627	53.50272144	*KDM5B;KDM4B;KDM2B;JARID2;KDM7A;PHF8*
Molecular Function	Double-Stranded DNA Binding (GO:0003690)	7.24E-04	0.0244614	0	0	1.800385728	13.0188049	*ATF2;NFAT5;KDM5B;GMEB1;WDR48;PRKDC;MAX;HNRNPU;PSIP1;FOXK1;NR2C2;YY1;HEY1;SIX4;HIVEP1;MEF2D;SOX4;HES7;OSR2;ZNF384;KLF12;MBNL2;ZNF460;ZFP1;EGR4;TFAP2E;FOXJ2;ETV1;SMAD5;TFAP4;CREB1;RFX7;NFIB;NFIC;ZNF638;POLR3F;TARDBP;CLOCK;CGGBP1;ZNF454*
Molecular Function	Ubiquitin Protein Ligase Binding (GO:0031625)	8.54E-04	0.027068	0	0	2.286866953	16.15746301	*USP13;GSK3B;VCP;UBE2D4;CUL3;CUL2;RNF8;SMC6;BCL10;SMAD5;ATXN3;UBE2W;PRKAR2A;JKAMP;DBT;SUMO2;CTNNB1;ARIH1;EIF4E2;PRKACB;PPARGC1A*
Molecular Function	Acetylation-Dependent Protein Binding (GO:0140033)	0.0011693	0.032935	0	0	7.483294483	50.52240969	*BRD3;ZMYND8;PSME4;PHIP;MLLT3*
Molecular Function	Lysine-Acetylated Histone Binding (GO:0070577)	0.0011693	0.032935	0	0	7.483294483	50.52240969	*BRD3;ZMYND8;PSME4;PHIP;MLLT3*
Molecular Function	RNA Polymerase II-specific DNA-binding Transcription Factor Binding (GO:0061629)	0.0015811	0.0421913	0	0	2.328449328	15.01759606	*MEF2A;ATF2;GSK3B;PRKDC;STAT3;DNMT3A;BCL10;ARID1A;PIAS2;CRKL;MTDH;CREB1;HEY1;CTNNB1;EP300;PPARGC1A;MEF2D;GTF2I*
Molecular Function	mRNA 3'-UTR Binding (GO:0003730)	0.0018814	0.0459015	0	0	3.257593755	20.44385598	*CELF1;DHX36;HNRNPA2B1;CIRBP;HNRNPU;PUM1;CPEB3;TARDBP;HNRNPA0;PUM2*
Molecular Function	Ubiquitin-Like Protein Ligase Binding (GO:0044389)	0.0019012	0.0459015	0	0	2.131253603	13.35282849	*USP13;GSK3B;VCP;UBE2D4;CUL3;CUL2;RNF8;SMC6;BCL10;SMAD5;ATXN3;UBE2W;PRKAR2A;JKAMP;DBT;SUMO2;CTNNB1;ARIH1;EIF4E2;PRKACB;PPARGC1A*
Molecular Function	Cis-Regulatory Region Sequence-Specific DNA Binding (GO:0000987)	0.0022391	0.0516017	0	0	1.53660237	9.375840688	*ATF2;GMEB1;ZNF570;ELL;ZNF492;HNRNPU;YY1;HEY1;SIX4;ZNF84;SOX4;MEF2A;HES7;ZNF484;ZNF682;KLF12;ZFP1;DNMT3A;ETV1;ZFX;CREB1;RFX7;ZNF714;ZNF780B;ZNF780A;ZNF236;CLOCK;NFAT5;ZNF670;FOXK1;NR2C2;NFIL3;DHX36;HIVEP1;ZNF623;HIVEP3;ZNF544;ZNF268;MEF2D;ZNF264;ZNF384;ZNF460;POU2F1;PBX2P1;EGR4;ZNF260;STAT3;FOXJ2;SMAD5;TFAP4;ZNF37A;NFIB;NFIC;SP4;ZNF736;RBAK;RBMX;ZNF454*
Molecular Function	Sequence-Specific DNA Binding (GO:0043565)	0.0024714	0.0526203	0	0	1.661780725	9.975632342	*ATF2;NFAT5;KDM5B;GMEB1;MAX;HNRNPU;FOXK1;PRDM2;NR2C2;YY1;HEY1;SIX4;HIVEP1;PLAGL2;PPARGC1A;MEF2D;SOX4;HES7;MEF2A;OSR2;ZNF384;KLF12;MBNL2;ZNF460;POU2F1;ZFP1;EGR4;KDM2B;TFAP2E;FOXJ2;ETV1;SMAD5;TFAP4;CREB1;RFX7;NFIB;NFIC;SP4;TERF2IP;CLOCK;ZNF454*
Molecular Function	Histone Acetyltransferase Activity (GO:0004402)	0.0024909	0.0526203	0	0	6.121424031	36.69860875	*ATF2;ING3;KAT6A;EP300;CLOCK*
Molecular Function	RNA Polymerase II Transcription Regulatory Region Sequence-Specific DNA Binding (GO:0000977)	0.0027438	0.0554062	0	0	1.49513121	8.81887845	*ATF2;GMEB1;ZNF570;ZNF492;HNRNPU;PRDM2;YY1;HEY1;SIX4;ZNF84;SOX4;MEF2A;HES7;ZNF484;ZNF682;OSR2;KLF12;ZFP1;DNMT3A;ETV1;ZNF14;ZFX;CREB1;RFX7;ZNF714;ZNF780B;ZNF780A;ZNF236;CLOCK;GTF2A1;NFAT5;ZNF670;MAX;FOXK1;NR2C2;NFIL3;DHX36;HIVEP1;ZNF623;HIVEP3;ZNF544;ZNF268;MEF2D;ZNF264;ZNF384;ZNF460;POU2F1;PBX2P1;EGR4;TFAP2E;ZNF260;STAT3;FOXJ2;SMAD5;TFAP4;ZNF37A;NFIB;NFIC;SP4;ZNF736;RBAK;RBMX;ZNF454*
Molecular Function	Ubiquitin Conjugating Enzyme Binding (GO:0031624)	0.0029438	0.0554062	0	0	5.854971116	34.12304084	*DCUN1D5;TRAF6;RNF217;SIAH1;ARIH1*
Molecular Function	Chromatin DNA Binding (GO:0031490)	0.0029506	0.0554062	0	0	3.285323298	19.13943039	*SBNO1;RBBP4;ATRX;STAT3;HNRNPU;EP300;ZFX;CLOCK;PPARGC1A*
Molecular Function	2-Oxoglutarate-Dependent Dioxygenase Activity (GO:0016706)	0.0032717	0.0579879	0	0	4.620648259	26.44143589	*KDM5B;KDM4B;KDM2B;JARID2;KDM7A;PHF8*
Molecular Function	Protein Serine Kinase Activity (GO:0106310)	0.0033169	0.0579879	0	0	13.44072524	76.72954597	*GSK3B;DYRK1A;PIK3CB*
Molecular Function	RNA Polymerase II Cis-Regulatory Region Sequence-Specific DNA Binding (GO:0000978)	0.003581	0.0605194	0	0	1.499965927	8.447966599	*ATF2;GMEB1;ZNF570;ZNF492;HNRNPU;YY1;HEY1;SIX4;ZNF84;SOX4;MEF2A;HES7;ZNF484;ZNF682;KLF12;ZFP1;DNMT3A;ETV1;ZFX;CREB1;RFX7;ZNF714;ZNF780B;ZNF780A;ZNF236;CLOCK;NFAT5;ZNF670;MAX;FOXK1;NR2C2;NFIL3;DHX36;HIVEP1;ZNF623;HIVEP3;ZNF544;ZNF268;MEF2D;ZNF264;ZNF384;ZNF460;POU2F1;PBX2P1;EGR4;ZNF260;STAT3;FOXJ2;SMAD5;TFAP4;ZNF37A;NFIB;NFIC;SP4;ZNF736;RBAK;RBMX;ZNF454*
Molecular Function	RNA Exonuclease Activity, Producing 5'-Phosphomonoesters (GO:0016896)	0.0040232	0.063742	0	0	5.386013986	29.70756616	*XRN1;ERI1;DIS3;ERI2;DCP2*
Molecular Function	pre-mRNA Binding (GO:0036002)	0.0040232	0.063742	0	0	5.386013986	29.70756616	*PRPF39;ERI1;U2AF2;CELF4;HNRNPU*
Molecular Function	N6-methyladenosine-containing RNA Binding (GO:1990247)	0.0046124	0.0649574	0	0	11.52002391	61.9663821	*YTHDF1;YTHDF3;HNRNPA2B1*
Molecular Function	Histone H4K12 Acetyltransferase Activity (GO:0043997)	0.0046124	0.0649574	0	0	11.52002391	61.9663821	*ING3;BRPF3;KAT6A*
Molecular Function	Histone H4K5 Acetyltransferase Activity (GO:0043995)	0.0046124	0.0649574	0	0	11.52002391	61.9663821	*ING3;BRPF3;KAT6A*
Molecular Function	Histone H4K8 Acetyltransferase Activity (GO:0043996)	0.0046124	0.0649574	0	0	11.52002391	61.9663821	*ING3;BRPF3;KAT6A*

Molecular Function	Tau-Protein Kinase Activity (GO:0050321)	0.0050925	0.067944	0	0	6.726256983	35.51460864	*GSK3B;PRKAA1;DYRK1A;MARK3*
Molecular Function	Histone H4 Acetyltransferase Activity (GO:0010485)	0.0050925	0.067944	0	0	6.726256983	35.51460864	*ING3;BRPF3;KAT6A;EP300*
Molecular Function	Cysteine-Type Peptidase Activity (GO:0008234)	0.0059182	0.0758318	0	0	2.45865434	12.6122161	*USP13;USP8;ATXN3;USP37;USP15;USP9X;CASP3;USP9Y;UFSP2;JOSD1;OTUD1;SENP1*
Molecular Function	Telomerase RNA Binding (GO:0070034)	0.0061141	0.0758318	0	0	6.330266185	32.26638704	*XRN1;DHX36;HNRNPU;DCP2*
Molecular Function	RNA Polymerase Core Enzyme Binding (GO:0043175)	0.0061324	0.0758318	0	0	4.808191808	24.49377469	*RRN3;WAC;HNRNPU;RPRD1A;RPRD2*
Molecular Function	Sequence-Specific Double-Stranded DNA Binding (GO:1990837)	0.0067717	0.0817439	0	0	1.576075906	7.872507583	*ATF2;NFAT5;KDM5B;GMEB1;MAX;HNRNPU;ARID4A;FOXK1;ARID4B;NR2C2;YY1;HEY1;ORC1;SIX4;DHX36;HIVEP1;MEF2D;SOX4;HES7;OSR2;ZNF384;KLF12;MBNL2;ZNF460;ZFP1;EGR4;TFAP2E;STAT3;FOXJ2;ETV1;SMAD5;TFAP4;CREB1;RFX7;NFIB;TBL1XR1;NFIC;CLOCK;ZNF454*
Molecular Function	Ubiquitin-Like Protein Conjugating Enzyme Binding (GO:0044390)	0.0069809	0.0823097	0	0	4.642150952	23.04631936	*DCUN1D5;TRAF6;RNF217;SIAH1;ARIH1*
Molecular Function	Histone H3K36 Methyltransferase Activity (GO:0046975)	0.0080135	0.0923368	0	0	8.959088796	43.24223806	*NSD3;SETD5;NSD2*
Molecular Function	Basal RNA Polymerase II Transcription Machinery Binding (GO:0001099)	0.0089175	0.0982867	0	0	4.342206181	20.49406806	*GTF2A1;WAC;HNRNPU;RPRD1A;RPRD2*
Molecular Function	Histone H3 Methyltransferase Activity (GO:0140938)	0.0089175	0.0982867	0	0	4.342206181	20.49406806	*NSD3;SETD5;SETDB1;NSD2;JARID2*
Molecular Function	Histone Deacetylase Binding (GO:0042826)	0.0093818	0.101204	0	0	2.699015471	12.60165164	*MEF2A;TFAP4;RBBP4;MIER3;TRAF6;DHX36;LCOR;PKN2;MEF2D*
Molecular Function	I-SMAD Binding (GO:0070411)	0.010142	0.1028401	0	0	8.062761506	37.01668852	*SMURF1;CTNNB1;SMAD5*
Molecular Function	Histone H3 Acetyltransferase Activity (GO:0010484)	0.010142	0.1028401	0	0	8.062761506	37.01668852	*BRPF3;KAT6A;EP300*
Molecular Function	Histone H3K9 Demethylase Activity (GO:0032454)	0.010142	0.1028401	0	0	8.062761506	37.01668852	*KDM4B;KDM7A;PHF8*
Molecular Function	Methylation-Dependent Protein Binding (GO:0140034)	0.0110178	0.109386	0	0	3.093210402	13.94494178	*MBTD1;ING2;ING3;ZMYND8;WDR5;KDM7A;PHF8*
Molecular Function	GTPase Regulator Activity (GO:0030695)	0.0112191	0.109386	0	0	1.702187122	7.643058253	*DENND1B;ITSN1;ARHGAP39;ARHGAP5;RASGRP1;TBC1D30;FNIP1;EVI5;SEC23B;RALGPS2;RGS7;ARFGEF2;ARHGEF12;DIS3;ELMOD2;RALGAPA2;DENND4C;ARHGAP29;TBC1D1;ADGRB3;RASA1;RAB3GAP2;SOS1;ARHGEF7;RAPGEF6*
Molecular Function	Poly-Pyrimidine Tract Binding (GO:0008187)	0.0115362	0.1103554	0	0	5.123437084	22.8621493	*KHDRBS1;SSB;U2AF2;HNRNPU*
Molecular Function	Zinc Ion Binding (GO:0008270)	0.0122415	0.114924	0	0	1.780042918	7.837391639	*KDM5B;KDM2B;ZMYND8;SIAH1;ERAP1;RNF8;SIRT5;LNPEP;PRDM2;IDE;BAZ1B;RASGRP1;PHF8;SLC25A16;ZNF84;KAT6A;PHF14;ARIH1;RBAK;ZFP37;KDM7A*
Molecular Function	Tau Protein Binding (GO:0048156)	0.0124716	0.114924	0	0	3.958453311	17.35505665	*GSK3B;PRKAA1;DYRK1A;EP300;MARK3*
Molecular Function	Protein-Lysine N-methyltransferase Activity (GO:0016279)	0.0126938	0.114924	0	0	3.366946779	14.70225673	*NSD3;SETD5;SETDB1;NSD2;KMT5B;JARID2*
Molecular Function	NF-kappaB Binding (GO:0051059)	0.0132535	0.1178866	0	0	4.890299644	21.14317146	*GSK3B;EP300;BCL10;MTDH*
Molecular Function	Methylated Histone Binding (GO:0035064)	0.0137899	0.1205426	0	0	2.947755961	12.62765596	*MBTD1;ING2;ING3;ZMYND8;WDR5;KDM7A;PHF8*
Molecular Function	Acetyltransferase Activity (GO:0016407)	0.0151253	0.1230916	0	0	4.677435026	19.60494242	*KAT6A;DBT;EP300;NAA15*
Molecular Function	Palmitoyltransferase Activity (GO:0016409)	0.0151253	0.1230916	0	0	4.677435026	19.60494242	*ZDHHC13;ZDHHC21;ZDHHC7;ZDHHC17*
Molecular Function	DNA Nuclease Activity (GO:0004536)	0.0152954	0.1230916	0	0	6.718270572	28.08373375	*DFFB;ERCC4;N4BP2*
Molecular Function	K48-linked Deubiquitinase Activity (GO:1990380)	0.0152954	0.1230916	0	0	6.718270572	28.08373375	*USP13;USP15;USP9X*
Molecular Function	RNA Polymerase III Activity (GO:0001056)	0.0152954	0.1230916	0	0	6.718270572	28.08373375	*POLR3C;POLR1D;POLR3F*
Molecular Function	G-quadruplex RNA Binding (GO:0002151)	0.0176286	0.1375032	0	0	13.42339833	54.20678961	*XRN1;DHX36*
Molecular Function	Primary miRNA Binding (GO:0070878)	0.0176286	0.1375032	0	0	13.42339833	54.20678961	*PUS10;STAT3*
Molecular Function	Ubiquitin-Dependent Protein Binding (GO:0140036)	0.0183302	0.1403718	0	0	6.201158674	24.79969825	*USP15;VCP;IDE*
Molecular Function	Single-Stranded RNA Binding (GO:0003727)	0.0185501	0.1403718	0	0	3.541037909	14.11910515	*DDX3X;AGO3;U2AF2;ZFR;HNRNPU*
Molecular Function	miRNA Binding (GO:0035198)	0.0193479	0.1442556	0	0	4.302793296	16.9752588	*AGO3;HNRNPA2B1;PUM1;PUM2*
Molecular Function	Phosphatase Binding (GO:0019902)	0.019823	0.145656	0	0	2.356765393	9.240669209	*VCP;CHCHD3;ELL;KIF3A;STAT3;MTMR9;TERF2IP;MAPK1;CTNNB1*
Molecular Function	Ubiquitin Ligase-Substrate Adaptor Activity (GO:1990756)	0.0203271	0.1472261	0	0	3.450062758	13.44076044	*KLHDC2;APPBP2;FEM1B;FEM1C;BTRC*
Molecular Function	Deubiquitinase Activity (GO:0101005)	0.0208771	0.1490802	0	0	2.333982473	9.030416998	*USP13;USP8;USP37;USP15;USP9X;USP9Y;JOSD1;MYSM1;OTUD1*
Molecular Function	Histone H4K20 Methyltransferase Activity (GO:0042799)	0.0240966	0.1638032	0	0	10.73816156	40.00701614	*NSD2;KMT5B*

Molecular Function	Nucleotide Transmembrane Transporter Activity (GO:0015215)	0.0240966	0.1638032	0	0	10.73816156	40.00701614	*SLC25A32;SLC25A36*
Molecular Function	rRNA Binding (GO:0019843)	0.024206	0.1638032	0	0	3.281425891	12.21068834	*MRPS27;ERI1;MRPS11;CIRBP;RPF2*
Molecular Function	RNA Polymerase II Complex Binding (GO:0000993)	0.0242312	0.1638032	0	0	3.983654045	14.8196413	*WAC;HNRNPU;RPRD1A;RPRD2*
Molecular Function	Cysteine-Type Deubiquitinase Activity (GO:0004843)	0.0250318	0.1667698	0	0	2.395755306	8.834602762	*USP13;USP8;USP37;USP15;USP9X;USP9Y;JOSD1;OTUD1*
Molecular Function	5'-3' RNA Polymerase Activity (GO:0034062)	0.025328	0.1667698	0	0	5.373779637	19.75318868	*POLR3C;MED21;POLR3F*
Molecular Function	Voltage-Gated Calcium Channel Activity (GO:0005245)	0.0263121	0.1710284	0	0	3.203130203	11.65211517	*CACNB4;CACNA2D2;CACNA1C;OPRM1;TPCN2*
Molecular Function	Ubiquitin-Like Protein Peptidase Activity (GO:0019783)	0.0269273	0.1728118	0	0	3.841181165	13.88439207	*USP15;USP9X;UFSP2;SENP1*
Molecular Function	RNA Polymerase II CTD Heptapeptide Repeat Kinase Activity (GO:0008353)	0.0313715	0.1913634	0	0	8.948003714	30.97669	*DYRK1A;MAPK1*
Molecular Function	Glucosyltransferase Activity (GO:0046527)	0.0313715	0.1913634	0	0	8.948003714	30.97669	*POGLUT1;ALG10B*
Molecular Function	Histone H3K14 Acetyltransferase Activity (GO:0036408)	0.0313715	0.1913634	0	0	8.948003714	30.97669	*BRPF3;KAT6A*
Molecular Function	palmitoyl-CoA Hydrolase Activity (GO:0016290)	0.0313715	0.1913634	0	0	8.948003714	30.97669	*MBLAC2;THEM4*
Molecular Function	Guanyl-Nucleotide Exchange Factor Activity (GO:0005085)	0.0325484	0.1913634	0	0	1.847465198	6.327616781	*DENND1B;ARFGEF2;ARHGEF12;DIS3;ITSN1;DENND4C;RASGRP1;RAB3GAP2;ARHGEF7;SOS1;FNIP1;RAPGEF6;RALGPS2*
Molecular Function	3'-5'-RNA Exonuclease Activity (GO:0000175)	0.0328375	0.1913634	0	0	3.584729981	12.24609638	*CNOT6;ERI1;DIS3;ERI2*
Molecular Function	Transcription Coactivator Binding (GO:0001223)	0.0328375	0.1913634	0	0	3.584729981	12.24609638	*CCNT2;EP300;MED6;VGLL4*
Molecular Function	DNA-directed 5'-3' RNA Polymerase Activity (GO:0003899)	0.0328375	0.1913634	0	0	3.584729981	12.24609638	*POLR3C;MED21;POLR1D;POLR3F*
Molecular Function	Ubiquitin-Specific Protease Binding (GO:1990381)	0.0335613	0.1933591	0	0	4.741078021	16.09302334	*VCP;SPATA2;LCOR*
Molecular Function	Store-Operated Calcium Channel Activity (GO:0015279)	0.0393872	0.2147235	0	0	7.669319538	24.80500011	*ORAI3;ORAI2*
Molecular Function	G-quadruplex DNA Binding (GO:0051880)	0.0393872	0.2147235	0	0	7.669319538	24.80500011	*XRN1;DHX36*
Molecular Function	G-rich Strand Telomeric DNA Binding (GO:0098505)	0.0393872	0.2147235	0	0	7.669319538	24.80500011	*HNRNPA2B1;TERF2IP*
Molecular Function	co-SMAD Binding (GO:0070410)	0.0393872	0.2147235	0	0	7.669319538	24.80500011	*USP9X;USP9Y*
Molecular Function	Histone H3K9 Methyltransferase Activity (GO:0046974)	0.0393872	0.2147235	0	0	7.669319538	24.80500011	*SETD5;SETDB1*
Molecular Function	GTPase Activator Activity (GO:0005096)	0.0423147	0.2271493	0	0	1.772077208	5.604406618	*ELMOD2;RALGAPA2;ARHGAP29;ARHGAP39;ARHGAP5;TBC1D1;ADGRB3;RASA1;TBC1D30;SOS1;EVI5;SEC23B;RGS7*
Molecular Function	Cysteine-Type Endopeptidase Inhibitor Activity (GO:0004869)	0.0430105	0.2271493	0	0	4.241576745	13.34531793	*PRKAA2;SPOCK1;ZDHHC17*
Molecular Function	Protein Kinase A Regulatory Subunit Binding (GO:0034237)	0.0430105	0.2271493	0	0	4.241576745	13.34531793	*ARFGEF2;PRRC1;WASF3*
Molecular Function	Calcium Channel Activity (GO:0005262)	0.0457796	0.2392808	0	0	2.243571763	6.918990333	*CACNB4;ORAI3;ORAI2;CACNA2D2;OPRM1;CACNA1C;TPCN2*
Molecular Function	mRNA 3'-UTR AU-rich Region Binding (GO:0035925)	0.0481802	0.2467409	0	0	4.029288703	12.22005799	*DHX36;CPEB3;HNRNPA0*
Molecular Function	Protein-Cysteine S-palmitoyltransferase Activity (GO:0019706)	0.0481802	0.2467409	0	0	4.029288703	12.22005799	*ZDHHC21;ZDHHC7;ZDHHC17*
Molecular Function	Catalytic Activity, Acting On A tRNA (GO:0140101)	0.0506757	0.2569259	0	0	3.071827614	9.161137363	*DTWD2;QTRT2;PUS10;TRDMT1*
Molecular Function	mRNA 5'-UTR Binding (GO:0048027)	0.053639	0.2640286	0	0	3.837218569	11.22570494	*DDX3X;MRPS11;DHX36*
Molecular Function	Peptidase Activator Activity (GO:0016504)	0.053639	0.2640286	0	0	3.837218569	11.22570494	*VCP;WDR48;PSME4*
Molecular Function	Phosphatidylinositol-3,5-Bisphosphate Binding (GO:0080025)	0.053639	0.2640286	0	0	3.837218569	11.22570494	*WDR45B;CLVS2;TPCN2*
Molecular Function	Voltage-Gated Monoatomic Cation Channel Activity (GO:0022843)	0.0558831	0.269431	0	0	2.306122449	6.651995107	*CACNB4;KCND2;CACNA2D2;OPRM1;CACNA1C;TPCN2*
Molecular Function	Endonuclease Activity (GO:0004519)	0.0572262	0.269431	0	0	2.536878216	7.257356271	*AGO3;DIS3;ERCC4;ZC3H12B;N4BP2*

Molecular Function	Dynactin Binding (GO:0034452)	0.0573936	0.269431	0	0	5.964407304	17.04521821	*GSK3B;HOOK3*
Molecular Function	Histone Methyltransferase Binding (GO:1990226)	0.0573936	0.269431	0	0	5.964407304	17.04521821	*CTNNB1;LCOR*
Molecular Function	Single-Stranded Telomeric DNA Binding (GO:0043047)	0.0573936	0.269431	0	0	5.964407304	17.04521821	*HNRNPA2B1;TERF2IP*
Molecular Function	poly(A) Binding (GO:0008143)	0.0593803	0.2761999	0	0	3.662609357	10.34245272	*KHDRBS1;DDX3X;HNRNPU*
Molecular Function	3'-5' Exonuclease Activity (GO:0008408)	0.0634716	0.2873207	0	0	2.828873861	7.799667418	*ERI1;DIS3;ERCC4;ERI2*
Molecular Function	Promoter-Specific Chromatin Binding (GO:1990841)	0.0634716	0.2873207	0	0	2.828873861	7.799667418	*DNMT1;ZNF609;SETDB1;HNRNPU*
Molecular Function	Regulatory RNA Binding (GO:0061980)	0.0634716	0.2873207	0	0	2.828873861	7.799667418	*AGO3;HNRNPA2B1;PUM1;PUM2*
Molecular Function	GTPase Binding (GO:0051020)	0.0640379	0.2873207	0	0	1.653838969	4.545211729	*MYO5A;ATG14;AP3M1;KIF3A;TMEM127;AP1G1;RASA1;RRAGD;TBC1D30;RAB3GAP2;RAF1;RAPGEF6;EVI5*
Molecular Function	Transition Metal Ion Binding (GO:0046914)	0.0662308	0.2890314	0	0	1.436313995	3.899032372	*KDM5B;KDM2B;ZMYND8;ERAP1;SLC11A2;SIAH1;RNF8;SIRT5;LNPEP;PRDM2;IDE;BAZ1B;RASGRP1;PHF8;SLC25A16;KAT6A;ZNF84;PHF14;ARIH1;FBXL5;RBAK;ZFP37;KDM7A*
Molecular Function	Transcription Coregulator Binding (GO:0001221)	0.0662528	0.2890314	0	0	2.047624855	5.557822884	*CCNT2;CHD6;EP300;LCOR;CTNNB1;MED6;VGLL4*
Molecular Function	Protein Tyrosine Kinase Activity (GO:0004713)	0.0662528	0.2890314	0	0	2.047624855	5.557822884	*FER;PEAK1;DYRK1A;BAZ1B;HIPK1;CAMKK2;TTN*
Molecular Function	RNA Polymerase II Core Promoter Sequence-Specific DNA Binding (GO:0000979)	0.0672696	0.2890314	0	0	5.367688022	14.48763891	*GTF2A1;POU2F1*
Molecular Function	Protein Kinase A Catalytic Subunit Binding (GO:0034236)	0.0672696	0.2890314	0	0	5.367688022	14.48763891	*GSK3B;PRKAR2A*
Molecular Function	Protein Serine/Threonine Kinase Activity (GO:0004674)	0.0702724	0.299396	0	0	1.500158278	3.983483772	*GSK3B;PRKAA1;PRKAA2;PRKDC;PDPK1;DYRK1A;CASK;HIPK1;CAMKK2;TTN;STK17B;MAPK1;PKN2;RAF1;PRKACB;MARK3;MAP4K3;MAP3K4*
Molecular Function	Signaling Receptor Complex Adaptor Activity (GO:0030159)	0.0728541	0.3078085	0	0	2.687150838	7.038445652	*SPATA2;SORBS1;NCK1;SH2B1*
Molecular Function	5'-3' Exonuclease Activity (GO:0008409)	0.0776565	0.3149747	0	0	4.879463155	12.46927407	*XRN1;DCP2*
Molecular Function	Ubiquitination-Like Modification-Dependent Protein Binding (GO:0140035)	0.0776565	0.3149747	0	0	4.879463155	12.46927407	*VCP;IDE*
Molecular Function	Histone H4 Methyltransferase Activity (GO:0140939)	0.0776565	0.3149747	0	0	4.879463155	12.46927407	*NSD2;KMT5B*
Molecular Function	Metalloendopeptidase Inhibitor Activity (GO:0008191)	0.0776565	0.3149747	0	0	4.879463155	12.46927407	*SPOCK2;SPOCK1*
Molecular Function	Ribosomal Large Subunit Binding (GO:0043023)	0.0776565	0.3149747	0	0	4.879463155	12.46927407	*CPSF6;MRRF*
Molecular Function	Double-Stranded RNA Binding (GO:0003725)	0.0844821	0.33994	0	0	2.24009324	5.535752312	*AGO3;ZFR;DHX36;HNRNPU;MTDH*
Molecular Function	Cysteine-Type Endopeptidase Activity (GO:0004197)	0.0880533	0.3505615	0	0	1.902149121	4.621867047	*USP13;USP8;USP37;USP15;USP9X;CASP3;USP9Y*
Molecular Function	Myosin V Binding (GO:0031489)	0.0885047	0.3505615	0	0	4.472609099	10.84473444	*RAB14;RAB39B*
Molecular Function	General Transcription Initiation Factor Binding (GO:0140296)	0.0920518	0.3617848	0	0	2.983573532	7.117028796	*GTF2A1;DR1;BCL10*
Molecular Function	SH2 Domain Binding (GO:0042169)	0.0993183	0.3831985	0	0	2.876867902	6.643913181	*KHDRBS1;AFAP1;ARHGAP5*
Molecular Function	Poly-Purine Tract Binding (GO:0070717)	0.0993183	0.3831985	0	0	2.876867902	6.643913181	*KHDRBS1;DDX3X;HNRNPU*
Molecular Function	tRNA-specific Ribonuclease Activity (GO:0004549)	0.0997677	0.3831985	0	0	4.128347975	9.515475487	*TSEN2;RPP14*
Molecular Function	Wnt Receptor Activity (GO:0042813)	0.1114016	0.4214971	0	0	3.833267012	8.412540272	*FZD3;LRP6*
Molecular Function	Ribosomal Small Subunit Binding (GO:0043024)	0.1114016	0.4214971	0	0	3.833267012	8.412540272	*DDX3X;EIF1B*
Molecular Function	DNA Secondary Structure Binding (GO:0000217)	0.1145081	0.4300416	0	0	2.684797768	5.818250628	*YY1;LIN54;SMC6*
Molecular Function	Phosphoric Ester Hydrolase Activity (GO:0042578)	0.116771	0.4353155	0	0	2.238361266	4.806971048	*DUSP5;PLCXD3;GDPD1;HDHD2*
Molecular Function	Nuclear Receptor Binding (GO:0016922)	0.1213338	0.44359	0	0	1.742818555	3.675970776	*NCOA6;EP300;LCOR;CTNNB1;TRIP12;ARID1A;PPARGC1A*
Molecular Function	Nuclear Estrogen Receptor Binding (GO:0030331)	0.1224117	0.44359	0	0	2.598056418	5.456866708	*NCOA6;CTNNB1;LCOR*
Molecular Function	Antiporter Activity (GO:0015297)	0.1229255	0.44359	0	0	2.192566412	4.596007714	*SLC25A16;SLC7A6;SLC35D1;SLC35E2B*
Molecular Function	RNA Polymerase Binding (GO:0070063)	0.1233653	0.44359	0	0	3.577530176	7.486360286	*CCNT2;PKN2*

Molecular Function	Histone Acetyltransferase Binding (GO:0035035)	0.1233653	0.44359	0	0	3.577530176	7.486360286	*MEF2A;KANSL1L*
Molecular Function	Core Promoter Sequence-Specific DNA Binding (GO:0001046)	0.1305078	0.4659679	0	0	2.516736402	5.124887083	*GTF2A1;RRN3;POU2F1*
Molecular Function	Single-Stranded DNA Binding (GO:0003697)	0.1324279	0.4695172	0	0	1.79178338	3.622478464	*WDR48;ERCC4;POLR3C;DHX36;HNRNPU;SMC6*
Molecular Function	TBP-class Protein Binding (GO:0017025)	0.1356199	0.4774952	0	0	3.353760446	6.700474552	*GTF2A1;DR1*
Molecular Function	Non-Membrane Spanning Protein Tyrosine Kinase Activity (GO:0004715)	0.1387861	0.4852727	0	0	2.440344871	4.819245499	*FER;PEAK1;DYRK1A*
Molecular Function	Protein Serine/Threonine Kinase Activator Activity (GO:0043539)	0.1421825	0.4937433	0	0	2.065749893	4.029542563	*CCNT2;DDX3X;TRAF6;SPRY2*
Molecular Function	Methyltransferase Activity (GO:0008168)	0.1472366	0.497361	0	0	2.36844696	4.537268705	*DNMT1;CARNMT1;NDUFAF7*
Molecular Function	1-Acylglycerol-3-Phosphate O-acyltransferase Activity (GO:0003841)	0.1481292	0.497361	0	0	3.156316566	6.02752398	*GPAT3;LCLAT1*
Molecular Function	R-SMAD Binding (GO:0070412)	0.1481292	0.497361	0	0	3.156316566	6.02752398	*PPM1A;SMURF1*
Molecular Function	Metalloaminopeptidase Activity (GO:0070006)	0.1481292	0.497361	0	0	3.156316566	6.02752398	*ERAP1;LNPEP*
Molecular Function	Microtubule Plus-End Binding (GO:0051010)	0.1481292	0.497361	0	0	3.156316566	6.02752398	*FBXW11;CLASP2*
Molecular Function	acyl-CoA Hydrolase Activity (GO:0047617)	0.160859	0.5025402	0	0	2.980810894	5.44661858	*MBLAC2;THEM4*
Molecular Function	Lysophosphatidic Acid Acyltransferase Activity (GO:0042171)	0.160859	0.5025402	0	0	2.980810894	5.44661858	*GPAT3;LCLAT1*
Molecular Function	Phosphatidylinositol Phospholipase C Activity (GO:0004435)	0.160859	0.5025402	0	0	2.980810894	5.44661858	*PLCB4;PLCH1*
Molecular Function	Pentosyltransferase Activity (GO:0016763)	0.1646135	0.5025402	0	0	2.236634124	4.035234327	*PARP11;QTRT2;SIRT5*
Molecular Function	Supercoiled DNA Binding (GO:0097100)	0.1675134	0.5025402	0	0	6.702364395	11.9750608	*PSIP1*
Molecular Function	4-galactosyl-N-acetylglucosaminide 3-alpha-L-fucosyltransferase Activity (GO:0017083)	0.1675134	0.5025402	0	0	6.702364395	11.9750608	*FUT9*
Molecular Function	DNA-methyltransferase Activity (GO:0009008)	0.1675134	0.5025402	0	0	6.702364395	11.9750608	*DNMT3A*
Molecular Function	G Protein-Coupled Opioid Receptor Activity (GO:0004985)	0.1675134	0.5025402	0	0	6.702364395	11.9750608	*OPRM1*
Molecular Function	Vasopressin Receptor Activity (GO:0005000)	0.1675134	0.5025402	0	0	6.702364395	11.9750608	*AVPR1B*
Molecular Function	RNA Endonuclease Activity, Producing 3'-Phosphomonoesters (GO:0016892)	0.1675134	0.5025402	0	0	6.702364395	11.9750608	*TSEN2*
Molecular Function	Annealing Activity (GO:0140666)	0.1675134	0.5025402	0	0	6.702364395	11.9750608	*DDX3X*
Molecular Function	Aspartic Endopeptidase Activity, Intramembrane Cleaving (GO:0042500)	0.1675134	0.5025402	0	0	6.702364395	11.9750608	*SPPL3*
Molecular Function	cAMP-dependent Protein Kinase Activity (GO:0004691)	0.1675134	0.5025402	0	0	6.702364395	11.9750608	*PRKACB*
Molecular Function	Cysteine-Type Endopeptidase Activity Involved In Execution Phase Of Apoptosis (GO:0097200)	0.1675134	0.5025402	0	0	6.702364395	11.9750608	*CASP3*
Molecular Function	deSUMOylase Activity (GO:0016929)	0.1675134	0.5025402	0	0	6.702364395	11.9750608	*SENP1*
Molecular Function	Melanocortin Receptor Activity (GO:0004977)	0.1675134	0.5025402	0	0	6.702364395	11.9750608	*OPRM1*
Molecular Function	Phosphatidylinositol-4,5-Bisphosphate 3-Kinase Activity (GO:0046934)	0.1675134	0.5025402	0	0	6.702364395	11.9750608	*PIK3CB*
Molecular Function	pre-mRNA 3'-Splice Site Binding (GO:0030628)	0.1675134	0.5025402	0	0	6.702364395	11.9750608	*U2AF2*
Molecular Function	Secondary Active Transmembrane Transporter Activity (GO:0015291)	0.1695481	0.5056523	0	0	1.917797287	3.403358607	*SLC25A16;SLC35D1;SLC4A4;SLC35E2B*
Molecular Function	DNA Endonuclease Activity (GO:0004520)	0.1737772	0.508262	0	0	2.823779504	4.941560966	*ERCC4;N4BP2*
Molecular Function	Ephrin Receptor Binding (GO:0046875)	0.1737772	0.508262	0	0	2.823779504	4.941560966	*CBL;NCK1*

Molecular Function	Small GTPase Binding (GO:0031267)	0.1819129	0.508262	0	0	1.422684254	2.424577146	*AP3M1;KIF3A;TMEM127;AP1G1;TBC1D30;MYO5A;RAB3GAP2;RAF1;RAPGEF6;EVI5*
Molecular Function	Signal Sequence Binding (GO:0005048)	0.1825582	0.508262	0	0	2.118696322	3.603237274	*SEC61A2;NUP153;TNPO1*
Molecular Function	PDZ Domain Binding (GO:0030165)	0.1838732	0.508262	0	0	1.851473704	3.135487174	*FZD3;SLC22A5;ARHGAP29;ATP2B2*
Molecular Function	Nucleosome Binding (GO:0031491)	0.1838732	0.508262	0	0	1.851473704	3.135487174	*SUPT16H;RBBP4;HP1BP3;ARID1A*
Molecular Function	poly(U) RNA Binding (GO:0008266)	0.1868539	0.508262	0	0	2.682451253	4.499619662	*KHDRBS1;SSB*
Molecular Function	Transcription Cis-Regulatory Region Binding (GO:0000976)	0.1926591	0.508262	0	0	1.248604913	2.056243885	*MEF2A;ATF2;OSR2;PBX2P1;ELL;TFAP2E;MAX;STAT3;ARID4A;FOXK1;ARID4B;PRDM2;NR2C2;ZNF14;YY1;TFAP4;NFIL3;TBL1XR1;DHX36;MEF2D;SOX4*
Molecular Function	1-Phosphatidylinositol-4-Phosphate 3-Kinase Activity (GO:0035005)	0.1974904	0.508262	0	0	5.361613352	8.696888062	*PIK3CB*
Molecular Function	Symporter Activity (GO:0015293)	0.1974904	0.508262	0	0	5.361613352	8.696888062	*SLC4A4*
Molecular Function	Telethonin Binding (GO:0031433)	0.1974904	0.508262	0	0	5.361613352	8.696888062	*TTN*
Molecular Function	DEAD/H-box RNA Helicase Binding (GO:0017151)	0.1974904	0.508262	0	0	5.361613352	8.696888062	*SMAD5*
Molecular Function	MHC Class II Protein Binding (GO:0042289)	0.1974904	0.508262	0	0	5.361613352	8.696888062	*MFAP3*
Molecular Function	NAD-dependent Protein Deacetylase Activity (GO:0034979)	0.1974904	0.508262	0	0	5.361613352	8.696888062	*SIRT5*
Molecular Function	RNA Polymerase II C-terminal Domain Binding (GO:0099122)	0.1974904	0.508262	0	0	5.361613352	8.696888062	*HNRNPU*
Molecular Function	Arachidonic Acid Binding (GO:0050544)	0.1974904	0.508262	0	0	5.361613352	8.696888062	*STX3*
Molecular Function	Beta-Galactoside (CMP) Alpha-2,3-Sialyltransferase Activity (GO:0003836)	0.1974904	0.508262	0	0	5.361613352	8.696888062	*ST3GAL5*
Molecular Function	Calcium-Dependent Protein Serine/Threonine Phosphatase Activity (GO:0004723)	0.1974904	0.508262	0	0	5.361613352	8.696888062	*PPM1A*
Molecular Function	Fatty Acid Elongase Activity (GO:0009922)	0.1974904	0.508262	0	0	5.361613352	8.696888062	*ELOVL4*
Molecular Function	Icosatetraenoic Acid Binding (GO:0050543)	0.1974904	0.508262	0	0	5.361613352	8.696888062	*STX3*
Molecular Function	Interleukin-6 Receptor Binding (GO:0005138)	0.1974904	0.508262	0	0	5.361613352	8.696888062	*ERAP1*
Molecular Function	Mannosyl-Oligosaccharide 1,2-Alpha-Mannosidase Activity (GO:0004571)	0.1974904	0.508262	0	0	5.361613352	8.696888062	*EDEM3*
Molecular Function	Mannosyl-Oligosaccharide Mannosidase Activity (GO:0015924)	0.1974904	0.508262	0	0	5.361613352	8.696888062	*EDEM3*
Molecular Function	Minor Groove Of Adenine-Thymine-Rich DNA Binding (GO:0003680)	0.1974904	0.508262	0	0	5.361613352	8.696888062	*LIN54*
Molecular Function	Phosphatidylinositol 3-Kinase Regulatory Subunit Binding (GO:0036312)	0.1974904	0.508262	0	0	5.361613352	8.696888062	*FAM83A*
Molecular Function	Pyrimidine Nucleotide-Sugar Transmembrane Transporter Activity (GO:0015165)	0.1974904	0.508262	0	0	5.361613352	8.696888062	*SLC35D1*
Molecular Function	Retinol Binding (GO:0019841)	0.1974904	0.508262	0	0	5.361613352	8.696888062	*ARID4A*
Molecular Function	High Voltage-Gated Calcium Channel Activity (GO:0008331)	0.2000609	0.5095141	0	0	2.554582836	4.11066453	*CACNB4;CACNA1C*
Molecular Function	Phospholipase C Activity (GO:0004629)	0.2000609	0.5095141	0	0	2.554582836	4.11066453	*PLCB4;PLCH1*
Molecular Function	snRNA Binding (GO:0017069)	0.2009917	0.5095141	0	0	2.012552301	3.22912294	*COIL;CCNT2;HNRNPU*
Molecular Function	Phosphoric Diester Hydrolase Activity (GO:0008081)	0.2103677	0.5155544	0	0	1.963363609	3.060684488	*PLCXD3;PLCB4;GDPD1*
Molecular Function	Nuclear Localization Sequence Binding (GO:0008139)	0.213372	0.5155544	0	0	2.43833882	3.766546197	*NUP153;TNPO1*
Molecular Function	Damaged DNA Binding (GO:0003684)	0.2198372	0.5155544	0	0	1.916517235	2.903270936	*ERCC4;EP300;SMC6*
Molecular Function	GDP Binding (GO:0019003)	0.2212574	0.5155544	0	0	1.704087967	2.570494896	*RAB14;RRAGD;GNAI3;TRIM23*
Molecular Function	Protein Kinase Binding (GO:0019901)	0.2228069	0.5155544	0	0	1.211178887	1.818524276	*CEP68;ATF2;KHDRBS1;GSK3B;USP37;AVPR1B;STAT3;PDE3B;XIAP;BCL10;TPCN2;SNAP91;TTN;NBEA;PRKAR2A;TRAF6;MFAP3;SPRY2;ARHGEF7;APPL1;HNRNPA0;FAM83A*
Molecular Function	tRNA (Cytosine) Methyltransferase Activity (GO:0016427)	0.2263893	0.5155544	0	0	4.467779323	6.636881855	*TRDMT1*

Molecular Function	tRNA-specific Adenosine Deaminase Activity (GO:0008251)	0.2263893	0.5155544	0	0	4.467779323	6.636881855	*ADAT2*
Molecular Function	7SK snRNA Binding (GO:0097322)	0.2263893	0.5155544	0	0	4.467779323	6.636881855	*CCNT2*
Molecular Function	Unmethylated CpG Binding (GO:0045322)	0.2263893	0.5155544	0	0	4.467779323	6.636881855	*KDM2B*
Molecular Function	Xylosyltransferase Activity (GO:0042285)	0.2263893	0.5155544	0	0	4.467779323	6.636881855	*POGLUT1*
Molecular Function	S-methyltransferase Activity (GO:0008172)	0.2263893	0.5155544	0	0	4.467779323	6.636881855	*DNMT3A*
Molecular Function	Alpha-(1->3)-Fucosyltransferase Activity (GO:0046920)	0.2263893	0.5155544	0	0	4.467779323	6.636881855	*FUT9*
Molecular Function	Arginine Binding (GO:0034618)	0.2263893	0.5155544	0	0	4.467779323	6.636881855	*SLC7A6*
Molecular Function	Coreceptor Activity Involved In Wnt Signaling Pathway (GO:0071936)	0.2263893	0.5155544	0	0	4.467779323	6.636881855	*LRP6*
Molecular Function	Cyclic Nucleotide-Dependent Protein Kinase Activity (GO:0004690)	0.2263893	0.5155544	0	0	4.467779323	6.636881855	*PRKACB*
Molecular Function	Icosanoid Binding (GO:0050542)	0.2263893	0.5155544	0	0	4.467779323	6.636881855	*STX3*
Molecular Function	Iron Ion Transmembrane Transporter Activity (GO:0005381)	0.2263893	0.5155544	0	0	4.467779323	6.636881855	*SLC11A2*
Molecular Function	Manganese Ion Transmembrane Transporter Activity (GO:0005384)	0.2263893	0.5155544	0	0	4.467779323	6.636881855	*SLC11A2*
Molecular Function	Mitochondrial Ribosome Binding (GO:0097177)	0.2263893	0.5155544	0	0	4.467779323	6.636881855	*MRPS27*
Molecular Function	Natural Killer Cell Lectin-Like Receptor Binding (GO:0046703)	0.2263893	0.5155544	0	0	4.467779323	6.636881855	*MICA*
Molecular Function	Oxidoreductase Activity, Acting On The Aldehyde Or Oxo Group Of Donors, Disulfide As Acceptor (GO:0016624)	0.2263893	0.5155544	0	0	4.467779323	6.636881855	*PDHA1*
Molecular Function	poly(G) Binding (GO:0034046)	0.2263893	0.5155544	0	0	4.467779323	6.636881855	*HNRNPU*
Molecular Function	Epidermal Growth Factor Receptor Binding (GO:0005154)	0.2267626	0.5155544	0	0	2.332202979	3.460643236	*EPGN;FER*
Molecular Function	Exonuclease Activity (GO:0004527)	0.229391	0.519202	0	0	1.871849762	2.75597529	*CNOT6;ERI1;ERCC4*
Molecular Function	Protein Phosphatase Binding (GO:0019903)	0.2341052	0.5197752	0	0	1.477989361	2.146017923	*VCP;AKAP11;KIF3A;STAT3;MTMR9;CTNNB1*
Molecular Function	NAD+ ADP-ribosyltransferase Activity (GO:0003950)	0.2402097	0.5197752	0	0	2.234911792	3.187526837	*PARP11;SIRT5*
Molecular Function	Tumor Necrosis Factor Receptor Superfamily Binding (GO:0032813)	0.2536921	0.5197752	0	0	2.1454039	2.942709093	*FEM1B;TRAF6*
Molecular Function	N-methyltransferase Activity (GO:0008170)	0.2536921	0.5197752	0	0	2.1454039	2.942709093	*TRMT11;METTL15*
Molecular Function	Nuclear Thyroid Hormone Receptor Binding (GO:0046966)	0.2536921	0.5197752	0	0	2.1454039	2.942709093	*NCOA6;TRIP12*
Molecular Function	5S rRNA Binding (GO:0008097)	0.254249	0.5197752	0	0	3.829326445	5.244036822	*RPF2*
Molecular Function	Tropomyosin Binding (GO:0005523)	0.254249	0.5197752	0	0	3.829326445	5.244036822	*NEBL*
Molecular Function	L-lysine Transmembrane Transporter Activity (GO:0015189)	0.254249	0.5197752	0	0	3.829326445	5.244036822	*SLC7A6*
Molecular Function	P-type Calcium Transporter Activity (GO:0005388)	0.254249	0.5197752	0	0	3.829326445	5.244036822	*ATP2B2*
Molecular Function	RNA Polymerase I Core Promoter Sequence-Specific DNA Binding (GO:0001164)	0.254249	0.5197752	0	0	3.829326445	5.244036822	*RRN3*
Molecular Function	UDP-xylosyltransferase Activity (GO:0035252)	0.254249	0.5197752	0	0	3.829326445	5.244036822	*POGLUT1*
Molecular Function	cAMP-dependent Protein Kinase Inhibitor Activity (GO:0004862)	0.254249	0.5197752	0	0	3.829326445	5.244036822	*PRKAR2A*
Molecular Function	Chloride:Monoatomic Cation Symporter Activity (GO:0015377)	0.254249	0.5197752	0	0	3.829326445	5.244036822	*SLC12A6*
Molecular Function	Cyclic-Nucleotide Phosphodiesterase Activity (GO:0004112)	0.254249	0.5197752	0	0	3.829326445	5.244036822	*PDE3B*

Molecular Function	Guanylate Kinase Activity (GO:0004385)	0.254249	0.5197752	0	0	3.829326445	5.244036822	*CASK*
Molecular Function	Histone H3K4 Demethylase Activity (GO:0032453)	0.254249	0.5197752	0	0	3.829326445	5.244036822	*KDM5B*
Molecular Function	Histone H4K16 Acetyltransferase Activity (GO:0046972)	0.254249	0.5197752	0	0	3.829326445	5.244036822	*KAT6A*
Molecular Function	Histone Reader Activity (GO:0140566)	0.254249	0.5197752	0	0	3.829326445	5.244036822	*PHF14*
Molecular Function	Metal-Dependent Deubiquitinase Activity (GO:0140492)	0.254249	0.5197752	0	0	3.829326445	5.244036822	*MYSM1*
Molecular Function	Peptide N-acetyltransferase Activity (GO:0034212)	0.254249	0.5197752	0	0	3.829326445	5.244036822	*EP300*
Molecular Function	Phosphatidylinositol Bisphosphate Kinase Activity (GO:0052813)	0.254249	0.5197752	0	0	3.829326445	5.244036822	*PIK3CB*
Molecular Function	Potassium:Chloride Symporter Activity (GO:0015379)	0.254249	0.5197752	0	0	3.829326445	5.244036822	*SLC12A6*
Molecular Function	pre-miRNA Binding (GO:0070883)	0.254249	0.5197752	0	0	3.829326445	5.244036822	*DHX36*
Molecular Function	Single-Stranded DNA Endodeoxyribonuclease Activity (GO:0000014)	0.254249	0.5197752	0	0	3.829326445	5.244036822	*ERCC4*
Molecular Function	DNA-binding Transcription Repressor Activity, RNA Polymerase II-specific (GO:0001227)	0.2609906	0.5220554	0	0	1.273819367	1.711084354	*YY1;KLF12;HEY1;NFIL3;MAX;PRDM2;FOXK1;ZNF555;ZNF169;ZFP37;ZNF554*
Molecular Function	RNA Endonuclease Activity, Producing 5'-Phosphomonoesters (GO:0016891)	0.2671896	0.5220554	0	0	2.06278123	2.722451741	*AGO3;RPP14*
Molecular Function	Cell Adhesion Mediator Activity (GO:0098631)	0.2671896	0.5220554	0	0	2.06278123	2.722451741	*CD47;CNTN4*
Molecular Function	S-adenosylmethionine-dependent Methyltransferase Activity (GO:0008757)	0.2682732	0.5220554	0	0	1.712187305	2.252809432	*DNMT1;CARNMT1;DNMT3A*
Molecular Function	Nuclear Receptor Coactivator Activity (GO:0030374)	0.2682732	0.5220554	0	0	1.712187305	2.252809432	*CNOT6;NCOA6;PPARGC1A*
Molecular Function	E-box Binding (GO:0070888)	0.278118	0.5220554	0	0	1.676429568	2.145343083	*TFAP4;MAX;CLOCK*
Molecular Function	Acylglycerol O-acyltransferase Activity (GO:0016411)	0.2806839	0.5220554	0	0	1.986278758	2.523619178	*GPAT3;LCLAT1*
Molecular Function	Aminopeptidase Activity (GO:0004177)	0.2806839	0.5220554	0	0	1.986278758	2.523619178	*ERAP1;LNPEP*
Molecular Function	Metal Ion Transmembrane Transporter Activity (GO:0046873)	0.2806839	0.5220554	0	0	1.986278758	2.523619178	*SLC11A2;ZDHHC13*
Molecular Function	1-Phosphatidylinositol-3-Kinase Activity (GO:0016303)	0.2811068	0.5220554	0	0	3.350486787	4.251837124	*PIK3CB*
Molecular Function	MAP Kinase Tyrosine Phosphatase Activity (GO:0033550)	0.2811068	0.5220554	0	0	3.350486787	4.251837124	*DUSP5*
Molecular Function	MAP Kinase Tyrosine/Serine/Threonine Phosphatase Activity (GO:0017017)	0.2811068	0.5220554	0	0	3.350486787	4.251837124	*DUSP5*
Molecular Function	RNA Polymerase I Transcription Regulatory Region Sequence-Specific DNA Binding (GO:0001163)	0.2811068	0.5220554	0	0	3.350486787	4.251837124	*RRN3*
Molecular Function	RNA Polymerase II General Transcription Initiation Factor Binding (GO:0001091)	0.2811068	0.5220554	0	0	3.350486787	4.251837124	*GTF2A1*
Molecular Function	STAT Family Protein Binding (GO:0097677)	0.2811068	0.5220554	0	0	3.350486787	4.251837124	*EP300*
Molecular Function	U3 snoRNA Binding (GO:0034511)	0.2811068	0.5220554	0	0	3.350486787	4.251837124	*PRKDC*
Molecular Function	cAMP Response Element Binding (GO:0035497)	0.2811068	0.5220554	0	0	3.350486787	4.251837124	*CREB1*
Molecular Function	Clathrin Heavy Chain Binding (GO:0032050)	0.2811068	0.5220554	0	0	3.350486787	4.251837124	*SNAP91*
Molecular Function	Cytidylyltransferase Activity (GO:0070567)	0.2811068	0.5220554	0	0	3.350486787	4.251837124	*CDS2*
Molecular Function	Eukaryotic Initiation Factor 4E Binding (GO:0008190)	0.2811068	0.5220554	0	0	3.350486787	4.251837124	*DDX3X*
Molecular Function	Histone H3 Kinase Activity (GO:0140996)	0.2811068	0.5220554	0	0	3.350486787	4.251837124	*DYRK1A*

Molecular Function	Protein Binding Involved In Heterotypic Cell-Cell Adhesion (GO:0086080)	0.2811068	0.5220554	0	0	3.350486787	4.251837124	*CD47*
Molecular Function	Protein Tyrosine/Threonine Phosphatase Activity (GO:0008330)	0.2811068	0.5220554	0	0	3.350486787	4.251837124	*DUSP5*
Molecular Function	Ribonuclease P RNA Binding (GO:0033204)	0.2811068	0.5220554	0	0	3.350486787	4.251837124	*RPP14*
Molecular Function	Small Ribosomal Subunit rRNA Binding (GO:0070181)	0.2811068	0.5220554	0	0	3.350486787	4.251837124	*MRPS11*
Molecular Function	Transcription Regulatory Region Nucleic Acid Binding (GO:0001067)	0.2884697	0.5337741	0	0	1.254837436	1.559970454	*YY1;LIN54;TFAP4;TBL1XR1;DHX36;STAT3;ARID4A;ARID4B;FOXK1;SOX4*
Molecular Function	Protease Binding (GO:0002020)	0.2951748	0.5377606	0	0	1.353082409	1.651014334	*GSK3B;VCP;SPATA2;LCOR;BCL10;TTN*
Molecular Function	L-arginine Transmembrane Transporter Activity (GO:0061459)	0.3069985	0.5377606	0	0	2.978055942	3.516822846	*SLC7A6*
Molecular Function	UDP-glucosyltransferase Activity (GO:0035251)	0.3069985	0.5377606	0	0	2.978055942	3.516822846	*POGLUT1*
Molecular Function	Ammonium Transmembrane Transporter Activity (GO:0008519)	0.3069985	0.5377606	0	0	2.978055942	3.516822846	*SLC12A6*
Molecular Function	cAMP-dependent Protein Kinase Regulator Activity (GO:0008603)	0.3069985	0.5377606	0	0	2.978055942	3.516822846	*PRKAR2A*
Molecular Function	Fatty Acid Synthase Activity (GO:0004312)	0.3069985	0.5377606	0	0	2.978055942	3.516822846	*ELOVL4*
Molecular Function	Filamin Binding (GO:0031005)	0.3069985	0.5377606	0	0	2.978055942	3.516822846	*NEBL*
Molecular Function	Magnesium Ion Transmembrane Transporter Activity (GO:0015095)	0.3069985	0.5377606	0	0	2.978055942	3.516822846	*ZDHHC13*
Molecular Function	Nuclear Retinoid X Receptor Binding (GO:0046965)	0.3069985	0.5377606	0	0	2.978055942	3.516822846	*NCOA6*
Molecular Function	Outward Rectifier Potassium Channel Activity (GO:0015271)	0.3069985	0.5377606	0	0	2.978055942	3.516822846	*KCND2*
Molecular Function	Phospholipase Activator Activity (GO:0016004)	0.3069985	0.5377606	0	0	2.978055942	3.516822846	*PDPK1*
Molecular Function	Protein-Arginine N-methyltransferase Activity (GO:0016274)	0.3069985	0.5377606	0	0	2.978055942	3.516822846	*NDUFAF7*
Molecular Function	Racemase And Epimerase Activity, Acting On Carbohydrates And Derivatives (GO:0016857)	0.3069985	0.5377606	0	0	2.978055942	3.516822846	*GLCE*
Molecular Function	Ribonuclease P Activity (GO:0004526)	0.3069985	0.5377606	0	0	2.978055942	3.516822846	*RPP14*
Molecular Function	Semaphorin Receptor Activity (GO:0017154)	0.3069985	0.5377606	0	0	2.978055942	3.516822846	*PLXNC1*
Molecular Function	O-acyltransferase Activity (GO:0008374)	0.3075948	0.5377606	0	0	1.849101911	2.180039222	*GPAT3;LCLAT1*
Molecular Function	Protein Serine/Threonine Phosphatase Activity (GO:0004722)	0.3177573	0.5435056	0	0	1.547151593	1.773758873	*PPP6C;PPM1A;PPM1G*
Molecular Function	Ubiquitin Conjugating Enzyme Activity (GO:0061631)	0.3209807	0.5435056	0	0	1.787372331	2.031124152	*UBE2W;UBE2D4*
Molecular Function	Protein Serine/Threonine Kinase Inhibitor Activity (GO:0030291)	0.3209807	0.5435056	0	0	1.787372331	2.031124152	*PRKAR2A;SPRY2*
Molecular Function	Hydrolase Activity, Acting On Carbon-Nitrogen (But Not Peptide) Bonds, In Linear Amides (GO:0016811)	0.3276955	0.5435056	0	0	1.517881108	1.693455142	*MIER3;SIRT5;GLS*
Molecular Function	Iron Ion Binding (GO:0005506)	0.3276955	0.5435056	0	0	1.517881108	1.693455142	*FBXL5;KDM7A;PHF8*
Molecular Function	tRNA (Guanine) Methyltransferase Activity (GO:0016423)	0.331959	0.5435056	0	0	2.680111266	2.955475831	*TRMT11*
Molecular Function	GABA Receptor Binding (GO:0050811)	0.331959	0.5435056	0	0	2.680111266	2.955475831	*ARFGEF2*

Molecular Function	Voltage-Gated Potassium Channel Activity Involved In Ventricular Cardiac Muscle Cell Action Potential Repolarization (GO:1902282)	0.331959	0.5435056	0	0	2.680111266	2.955475831	*KCNJ3*
Molecular Function	MAP Kinase Activity (GO:0004707)	0.331959	0.5435056	0	0	2.680111266	2.955475831	*MAPK1*
Molecular Function	P-type Ion Transporter Activity (GO:0015662)	0.331959	0.5435056	0	0	2.680111266	2.955475831	*ATP2B2*
Molecular Function	RNA Polymerase I Activity (GO:0001054)	0.331959	0.5435056	0	0	2.680111266	2.955475831	*POLR1D*
Molecular Function	Cysteine-Type Endopeptidase Activity Involved In Apoptotic Process (GO:0097153)	0.331959	0.5435056	0	0	2.680111266	2.955475831	*CASP3*
Molecular Function	Fucosyltransferase Activity (GO:0008417)	0.331959	0.5435056	0	0	2.680111266	2.955475831	*FUT9*
Molecular Function	Ligand-Gated Sodium Channel Activity (GO:0015280)	0.331959	0.5435056	0	0	2.680111266	2.955475831	*TPCN2*
Molecular Function	Monoatomic Cation:Bicarbonate Symporter Activity (GO:0140410)	0.331959	0.5435056	0	0	2.680111266	2.955475831	*SLC4A4*
Molecular Function	Phosphatidylinositol 3-Kinase Activity (GO:0035004)	0.331959	0.5435056	0	0	2.680111266	2.955475831	*PIK3CB*
Molecular Function	Pseudouridine Synthase Activity (GO:0009982)	0.331959	0.5435056	0	0	2.680111266	2.955475831	*PUS10*
Molecular Function	Sequence-Specific mRNA Binding (GO:1990825)	0.331959	0.5435056	0	0	2.680111266	2.955475831	*SSB*
Molecular Function	Telomeric DNA Binding (GO:0042162)	0.3343015	0.5435056	0	0	1.729625303	1.895171351	*HNRNPA2B1;TERF2IP*
Molecular Function	Transition Metal Ion Transmembrane Transporter Activity (GO:0046915)	0.3343015	0.5435056	0	0	1.729625303	1.895171351	*SLC30A5;SLC11A2*
Molecular Function	Frizzled Binding (GO:0005109)	0.3343015	0.5435056	0	0	1.729625303	1.895171351	*ZNRF3;LRP6*
Molecular Function	Phosphatidylinositol Binding (GO:0035091)	0.334465	0.5435056	0	0	1.327909714	1.45435737	*DENND1B;ING2;SNX27;SNAP91;APPL1*
Molecular Function	Kinase Inhibitor Activity (GO:0019210)	0.3475446	0.5535698	0	0	1.675487465	1.770759611	*LRP6;NCK1*
Molecular Function	Nuclease Activity (GO:0004518)	0.3475581	0.5535698	0	0	1.462533283	1.545639413	*DFFB;DIS3;N4BP2*
Molecular Function	Phosphatidylinositol Phosphate Binding (GO:1901981)	0.3559595	0.5535698	0	0	1.289402905	1.331873684	*DENND1B;SNX27;WDR45B;CLVS2;PLEKHA3*
Molecular Function	LRR Domain Binding (GO:0030275)	0.3560217	0.5535698	0	0	2.436338349	2.516161515	*LRRFIP2*
Molecular Function	NAD+ Binding (GO:0070403)	0.3560217	0.5535698	0	0	2.436338349	2.516161515	*SIRT5*
Molecular Function	RNA 7-Methylguanosine Cap Binding (GO:0000340)	0.3560217	0.5535698	0	0	2.436338349	2.516161515	*EIF4E2*
Molecular Function	Acid-Thiol Ligase Activity (GO:0016878)	0.3560217	0.5535698	0	0	2.436338349	2.516161515	*AASDH*
Molecular Function	Hydrolase Activity, Acting On Carbon-Nitrogen (But Not Peptide) Bonds, In Cyclic Amides (GO:0016812)	0.3560217	0.5535698	0	0	2.436338349	2.516161515	*MBLAC2*
Molecular Function	Intracellular Ligand-Gated Monoatomic Ion Channel Activity (GO:0005217)	0.3560217	0.5535698	0	0	2.436338349	2.516161515	*TPCN2*
Molecular Function	Lipase Activator Activity (GO:0060229)	0.3560217	0.5535698	0	0	2.436338349	2.516161515	*PDPK1*
Molecular Function	poly(A)-specific Ribonuclease Activity (GO:0004535)	0.3560217	0.5535698	0	0	2.436338349	2.516161515	*CNOT6*
Molecular Function	Solute:Monoatomic Cation Symporter Activity (GO:0015294)	0.3560217	0.5535698	0	0	2.436338349	2.516161515	*SLC11A2*
Molecular Function	SH3 Domain Binding (GO:0017124)	0.3574698	0.5535698	0	0	1.436341901	1.477571091	*KHDRBS1;AFAP1;CBL*
Molecular Function	Carboxylic Acid Transmembrane Transporter Activity (GO:0046943)	0.3574698	0.5535698	0	0	1.436341901	1.477571091	*SLC7A6;SLC25A32;SLC19A2*
Molecular Function	Receptor Tyrosine Kinase Binding (GO:0030971)	0.3574698	0.5535698	0	0	1.436341901	1.477571091	*CBL;NCK1;SH2B1*
Molecular Function	GTPase Activity (GO:0003924)	0.358128	0.5535698	0	0	1.162144753	1.193365474	*RGS17;DDX3X;RAB14;RASA1;RRAGD;ATL2;GNAI3;RAB39B;RABL2B;TRIM23;RGS7*

Molecular Function	Ubiquitin-Like Protein Conjugating Enzyme Activity (GO:0061650)	0.3606983	0.5558482	0	0	1.624630708	1.65665762	*UBE2W;UBE2D4*
Molecular Function	Protein Tyrosine Kinase Binding (GO:1990782)	0.3664422	0.5629885	0	0	1.324160287	1.329343566	*KHDRBS1;MFAP3;CBL;NCK1*
Molecular Function	Protein Heterodimerization Activity (GO:0046982)	0.3768587	0.5638239	0	0	1.179034449	1.150602065	*MEF2A;QTRT2;GTF2A1;ZHX3;RALGAPA2;RRAGD;NOLC1;SUPT5H*
Molecular Function	3',5'-cyclic-AMP Phosphodiesterase Activity (GO:0004115)	0.3792188	0.5638239	0	0	2.233194251	2.165398769	*PDE3B*
Molecular Function	tRNA Methyltransferase Activity (GO:0008175)	0.3792188	0.5638239	0	0	2.233194251	2.165398769	*TRDMT1*
Molecular Function	Transmembrane Receptor Protein Tyrosine Kinase Adaptor Activity (GO:0005068)	0.3792188	0.5638239	0	0	2.233194251	2.165398769	*SH2B1*
Molecular Function	DNA Replication Origin Binding (GO:0003688)	0.3792188	0.5638239	0	0	2.233194251	2.165398769	*ORC1*
Molecular Function	Ubiquitin-Ubiquitin Ligase Activity (GO:0034450)	0.3792188	0.5638239	0	0	2.233194251	2.165398769	*TRAF6*
Molecular Function	K63-linked Deubiquitinase Activity (GO:0061578)	0.3792188	0.5638239	0	0	2.233194251	2.165398769	*USP9X*
Molecular Function	NADPH Binding (GO:0070402)	0.3792188	0.5638239	0	0	2.233194251	2.165398769	*HMGCR*
Molecular Function	SUMO Ligase Activity (GO:0061665)	0.3792188	0.5638239	0	0	2.233194251	2.165398769	*PIAS2*
Molecular Function	Histone H3K4 Methyltransferase Activity (GO:0042800)	0.3792188	0.5638239	0	0	2.233194251	2.165398769	*NSD3*
Molecular Function	Phosphatidylinositol Phosphate Kinase Activity (GO:0016307)	0.3792188	0.5638239	0	0	2.233194251	2.165398769	*PIK3CB*
Molecular Function	Ribonucleoside Triphosphate Phosphatase Activity (GO:0017111)	0.3810392	0.564874	0	0	1.139410012	1.099363057	*RGS17;DDX3X;RAB14;RASA1;RRAGD;ATL2;GNAI3;RAB39B;RABL2B;TRIM23;RGS7*
Molecular Function	G Protein-Coupled Receptor Binding (GO:0001664)	0.3911686	0.5751463	0	0	1.200677286	1.12697568	*ARHGEF12;PDCD6IP;ZNRF3;GNAI3;GPRASP2;LRP6*
Molecular Function	1-Phosphatidylinositol Binding (GO:0005545)	0.4015814	0.5751463	0	0	2.061303092	1.880619508	*SNAP91*
Molecular Function	3'-5' DNA Helicase Activity (GO:0043138)	0.4015814	0.5751463	0	0	2.061303092	1.880619508	*NAV2*
Molecular Function	Transmembrane-Ephrin Receptor Activity (GO:0005005)	0.4015814	0.5751463	0	0	2.061303092	1.880619508	*EPHB1*
Molecular Function	Voltage-Gated Potassium Channel Activity Involved In Cardiac Muscle Cell Action Potential Repolarization (GO:0086008)	0.4015814	0.5751463	0	0	2.061303092	1.880619508	*KCNJ3*
Molecular Function	Water Channel Activity (GO:0015250)	0.4015814	0.5751463	0	0	2.061303092	1.880619508	*TNPO1*
Molecular Function	MAP Kinase Phosphatase Activity (GO:0033549)	0.4015814	0.5751463	0	0	2.061303092	1.880619508	*DUSP5*
Molecular Function	RNA Exonuclease Activity (GO:0004532)	0.4015814	0.5751463	0	0	2.061303092	1.880619508	*DCP2*
Molecular Function	SUMO Binding (GO:0032183)	0.4015814	0.5751463	0	0	2.061303092	1.880619508	*RNF111*
Molecular Function	Adenine Nucleotide Transmembrane Transporter Activity (GO:0000295)	0.4015814	0.5751463	0	0	2.061303092	1.880619508	*SLC25A53*
Molecular Function	Cyclin-Dependent Protein Serine/Threonine Kinase Activator Activity (GO:0061575)	0.4015814	0.5751463	0	0	2.061303092	1.880619508	*CCNT2*
Molecular Function	Muscle Alpha-Actinin Binding (GO:0051371)	0.4015814	0.5751463	0	0	2.061303092	1.880619508	*TTN*
Molecular Function	Phosphotyrosine Residue Binding (GO:0001784)	0.4122182	0.5887173	0	0	1.448693819	1.283836041	*RASA1;CRKL*
Molecular Function	Death Receptor Binding (GO:0005123)	0.4231395	0.5975815	0	0	1.913967812	1.64611427	*FEM1B*
Molecular Function	Ephrin Receptor Activity (GO:0005003)	0.4231395	0.5975815	0	0	1.913967812	1.64611427	*EPHB1*
Molecular Function	Phospholipase Binding (GO:0043274)	0.4231395	0.5975815	0	0	1.913967812	1.64611427	*PDPK1*
Molecular Function	Protein Phosphatase Activator Activity (GO:0072542)	0.4231395	0.5975815	0	0	1.913967812	1.64611427	*PPP4R3B*
Molecular Function	Phosphatidylinositol-3-Phosphate Binding (GO:0032266)	0.4247813	0.5982336	0	0	1.410496995	1.207640621	*SNX27;WDR45B*

Molecular Function	Guanyl Ribonucleotide Binding (GO:0032561)	0.4268426	0.5994715	0	0	1.111999067	0.946689131	*ARL17A;RAB14;RRAGD;ATL2;GNAI3;RAB39B;ARL5A;TRIM23;RABL6*
Molecular Function	Phosphatidylinositol Bisphosphate Binding (GO:1902936)	0.4317958	0.6047527	0	0	1.204632478	1.011653349	*WDR45B;CLVS2;SNAP91;TPCN2*
Molecular Function	GTP Binding (GO:0005525)	0.4371874	0.6066536	0	0	1.111195203	0.919395546	*ARL17A;RAB14;RRAGD;ATL2;RAB39B;ARL5A;TRIM23;RABL6*
Molecular Function	Translation Initiation Factor Activity (GO:0003743)	0.4372029	0.6066536	0	0	1.374258982	1.137003856	*EIF4E2;EIF1B*
Molecular Function	RNA Endonuclease Activity (GO:0004521)	0.4372029	0.6066536	0	0	1.374258982	1.137003856	*AGO3;ZC3H12B*
Molecular Function	3',5'-Cyclic-Nucleotide Phosphodiesterase Activity (GO:0004114)	0.4439221	0.6066536	0	0	1.786277237	1.450646926	*PDE3B*
Molecular Function	CXCR Chemokine Receptor Binding (GO:0045236)	0.4439221	0.6066536	0	0	1.786277237	1.450646926	*CXCL13*
Molecular Function	Water Transmembrane Transporter Activity (GO:0005372)	0.4439221	0.6066536	0	0	1.786277237	1.450646926	*TNPO1*
Molecular Function	RNA Methyltransferase Activity (GO:0008173)	0.4439221	0.6066536	0	0	1.786277237	1.450646926	*TRDMT1*
Molecular Function	Four-Way Junction DNA Binding (GO:0000400)	0.4439221	0.6066536	0	0	1.786277237	1.450646926	*YY1*
Molecular Function	Phosphatidylinositol Kinase Activity (GO:0052742)	0.4439221	0.6066536	0	0	1.786277237	1.450646926	*PIK3CB*
Molecular Function	Purine Ribonucleoside Triphosphate Binding (GO:0035639)	0.4494133	0.6109515	0	0	1.053745381	0.842798509	*RTEL1;HSPA4L;ATL2;HNRNPU;RAB39B;IDE;ARL17A;RAB14;PEAK1;STK17B;RRAGD;DHX36;PRKACB;ARL5A;N4BP2;MAP4K3;RABL6;TRIM23*
Molecular Function	Transcription Corepressor Binding (GO:0001222)	0.4494772	0.6109515	0	0	1.339832869	1.071424487	*CTNNB1;LCOR*
Molecular Function	Cell-Cell Adhesion Mediator Activity (GO:0098632)	0.4615984	0.6173931	0	0	1.307086079	1.010456035	*CD47;CNTN4*
Molecular Function	Basic Amino Acid Transmembrane Transporter Activity (GO:0015174)	0.4639569	0.6173931	0	0	1.674547983	1.285992079	*SLC7A6*
Molecular Function	Calcium-Release Channel Activity (GO:0015278)	0.4639569	0.6173931	0	0	1.674547983	1.285992079	*TPCN2*
Molecular Function	Hydrolase Activity, Acting On Carbon-Nitrogen (But Not Peptide) Bonds, In Cyclic Amidines (GO:0016814)	0.4639569	0.6173931	0	0	1.674547983	1.285992079	*SLC25A16*
Molecular Function	Nuclear Retinoic Acid Receptor Binding (GO:0042974)	0.4639569	0.6173931	0	0	1.674547983	1.285992079	*NCOA6*
Molecular Function	Phosphatase Activator Activity (GO:0019211)	0.4639569	0.6173931	0	0	1.674547983	1.285992079	*PPP4R3B*
Molecular Function	Quaternary Ammonium Group Transmembrane Transporter Activity (GO:0015651)	0.4639569	0.6173931	0	0	1.674547983	1.285992079	*SLC22A5*
Molecular Function	RNA Nuclease Activity (GO:0004540)	0.4639581	0.6173931	0	0	1.199837632	0.921428522	*CNOT6;AGO3;ZC3H12B*
Molecular Function	Transforming Growth Factor Beta Receptor Binding (GO:0005160)	0.4832708	0.6342242	0	0	1.575963348	1.146006105	*USP15*
Molecular Function	Aspartic-Type Endopeptidase Activity (GO:0004190)	0.4832708	0.6342242	0	0	1.575963348	1.146006105	*SPPL3*
Molecular Function	Histone Deacetylase Activity (GO:0004407)	0.4832708	0.6342242	0	0	1.575963348	1.146006105	*MIER3*
Molecular Function	Mannose Binding (GO:0005537)	0.4832708	0.6342242	0	0	1.575963348	1.146006105	*CD209*
Molecular Function	Nucleoside Monophosphate Kinase Activity (GO:0050145)	0.4832708	0.6342242	0	0	1.575963348	1.146006105	*CASK*
Molecular Function	Protein Kinase Inhibitor Activity (GO:0004860)	0.4853629	0.6342242	0	0	1.246161819	0.900798591	*SPRY2;NCK1*
Molecular Function	Protein Phosphorylated Amino Acid Binding (GO:0045309)	0.4853629	0.6342242	0	0	1.246161819	0.900798591	*RASA1;CRKL*
Molecular Function	Cyclin-Dependent Protein Serine/Threonine Kinase Regulator Activity (GO:0016538)	0.496998	0.6441977	0	0	1.217776652	0.851432099	*CCNT2;CCNG2*
Molecular Function	DNA Polymerase Binding (GO:0070182)	0.5018898	0.6441977	0	0	1.488332561	1.026018955	*RTEL1*
Molecular Function	Voltage-Gated Sodium Channel Activity (GO:0005248)	0.5018898	0.6441977	0	0	1.488332561	1.026018955	*TPCN2*

Molecular Function	MAP Kinase Kinase Kinase Activity (GO:0004709)	0.5018898	0.6441977	0	0	1.488332561	1.026018955	*MAP3K4*
Molecular Function	RNA Cap Binding (GO:0000339)	0.5018898	0.6441977	0	0	1.488332561	1.026018955	*EIF4E2*
Molecular Function	Cysteine-Type Endopeptidase Inhibitor Activity Involved In Apoptotic Process (GO:0043027)	0.5018898	0.6441977	0	0	1.488332561	1.026018955	*XIAP*
Molecular Function	Histone Methyltransferase Activity (GO:0042054)	0.5018898	0.6441977	0	0	1.488332561	1.026018955	*KMT5B*
Molecular Function	Protein Tyrosine Phosphatase Activity (GO:0004725)	0.5187568	0.6539907	0	0	1.100876942	0.722527669	*DUSP5;EYA3;PTPN4*
Molecular Function	RNA Stem-Loop Binding (GO:0035613)	0.5198387	0.6539907	0	0	1.409926067	0.922425299	*DDX3X*
Molecular Function	Actinin Binding (GO:0042805)	0.5198387	0.6539907	0	0	1.409926067	0.922425299	*TTN*
Molecular Function	Deaminase Activity (GO:0019239)	0.5198387	0.6539907	0	0	1.409926067	0.922425299	*SLC25A16*
Molecular Function	Fibroblast Growth Factor Binding (GO:0017134)	0.5198387	0.6539907	0	0	1.409926067	0.922425299	*CXCL13*
Molecular Function	Phosphatidic Acid Binding (GO:0070300)	0.5198387	0.6539907	0	0	1.409926067	0.922425299	*RAPGEF6*
Molecular Function	Sialyltransferase Activity (GO:0008373)	0.5198387	0.6539907	0	0	1.409926067	0.922425299	*ST3GAL5*
Molecular Function	Solute:Proton Symporter Activity (GO:0015295)	0.5198387	0.6539907	0	0	1.409926067	0.922425299	*SLC11A2*
Molecular Function	Kinase Activity (GO:0016301)	0.5332658	0.6691177	0	0	1.050388871	0.66041658	*GSK3B;ETNK1;PKN2;PIK3CB*
Molecular Function	G Protein-Coupled Serotonin Receptor Activity (GO:0004993)	0.5371418	0.6691177	0	0	1.339360223	0.832403219	*DHX8*
Molecular Function	Lipid Kinase Activity (GO:0001727)	0.5371418	0.6691177	0	0	1.339360223	0.832403219	*PIK3CB*
Molecular Function	Lysophospholipase Activity (GO:0004622)	0.5371418	0.6691177	0	0	1.339360223	0.832403219	*GDPD1*
Molecular Function	ATP Binding (GO:0005524)	0.5508501	0.6845122	0	0	0.995392429	0.593545115	*RTEL1;PEAK1;STK17B;HSPA4L;DHX36;HNRNPU;IDE;PRKACB;N4BP2;MAP4K3*
Molecular Function	tRNA Binding (GO:0000049)	0.5525806	0.6845173	0	0	1.093229492	0.648455614	*MRPS27;SSB*
Molecular Function	Ubiquitin Binding (GO:0043130)	0.5535544	0.6845173	0	0	1.043471173	0.617103817	*USP13;WDR48;PLAA*
Molecular Function	Adenyl Ribonucleotide Binding (GO:0032559)	0.5582827	0.6879534	0	0	0.988262133	0.576047851	*RTEL1;PEAK1;STK17B;HSPA4L;DHX36;HNRNPU;IDE;HMGCR;PRKACB;N4BP2;MAP4K3*
Molecular Function	Channel Activity (GO:0015267)	0.563164	0.6879534	0	0	1.071309192	0.615129107	*SLC12A6;TNPO1*
Molecular Function	Exopeptidase Activity (GO:0008238)	0.563164	0.6879534	0	0	1.071309192	0.615129107	*ERAP1;LNPEP*
Molecular Function	Zinc Ion Transmembrane Transporter Activity (GO:0005385)	0.5699022	0.6879534	0	0	1.217473764	0.68457392	*SLC30A5*
Molecular Function	N-acetyltransferase Activity (GO:0008080)	0.5699022	0.6879534	0	0	1.217473764	0.68457392	*EP300*
Molecular Function	Alpha-Actinin Binding (GO:0051393)	0.5699022	0.6879534	0	0	1.217473764	0.68457392	*TTN*
Molecular Function	Calmodulin-Dependent Protein Kinase Activity (GO:0004683)	0.5699022	0.6879534	0	0	1.217473764	0.68457392	*EEF2K*
Molecular Function	Disordered Domain Specific Binding (GO:0097718)	0.5699022	0.6879534	0	0	1.217473764	0.68457392	*DFFB*
Molecular Function	Mannosyltransferase Activity (GO:0000030)	0.5699022	0.6879534	0	0	1.217473764	0.68457392	*TMTC3*
Molecular Function	Protein Tyrosine/Serine/Threonine Phosphatase Activity (GO:0008138)	0.5699022	0.6879534	0	0	1.217473764	0.68457392	*DUSP5*
Molecular Function	Endopeptidase Inhibitor Activity (GO:0004866)	0.5769792	0.6948419	0	0	0.991723567	0.545397457	*PRKAA2;SPOCK2;SPOCK1;ZDHHC17*
Molecular Function	Myosin Binding (GO:0017022)	0.5837873	0.699999	0	0	1.029997857	0.554363963	*RAB14;RAB39B*
Molecular Function	Notch Binding (GO:0005112)	0.5854035	0.699999	0	0	1.164479652	0.623525171	*CUL3*
Molecular Function	SUMO Transferase Activity (GO:0019789)	0.5854035	0.699999	0	0	1.164479652	0.623525171	*PIAS2*
Molecular Function	Nuclear Androgen Receptor Binding (GO:0050681)	0.6003469	0.7134703	0	0	1.115901715	0.569386244	*EP300*
Molecular Function	Phosphatidylinositol-4-Phosphate Binding (GO:0070273)	0.6003469	0.7134703	0	0	1.115901715	0.569386244	*PLEKHA3*
Molecular Function	Cadherin Binding (GO:0045296)	0.6008912	0.7134703	0	0	0.955671973	0.486763317	*USP8;DDX3X;MPRIP;ABI1;CTNNB1;PKN2;CBL;ARGLU1;CRKL;HCFC1;NCK1*
Molecular Function	Metalloexopeptidase Activity (GO:0008235)	0.6133575	0.7214802	0	0	0.973664219	0.47593427	*ERAP1;LNPEP*

Molecular Function	NAD Binding (GO:0051287)	0.6147523	0.7214802	0	0	1.071210014	0.521181995	*SIRT5*
Molecular Function	Inward Rectifier Potassium Channel Activity (GO:0005242)	0.6147523	0.7214802	0	0	1.071210014	0.521181995	*KCNJ3*
Molecular Function	Protein Serine/Threonine/Tyrosine Kinase Activity (GO:0004712)	0.6147523	0.7214802	0	0	1.071210014	0.521181995	*PRKAA2*
Molecular Function	Serotonin Receptor Activity (GO:0099589)	0.6147523	0.7214802	0	0	1.071210014	0.521181995	*DHX8*
Molecular Function	Protein Kinase Activator Activity (GO:0030295)	0.6184385	0.7241301	0	0	0.944868324	0.454063541	*DDX3X;TRAF6;SPRY2*
Molecular Function	Voltage-Gated Monoatomic Ion Channel Activity (GO:0005244)	0.6286393	0.7326899	0	0	1.029956136	0.478103249	*KCND2*
Molecular Function	Lipoprotein Particle Receptor Binding (GO:0070325)	0.6286393	0.7326899	0	0	1.029956136	0.478103249	*RELN*
Molecular Function	G Protein-Coupled Amine Receptor Activity (GO:0008227)	0.6420263	0.744868	0	0	0.9917581	0.439473834	*DHX8*
Molecular Function	Neuropeptide Binding (GO:0042923)	0.6420263	0.744868	0	0	0.9917581	0.439473834	*OPRM1*
Molecular Function	Protein Phosphatase Regulator Activity (GO:0019888)	0.6502444	0.7526802	0	0	0.907464237	0.390578927	*PPP6R2;PPP4R3B*
Molecular Function	Gamma-Tubulin Binding (GO:0043015)	0.6549314	0.7563786	0	0	0.956288496	0.404725051	*DDX3X*
Molecular Function	Metallopeptidase Activity (GO:0008237)	0.656949	0.7569844	0	0	0.891992551	0.374769655	*ERAP1;LNPEP;IDE;MYSM1*
Molecular Function	Endopeptidase Activity (GO:0004175)	0.6647611	0.7642492	0	0	0.907712132	0.37064387	*USP13;USP8;USP37;USP15;USP9X;USP9Y;CASP3;TMPRSS4;ERAP1;IDE;SENP1*
Molecular Function	Retinoid Binding (GO:0005501)	0.6673718	0.7655147	0	0	0.923265071	0.373375722	*ARID4A*
Molecular Function	Growth Factor Receptor Binding (GO:0070851)	0.6766187	0.7705542	0	0	0.863229406	0.337218268	*EPGN;FER;ERAP1*
Molecular Function	Hexosyltransferase Activity (GO:0016758)	0.6766187	0.7705542	0	0	0.863229406	0.337218268	*POGLUT1;FUT9;TMTC3*
Molecular Function	Kinesin Binding (GO:0019894)	0.6793644	0.7705542	0	0	0.892443208	0.345016456	*AP1G1*
Molecular Function	Potassium Ion Transmembrane Transporter Activity (GO:0015079)	0.6793644	0.7705542	0	0	0.892443208	0.345016456	*SLC12A6*
Molecular Function	snoRNA Binding (GO:0030515)	0.6793644	0.7705542	0	0	0.892443208	0.345016456	*PRKDC*
Molecular Function	NADH Dehydrogenase (Ubiquinone) Activity (GO:0008137)	0.7020696	0.793973	0	0	0.836578581	0.295916857	*NDUFC2*
Molecular Function	Cytokine Receptor Binding (GO:0005126)	0.7031438	0.793973	0	0	0.827459777	0.291426278	*USP15;TRAF6;ERAP1*
Molecular Function	Calcium Ion Transmembrane Transporter Activity (GO:0015085)	0.7079845	0.7942771	0	0	0.810922596	0.280038378	*ATP2B2;TPCN2*
Molecular Function	MHC Protein Binding (GO:0042287)	0.7128128	0.7942771	0	0	0.811185569	0.274615907	*MFAP3*
Molecular Function	NADH Dehydrogenase (Quinone) Activity (GO:0050136)	0.7128128	0.7942771	0	0	0.811185569	0.274615907	*NDUFC2*
Molecular Function	Nucleosomal DNA Binding (GO:0031492)	0.7128128	0.7942771	0	0	0.811185569	0.274615907	*RBBP4*
Molecular Function	Sodium Channel Regulator Activity (GO:0017080)	0.7128128	0.7942771	0	0	0.811185569	0.274615907	*TMPRSS4*
Molecular Function	Solute:Sodium Symporter Activity (GO:0015370)	0.7128128	0.7942771	0	0	0.811185569	0.274615907	*SLC4A4*
Molecular Function	Protein Kinase Regulator Activity (GO:0019887)	0.7219252	0.7987946	0	0	0.80251046	0.261484941	*PRKAR2A;CCNG2;NCK1*
Molecular Function	NADP Binding (GO:0050661)	0.7231691	0.7987946	0	0	0.787286264	0.255169113	*HMGCR*
Molecular Function	Inorganic Cation Transmembrane Transporter Activity (GO:0022890)	0.7231691	0.7987946	0	0	0.787286264	0.255169113	*SLC12A6*
Molecular Function	Ion Channel Inhibitor Activity (GO:0008200)	0.7231691	0.7987946	0	0	0.787286264	0.255169113	*RASA1*
Molecular Function	Amino Acid Binding (GO:0016597)	0.7331524	0.8045634	0	0	0.764752633	0.237380503	*SLC7A6*
Molecular Function	Neuropeptide Receptor Activity (GO:0008188)	0.7331524	0.8045634	0	0	0.764752633	0.237380503	*OPRM1*
Molecular Function	Protein Kinase C Binding (GO:0005080)	0.7331524	0.8045634	0	0	0.764752633	0.237380503	*AVPR1B*
Molecular Function	N-acyltransferase Activity (GO:0016410)	0.7427762	0.811394	0	0	0.74347087	0.221078875	*EP300*
Molecular Function	Sodium Channel Activity (GO:0005272)	0.7427762	0.811394	0	0	0.74347087	0.221078875	*TPCN2*

Molecular Function	ATPase Binding (GO:0051117)	0.7441779	0.811394	0	0	0.753619208	0.222675765	*ATXN3;TOR1AIP2*
Molecular Function	Ligand-Gated Monoatomic Cation Channel Activity (GO:0099094)	0.7575436	0.8241944	0	0	0.732895791	0.203506281	*TPCN2;KCNJ3*
Molecular Function	Phospholipase Activity (GO:0004620)	0.7703013	0.83628	0	0	0.713277623	0.186146577	*PLCB4;GDPD1*
Molecular Function	Kinase Binding (GO:0019900)	0.7763942	0.8410938	0	0	0.837396308	0.211940685	*CEP68;ATF2;GSK3B;USP37;STAT3;TPCN2;SNAP91;TTN;NBEA;CTNNB1;SPRY2;ARHGEF7;HNRNPA0;FAM83A*
Molecular Function	Voltage-Gated Potassium Channel Activity (GO:0005249)	0.7883398	0.8522138	0	0	0.685736733	0.163086084	*KCND2;KCNJ3*
Molecular Function	CCR Chemokine Receptor Binding (GO:0048020)	0.8011051	0.8618999	0	0	0.622214316	0.137984189	*CXCL13*
Molecular Function	Kinase Activator Activity (GO:0019209)	0.8011051	0.8618999	0	0	0.622214316	0.137984189	*BCL10*
Molecular Function	Calcium Ion Binding (GO:0005509)	0.8023999	0.8618999	0	0	0.794097921	0.174819193	*SYT4;GLCE;SPOCK2;ITSN1;SPOCK1;ASTN2;RASGRP1;CAMKK2;ADGRL3;TTN*
Molecular Function	Phosphatase Activity (GO:0016791)	0.8051188	0.8627337	0	0	0.660235909	0.143116325	*DUSP5;HDHD2*
Molecular Function	Potassium Channel Regulator Activity (GO:0015459)	0.8082811	0.8627337	0	0	0.608041472	0.129418857	*RASA1*
Molecular Function	Proton Transmembrane Transporter Activity (GO:0015078)	0.8082811	0.8627337	0	0	0.608041472	0.129418857	*SLC11A2*
Molecular Function	Chemokine Activity (GO:0008009)	0.8151984	0.8682891	0	0	0.594498532	0.121470145	*CXCL13*
Molecular Function	Calcium-Dependent Phospholipid Binding (GO:0005544)	0.8218666	0.8735563	0	0	0.581544416	0.114085763	*SYT4*
Molecular Function	Pyrophosphatase Activity (GO:0016462)	0.8344906	0.8851187	0	0	0.557255447	0.10082632	*DDX3X*
Molecular Function	Chemokine Receptor Binding (GO:0042379)	0.8404635	0.8895929	0	0	0.545854503	0.094870438	*CXCL13*
Molecular Function	Polyubiquitin Modification-Dependent Protein Binding (GO:0031593)	0.8571214	0.9034523	0	0	0.514282658	0.079289893	*VCP*
Molecular Function	Serine-Type Endopeptidase Inhibitor Activity (GO:0004867)	0.8571214	0.9034523	0	0	0.514282658	0.079289893	*SPOCK1*
Molecular Function	Oxidoreduction-Driven Active Transmembrane Transporter Activity (GO:0015453)	0.8622786	0.9070026	0	0	0.504552969	0.074763051	*NDUFC2*
Molecular Function	Microtubule Binding (GO:0008017)	0.8643241	0.9072719	0	0	0.68694774	0.100162104	*FBXW11;KIF3A;JMY;HOOK3;MID2;CLASP2*
Molecular Function	Amino Acid Transmembrane Transporter Activity (GO:0015171)	0.8720421	0.9134821	0	0	0.486155013	0.066563175	*SLC7A6*
Molecular Function	G Protein-Coupled Receptor Activity (GO:0004930)	0.8902173	0.9305982	0	0	0.655599945	0.076239549	*DHX8;ELOVL4;ADGRB3;OPRM1;LGR4;ADGRL3*
Molecular Function	UDP-glycosyltransferase Activity (GO:0008194)	0.8935323	0.9321418	0	0	0.445526194	0.050154108	*POGLUT1*
Molecular Function	Tubulin Binding (GO:0015631)	0.8972753	0.9341243	0	0	0.678665999	0.073562374	*DDX3X;KIF3A;JMY;HOOK3;ARHGEF7;MID2;APPL1;CLASP2*
Molecular Function	L-amino Acid Transmembrane Transporter Activity (GO:0015179)	0.9010827	0.9361658	0	0	0.431109516	0.044903612	*SLC7A6*
Molecular Function	Phosphatidylinositol-4,5-Bisphosphate Binding (GO:0005546)	0.9315305	0.96582	0	0	0.371040025	0.026316515	*SNAP91*
Molecular Function	Hormone Activity (GO:0005179)	0.9340044	0.9664086	0	0	0.365938232	0.024984132	*ADIPOQ*
Molecular Function	G Protein-Coupled Peptide Receptor Activity (GO:0008528)	0.9409031	0.9695892	0	0	0.351438401	0.021407918	*AVPR1B*
Molecular Function	Phosphotransferase Activity, Alcohol Group As Acceptor (GO:0016773)	0.9409031	0.9695892	0	0	0.351438401	0.021407918	*ETNK1*
Molecular Function	Potassium Channel Activity (GO:0005267)	0.9450973	0.9710375	0	0	0.342391498	0.019333962	*KCND2*
Molecular Function	Protein Homodimerization Activity (GO:0042803)	0.9461391	0.9710375	0	0	0.698655816	0.038681565	*PDCD6IP;ZHX3;CARNMT1;ABCB7;GLCE;STAT3;ADIPOQ;RNF8;TMEM192;IDE;SPPL3;MID2;LRP6;QTRT2;TFAP4;APPL1;RBM44*
Molecular Function	Growth Factor Activity (GO:0008083)	0.9559788	0.979154	0	0	0.317835618	0.01430882	*EPGN*
Molecular Function	Metal Ion Binding (GO:0046872)	0.9653953	0.9868053	0	0	0.625054111	0.022012922	*SYT4;GLCE;SPOCK2;ITSN1;DHX36;SPOCK1;ASTN2;PRKACB;RASGRP1;ADGRL3;TTN;CAMKK2*

Molecular Function	Oxidoreductase Activity, Acting On The CH-OH Group Of Donors, NAD Or NADP As Acceptor (GO:0016616)	0.9695412	0.989049	0	0	0.283875359	0.008780927	*HMGCR*
Molecular Function	Metalloendopeptidase Activity (GO:0004222)	0.9755822	0.9906526	0	0	0.266759388	0.006594524	*IDE*
Molecular Function	Magnesium Ion Binding (GO:0000287)	0.9772493	0.9906526	0	0	0.348225827	0.008013896	*DHX36;PRKACB*
Molecular Function	Carboxylic Ester Hydrolase Activity (GO:0052689)	0.9781378	0.9906526	0	0	0.258949188	0.005723992	*GDPD1*
Molecular Function	Monoatomic Cation Channel Activity (GO:0005261)	0.9789289	0.9906526	0	0	0.256445918	0.005461342	*TPCN2*
Molecular Function	Serine-Type Endopeptidase Activity (GO:0004252)	0.989921	0.9985409	0	0	0.214859348	0.002176549	*TMPRSS4*
Molecular Function	Actin Binding (GO:0003779)	0.9906628	0.9985409	0	0	0.29392573	0.002757322	*HNRNPU;SORBS1*
Molecular Function	Serine-Type Peptidase Activity (GO:0008236)	0.9946176	0.9999948	0	0	0.188786632	0.001018878	*TMPRSS4*
Molecular Function	Cytokine Activity (GO:0005125)	0.9985754	0.9999948	0	0	0.150106472	2.14E-04	*CXCL13*
Molecular Function	Receptor Ligand Activity (GO:0048018)	0.9993447	0.9999948	0	0	0.251098988	1.65E-04	*EPGN;NRG4;CXCL13*
Molecular Function	Olfactory Receptor Activity (GO:0004984)	0.9999948	0.9999948	0	0	0.072889016	3.76E-07	*OR5B2*

hsa-miR-5689

Gene ontology (GO)	Term	P-value	Adjusted P-value	Old P-value	Old Adjusted P-value	Odds Ratio	Combined Score
Biological process	Regulation Of Histamine Secretion By Mast Cell (GO:1903593)	0.002098387	0.033404266	0	0	666.2666667	4108.590826
Biological process	Regulation Of Protein Localization To Cell Cortex (GO:1904776)	0.002447753	0.033404266	0	0	555.1944444	3338.153583
Biological process	Glucan Metabolic Process (GO:0044042)	0.002797015	0.033404266	0	0	475.8571429	2797.6605
Biological process	Positive Regulation Of Receptor Recycling (GO:0001921)	0.003146172	0.033404266	0	0	416.3541667	2398.853181
Biological process	SNARE Complex Assembly (GO:0035493)	0.003146172	0.033404266	0	0	416.3541667	2398.853181
Biological process	Cellular Response To Forskolin (GO:1904322)	0.003495224	0.033404266	0	0	370.0740741	2093.271349
Biological process	Response To Forskolin (GO:1904321)	0.003495224	0.033404266	0	0	370.0740741	2093.271349
Biological process	Negative Regulation Of Secretion (GO:0051048)	0.004541754	0.033404266	0	0	277.5138889	1497.032601
Biological process	Regulation Of Hormone Secretion (GO:0046883)	0.005238917	0.033404266	0	0	237.8452381	1249.077685
Biological process	Regulation Of Receptor Recycling (GO:0001919)	0.005935662	0.033404266	0	0	208.09375	1066.850192
Biological process	Regulation Of cAMP-mediated Signaling (GO:0043949)	0.006283878	0.033404266	0	0	195.8431373	992.8792721
Biological process	Energy Reserve Metabolic Process (GO:0006112)	0.006283878	0.033404266	0	0	195.8431373	992.8792721
Biological process	Glycogen Metabolic Process (GO:0005977)	0.006631989	0.033404266	0	0	184.9537037	927.700129
Biological process	Regulation Of Spindle Organization (GO:0090224)	0.006979996	0.033404266	0	0	175.2105263	869.8689178
Biological process	Organelle Membrane Fusion (GO:0090174)	0.009065844	0.036612822	0	0	133.12	626.0954814
Biological process	Regulation Of Immunoglobulin Production (GO:0002637)	0.009413121	0.036612822	0	0	127.9935897	597.1733894
Biological process	Membrane Fusion (GO:0061025)	0.009760292	0.036612822	0	0	123.2469136	570.5633183
Biological process	Positive Regulation Of Immunoglobulin Production (GO:0002639)	0.011841135	0.036612822	0	0	100.8080808	447.2023641
Biological process	Substrate Adhesion-Dependent Cell Spreading (GO:0034446)	0.012533917	0.036612822	0	0	95.03809524	416.2019442
Biological process	Positive Regulation Of Signaling (GO:0023056)	0.012880151	0.036612822	0	0	92.39351852	402.1028593
Biological process	Positive Regulation Of Production Of Molecular Mediator Of Immune Response (GO:0002702)	0.013226282	0.036612822	0	0	89.89189189	388.8318192
Biological process	Response To Peptide (GO:1901652)	0.013226282	0.036612822	0	0	89.89189189	388.8318192
Biological process	Positive Regulation Of Cytokinesis (GO:0032467)	0.013572308	0.036612822	0	0	87.52192982	376.3201193
Biological process	Regulation Of Mitotic Spindle Organization (GO:0060236)	0.013572308	0.036612822	0	0	87.52192982	376.3201193
Biological process	Negative Regulation Of Secretion By Cell (GO:1903531)	0.01391823	0.036612822	0	0	85.27350427	364.5063483
Biological process	Monoatomic Cation Transport (GO:0006812)	0.014609764	0.036612822	0	0	81.10569106	342.7579416

Biological process	Positive Regulation Of Cell Division (GO:0051781)	0.014955374	0.036612822	0	0	79.17063492	332.7292055
Biological process	Cellular Response To Ketone (GO:1901655)	0.015300881	0.036612822	0	0	77.3255814	323.208935
Biological process	Positive Regulation Of Protein Localization To Cell Periphery (GO:1904377)	0.017716522	0.039780215	0	0	66.47666667	268.1175239
Biological process	Adenylate Cyclase-Inhibiting G Protein-Coupled Receptor Signaling Pathway (GO:0007193)	0.018061198	0.039780215	0	0	65.16993464	261.5914255
Biological process	Cellular Response To Alcohol (GO:0097306)	0.018405771	0.039780215	0	0	63.91346154	255.3400953
Biological process	Response To Peptide Hormone (GO:0043434)	0.019094606	0.039979332	0	0	61.54012346	243.5973091
Biological process	Regulation Of Secretion By Cell (GO:1903530)	0.02218924	0.045050881	0	0	52.72486772	200.7840888
Biological process	Cell-Cell Junction Organization (GO:0045216)	0.023561944	0.046430891	0	0	49.56716418	185.7837979
Biological process	Cell-Cell Junction Assembly (GO:0007043)	0.024590389	0.04707303	0	0	47.43571429	175.7682775
Biological process	Vesicle Fusion (GO:0006906)	0.027328369	0.048786849	0	0	42.5534188	153.1850717
Biological process	Calcium Ion Transmembrane Transport (GO:0070588)	0.027670153	0.048786849	0	0	42.01265823	150.7162502
Biological process	Regulation Of Cytokinesis (GO:0032465)	0.027670153	0.048786849	0	0	42.01265823	150.7162502
Biological process	Exocytosis (GO:0006887)	0.029718696	0.051055196	0	0	39.03529412	137.247272
Biological process	Retrograde Transport, Endosome To Golgi (GO:0042147)	0.032444332	0.052299202	0	0	35.66308244	122.2612325
Biological process	Vesicle Organization (GO:0016050)	0.032444332	0.052299202	0	0	35.66308244	122.2612325
Biological process	Cell Junction Assembly (GO:0034329)	0.032784574	0.052299202	0	0	35.28191489	120.5864291
Biological process	Extracellular Structure Organization (GO:0043062)	0.037537214	0.057401175	0	0	30.6867284	100.7268068
Biological process	External Encapsulating Structure Organization (GO:0045229)	0.037875921	0.057401175	0	0	30.40366972	99.52457965
Biological process	Calcium Ion Transport (GO:0006816)	0.038553028	0.057401175	0	0	29.85285285	97.19254886
Biological process	Positive Regulation Of Cell Cycle Process (GO:0090068)	0.040581901	0.059108421	0	0	28.31339031	90.72836534
Biological process	Protein-Containing Complex Organization (GO:0043933)	0.043281355	0.060883917	0	0	26.49066667	83.18157654
Biological process	Cytosolic Transport (GO:0016482)	0.043618328	0.060883917	0	0	26.27910053	82.31344422
Biological process	Regulation Of Cell Adhesion (GO:0030155)	0.049331335	0.06745305	0	0	23.13519814	69.61834103
Biological process	Cellular Response To Organic Cyclic Compound (GO:0071407)	0.052009671	0.069692959	0	0	21.90066225	64.74548832
Biological process	Adenylate Cyclase-Modulating G Protein-Coupled Receptor Signaling Pathway (GO:0007188)	0.055681821	0.073150628	0	0	20.40226337	58.92380851
Biological process	Membrane Organization (GO:0061024)	0.057014122	0.073460504	0	0	19.90662651	57.0216613
Biological process	Positive Regulation Of Hydrolase Activity (GO:0051345)	0.06000591	0.074451778	0	0	18.87428571	53.09925853
Biological process	Extracellular Matrix Organization (GO:0030198)	0.06000591	0.074451778	0	0	18.87428571	53.09925853
Biological process	Endosomal Transport (GO:0016197)	0.06133298	0.074714721	0	0	18.44878957	51.49864427
Biological process	Regulation Of GTPase Activity (GO:0043087)	0.072548387	0.086798963	0	0	15.47730829	40.60474198
Biological process	Cellular Response To Lipid (GO:0071396)	0.077132998	0.090665103	0	0	14.51248164	37.18423022

Biological process	Positive Regulation Of GTPase Activity (GO:0043547)	0.079091877	0.091364754	0	0	14.13447783	35.86122126
Biological process	Regulation Of Inflammatory Response (GO:0050727)	0.08104719	0.09203664	0	0	13.77545328	34.61390792
Biological process	Cellular Component Assembly (GO:0022607)	0.087539225	0.097752134	0	0	12.6988417	30.93016624
Biological process	Monoatomic Cation Transmembrane Transport (GO:0098655)	0.094313513	0.102961429	0	0	11.73392857	27.70534021
Biological process	Inorganic Cation Transmembrane Transport (GO:0098662)	0.09527774	0.102961429	0	0	11.60777385	27.28940126
Biological process	Regulation Of Intracellular Signal Transduction (GO:1902531)	0.099445898	0.105759923	0	0	11.09065315	25.59879707
Biological process	Protein-Containing Complex Assembly (GO:0065003)	0.109318985	0.114443312	0	0	10.02344546	22.18674824
Biological process	Positive Regulation Of Macromolecule Metabolic Process (GO:0010604)	0.12066794	0.1243808	0	0	9.012855831	19.05960162
Biological process	Vesicle-Mediated Transport (GO:0016192)	0.135297921	0.13734789	0	0	7.960569106	15.9233362
Biological process	Negative Regulation Of Cellular Process (GO:0048523)	0.173493773	0.173493773	0	0	6.050062189	10.59737103
Molecular Function	5'-Nucleotidase Activity (GO:0008253)	0.004193015	0.037248945	0	0	302.7575758	1657.396457
Molecular Function	Nucleotidase Activity (GO:0008252)	0.004890388	0.037248945	0	0	256.1538462	1362.86237
Molecular Function	Syntaxin-1 Binding (GO:0017075)	0.005587342	0.037248945	0	0	221.9777778	1151.454594
Molecular Function	Monoatomic Cation Transmembrane Transporter Activity (GO:0008324)	0.012880151	0.051520604	0	0	92.39351852	402.1028593
Molecular Function	Monoatomic Ion Channel Activity (GO:0005216)	0.012880151	0.051520604	0	0	92.39351852	402.1028593
Molecular Function	Syntaxin Binding (GO:0019905)	0.018405771	0.058904861	0	0	63.91346154	255.3400953
Molecular Function	GDP Binding (GO:0019003)	0.023218923	0.058904861	0	0	50.32070707	189.3461363
Molecular Function	Calcium Ion Transmembrane Transporter Activity (GO:0015085)	0.023561944	0.058904861	0	0	49.56716418	185.7837979
Molecular Function	Calcium Channel Activity (GO:0005262)	0.031422988	0.065784298	0	0	36.85740741	127.5345741
Molecular Function	Hexosyltransferase Activity (GO:0016758)	0.033124714	0.065784298	0	0	34.90877193	118.9507894
Molecular Function	Monoatomic Cation Channel Activity (GO:0005261)	0.036181364	0.065784298	0	0	31.87339744	105.7945348
Molecular Function	G Protein-Coupled Receptor Binding (GO:0001664)	0.047989737	0.079982895	0	0	23.8057554	72.29255881
Molecular Function	Magnesium Ion Binding (GO:0000287)	0.053012378	0.081557505	0	0	21.47077922	63.06461335
Molecular Function	GTP Binding (GO:0005525)	0.068273783	0.097533976	0	0	16.49416667	44.27412773
Molecular Function	Guanyl Ribonucleotide Binding (GO:0032561)	0.076479245	0.101972327	0	0	14.64296296	37.64319024
Molecular Function	GTPase Activity (GO:0003924)	0.089156077	0.106788791	0	0	12.45517677	30.10873048
Molecular Function	Ribonucleoside Triphosphate Phosphatase Activity (GO:0017111)	0.090770473	0.106788791	0	0	12.22057001	29.32229522
Molecular Function	Cadherin Binding (GO:0045296)	0.10646221	0.118291344	0	0	10.31184486	23.09817358
Molecular Function	Purine Ribonucleoside Triphosphate Binding (GO:0035639)	0.15518679	0.163354516	0	0	6.848421053	12.75946987
Molecular Function	Metal Ion Binding (GO:0046872)	0.16872516	0.16872516	0	0	6.241346154	11.10637663

Genes

VAMP3

GNAI1

GBE1

VAMP3

VAMP3

GNAI1

GNAI1

VAMP3

VAMP3

VAMP3

GNAI1

GBE1

GBE1

GNAI1

VAMP3

VAMP3

VAMP3

VAMP3

VAMP3

VAMP3

VAMP3

GNAI1

PKP4

GNAI1

VAMP3

TRPM6

PKP4

GNAI1

GNAI1

GNAI1

GNAI1

GNAI1

VAMP3

PKP4

PKP4

VAMP3
TRPM6

PKP4

VAMP3
VAMP3

VAMP3

PKP4

MATN2

MATN2

TRPM6

PKP4

VAMP3

VAMP3

PKP4

GNAI1

GNAI1

VAMP3

PKP4

MATN2

VAMP3

PKP4

GNAI1

PKP4

VAMP3

VAMP3

TRPM6

TRPM6

GNAI1

VAMP3

VAMP3

VAMP3

VAMP3

NT5DC1

NT5DC1

VAMP3

TRPM6

TRPM6

VAMP3

GNAI1
TRPM6

TRPM6

GBE1

TRPM6

GNAI1

GNAI1

GNAI1
GNAI1

GNAI1

GNAI1

PKP4

GNAI1

GNAI1
